# Using Terpene Synthase Plasticity in Catalysis: On the Enzymatic Conversion of Synthetic Farnesyl Diphosphate Analogues

**DOI:** 10.1002/chem.202103049

**Published:** 2021-09-29

**Authors:** Anwei Hou, Jeroen S. Dickschat

**Affiliations:** ^1^ Kekulé-Institute for Organic Chemistry and Biochemistry University of Bonn Gerhard-Domagk-Straße 1 53121 Bonn Germany

**Keywords:** biosynthesis, enzymes, terpenoids, configuration determination, substrate analogues

## Abstract

Four synthetic farnesyl diphosphate analogues were enzymatically converted with three bacterial sesquiterpene synthases, including β‐himachalene synthase (HcS) and (*Z*)‐γ‐bisabolene synthase (BbS) from *Cryptosporangium arvum*, and germacrene A synthase (SmTS6) from *Streptomyces mobaraensis*. These enzyme reactions not only yielded several previously unknown compounds, showing that this approach opened the door to a new chemical space, but substrates with blocked or altered reactivities also gave interesting insights into the cyclisation mechanisms and the potential to catalyse reactions with different initial cyclisation modes.

## Graphical abstract

**Four synthetic FPP analogues were converted** by three robust bacterial sesquiterpene synthases, yielding previously unknown compounds with high stereoselectivity, which demonstrates that this approach can open the doors to chemical terra incognita. The altered or partially blocked reactivity in the substrates also gave interesting insights into the enzyme mechanisms which adds to our understanding of terpene biosynthesis.

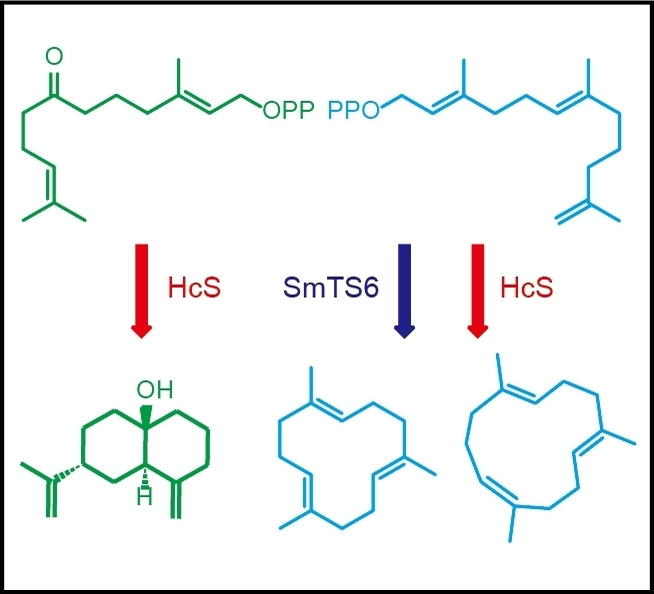

## Introduction

Terpenoids are the largest class of natural products and are widely distributed in all kingdoms of life where they fulfill diverse biological functions.^[1]^ Their structural diversity is controlled by terpene synthases (TPSs), which catalyse the conversion of acyclic oligoprenyl diphosphates into structurally complex terpene hydrocarbons or alcohols.[^2^, ^3^] Monoterpene synthases (MTPSs) can convert geranyl diphosphate (C_10_, GPP) into monoterpenes,^[4]^ sesquiterpene synthases (STPSs) catalyse the transformation of farnesyl diphosphate (C_15_, FPP) into sesquiterpenes,^[5]^ and diterpenes synthases (DTPSs) and sesterterpene synthases (StTPSs) can use geranylgeranyl diphosphate (C_20_, GGPP) and geranylfarnesyl diphosphate (C_25_, GFPP) to form diterpenes and sesterterpenes, respectively.[^6^, ^7^]

**Scheme 1:**
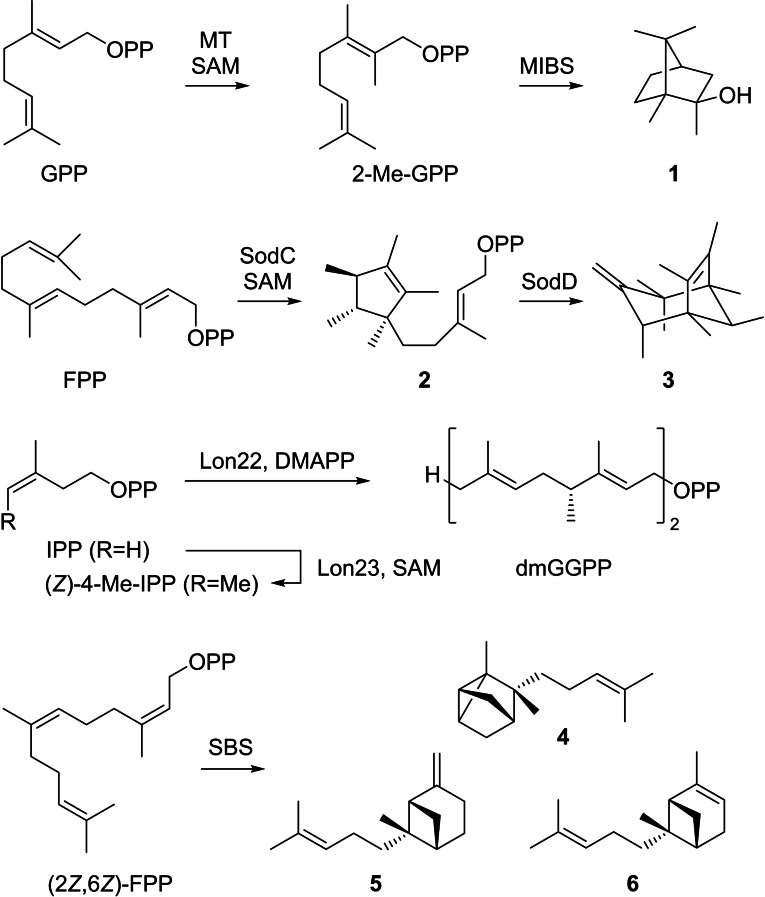
Non‐canonical terpenoid biosynthesis.

Besides these regular cases, some non‐canonical TPSs naturally accept modified substrates, e. g. the biosynthesis of 2‐methylisoborneol (**1**) proceeds through methylation of GPP to 2‐Me‐GPP, followed by cyclisation by the 2‐methylisoborneol synthase (MIBS, Scheme 1).[^8^, ^9^, ^10^] Other recently described examples include the biosynthesis of sodorifen (**3**) that starts with a methylation induced cyclisation of FPP to presodorifen diphosphate (**2**) by SodC followed by conversion into **3** by SodD,^[11]^ or the biosynthesis of longestin involving methylation of IPP to (*Z*)‐4‐methyl‐IPP by Lon23 and specific incorporation into (4*R*,12*R*)‐4,12‐dimethyl‐GGPP (dmGGPP) by Lon22.^[12]^ Furthermore, a santalene and bergamotene synthase (SBS) from the wild tomato *Solanum habrochaites* has been reported that naturally converts (2*Z*,6*Z*)‐FPP into a mixture of sesquiterpenes including (+)‐α‐santalene (**4**), (+)‐*endo*‐β‐bergamotene (**5**), and (−)‐*endo*‐α‐bergamotene (**6**).^[13]^

**Scheme 2:**
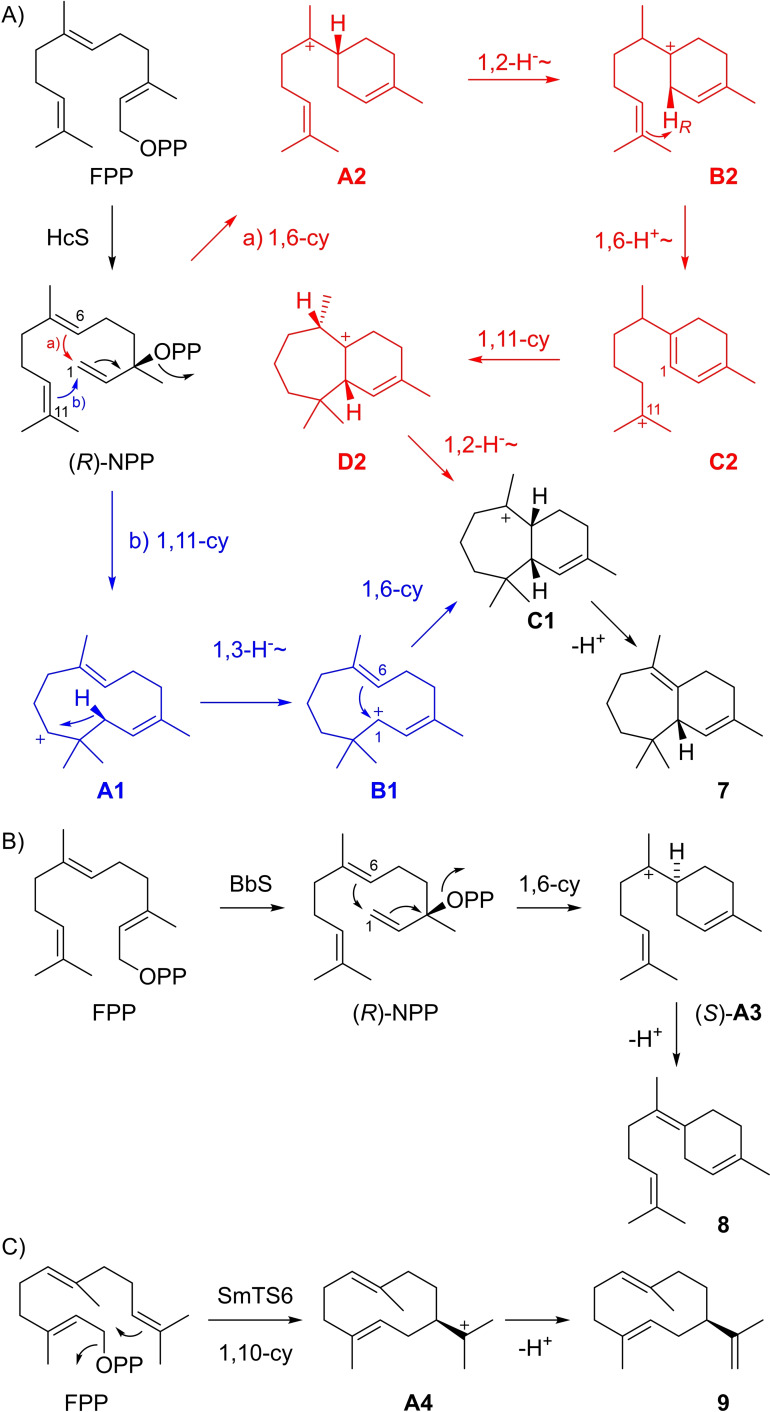
Cyclisation reactions with FPP. Cyclisation to A) β‐himachalene (**7**) by HcS, B) (*Z*)‐γ‐bisabolene (**8**) by BbS, and C) germacrene A (**9**) by SmTS6.

These natural systems raise the question whether also canonical TPSs have the potential to convert substrates other than the regular oligoprenyl diphosphates. Recent research has demonstrated that this is indeed the case,^[14]^ revealing that e. g. halogenated substrates,[^15^, ^16^] substrates with additional or missing Me groups or altered methylation pattern,[^16^, ^17^, ^18^, ^19^] functional groups attached to[^20^, ^21^] or heteroatoms inserted into the chain,^[22]^ with hydrogenated double bonds,[^23^, ^24^] or stereoisomers with *Z*‐configured olefins such as (2*Z*,6*E*)‐FPP can be converted.[^25^, ^26^] The substrate modifications can block reactivity, as for compounds with hydrogenated olefins, or open new reaction pathways as for compounds with altered methylation patterns in which cations can be stabilised at different carbons of the isoprenoid chain. Also functional groups may directly engage in the TPS catalysed reaction. Here we report on the synthesis of four new FPP analogues and their enzymatic conversion with β‐himachalene synthase (HcS)^[27]^ and (*Z*)‐γ‐bisabolene synthase (BbS)^[28]^ from *Cryptosporangium arvum*, and germacrene A synthase (SmTS6) from *Streptomyces mobaraensis*.^[29]^ For HcS cyclisation cascades from FPP to β‐himachalene (**7**) with initial 1,11‐ (blue path in Scheme 2A) or 1,6‐cyclisation (red path) can be formulated. The co‐occurrence of side products arising by 1,11‐cyclisation (9‐*epi*‐β‐caryophyllene and γ‐humulene) with simultaneous absence of any 1,6‐cyclised products may favour the pathway through initial 1,11‐cyclisation for **7**.^[27]^ BbS catalyses a 1,6‐cyclisation of FPP via nerolidyl diphosphate (NPP) and the bisabolyl cation (**A 3**) into (*Z*)‐γ‐bisabolene (**8**, Scheme 2B). Previous incubation experiments with (*R*)‐ and (*S*)‐NPP have demonstrated that this process involves the intermediates (*R*)‐NPP and (*S*)‐**A 3**.^[28]^ SmTS6 converts FPP through 1,10‐cyclisation to the (*E*,*E*)‐germacradienyl cation **A 4** into germacrene A (**9**, Scheme 2C).

## Results and Discussion

**Scheme 3:**
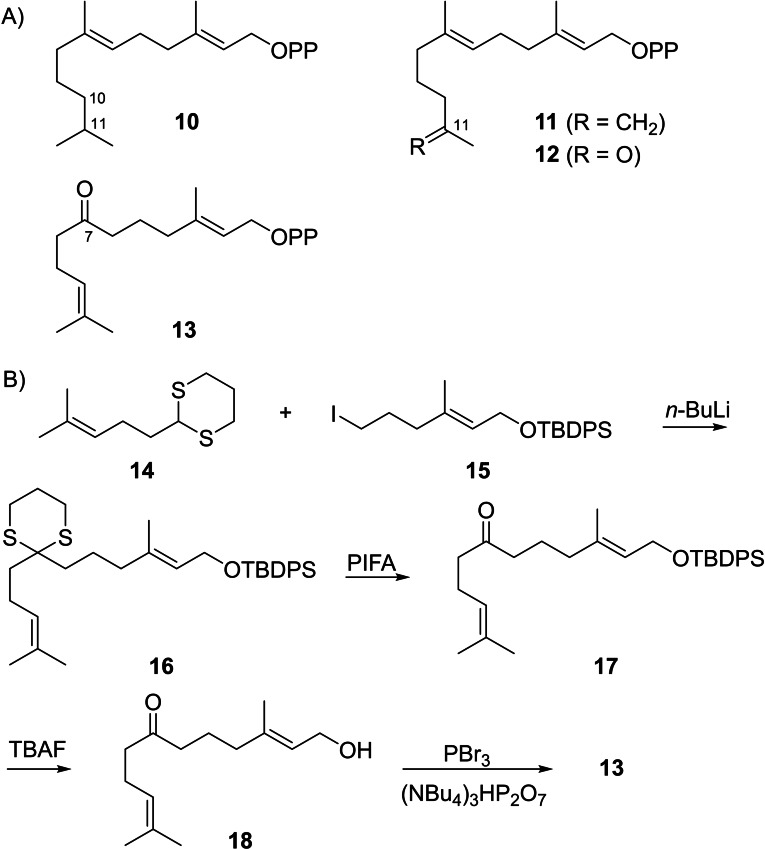
A) FPP analogues **10**–**13** used in this study. B) Synthesis of FPP analogue **13**, for reaction conditions cf. Scheme S1.

For investigations with the selected enzymes the FPP analogues **10**–**13** were designed. Substrate **10** has a saturated bond instead of the terminal double bond of FPP, in analogue **11** the position of the terminal double bond is shifted, and in substrates **12** and **13** a methyl group at the middle or the terminal double bond of FPP is removed and exchanged by a ketone group (Scheme 3A). We hypothesised that substrate **10** may be converted smoothly with BbS, but cannot react in a 1,10‐cyclisation and may thus only give acyclic products with SmTS6. With HcS further insights into the question of initial 1,6‐ versus 1,11‐cyclisation may be obtained. Analogues **11** and **12** could undergo the usual 1,6‐cyclisation with BbS, but with SmTS6 and HcS new reaction paths may be opened. Finally, with **13** new reaction paths may be observed with BbS, while with SmTS6 a 1,10‐cyclisation could still be possible, and for HcS the result may depend on the question of initial 1,6‐ or 1,11‐cyclisation, potentially leading to new reaction paths. The FPP analogues **10**–**12** were synthesised as reported previously,[^19^, ^30^] and **13** was synthesised through Corey‐Seebach umpolung from 1,3‐dithiane **14** and iodide **15** to yield **16** (Scheme 3B). Deprotection via **17** to **18** followed by bromination and phosphorylation yielded **13**.

**Scheme 4:**
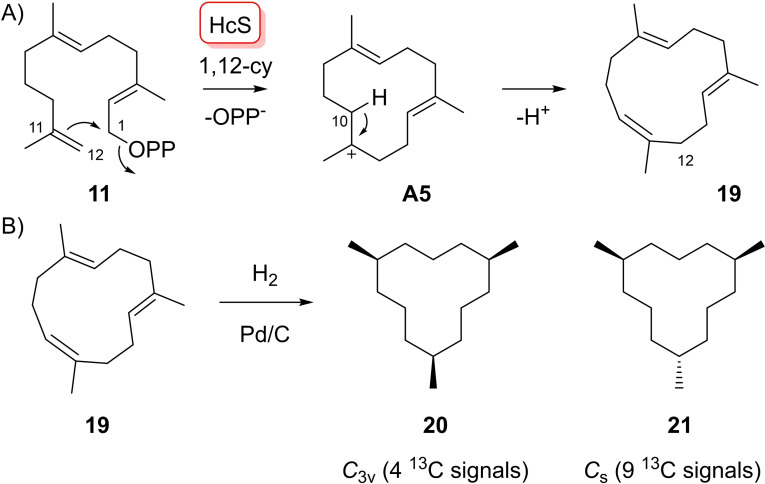
A) Enzymatic conversion of FPP analogue **11** with HcS. B) Catalytic hydrogenation of **19**.

In a first series of experiments, **10**–**13** were enzymatically converted with HcS (Figures S1 and S2).^[27]^ This enzyme did not accept **10** as substrate, suggesting that HcS does not catalyse an initial 1,6‐cyclisation, but proceeds through 1,11‐cyclisation, in agreement with our previous report.^[27]^ With substrate **11** one major product **19** was obtained (Scheme 4A) that was isolated and characterised as (1*Z*,5*E*,9*E*)‐1,5,9‐trimethylcyclododeca‐1,5,9‐triene (**19**) by NMR spectroscopy (Table S1, Figures S3–S10). The ^1^H NMR spectrum of **19** showed line broadening for all CH_2_ groups, pointing to slowly interconverting conformers, but sharp signals in the ^13^C NMR spectrum. As a result of line broadening, signals for the hydrogens attached to C12 were missing in all ^1^H based spectra. Therefore, to secure the structure of **19** a catalytic hydrogenation was performed that yielded an inseparable mixture of the two possible diastereomers, *C*_3v_ symmetric **20** and *C*_s_ symmetric **21** (Scheme 4B), that were observed by ^13^C NMR and GC/MS (Figures S11 and S12). Compound **19** can be formed from **11** through a newly opened reaction path that is not possible for FPP, i. e. by 1,12‐cyclisation, leading to a cationic intermediate with a tertiary cation at C11 in **A5**, followed by deprotonation to **19** (Scheme 4A).

**Scheme 5:**
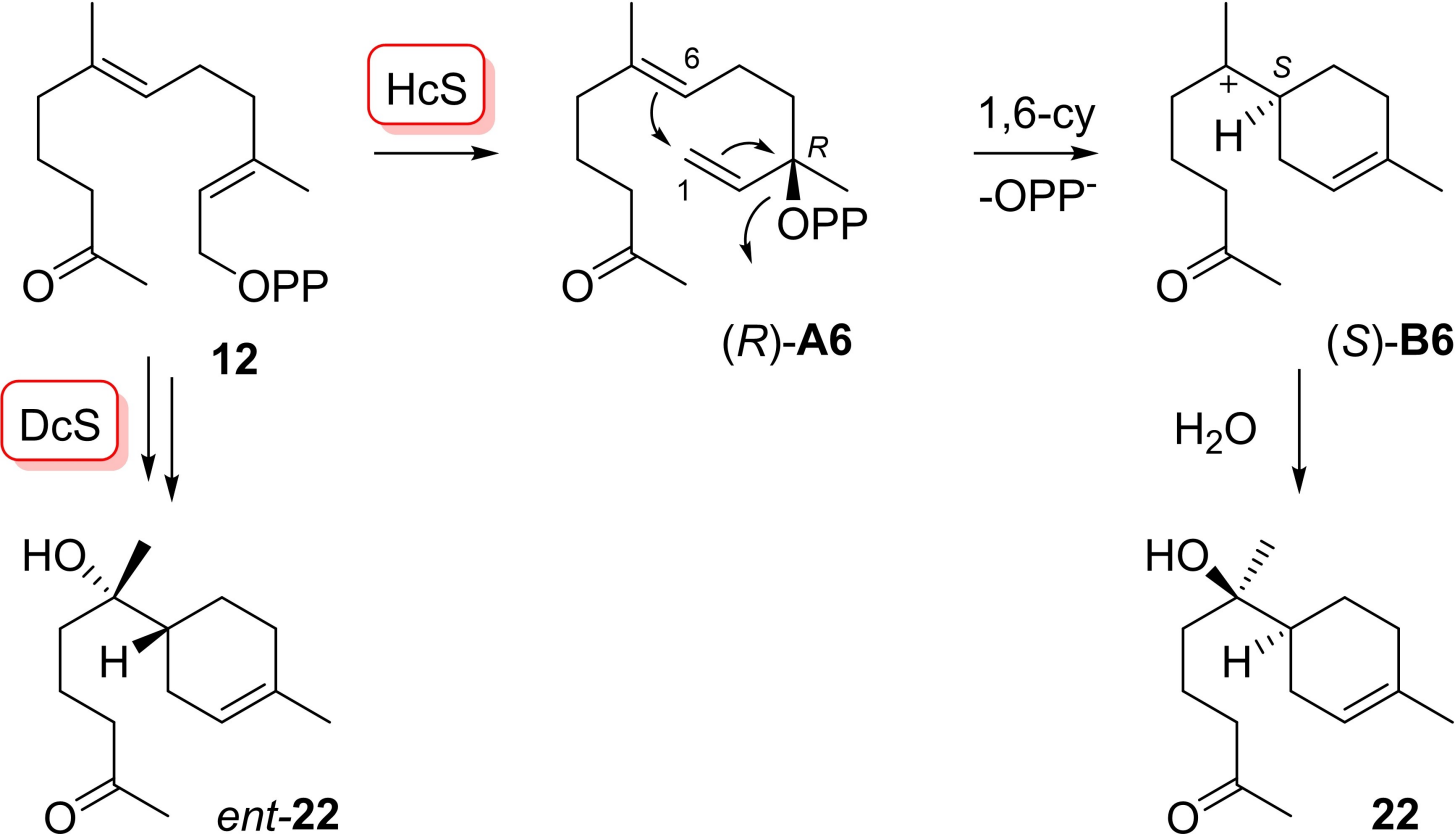
Enzymatic conversion of FPP analogue **12** with HcS.

Substrate **12** was converted by HcS into multiple products. The main compound was isolated and identified as **22** ([α]_D_^25^=−16.2, *c* 0.21, CH_2_Cl_2_), the enantiomer of *ent*‐**22** ([α]_D_^20^=+51.7, *c* 0.12, CH_2_Cl_2_) that we had previously obtained from **12** with dauc‐8‐en‐11‐ol synthase (DcS) from *Streptomyces venezuelae* (Scheme 5).^[19]^ The formation of **22** requires isomerisation to (*R*)‐**A6** and subsequent 1,6‐cyclisation by *anti*‐S_N_2’ reaction, resulting in (*S*)‐**B 6**. Final attack of water yields **22**. These results show that HcS can also catalyse a 1,6‐cyclisation with substrate **12**.

**Scheme 6:**
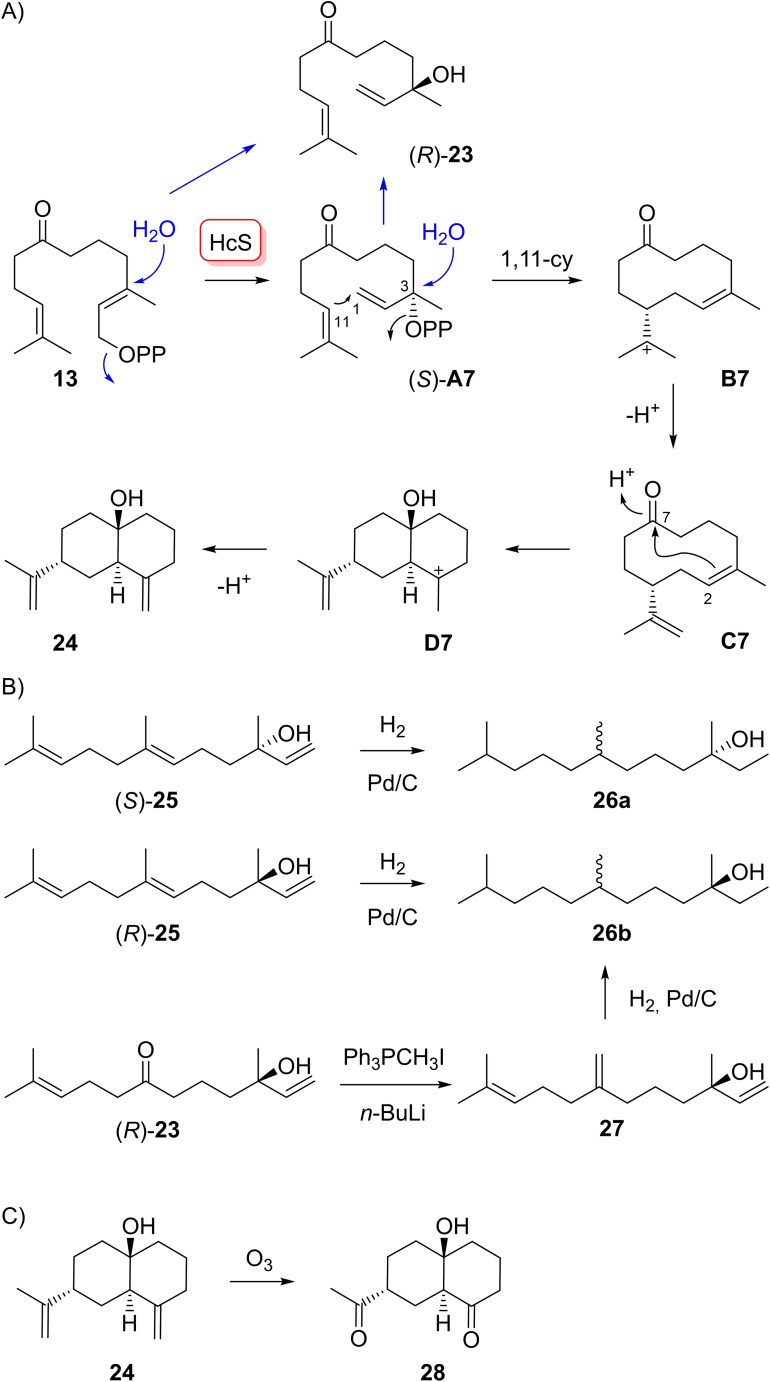
A) Enzymatic conversion of FPP analogue **13** with HcS. B) Correlation of (*R*)‐**23** with both enantiomers of nerolidol (**25**). C) Degradation of **24** by ozonolysis.

FPP analogue **13** yielded with HcS compounds **23** and **24** (Scheme 6A). Both compounds were isolated and their structures elucidated by NMR spectroscopy (Tables S2 and S3, Figures S13–S28). The absolute configuration of **23** was determined by chemical correlation with both enantiomers of nerolidol (**25**, Scheme 6B). Therefore, (*S*)‐ and (*R*)‐**25** were converted by catalytic hydrogenation, yielding silica gel chromatographically inseparable diastereoisomeric mixtures of (3*R*,7*RS*)‐**26 a** and (3*S*,7*RS*)‐**26 b** that were also inseparable by GC on a chiral stationary phase (Figure S29). However, the retention times for **26 a** and **26 b** were clearly different, allowing to conclude on the configuration at C3. Compound **23** was converted by Wittig reaction into **27** (Table S4, Figures S30–S37) followed by catalytic hydrogenation that yielded a sample identical to **26 b** with minor formation of **26 a** (3 %). Thus, the enzyme product is (*R*)‐**23** (94 % *ee*, Figure S29). Its formation can be explained from **13** either directly or by isomerisation to (*S*)‐**A 7** and hydrolysis with inversion of configuration at C3.

The formation of **24** can be explained by 1,11‐cyclisation of **A 7** to **B 7** with *anti*‐S_N_2’ attack at C1. A reprotonation induced cyclisation of **C 7** to **D 7** and deprotonation yields **24**. If (*S*)‐**A 7** is a common intermediate for both products **23** and **24**, the shown absolute configuration of **24** may result (Scheme 6A). Its ozonolysis to **28** (Table S5, Figures S38–S45) was followed by trials to convert this compound into the bis‐dinitrophenylhydrazone for crystallisations. Unfortunately, this approach was not successful and clarification of the absolute configuration of **28** is open.

In a second series of experiments, the substrate analogues **10**–**13** were enzymatically converted with BbS (Scheme 7, Figures S46–S47).^[28]^ The FPP analogues **10**–**12** can be converted through 1,6‐cyclisation into compounds **29**–**31** (Tables S6–S8, Figures S48–S71), while **13** yielded the acyclic product **23**. These reactions proceed with similar efficiency as observed for the native substrate FPP, showing that the structural modifications did not influence acceptance by BbS. Based on the cyclisation mechanism of BbS with FPP, (*R*)‐**A 7** in analogy to (*R*)‐NPP may be the intermediate which should further react by abstraction of diphosphate and S_N_2 attack of water with inversion of configuration to yield (*S*)‐**23**. The absolute configuration of **23** was confirmed by analysis on a chiral GC column, compared to (*R*)‐**23** obtained from **13** with HcS (Figure S72).

**Scheme 7:**
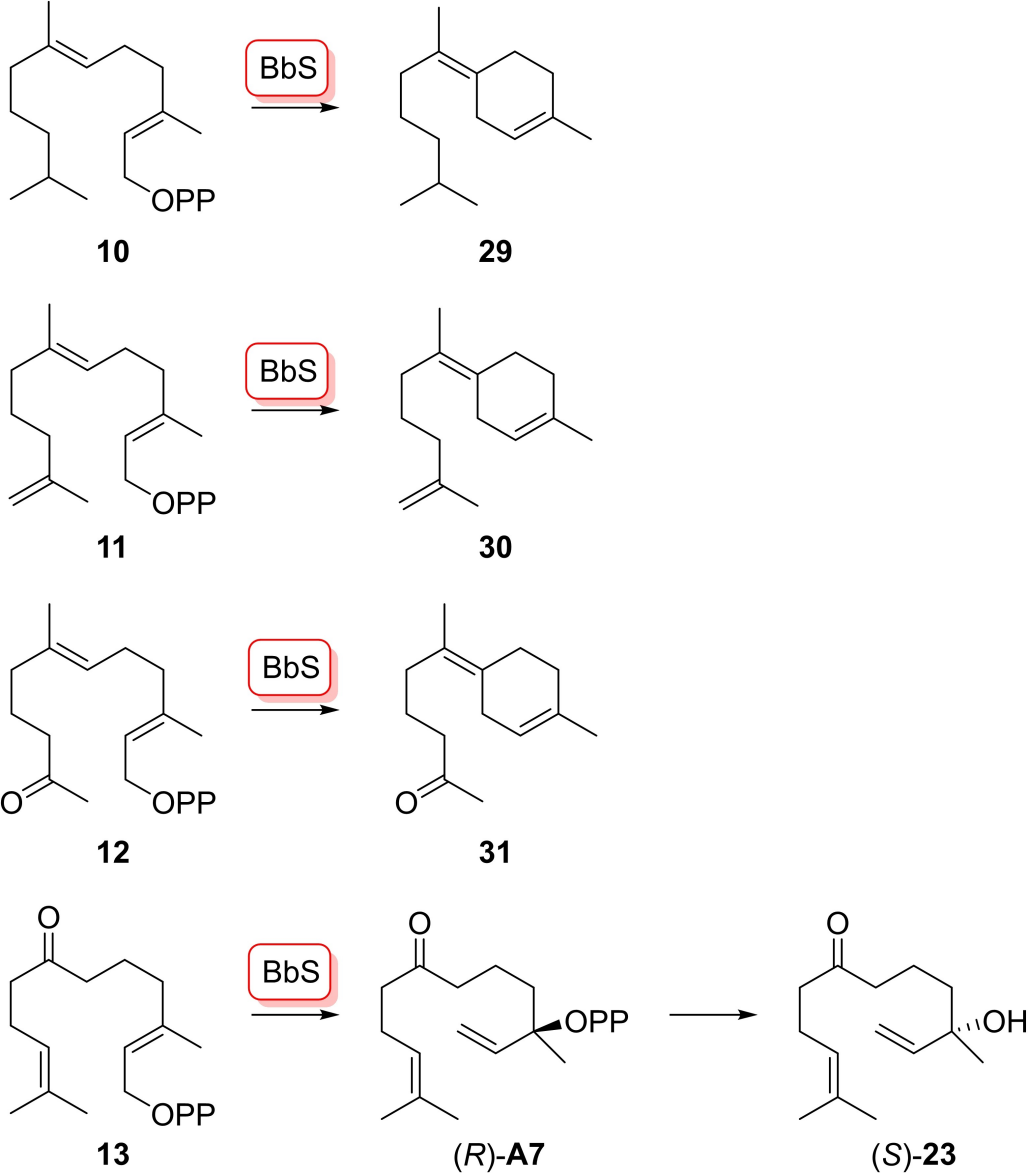
Enzymatic conversions FPP analogues **10**–**13** with BbS.

The FPP analogues **10**–**13** were finally tested with SmTS6 (Figures S73 and S74). With substrate **10**, only two acyclic products (6*E*)‐10,11‐dihydro‐β‐farnesene (**32**) and (6*E*)‐10,11‐dihydro‐nerolidol (**33**) were obtained (Scheme 8A, Tables S9 and S10, Figures S75–S90), demonstrating that SmTS6 cannot switch to a 1,6‐cyclisation mode. The absolute configuration of **33** was determined by chemical correlation to (*R*)‐ and (*S*)‐**25**. Catalytic hydrogenation of **33** and comparison to the **25** hydrogenation products **26 a** and **26 b** by GC using a chiral stationary phase confirmed the structure of (*R*)‐**33** (92 % *ee*, Figure S91). Taking analogue **11** as substrate, two products (1*E*,5*E*,9*E*)‐1,5,9‐trimethylcyclododeca‐1,5,9‐triene (**34**, Table S11, Figures S92–S99) and (4*E*,8*E*)‐1,5,9‐trimethyl‐cyclododeca‐4,8‐dien‐1‐ol (**35**, Table S12, Figures S100–S107) were isolated. Their formation can be rationalised through a 1,12‐cyclisation of **11** to cation **A5**. Its deprotonation leads to **34**, while attack by water gives raise to alcohol **35**. Compound **34** is *C*_3h_ symmetric and shows only five signals in the ^13^C NMR. Its structure was confirmed by catalytic hydrogenation that resulted in the same products as obtained with **19** (Scheme 4, Figure S108).

**Scheme 8:**
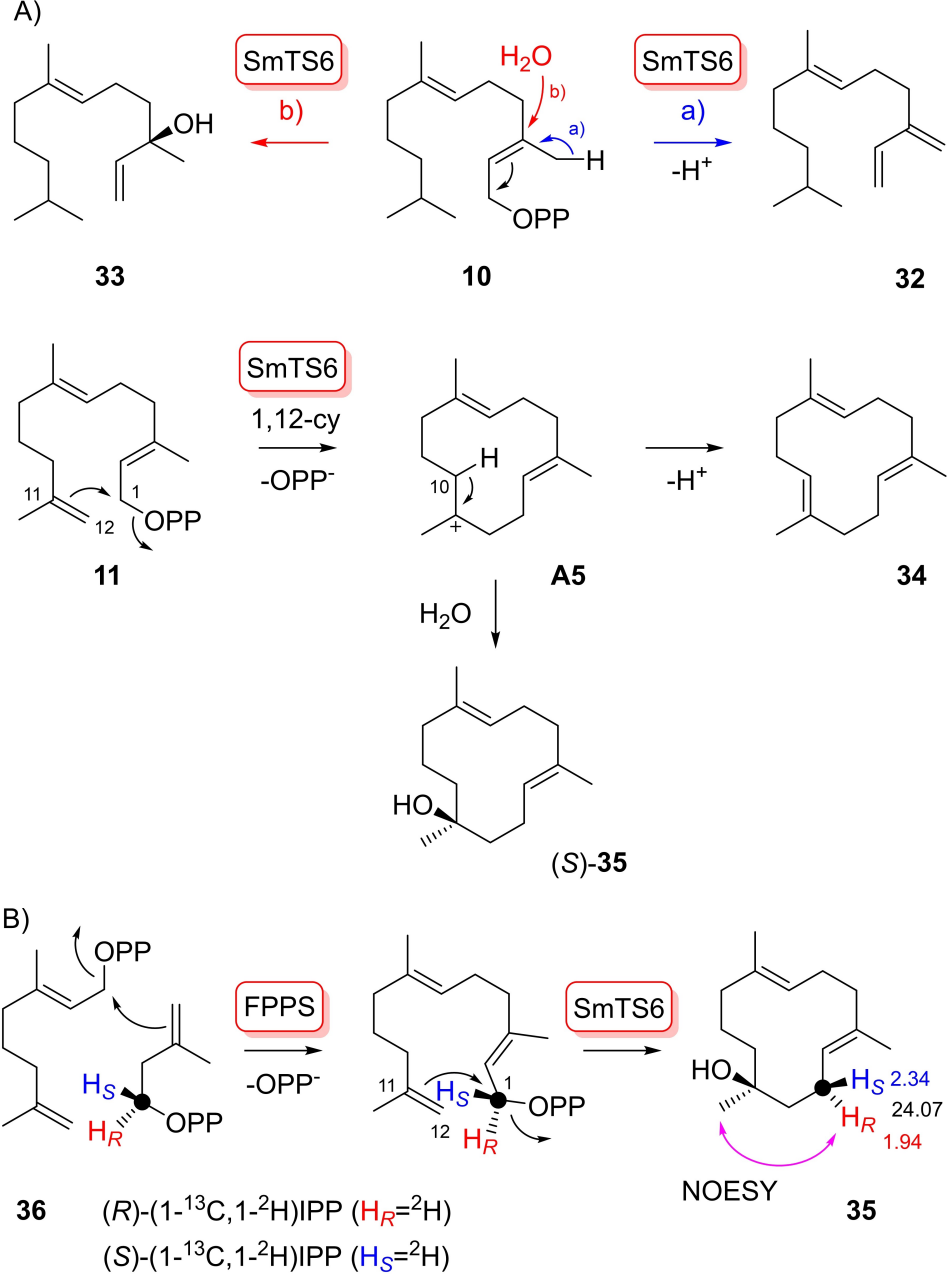
A) Enzymatic conversions FPP analogues **10** and **12** with SmTS6. B) Determination of the absolute configuration of **35** by labelling experiments with **36** and (*R*)‐ and (*S*)‐(1‐^13^C,1‐^2^H)IPP.

The absolute configuration of **35** was determined by labelling experiments with (*R*)‐ and (*S*)‐(1‐^13^C,1‐^2^H)IPP^[31]^ and the GPP analogue **36** ^[19]^ that were converted into stereoselectively deuterated (*R*)‐ and (*S*)‐(1‐^13^C,1‐^2^H)‐**11** with FPP synthase (FPPS) from *Streptomyces coelicolor* ^[32]^ (Scheme 8B). The incorporation of labelling into **35** can be followed by HSQC spectroscopy into one of the diastereotopic positions assigned by NOESY spectroscopy with the unlabelled compound. Assuming inversion of configuration in the cyclisation to **35**, the absolute configuration of (*S*)‐**35** was concluded (Figure S109). With substrate **12**, SmTS6 showed only poor conversion that was insufficient for product isolation, again in line with the inability of SmTS6 to perform 1,6‐cyclisations. For analogue **13**, (*S*)‐**23** was obtained, as confirmed by GC analysis on a chiral stationary phase (Figure S110).

## Conclusion

Four synthetic FPP analogues were converted with the bacterial β‐himachalene synthase (HcS), (*Z*)‐γ‐bisabolene synthase (BbS), and germacrene A synthase (SmTS6), yielding interesting products in most cases. Most of these compounds are unknown or only poorly described. One exception is **34** that has been obtained before from isoprene in a metathesis reaction using a tungsten‐carbene complex,^[33]^ while the isomer **19** was only obtained as a mixture with **34** and other cyclotrimers of isoprene.^[34]^ Their enzymatic formation reported here opens the possibility to obtain both stereoisomers selectively with different STPSs. It is also interesting to note that different enzymes can produce enantiomers, as observed for the new compound **22** for which the enzymatic formation of its enantiomer was previously reported from the same FPP analogue.^[19]^ For compound **33** a synthesis from 6‐methylheptan‐2‐one has been reported.^[35]^ (*E*)‐*iso*‐γ‐Bisabolene, a stereoisomer of **30**, has been tentatively identified by GC/MS in headspace extracts from the fungus *Fusarium* where it could be a side product of trichodiene synthase,[^36^, ^37^] and the *E*/*Z* mixture of **30** has been obtained by synthesis,^[38]^ but the pure *Z* isomer has not been made available before. Also the enzyme products (*R*)‐ and (*S*)‐**23**, **24**, **29**, **31**, **32** and **35** and their synthetic derivatives **27** and **28** were isolated in this study for the first time and obtained in high stereoisomeric purity, showing that the enzymatic conversion of FPP analogues can open the doors towards a new chemical space.

BbS catalyses an initial 1,6‐cyclisation of FPP and yielded corresponding products from all substrate analogues, only with substrate **13** for which a 1,6‐cyclisation is blocked the acyclic product (*S*)‐**23** was obtained. The 1,10‐cyclising SmTS6 gave acyclic products with **10** for which such a cyclisation is not possible, however, substrate **13** that could in principle undergo 1,10‐cyclisation also only gave an acyclic product, likely because the conformational fold of this substrate in the active site is disturbed. With FPP analogue **11** a 1,12‐cyclisation was observed, demonstrating that new cyclisation paths can be opened that follow the intrinsic reactivity of the substrate. The results with HcS are less clear. With **10** no product was obtained, while with **11** a 1,12‐cyclisation and with **13** a 1,10‐cyclisation was manifested. However, these observations were contrasted by the conversion of **12** through initial 1,6‐cyclisation. We have discussed in our previous work that the HcS mechanism can be understood either by initial 1,6‐ or 1,11‐cyclisation.^[27]^ The results obtained here seem to indicate that HcS can switch between these modes, which may also be true for the natural FPP cyclisation. Future research may further clarify this point.

## Conflict of interest

The authors declare no conflict of interest.

## Supporting information

As a service to our authors and readers, this journal provides supporting information supplied by the authors. Such materials are peer reviewed and may be re‐organized for online delivery, but are not copy‐edited or typeset. Technical support issues arising from supporting information (other than missing files) should be addressed to the authors.

Supporting Information

## References

[1] J. Gershenzon , N. Dudareva , Nat. Chem. Biol. 2007, 3, 408–414. 17576428 10.1038/nchembio.2007.5

[2] J. S. Dickschat , Nat. Prod. Rep. 2016, 33, 87–110. 26563452 10.1039/c5np00102a

[3] D. W. Christianson , Chem. Rev. 2017, 117, 11570–11648. 28841019 10.1021/acs.chemrev.7b00287 PMC5599884

[4] J. Bohlmann , C. L. Steele , R. Croteau , J. Biol. Chem. 1997, 272, 21784–21792. 9268308 10.1074/jbc.272.35.21784

[5] D. E. Cane , I. Kang , Arch. Biochem. Biophys. 2000, 376, 354–364. 10775423 10.1006/abbi.2000.1734

[6] J. S. Dickschat , Angew. Chem. Int. Ed. 2019, 58, 15964–15976; 10.1002/anie.201905312 31183935

[7] A. Minami , T. Ozaki , C. Liu , H. Oikawa , Nat. Prod. Rep. 2018, 35, 1330–1346. 29855001 10.1039/c8np00026c

[8] C.-M. Wang , D. E. Cane , J. Am. Chem. Soc. 2008, 130, 8908–8909. 18563898 10.1021/ja803639g PMC3023297

[9] M. Komatsu , M. Tsuda , S. Ōmura , H. Oikawa , H. Ikeda , Proc. Natl. Acad. Sci. USA 2008, 105, 7422–7427. 18492804 10.1073/pnas.0802312105 PMC2387273

[10] J. S. Dickschat , T. Nawrath , V. Thiel , B. Kunze , R. Müller , S. Schulz , Angew. Chem. Int. Ed. 2007, 46, 8287–8290; 10.1002/anie.200702496 17899580

[11] S. von Reuss , D. Domik , M. C. Lemfack , N. Magnus , M. Kai , T. Weise , B. Piechulla , J. Am. Chem. Soc. 2018, 140, 11855–11862. 30133268 10.1021/jacs.8b08510

[12] T. Ozaki , S. S. Shinde , L. Gao , R. Okuizumi , C. Liu , Y. Ogasawara , X. Lei , T. Dairi , A. Minami , H. Oikawa , Angew. Chem. Int. Ed. 2018, 57, 6629–6632; 10.1002/anie.201802116 29603559

[13] C. Sallaud , D. Rontein , S. Onillon , F. o. Jabès , P. Duffé , C. c. Giacalone , S. Thoraval , C. Escoffier , G. t. Herbette , N. Leonhardt , M. Causse , A. Tissier , Plant Cell 2009, 21, 301–317. 19155349 10.1105/tpc.107.057885 PMC2648096

[14] V. Harms , A. Kirschning , J. S. Dickschat , Nat. Prod. Rep. 2020, 37, 1080–1097. 32068211 10.1039/c9np00055k

[15] Y. Jin , D. C. Williams , R. Croteau , R. M. Coates , J. Am. Chem. Soc. 2005, 127, 7834–7842. 15913373 10.1021/ja050592r

[16] O. Cascón , S. Touchet , D. J. Miller , V. Gonzalez , J. A. Faraldos , R. K. Allemann , Chem. Commun. 2012, 48, 9702–9704. 10.1039/c2cc35542f 22914774

[17] A. Hou , L. Lauterbach , J. S. Dickschat , Chem. Eur. J. 2020, 26, 2178–2182. 31898827 10.1002/chem.201905827 PMC7065205

[18] V. Harms , B. Schröder , C. Oberhauser , C. D. Tran , S. Winkler , G. Dräger , A. Kirschning , Org. Lett. 2020, 22, 4360–4365. 32432889 10.1021/acs.orglett.0c01345

[19] L. Lauterbach , A. Hou , J. S. Dickschat , Chem. Eur. J. 2021, 27, 7923–7929. 33769623 10.1002/chem.202100962 PMC8252471

[20] M. Demiray , X. Tang , T. Wirth , J. A. Faraldos , R. K. Allemann , Angew. Chem. Int. Ed. 2017, 56, 4347–4350; 10.1002/anie.201609557 PMC5396139 28294491

[21] F. Huynh , D. J. Grundy , R. L. Jenkins , D. J. Miller , R. K. Allemann , ChemBioChem 2018, 19, 1834–1838. 29802753 10.1002/cbic.201800218 PMC6334173

[22] C. Oberhauser , V. Harms , K. Seidel , B. Schröder , K. Ekramzadeh , S. Beutel , S. Winkler , L. Lauterbach , J. S. Dickschat , A. Kirschning , Angew. Chem. Int. Ed. 2018, 57, 11802–11806; 10.1002/anie.201805526 29953712

[23] S. Y. Chow , H. J. Williams , Q. Huang , S. Nanda , A. I. Scott , J. Org. Chem. 2005, 70, 9997–10003. 16292833 10.1021/jo0517489

[24] G. Li , Y.-W. Guo , J. S. Dickschat , Angew. Chem. Int. Ed. 2021, 60, 1488–1492; 10.1002/anie.202014180 PMC7839432 33169911

[25] J. A. Faraldos , P. E. O'Maille , N. Dellas , J. P. Noel , R. M. Coates , J. Am. Chem. Soc. 2010, 132, 4281–4289. 20201526 10.1021/ja909886q

[26] P. Rabe , J. Rinkel , T. A. Klapschinski , L. Barra , J. S. Dickschat , Org. Biomol. Chem. 2016, 14, 158–164. 26469060 10.1039/c5ob01998b

[27] J. Rinkel , J. S. Dickschat , Beilstein J. Org. Chem. 2019, 15, 1008–1019. 31164939 10.3762/bjoc.15.99 PMC6541374

[28] J. Rinkel , J. S. Dickschat , Beilstein J. Org. Chem. 2019, 15, 789–794. 30992727 10.3762/bjoc.15.75 PMC6444425

[29] A. Hou , J. S. Dickschat , Angew. Chem. Int. Ed. 2020, 59, 19961–19965; 10.1002/anie.202010084 PMC7693059 32749032

[30] J. Rinkel , L. Lauterbach , J. S. Dickschat , Angew. Chem. Int. Ed. 2017, 56, 16385–16389; 10.1002/anie.201711142 29125678

[31] J. Rinkel , J. S. Dickschat , Org. Lett. 2019, 21, 2426–2429. 30859837 10.1021/acs.orglett.9b00725

[32] P. Rabe , J. Rinkel , B. Nubbemeyer , T. G. Köllner , F. Chen , J. S. Dickschat , Angew. Chem. Int. Ed. 2016, 55, 15420–15423; 10.1002/anie.201608971 27862766

[33] E. Thorn-Csanyi , J. Hammer , J. U. Zilles , Macromol. Rapid Commun. 1994, 15, 797–800.

[34] H. Morikawa , S. Kitazume , Ind. Eng. Chem. Prod. Res. Dev. 1979, 18, 254–258.

[35] A. Ofner , W. Kimel , A. Holmgren , F. Forrester , Helv. Chim. Acta 1959, 42, 2577–2584.

[36] J. S. Dickschat , N. L. Brock , C. A. Citron , B. Tudzynski , ChemBioChem 2011, 12, 2088–2095. 21748838 10.1002/cbic.201100268

[37] Y. J. Hong , D. J. Tantillo , J. Am. Chem. Soc. 2014, 136, 2450–2463. 24490652 10.1021/ja4106489

[38] N. A. Braun , M. Meier , G. Schmaus , B. Hölscher , W. Pickenhagen , Helv. Chim. Acta 2003, 86, 2698–2708.

