# Supplementary material for: Using Terpene Synthase Plasticity in Catalysis: On the Enzymatic Conversion of Synthetic Farnesyl Diphosphate Analogues
Source: Chemistry. 2021 Sep 29;27(63):15644–9. doi: 10.1002/chem.202103049 (PMC9292696; doi:10.1002/chem.202103049)
Supplement: Supplementary file 1 — Supporting Information [file CHEM-27-15644-s001.pdf]

Chemistry–A European Journal

Supporting Information

Using Terpene Synthase Plasticity in Catalysis: On the Enzymatic Conversion of Synthetic Farnesyl Diphosphate Analogues

Anwei Hou and Jeroen S. Dickschat*

Synthesis of trisammonium (*E*)-3,11-dimethyl-7-oxododeca-2,10-dien-1-yl diphosphate (13)

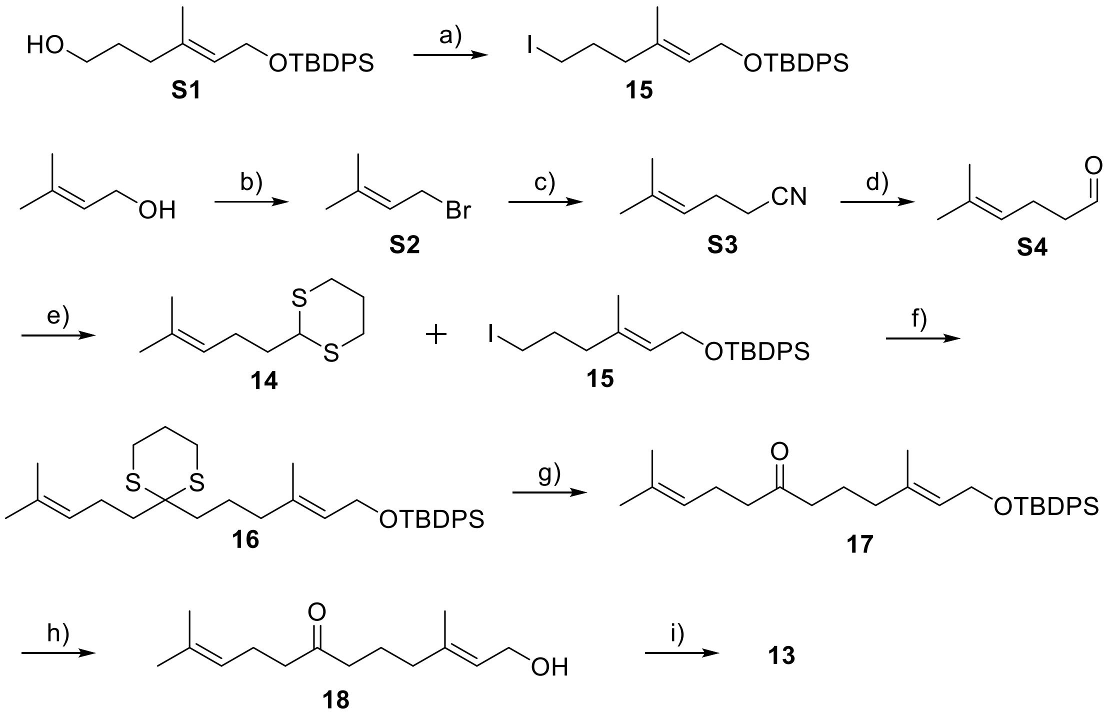

Scheme S1. Synthesis of compound **13**. Synthetic conditions: a) MsCl, Et_3N, CH_2Cl_2, 0 °C, 2 h; NaI, acetone, reflux, 8 h, 86%; b) PBr_3, Et_2O, 0 °C, 1 h; c) ACN, *n*-BuLi, THF, –78 °C to room temperature, overnight, 37% (two steps); d) DIBAL-H, CH_2Cl_2, –78 °C, 1 h, 66%; e) 1,3-propanedithiol, BF_3•Et_2O, CH_2Cl_2, 0 °C, 2 h, 46%; f) *n*-BuLi, 0 °C to room temperature, overnight, 69%; g) PIFA, CH_3OH/H_2O, room temperature, 30 min, 39%; h) TBAF, THF, room temperature, 1.5 h, 93%; i) PBr_3, THF, 0 °C, 45 min; $(NBu_4)_3HP_2O_7$, ACN, room temperature, overnight, 76%.

Synthesis of (*E*)-tert-butyl((6-iodo-3-methylhex-2-en-1-yl)oxy)diphenylsilane (15)
Alcohol **S1** was obtained by a published procedure.[1] To a CH_2Cl_2 (115 mL, 0 °C) solution of **S1** (8.44 g, 22.90 mmol) and Et_3N (4.63 g, 45.8 mmol, 2.0 eq) MsCl (3.15 g, 27.48 mmol, 1.2 eq) was added dropwise. The mixture was stirred at 0 °C for 2 h and then quenched by pouring onto an ice-cold aqueous $NaHCO_3$ solution (100 mL sat. $NaHCO_3$ with 100 mL ice-water). The product was extracted with Et_2O (3 x 100 mL). The combined organic layers were washed with HCl (100 mL; 1 M in H_2O) and brine. The solvent was removed under reduced pressure to afford the crude product which was used for the next step directly.

The crude product and NaI (4.46 g, 29.8 mmol, 1.3 eq) were dissolved in acetone (200 mL). The mixture was heated to reflux and kept at that temperature for 8 h. The solvent was removed under reduced pressure. Then aqueous sat. $NaHCO_3/Na_2S_2O_3$ (1/1, 100 mL) was added to the residue, and the product was extracted with Et_2O (3 x 60 mL). The combined organic layers were dried with $MgSO_4$ and concentrated under reduced pressure. The product **15** (9.37 g, 19.6 mmol, 86%) was purified via silica gel chromatography (cyclohexane/ethyl acetate, 20:1, R_f = 0.50) and was obtained as

colourless oil. EI-MS (70 eV): *m*/*z* (%) = 421 (30), 309 (14), 281 (3), 249 (4), 207 (8), 199 (100), 181 (10), 155 (8), 135 (6), 121 (4), 105 (6), 95 (15), 77 (10), 67 (7), 57 (13), 41 (11), 32 (4). GC (HP5-MS): *I* = 2802. ^{1}H NMR (500 MHz, C_6D_6): δ = 7.84 – 7.79 (m, 4H), 7.28 – 7.21 (m, 6H), 5.49 (tq, $^{3}J_{H,H}$ = 6.5 Hz, $^{4}J_{H,H}$ = 1.3 Hz, 1H), 4.27 (dq, $^{3}J_{H,H}$ = 6.4 Hz, $^{5}J_{H,H}$ = 0.9 Hz, 2H), 2.66 (t, $^{3}J_{H,H}$ = 7.0 Hz, 2H), 1.76 – 1.71 (m, 2H), 1.54 – 1.46 (m, 2H), 1.19 (s, 9H), 1.14 (q, $^{4}J_{H,H}$ = 1.2 Hz, 3H) ppm. ^{13}C NMR (126 MHz, C_6D_6): δ = 136.06 (4 x CH), 135.19 (C_q), 134.45 (2 x C_q), 129.99 (2 x CH), 128.08 (4 x CH), 125.80 (CH), 61.36 (CH_2), 39.96 (CH_2), 31.58 (CH_2), 27.11 (3 x CH_3), 19.48 (C_q), 15.95 (CH_3), 6.01 (CH_2) ppm.

Synthesis of 5-methylhex-4-enenitrile (S3)

To a Et_2O (80 mL, 0 °C) solution of 3-methylbut-2-en-1-ol (8.60 g, 100 mmol) was added PBr_3 (10.8 g, 40 mmol, 0.4 eq) dropwise. The mixture was stirred at 0 °C for 1 h and was quenched by pouring onto ice-water (400 mL). The product was extracted with Et_2O (3 x 70 mL). The combined extracts were washed with brine, dried with $MgSO_4$ and concentrated to afford crude **S2** which was used for the next step directly. Acetonitrile (2.46 g, 60 mmol, 0.6 eq) was dissolved in THF (200 mL). The mixture was cooled to –78 °C, followed by the dropwise addition of *n*-BuLi (37.5 mL, 1.6 M in hexane, 60 mmol, 0.6 eq). After stirring at –78 °C for 30 min, crude **S2** was added dropwise to the mixture. The reaction mixture was stirred overnight without further cooling, and then quenched by pouring onto an ice-cold aqueous NH_4Cl solution (100 mL sat. NH_4Cl with 100 mL ice-water). The product was extracted with Et_2O (3 x 80 mL). The combined organic layers were washed with sat. NaCl, dried with $MgSO_4$ and concentrated under reduced pressure. The product **S3** (4.00 g, 36.6 mmol, 37%) was purified by flash chromatography (pentane/Et_2O, 15:1, R_f = 0.31) and obtained as colourless oil. EI-MS (70 eV): *m*/*z* (%) = 109 (27), 94 (3), 69 (100), 53 (10), 41 (58). GC (HP5-MS): *I* = 976. ^{1}H NMR (500 MHz, C_6D_6): δ = 4.81 (thept, $^{3}J_{H,H}$ = 7.2 Hz, $^{4}J_{H,H}$ = 1.4 Hz, 1H), 1.75 – 1.67 (m, 2H), 1.50 (d, $^{4}J_{H,H}$ = 1.3 Hz, 3H), 1.43 (t, $^{3}J_{H,H}$ = 7.2 Hz, 2H), 1.32 (d, $^{4}J_{H,H}$ = 1.2 Hz, 3H) ppm. ^{13}C NMR (126 MHz, C_6D_6): δ = 134.81 (C_q), 120.94 (CH), 119.23 (C_q), 25.59 (CH_3), 24.21 (CH_2), 17.58 (CH_3), 17.17 (CH_2).

Synthesis of 5-methylhex-4-enal (S4)

To a CH_2Cl_2 (180 mL, –78 °C) solution of **S3** (4.00 g, 36.6 mmol) was added DIBAL-H (40 mL, 1 M in hexane, 40 mmol, 1.1 eq) dropwise. The reaction mixture was stirred at –78 °C for 1 h, followed by the addition of EtOH (4 mL). After stirring for 20 min, aqueous sat. NH_4Cl (54 mL) and Na-K-tartrate solution (140 mL) were added, and the mixture was stirred at room temperature for 30 min. The product was extracted with Et_2O (3 x 100 mL). The combined extracts were washed with brine, dried with $MgSO_4$ and concentrated under reduced pressure. Purification by flash chromatography (pentane/Et_2O, 15:1, R_f = 0.28) gave **S4** (2.73 g, 24.33 mmol, 66%) as a colourless oil. EI-MS (70 eV): *m*/*z* (%) = 112 (8), 94 (26), 79 (16), 69 (31), 56 (72), 41 (100). GC (HP5-MS): *I* = 896. ^{1}H NMR (500 MHz, C_6D_6): δ = 9.31 (t, $^{3}J_{H,H}$ = 1.6 Hz, 1H), 4.95 (thept, $^{3}J_{H,H}$ = 7.2 Hz, $^{4}J_{H,H}$ = 1.5 Hz, 1H), 2.10 – 2.02 (m, 2H), 1.93 – 1.83 (m, 2H), 1.56 (d, $^{4}J_{H,H}$ = 1.4 Hz, 3H), 1.42 (d, $^{4}J_{H,H}$ = 1.2 Hz, 3H) ppm. ^{13}C NMR (126 MHz, C_6D_6): δ =

200.44 (CH), 132.54 (C_q), 123.07 (CH), 43.89 (CH_2), 25.70 (CH_3), 21.15 (CH_2), 17.59 (CH_3) ppm.

Synthesis of 2-(4-methylpent-3-en-1-yl)-1,3-dithiane (14)

To a CH_2Cl_2 (56 mL, 0 °C) solution of **S4** (1.54 g, 13.73 mmol) and 1,3-propanedithiol (1.63 g, 15.1 mmol, 1.1 eq) was added BF_3•Et_2O (0.58 g, 4.12 mmol, 0.3 eq) dropwise. The mixture was stirred at 0 °C for 2 h and quenched by pouring onto an aqueous $NaHCO_3$ solution (70 mL sat. $NaHCO_3$ with 100 mL ice-water), followed by extraction with Et_2O (3 x 70 mL). The combined extracts were washed with brine, dried with $MgSO_4$ and concentrated under reduced pressure. Purification via silica gel chromatography (cyclohexane/ethyl acetate, 40:1, R_f = 0.31) provided **14** (1.78 g, 8.80 mmol, 46%) as a colourless oil. EI-MS (70 eV): *m*/*z* (%) = 202 (48), 169 (6), 159 (3), 145 (40), 133 (23), 119 (57), 106 (20), 95 (64), 85 (20), 69 (39), 59 (20), 41(100). GC (HP5-MS): *I* = 1648. ^{1}H NMR (500 MHz, C_6D_6): δ = 5.09 (thept, $^3J_{H,H}$ = 7.2 Hz, $^4J_{H,H}$ = 1.4 Hz, 1H), 3.93 (t, $^3J_{H,H}$ = 7.0 Hz, 1H), 2.40 – 2.33 (m, 4H), 2.32 – 2.19 (m, 2H), 1.92 – 1.83 (m, 2H), 1.61 (d, $^4J_{H,H}$ = 1.3 Hz, 3H), 1.62 – 1.55 (m, 1H), 1.55 (d, $^4J_{H,H}$ = 1.3 Hz, 3H), 1.49 – 1.39 (m, 1H) ppm. ^{13}C NMR (126 MHz, C_6D_6): δ = 132.68 (C_q), 123.72 (CH), 47.23 (CH), 36.07 (CH_2), 30.29 (2 x CH_2), 26.27 (CH_2), 25.86 (CH_3), 25.57 (CH_2), 17.79 (CH_3) ppm.

Synthesis of (*E*)-tert-butyl((3-methyl-6-(2-(4-methylpent-3-en-1-yl)-1,3-dithian-2-yl)hex-2-en-1-yl)oxy)diphenylsilane (16)

To a THF (3 mL, 0 °C) solution of **14** (202 mg, 1.00 mmol) was added *n*-BuLi (0.63 mL, 1.6 M in hexane, 1.01 mmol, 1.0 eq) dropwise. The mixture was stirred at 0 °C for 1 h, followed by the dropwise addition of **15** (478 mg, 1.00 mmol, 1.0 eq). The reaction mixture was stirred overnight without further cooling, then quenched by the addition of aqueous sat. NH_4Cl. The product was extracted with Et_2O (3 x 30 mL). The combined extracts were dried with $MgSO_4$ and concentrated under reduce pressure. The product **16** (0.38 g, 0.69 mmol, 69%) was purified via silica gel chromatography (cyclohexane/ethyl acetate, 40:1, R_f = 0.29) and was obtained as a colourless oil. ^{1}H NMR (500 MHz, C_6D_6): δ = 7.85 – 7.78 (m, 4H), 7.29 – 7.21 (m, 6H), 5.59 (t, $^3J_{H,H}$ = 6.4 Hz, 1H), 5.19 (t, $^3J_{H,H}$ = 7.1 Hz, 1H), 4.30 (d, $^3J_{H,H}$ = 6.3 Hz, 2H), 2.48 – 2.39 (m, 4H), 2.36 – 2.27 (m, 2H), 2.04 – 1.96 (m, 2H), 1.95 – 1.86 (m, 4H), 1.73 – 1.68 (m, 2H), 1.66 (d, $^4J_{H,H}$ = 1.3 Hz, 3H), 1.59 (d, $^4J_{H,H}$ = 1.2 Hz, 3H), 1.57 – 1.52 (m, 2H), 1.34 (d, $^4J_{H,H}$ = 1.2 Hz, 3H), 1.17 (s, 9H) ppm. ^{13}C NMR (126 MHz, C_6D_6): δ = 136.82 (C_q), 136.04 (4 x CH), 134.48 (2 x C_q), 131.87 (C_q), 129.90 (2 x CH), 128.06 (4 x CH), 125.18 (CH), 124.35 (CH), 61.42 (CH_2), 53.56 (C_q), 39.73 (CH_2), 38.92 (CH_2), 38.31 (CH_2), 27.14 (3 x CH_3), 26.11 (2 x CH_2), 25.88 (CH_3), 25.78 (CH_2), 23.78 (CH_2), 22.42 (CH_2), 19.48 (C_q), 17.75 (CH_3), 16.08 (CH_3) ppm.

Synthesis of (*E*)-12-((tert-butyldiphenylsilyl)oxy)-2,10-dimethyldodeca-2,10-dien-6-one (17)

To compound **16** (3.22 g, 5.82 mmol) were added CH_3OH (9 mL) and water (1 mL) to obtain an emulsion. With stirring at room temperature, PIFA (3.76 g, 8.73 mmol, 1.5

eq) was added. The reaction mixture was stirred at room temperature for 30 min, and then quenched by the addition of sat. $NaHCO_3$ (70 mL). The product was extracted with Et_2O (3 x 50 mL). The organic layers were combined, dried with $MgSO_4$ and concentrated under reduced pressure. Purification via silica gel chromatography (cyclohexane/ethyl acetate, 30:1, R_f = 0.22) provided **17** (1.06 g, 2.29 mmol, 39%) as a colorless oil. EI-MS (70 eV): *m*/*z* (%) = 405 (1), 323 (7), 245 (2), 199 (100), 189 (11), 175 (2), 147 (3), 135 (7), 121 (9), 105 (6), 95 (6), 81 (8), 69 (20), 55 (10), 41 (15). GC (HP5-MS): *I* = 3158. ^{1}H NMR (500 MHz, C_6D_6): δ = 7.87 – 7.80 (m, 4H), 7.30 – 7.20 (m, 6H), 5.55 (tq, $^3J_{H,H}$ = 6.3 Hz, $^4J_{H,H}$ = 1.3 Hz, 1H), 5.12 (thept, $^3J_{H,H}$ = 7.2 Hz, $^4J_{H,H}$ = 1.5 Hz, 1H), 4.32 (dq, $^3J_{H,H}$ = 6.2 Hz, $^5J_{H,H}$ = 0.8 Hz, 2H), 2.34 – 2.24 (m, 2H), 2.09 (t, $^3J_{H,H}$ = 7.3 Hz, 2H), 1.96 (t, $^3J_{H,H}$ = 7.3 Hz, 2H), 1.82 (t, $^3J_{H,H}$ = 7.4 Hz, 2H), 1.63 (d, $^4J_{H,H}$ = 1.4 Hz, 3H), 1.65 – 1.57 (m, 2H), 1.54 (d, $^4J_{H,H}$ = 1.2 Hz, 3H), 1.29 (d, $^4J_{H,H}$ = 1.2 Hz, 3H), 1.19 (s, 9H) ppm. ^{13}C NMR (126 MHz, C_6D6): δ = 208.19 (C_q), 136.80 (C_q), 136.07 (4 x CH), 134.49 (2 x C_q), 132.14 (C_q), 129.96 (2 x CH), 128.09 (4 x CH), 125.25 (CH), 123.89 (CH), 61.47 (CH_2), 42.77 (CH_2), 41.87 (CH_2), 39.10 (CH_2), 27.13 (3 x CH_3), 25.84 (CH_3), 22.93 (CH_2), 21.84 (CH_2), 19.50 (C_q), 17.69 (CH_3), 15.98 (CH_3) ppm.

Synthesis of (*E*)-12-hydroxy-2,10-dimethyldodeca-2,10-dien-6-one (18)

Ketone **17** (0.51 g, 1.10 mmol) was dissolved in THF (6 mL). The mixture was cooled to 0 °C, followed by the dropwise addition of TBAF (1.32 mL, 1 M in THF, 1.32 mmol, 1.2 eq). After stirring at room temperature for 1.5 h, the reaction was quenched by pouring onto ice-water (50 mL), and the product was extracted with Et_2O (3 x 50 mL). The combined extracts were washed with brine, dried with $MgSO_4$ and concentrated under reduced pressure. Purification via silica gel chromatography (pentane/Et_2O, 1:2, R_f = 0.30) provided **18** (0.23 g, 1.03 mmol, 93%) as a colorless oil. EI-MS (70 eV): *m*/*z* (%) = 206 (5), 191 (15), 177 (2), 173 (3), 163 (3), 145 (4), 137 (7), 124 (19), 109 (20), 95 (36), 82 (59), 69 (88), 55 (69), 41 (100). GC (HP5-MS): *I* = 1830. ^{1}H NMR (300 MHz, C_6D_6): δ = 5.34 (tq, $^3J_{H,H}$ = 6.7 Hz, $^4J_{H,H}$ = 1.3 Hz, 1H), 5.12 (thept, $^3J_{H,H}$ = 7.2 Hz, $^4J_{H,H}$ = 1.4 Hz, 1H), 3.96 (d, $^3J_{H,H}$ = 6.6 Hz, 2H), 2.36 – 2.21 (m, 2H), 2.14 – 2.02 (m, 2H), 1.96 (t, $^3J_{H,H}$ = 7.2 Hz, 2H), 1.88 – 1.76 (m, 2H), 1.63 (d, $^3J_{H,H}$ = 1.4 Hz, 3H), 1.67 – 1.58 (m, 2H), 1.54 (d, $^3J_{H,H}$ = 1.3 Hz, 3H), 1.43 (d, $^3J_{H,H}$ = 1.2 Hz, 3H), 0.83 (s, 1H) ppm. ^{13}C NMR (75 MHz, C_6D6): δ = 208.42 (C_q), 137.67 (C_q), 132.20 (C_q), 125.43 (CH), 123.84 (CH), 59.32 (CH_2), 42.76 (CH_2), 41.89 (CH_2), 39.11 (CH_2), 25.82 (CH_3), 22.92 (CH_2), 21.84 (CH_2), 17.68 (CH_3), 15.92 (CH_3) ppm.

Synthesis of trisammonium (*E*)-3,11-dimethyl-7-oxododeca-2,10-dien-1-yl diphosphate (13)

Alcohol **18** (230 mg, 1.03 mmol) was dissolved in Et_2O (10 mL). The solution was cooled to 0 °C, followed by the dropwise addition of PBr_3 (111 mg, 0.41 mmol, 0.4 eq). After stirring at 0 °C for 45 min, the reaction was quenched by pouring onto ice-water (100 mL). The product was extracted with Et_2O (3 x 50 mL). The combined extracts were washed with brine, dried with $MgSO_4$ and concentrated under reduced pressure. The crude bromide was used for next step directly.

$(NBu_4)_3HP_2O_7$ (1.80 g, 1.98 mmol, 1.9 eq) was dissolved in acetonitrile (1 mL), followed

by the dropwise addition of the bromide. The mixture was stirred at room temperature overnight. The solvent was removed under reduced pressure. The residue was loaded onto an ion-exchange column (DOWEX® 50WX8, $NH4^+$ form, pH ~ 7.0) and washed with two column volumes of elution buffer (2% *i*PrOH in 25 mM aqueous NH_4CO_3). The eluate was lyophilised to provide **13** (340 mg, 0.78 mmol, 76%) as a colourless powder. ^{1}H NMR (300 MHz, D_2O): δ = 5.37 (d, $^3J_{H,H}$ = 7.2 Hz, 1H), 5.04 (t, $^3J_{H,H}$ = 6.8 Hz, 1H), 4.39 (t, $^3J_{H,H}$ = 6.7 Hz, 2H), 2.54 – 2.40 (m, 4H), 2.24 – 2.08 (m, 2H), 2.03 – 1.89 (m, 2H), 1.62 (s, 3H), 1.61 – 1.60 (m, 5H), 1.54 (s, 3H) ppm. ^{13}C NMR (75 MHz, D_2O): δ = 218.69 (C_q), 141.83 (C_q), 134.20 (C_q), 122.45 (CH), 120.39 (CH), 62.40 (CH_2), 42.14 (CH_2), 41.80 (CH_2), 37.97 (CH_2), 24.76 (CH_3), 22.35 (CH_2), 21.13 (CH_2), 16.84 (CH_3), 15.30 (CH_3) ppm.

Incubation reactions with HcS and substrates 10 – 13

HcS was purified following a published procedure.[2] For small scale reactions, the purified protein solution (1 mL, ca. 3 mg/mL) was mixed with Tris buffer (3 mL; 50 mM Tris, 1 mM $MgCl_2$, pH = 7.6), incubation buffer (5 mL, 50 mM Tris, 10 mM $MgCl_2$, 20% glycerol, pH = 7.6) and each substate analogue **10** – **13** (1 mL; 1 mg/mL in 25 mM aqueous NH_4HCO_3). The mixture was incubated at 28 °C overnight, and extracted with hexane (200 µL), with separation of the layers by centrifugation (12000 rpm x 6 min), followed by GC/MS analysis of the products.

For large scale enzymatic reactions, the purified protein solution (50 mL; obtained from 4 L *E.coli* culture) was mixed with Tris buffer (150 mL), incubation buffer (250 mL; 50 mM Tris, 5 mM $MgCl_2$, 5% glycerol, pH = 7.6) and each substrate analogue **10** – **13** (50 mL; 1 mg/mL in 25 mM aqueous NH_4HCO_3). The mixture was incubated at 28 °C overnight. The products were extracted with pentane (2 x 300 mL). Each reaction was performed twice, and all the extracts were combined and concentrated under reduced pressure. The products were purified via silica gel chromatography.

(1*Z*,5*E*,9*E*)-1,5,9-Trimethylcyclododeca-1,5,9-triene (19). Yield: 0.5 mg, 2.4 µmol, 1%. TLC (pentane, 100%): R_f = 0.50. MS spectrum cf. Figure S2A. GC (HP5-MS): *I* = 1570. HRMS (APCI): *m*/*z* = 205.1958 (calc. for $[C_{15}H_{24} + H]^+$: 205.1951). IR (diamond ATR): $\tilde{\nu}$ = 2954 (m), 2910 (s), 2850 (m), 1737 (w), 1671 (w), 1446 (m), 1383 (m), 1227 (w), 1148 (w), 1084 (w), 1047 (w), 934 (w), 872 (w), 829 (m), 814 (w), 735 (w), 552 (w), 494 (w), 454 (w), 419 (w) cm^{-1}. NMR data cf. Table S1 and Figures S3 – S10.

(*R*)-6-Hydroxy-6-((*S*)-4-methylcyclohex-3-en-1-yl)heptan-2-one (22). Yield: 2.1 mg, 9.4 µmol, 4%. TLC (Et_2O, 100%): R_f = 0.49. Optical rotation: $[\alpha]_D^{25}$ = –16.2 (*c* 0.21, CH_2Cl_2). MS spectrum cf. Figure S2B. Other spectroscopic data were the same as published previously.[1]

(*R*)-10-Hydroxy-2,10-dimethyldodeca-2,11-dien-6-one (23). Yield: 10.0 mg, 44.6 µmol, 19%. TLC (Et_2O/pentane, 3:1): R_f = 0.68. Optical rotation: $[\alpha]_D^{25}$ = –6.5 (*c* 1.00, CH_2Cl_2). MS spectrum cf. Figure S2C. GC (HP5-MS): *I* = 1382. HRMS (ESI): *m*/*z* = 225.1850 (calc. for $[C_{14}H_{24}O_2 + H]^+$: 225.1849). IR (diamond ATR): $\tilde{\nu}$ = 3446 (br w), 2967 (m), 2917 (m), 2859 (m), 1707 (s), 1642 (w), 1451 (m), 1409 (m), 1375 (m), 1219 (w), 1163 (w), 1111 (w), 1084 (m), 995 (m), 918 (s), 833 (w), 692 (w), 538 (w), 427 (w) cm^{-1}. NMR data cf. Table S2 and Figures S13 – S20.

(4a*R*,7*R*,8a*R*)-1-Methylene-7-(prop-1-en-2-yl)octahydronaphthalen-4a(*2H*)-ol (24). Yield: 2.8 mg, 13.6 µmol, 6%. TLC (Et_2O/pentane, 3:1): R_f = 0.84. Optical rotation: $[\alpha]_D^{25}$ = –57.5 (*c* 0.28, CH_2Cl_2). MS spectrum cf. Figure S2D. GC (HP5-MS): *I* = 1587. HRMS (APCI): *m*/*z* = 207.1740 (calc. for $[C_{14}H_{22}O + H]^+$: 207.1743). IR (diamond ATR): $\tilde{\nu}$ = 3569 (w), 3469 (w), 3083 (w), 2932 (s), 2863 (m), 1708 (w), 1641 (m), 1441 (m), 1372 (w), 1253 (w), 1187 (w), 1166 (w), 1096 (w), 1037 (w), 983 (w), 956 (w), 938 (m), 889 (s), 864 (w), 803 (w), 756 (w), 622 (w), 582 (w), 545 (w), 482 (w) cm^{-1}. NMR data cf. Table S3 and Figures S21 – S28.

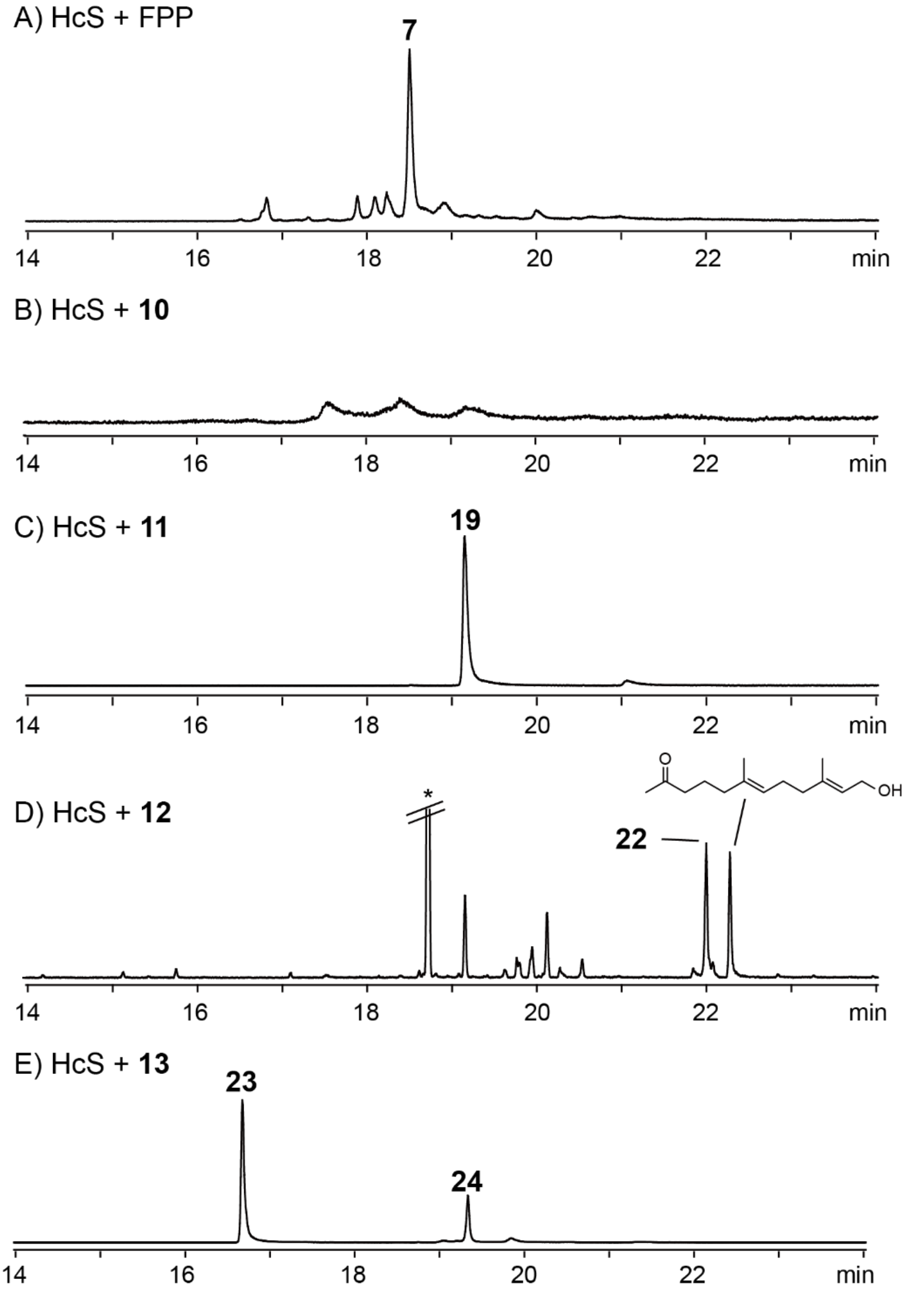

Figure S1. Total ion chromatograms of the extracts from the incubation reactions with HcS and A) FPP, B) substrates **10**, C) **11**, D) **12** and E) **13**. The asterisk indicates an impurity from the solvent.

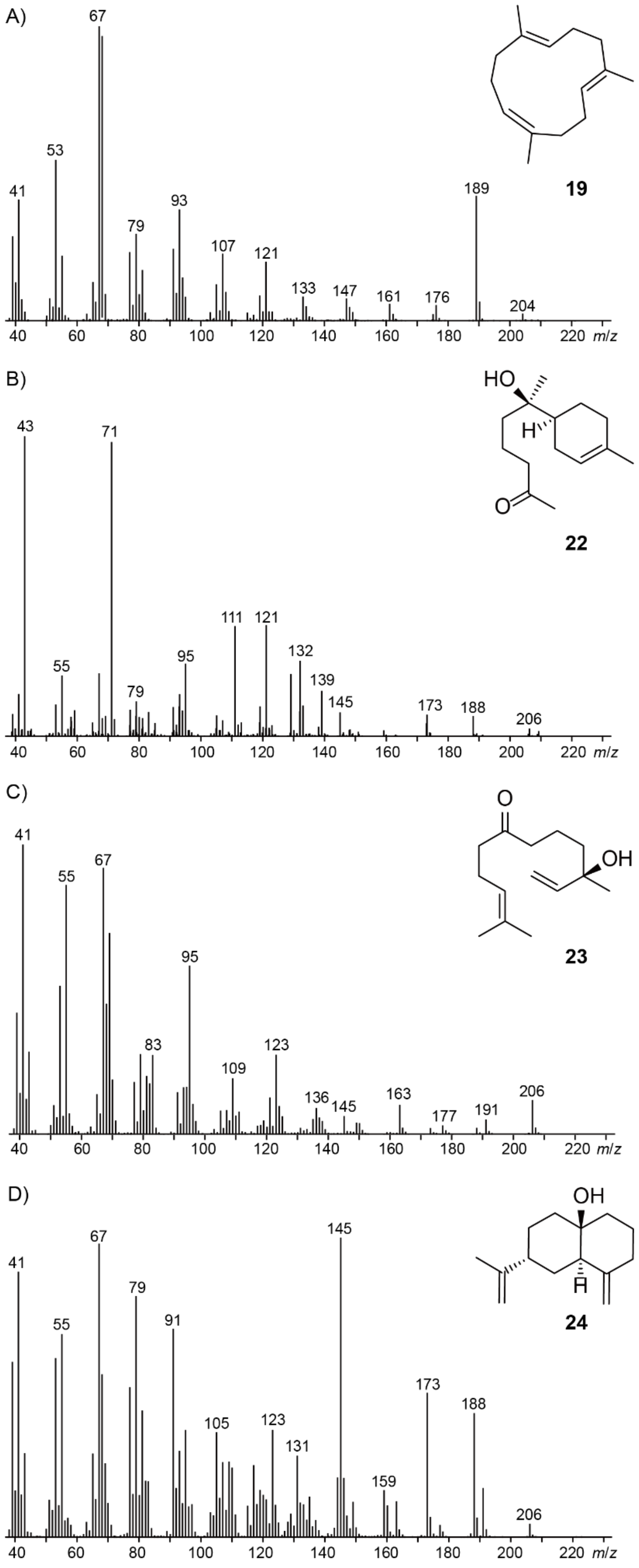

Figure S2. EI mass spectra of compounds A) **19**, B) **22**, C) **23** and D) **24**.

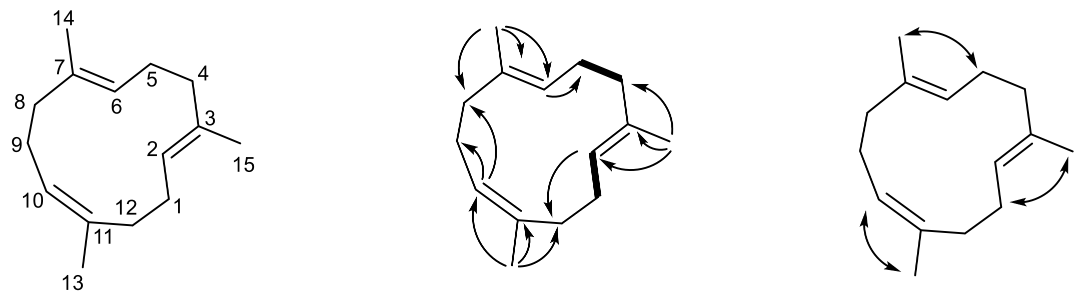

19

Figure S3. Structure elucidation of **19**. Bold: ^{1}H,^{1}H-COSY, single headed arrows: key HMBC, and double headed arrows: key NOESY correlations.

Table S1. NMR data of **19** in C_6D_6 recorded at 298 K.

C[a]	type	^{13}C[b]	^{1}H[b]
1	CH_2	24.82	2.13 (m, 2H)
2	CH	126.39	4.97 (t, *J* = 7.9 Hz, 1H)
3	C_q	132.01	–
4	CH_2	39.03	1.99 (m, 2H)
5	CH_2	25.40	2.08 (m, 2H)
6	CH	125.37	4.93 (t, *J* = 7.6 Hz, 1H)
7	C_q	135.28	–
8	CH_2	39.51	2.02 (m, 2H)
9	CH_2	28.12	2.10 (m, 1H)
			2.04 (m, 1H)
10	CH	127.52	5.13 (t, *J* = 7.8 Hz, 1H)
11	C_q	133.79	–
12	CH_2	31.38	missing
13	CH_3	22.92	1.68 (s, 3H)
14	CH_3	17.79	1.52 (d, *J* = 1.5 Hz, 3H)
15	CH_3	15.55	1.49 (d, *J* = 1.4 Hz, 3H)

[a] Carbon numbering as shown in Figure S3. [b] Chemical shifts δ in ppm, multiplicity: s = singlet, d = doublet, t = triplet, m = multiplet.

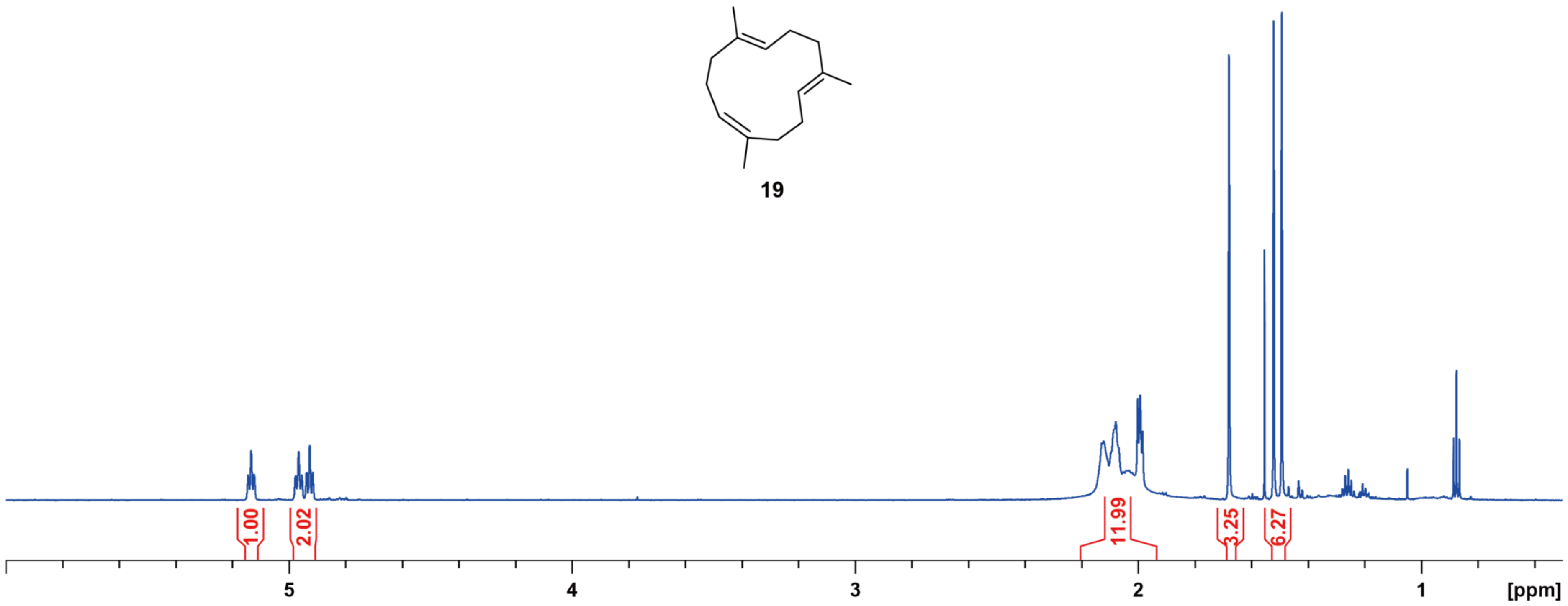

Figure S4. ^{1}H NMR spectrum of compound **19** (C_6D_6, 700 MHz).

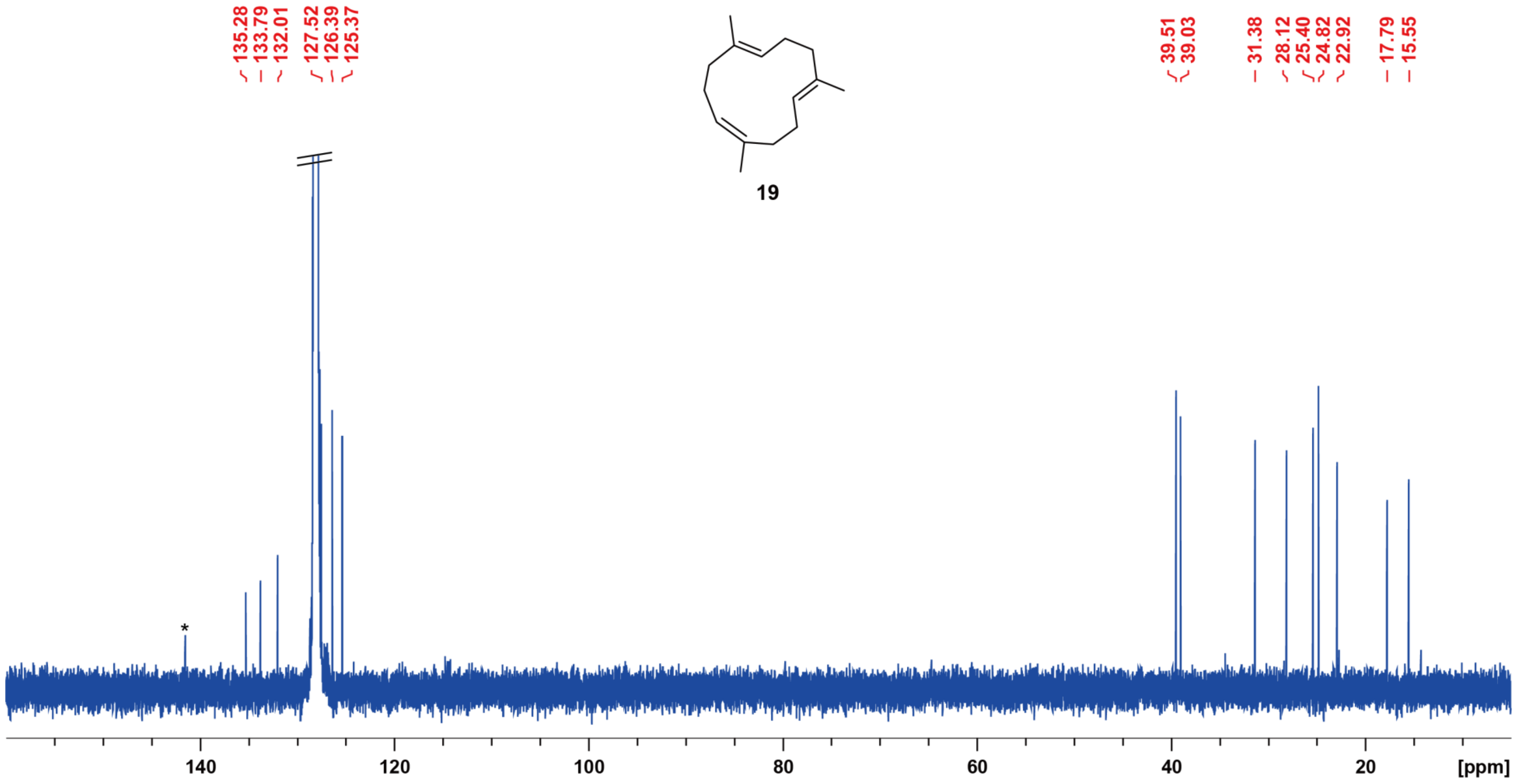

Figure S5. ^{13}C NMR spectrum of compound **19** (C_6D_6, 176 MHz). Asterisk indicates the peak of impurity from commercial C_6D_6.

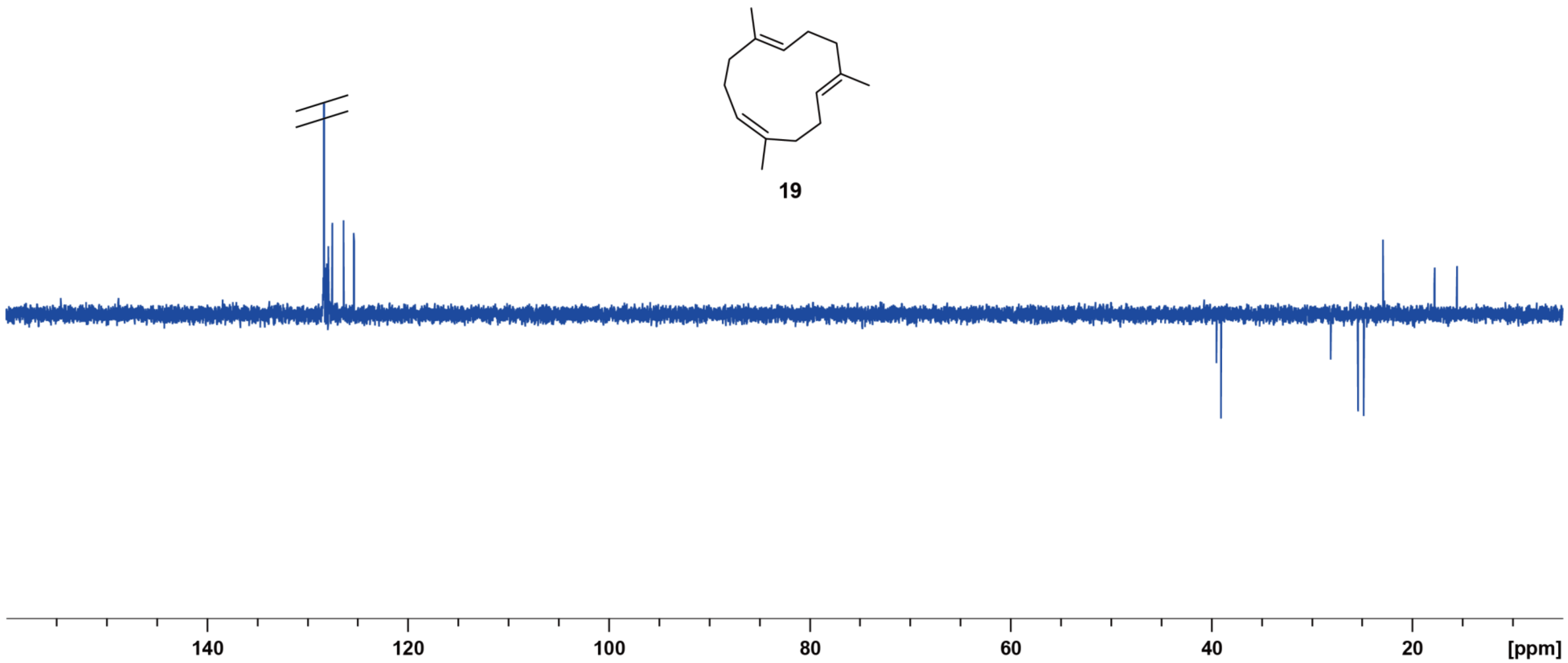

Figure S6. ^{13}C DEPT spectrum of compound **19** (C_6D_6, 176 MHz).

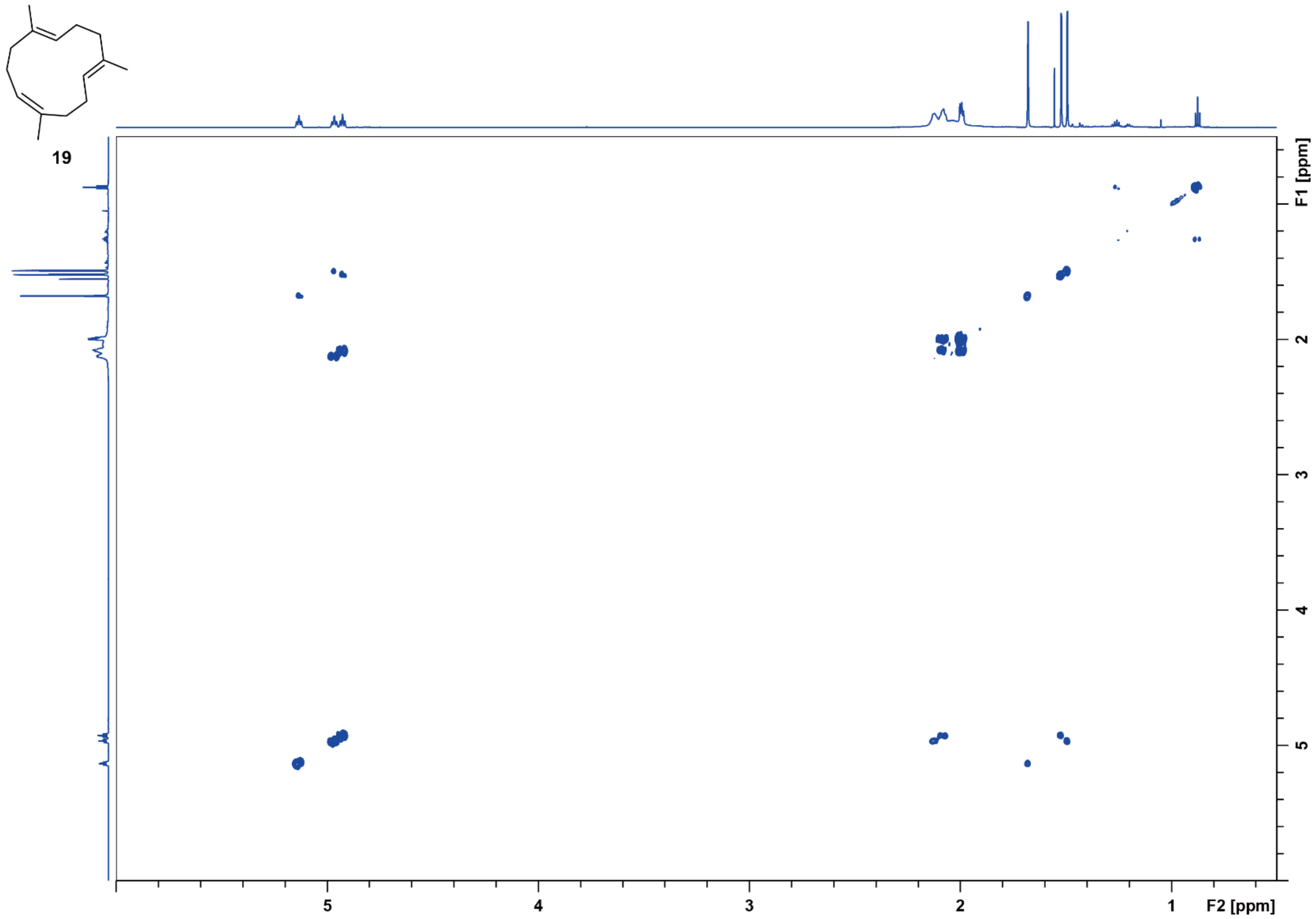

Figure S7. ^{1}H-^{1}H-COSY spectrum of compound **19** (C_6D_6, 700 MHz).

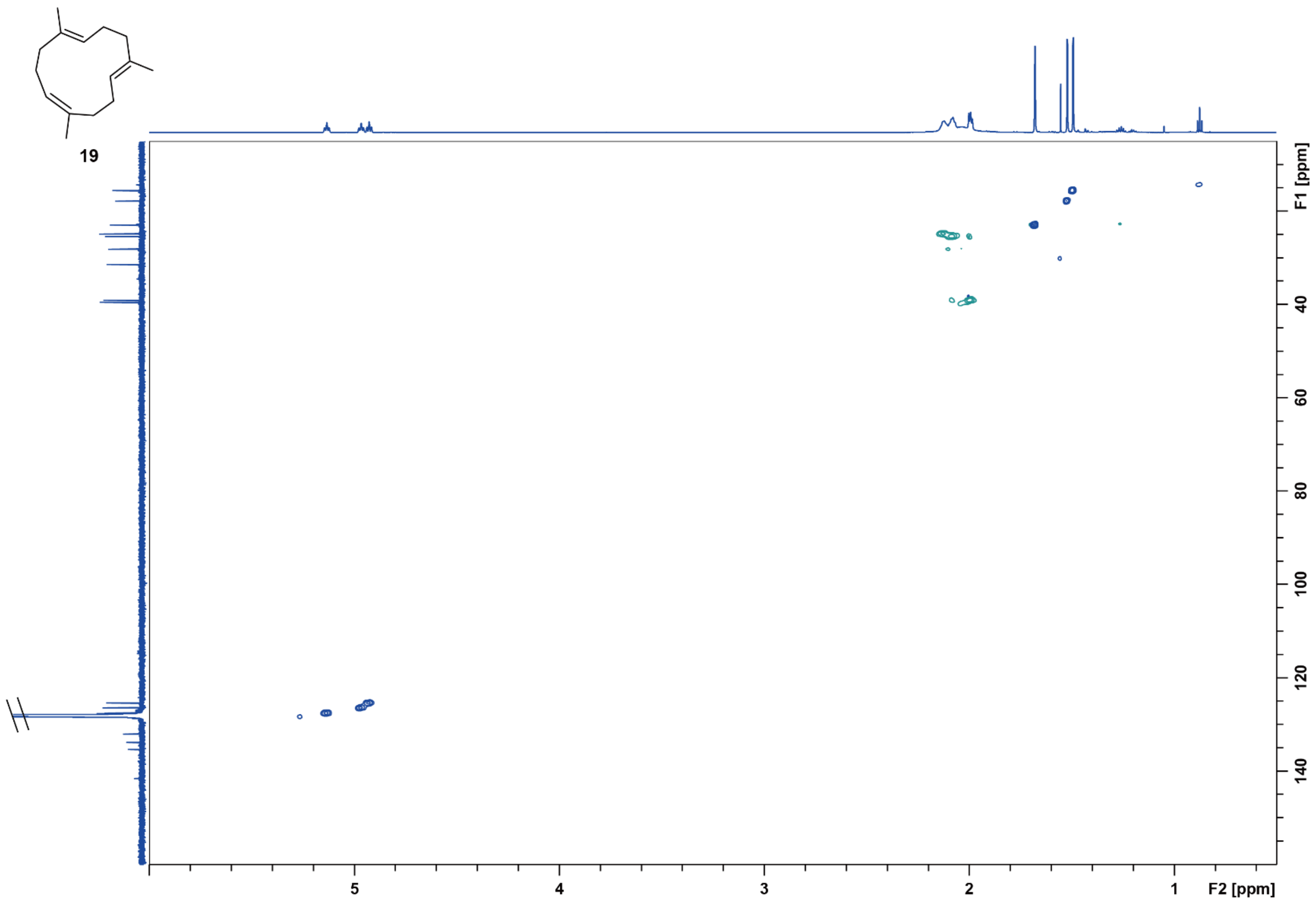

Figure S8. HSQC spectrum of compound **19** (C_6D_6).

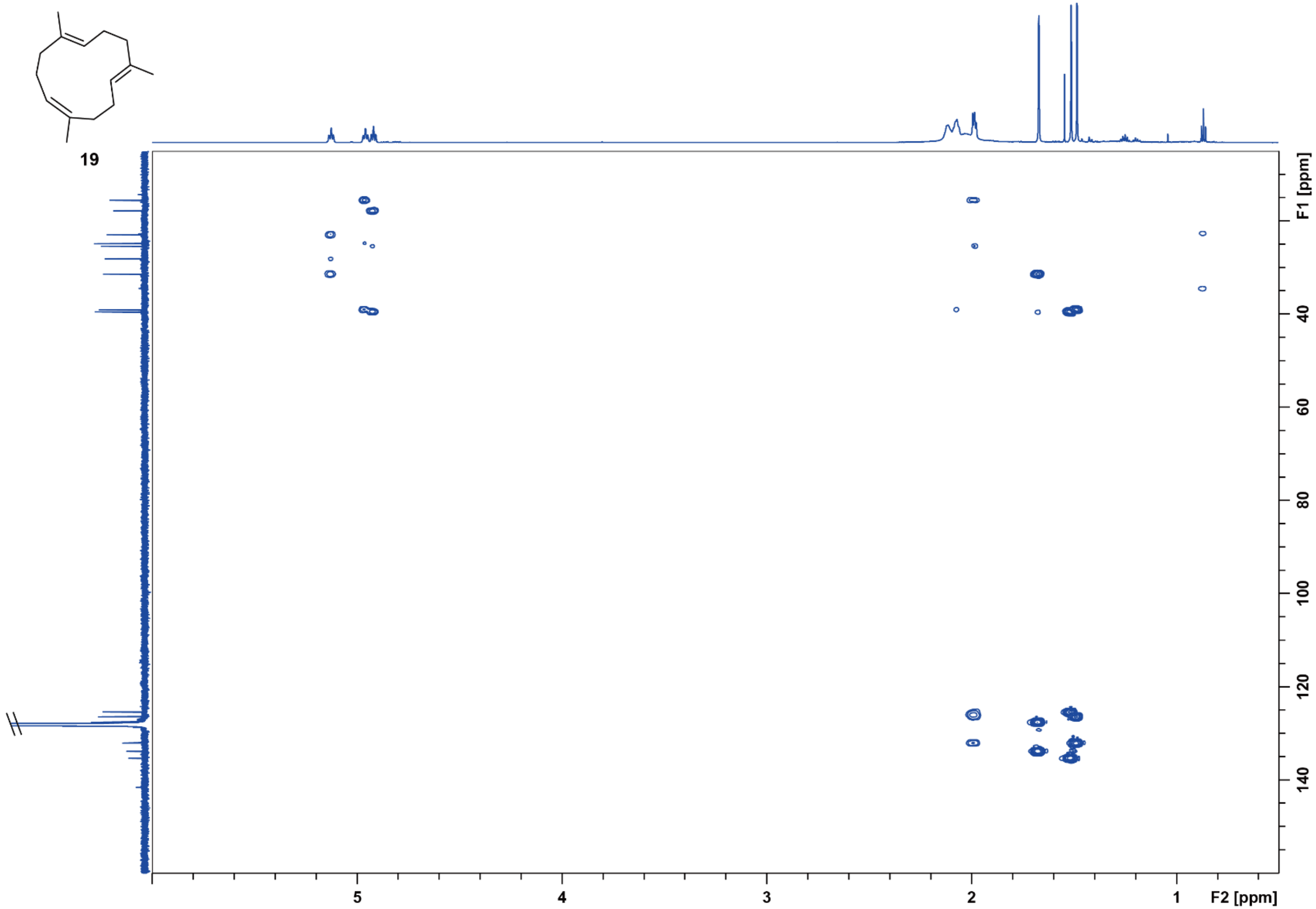

Figure S9. HMBC spectrum of compound **19** (C_6D_6).

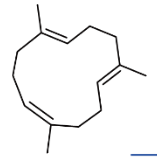

19

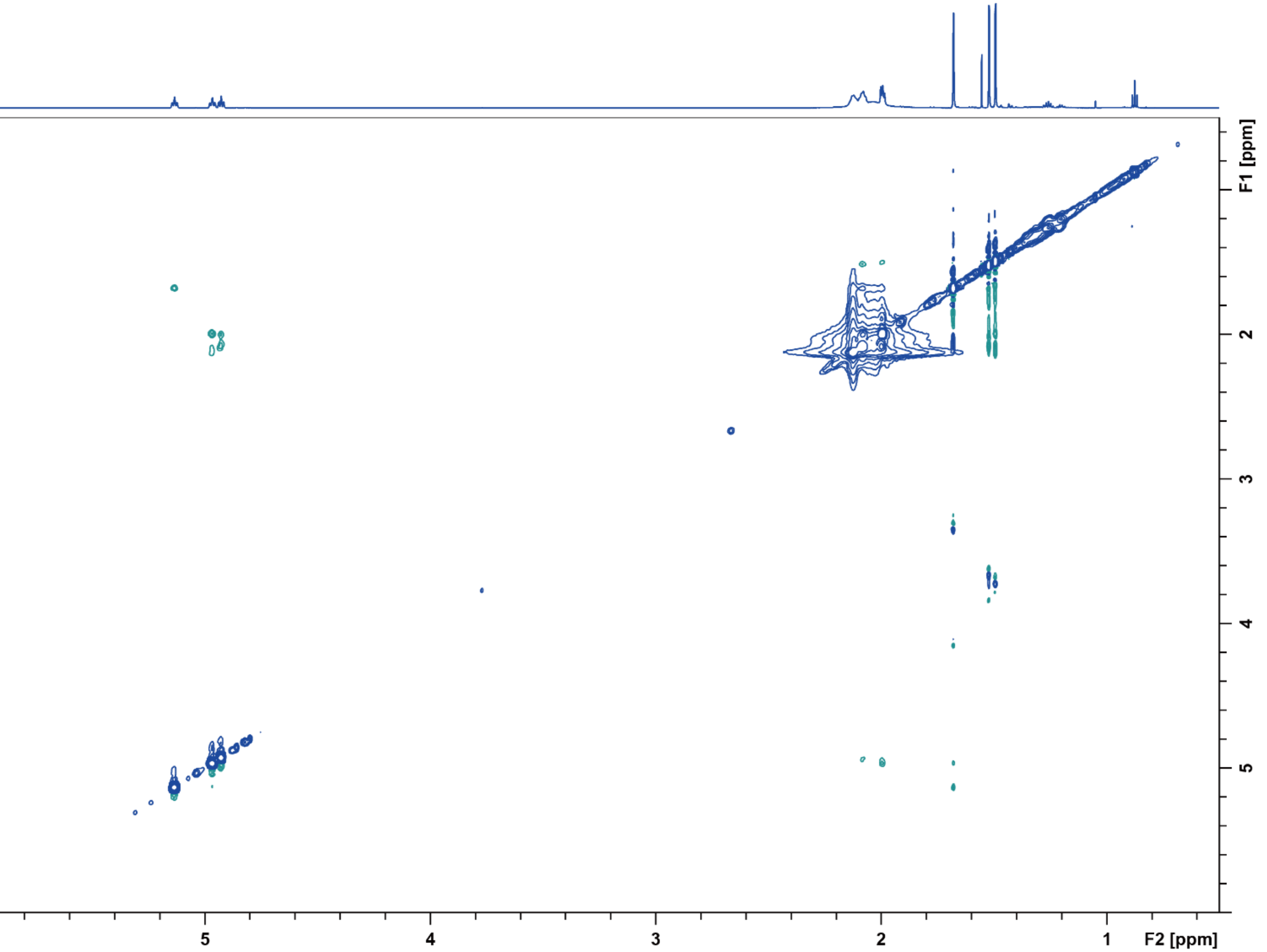

Figure S10. NOESY spectrum of compound **19** (C_6D_6, 700 MHz).

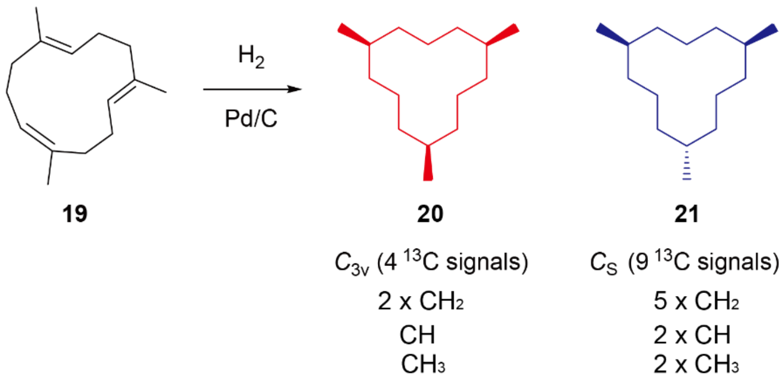

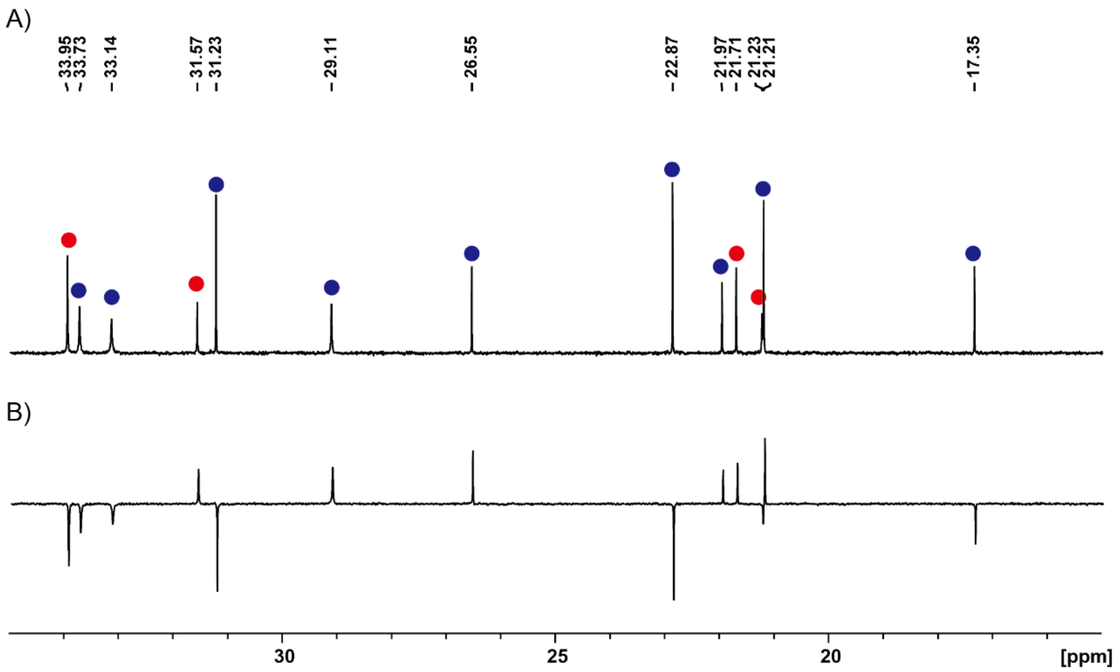

Figure S11. A) ^{13}C NMR spectrum and B) ^{13}C DEPT spectrum of hydrogenation product from **19**. Red dots indicate the signals from **20**, blue dots indicate the signals from **21**.

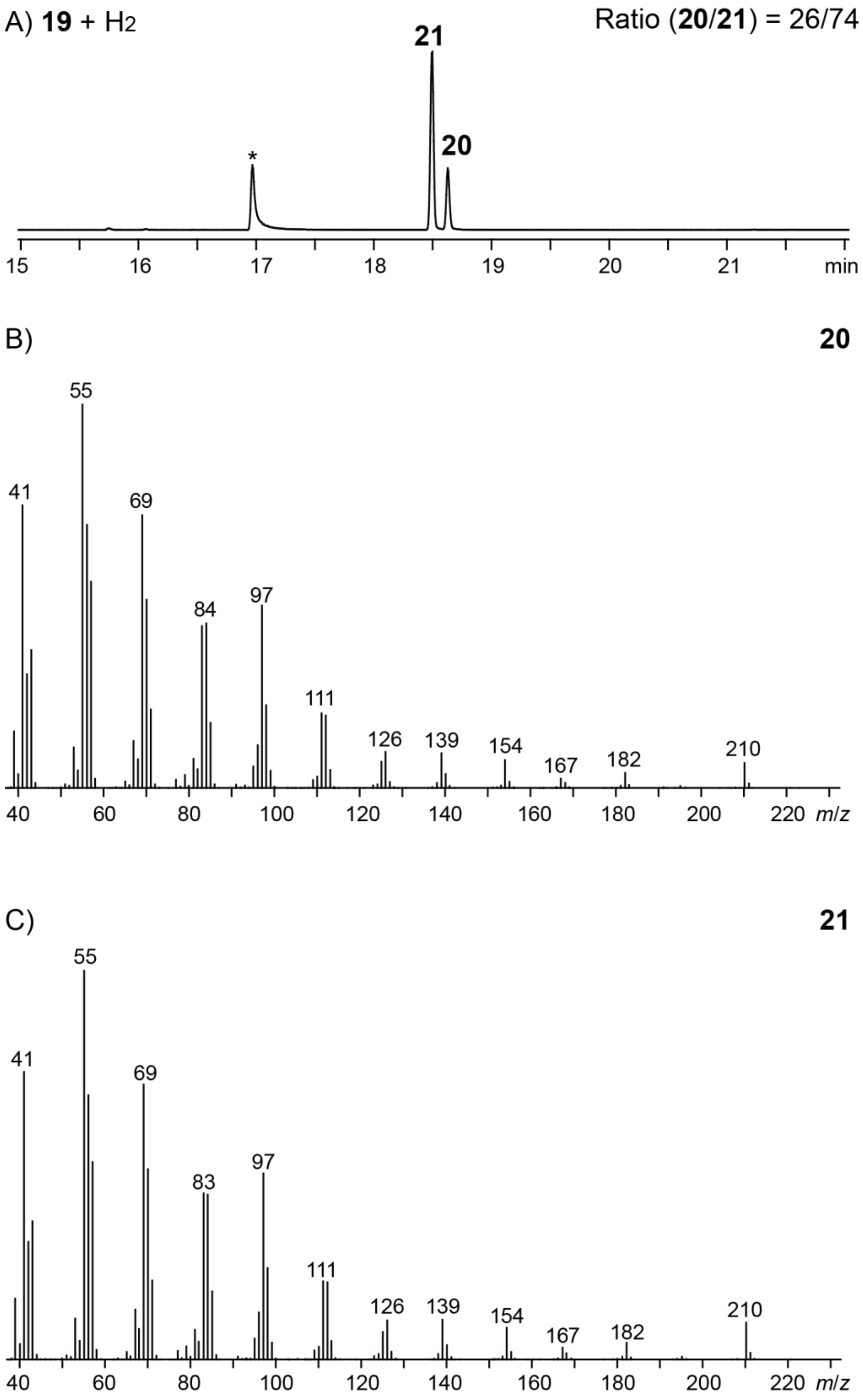

Figure S12. A) Total ion chromatogram of the hydrogenation products from **19**, and EI mass spectra of the products B) **20** and C) **21**. The asterisk indicates an impurity from the solvent.

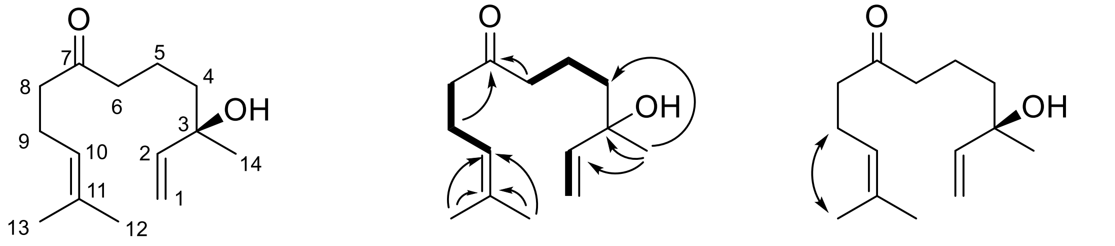

23

Figure S13. Structure elucidation of **23**. Bold: ^{1}H,^{1}H-COSY, single headed arrows: key HMBC, and double headed arrows: key NOESY correlations.

Table S2. NMR data of **23** in C_6D_6 recorded at 298 K.

C[a]	type	^{13}C[b]	^{1}H[b]
1	CH_2	111.50	5.19 (dd, J = 17.3, 1.6 Hz, 1H)
			4.94 (dd, J = 10.7, 1.6 Hz, 1H)
2	CH	145.59	5.74 (dd, J = 17.3, 10.7 Hz, 1H)
3	C_q	72.77	–
4	CH_2	41.87	1.31 (m, 2H)
5	CH_2	18.51	1.58 (m, 2H)
6	CH_2	42.83	1.97 (t, J = 7.2 Hz, 2H)
7	C_q	208.65	–
8	CH_2	42.62	2.06 (t, J = 7.4 Hz, 2H)
9	CH_2	22.91	2.27 (m, 2H)
10	CH	123.85	5.10 (thept, J = 7.3, 1.4 Hz, 1H)
11	C_q	132.16	–
12	CH_3	17.66	1.53 (d, J = 1.3 Hz, 3H)
13	CH_3	25.81	1.62 (d, J = 1.2 Hz, 3H)
14	CH_3	28.24	1.09 (s, 3H)

[a] Carbon numbering as shown in Figure S13. [b] Chemical shifts δ in ppm, multiplicity: s = singlet, d = doublet, t = triplet, hept = heptet, m = multiplet.

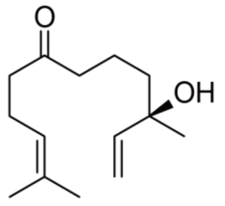

23

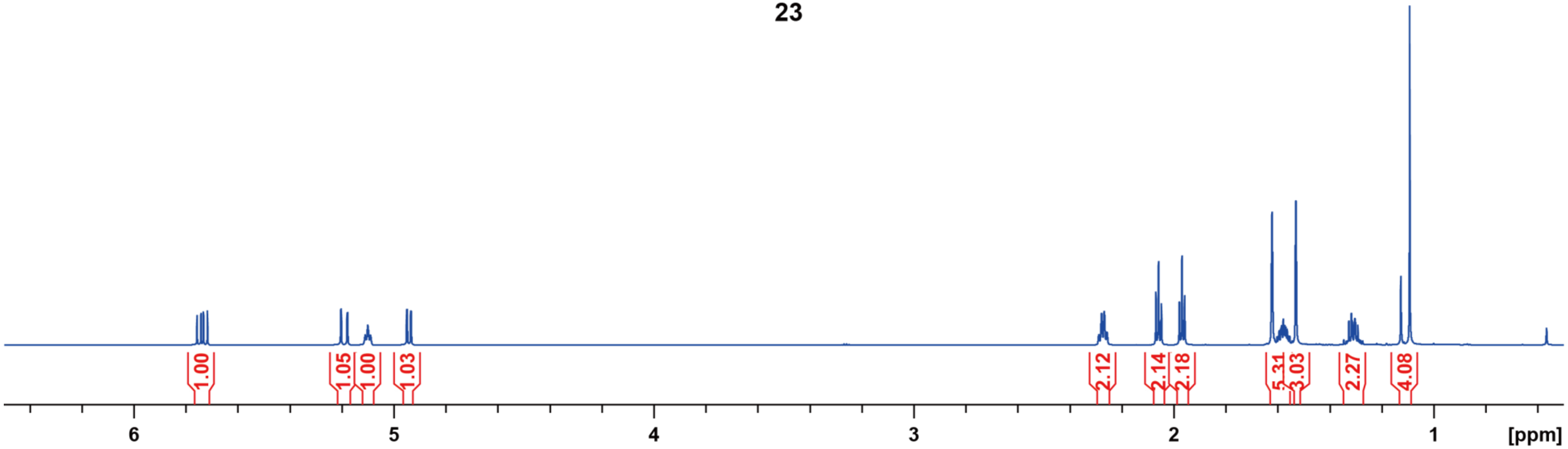

Figure S14. ^{1}H NMR spectrum of compound **23** (C_6D_6, 700 MHz).

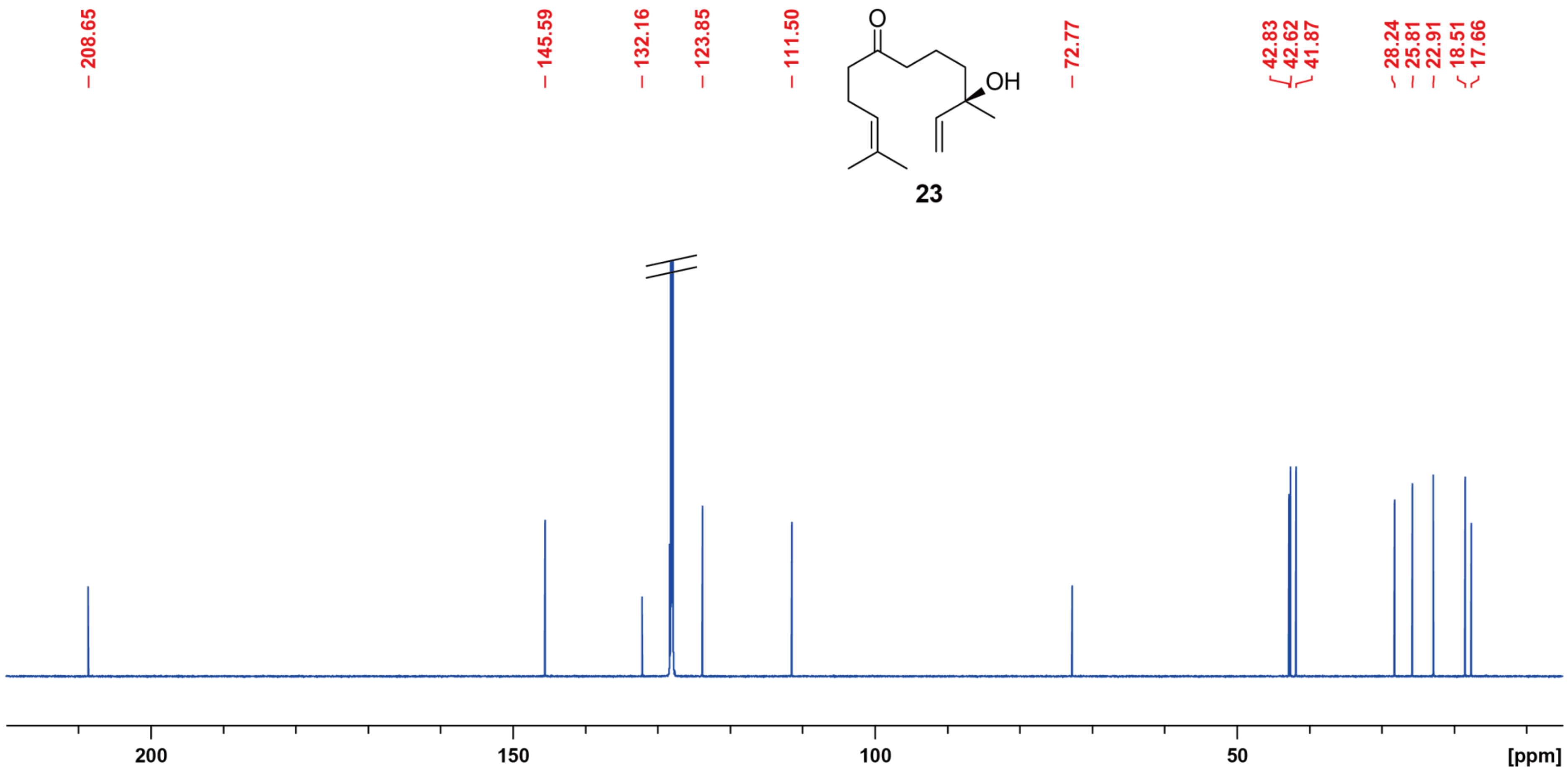

Figure S15. ^{13}C NMR spectrum of compound **23** (C_6D_6, 176 MHz).

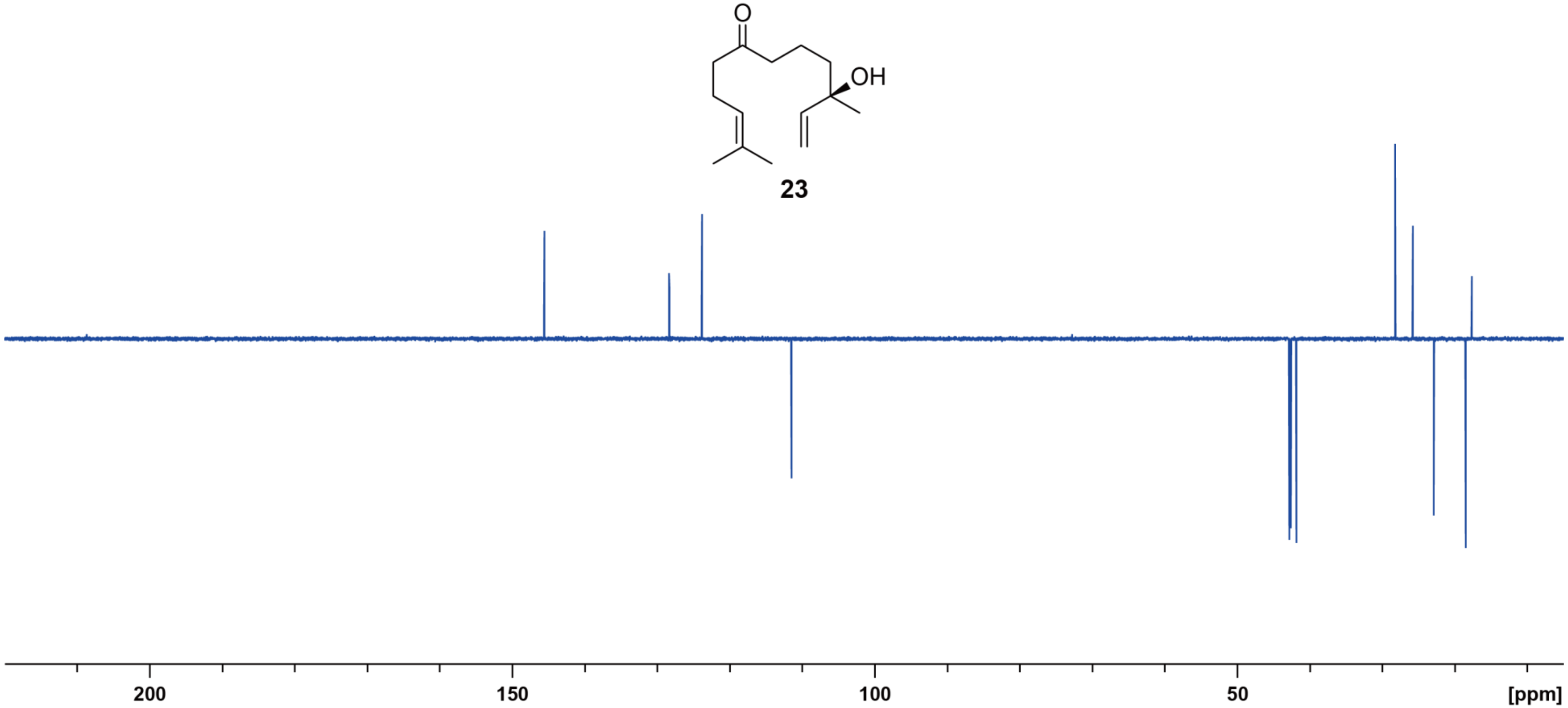

Figure S16. ^{13}C DEPT spectrum of compound **23** (C_6D_6, 176 MHz).

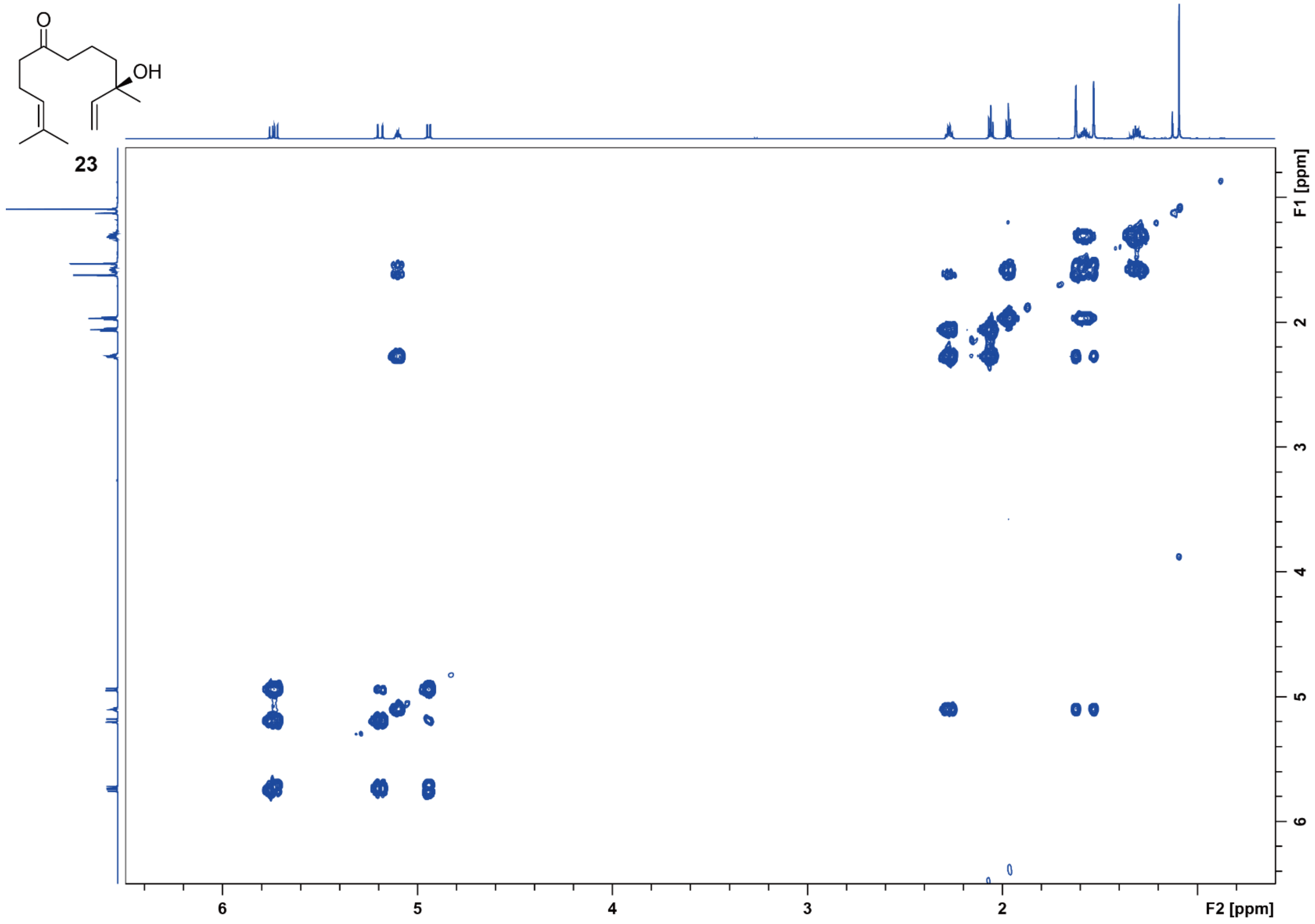

Figure S17. ^{1}H-^{1}H-COSY spectrum of compound **23** (C_6D_6, 700 MHz).

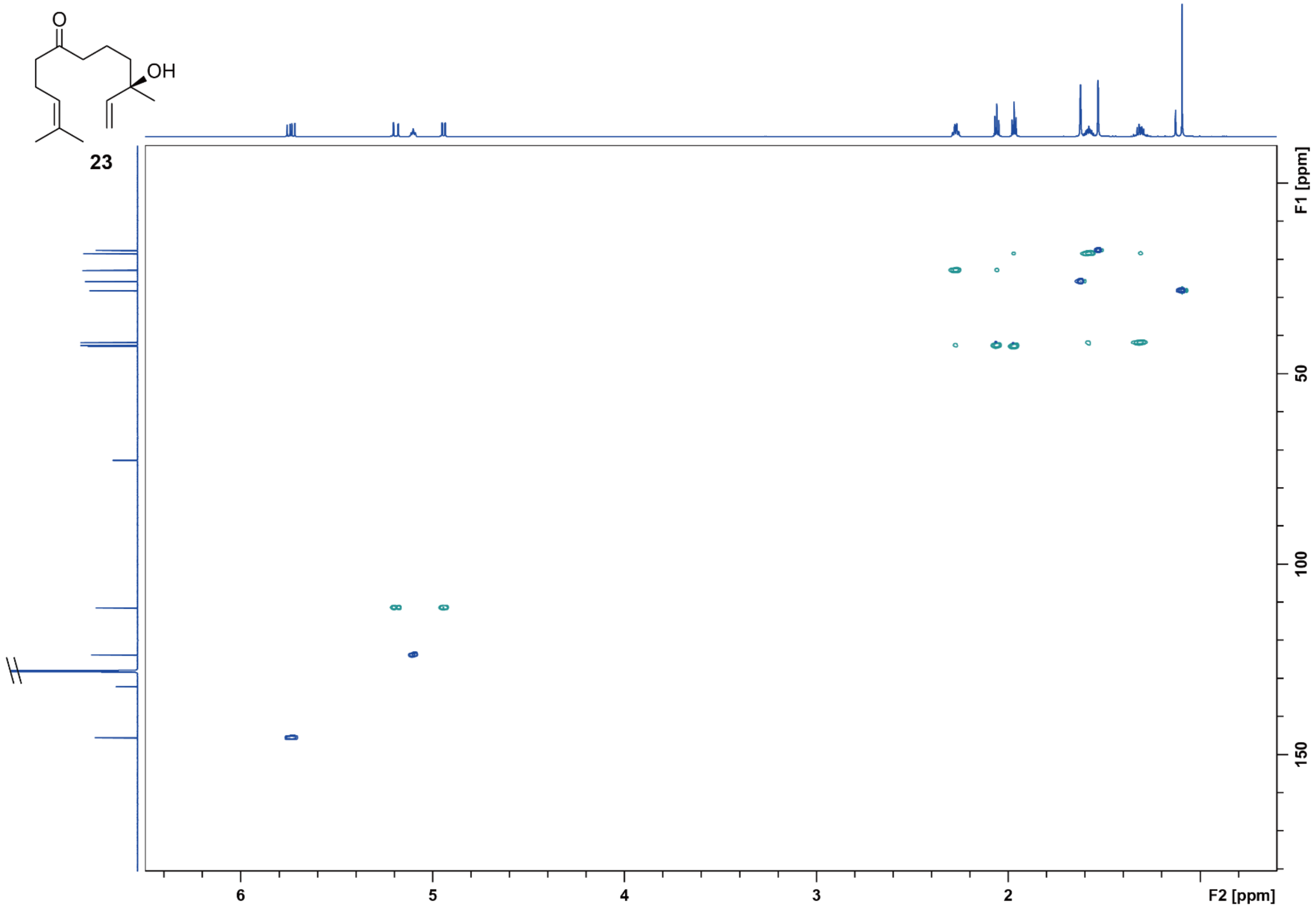

Figure S18. HSQC spectrum of compound **23** (C_6D_6).

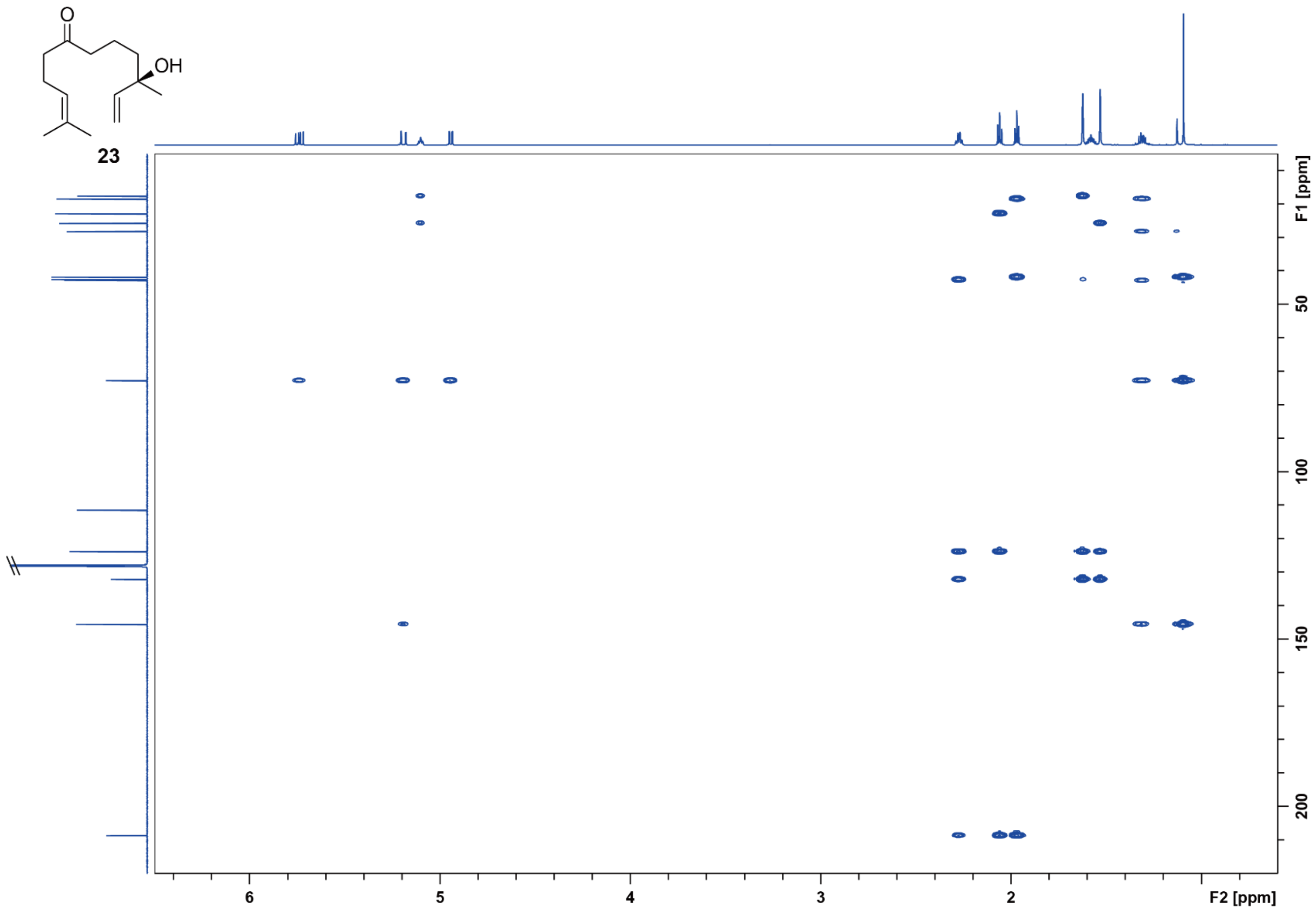

Figure S19. HMBC spectrum of compound **23** (C_6D_6).

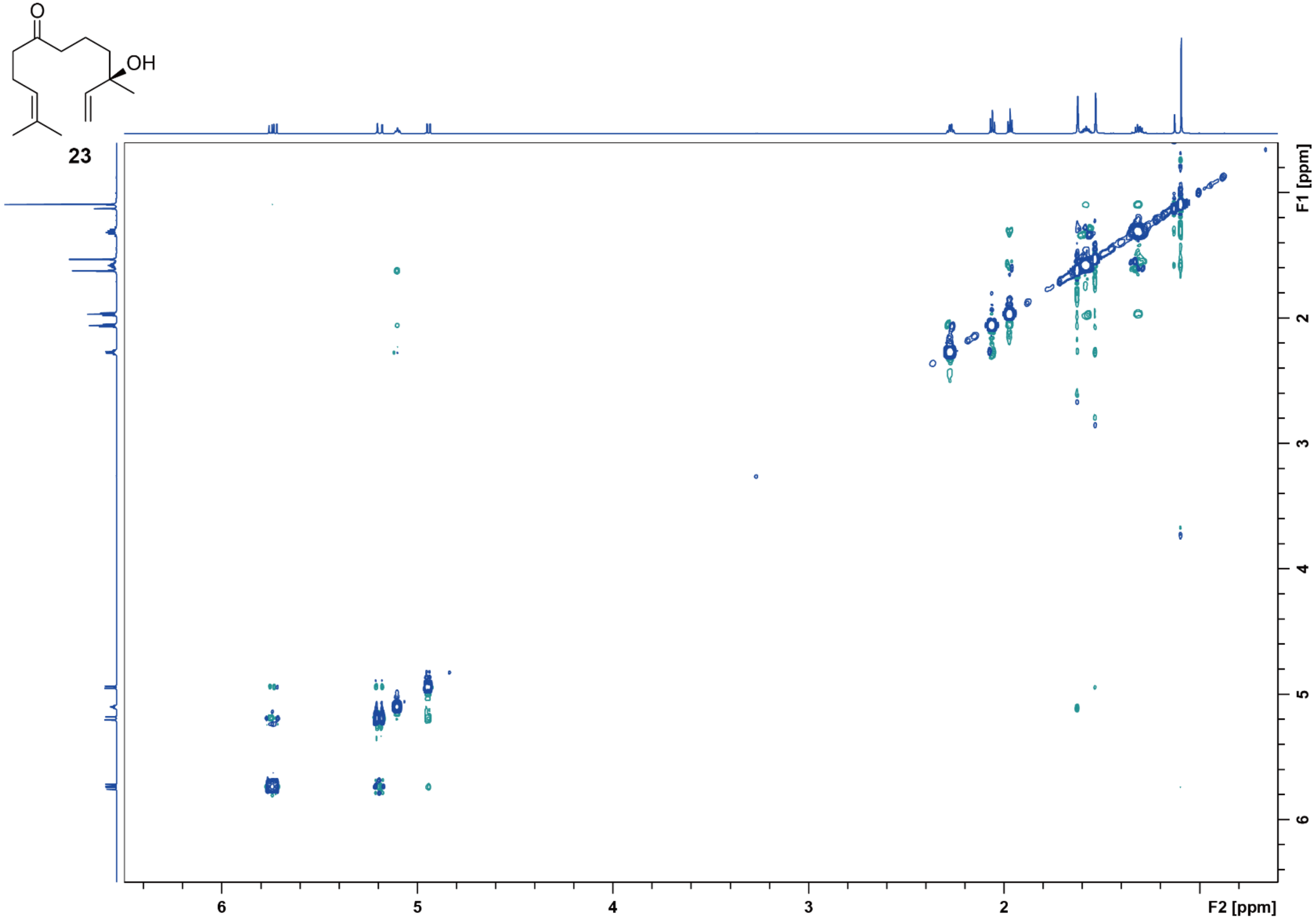

Figure S20. NOESY spectrum of compound **23** (C_6D_6, 700 MHz).

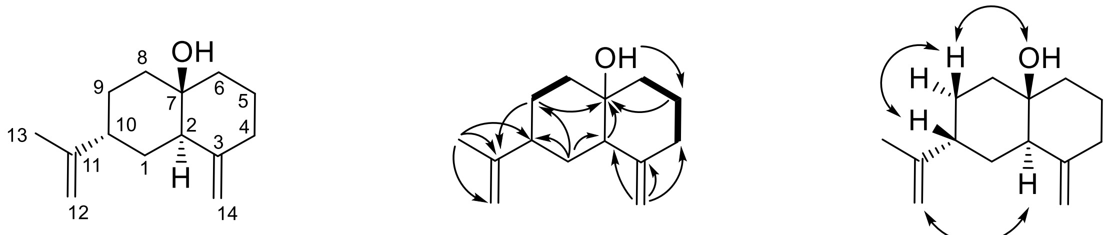

24

Figure S21. Structure elucidation of **24**. Bold: $^{1}H,^{1}H$-COSY, single headed arrows: key HMBC, and double headed arrows: key NOESY correlations.

Table S3. NMR data of **24** in C_6D_6 recorded at 298 K.

C[a]	type	^{13}C[b]	^{1}H[b]
1	CH_2	25.82	1.75 (m, 2H)
2	CH	44.40	2.14 (m, 1H)
3	C_q	150.35	–
4	CH_2	36.89	2.14 (m, 1H)
			1.78 (m, 1H)
5	CH_2	24.11	1.51 (m, 1H)
			1.43 (m, 1H)
6	CH_2	40.04	1.52 (m, 1H)
			1.15 (m, 1H)
7	C_q	71.81	–
8	CH_2	34.56	1.51 (m, 2H)
9	CH_2	23.14	2.06 (m, 1H)
			1.76 (m, 1H)
10	CH	38.84	2.31 (br s, 1H)
11	C_q	146.45	–
12	CH_2	111.06	4.95 (sext, J = 1.6 Hz, 1H)
			4.87 (d, J = 2.2 Hz, 1H)
13	CH_3	22.81	1.63 (s, 3H)
14	CH_2	108.31	4.81 (q, J = 1.7 Hz, 1H)
			4.62 (q, J = 1.6 Hz, 1H)

[a] Carbon numbering as shown in Figure S21. [b] Chemical shifts δ in ppm, multiplicity: s = singlet, d = doublet, q = quartet, sext = sextet, m = multiplet, br = broad.

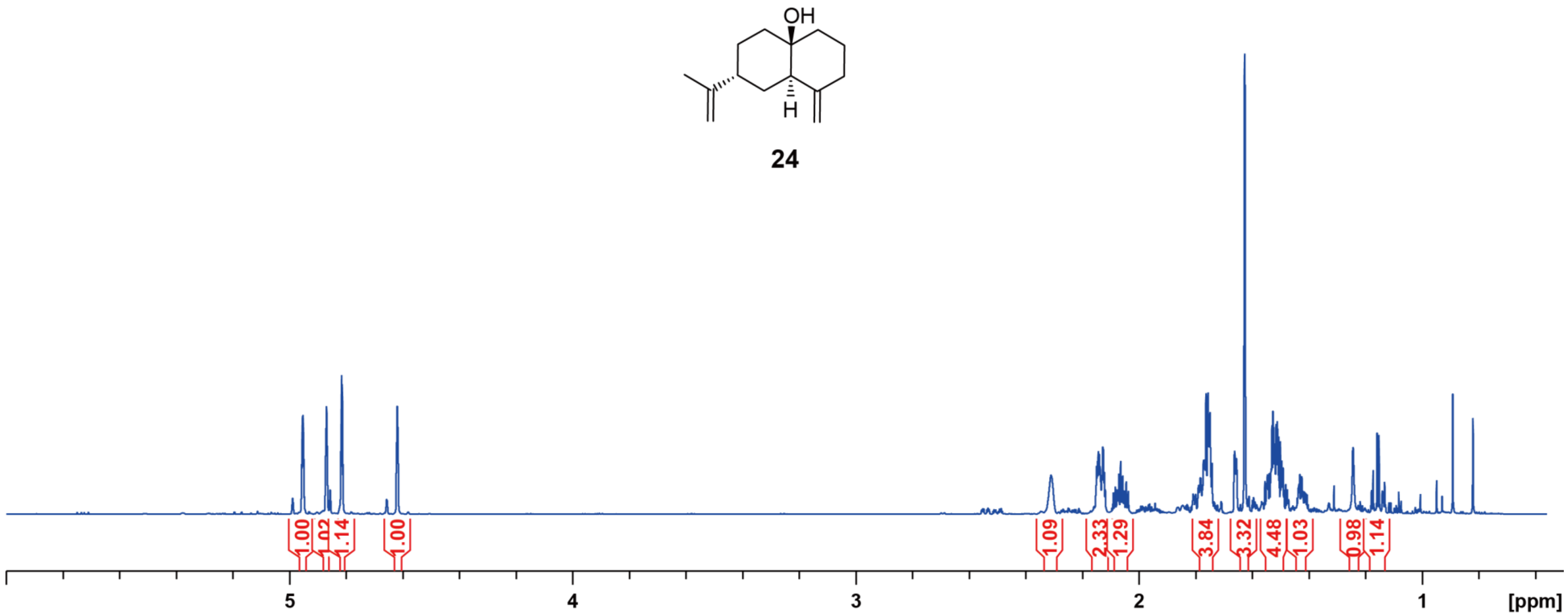

Figure S22. ^{1}H NMR spectrum of compound **24** (C_6D_6, 700 MHz).

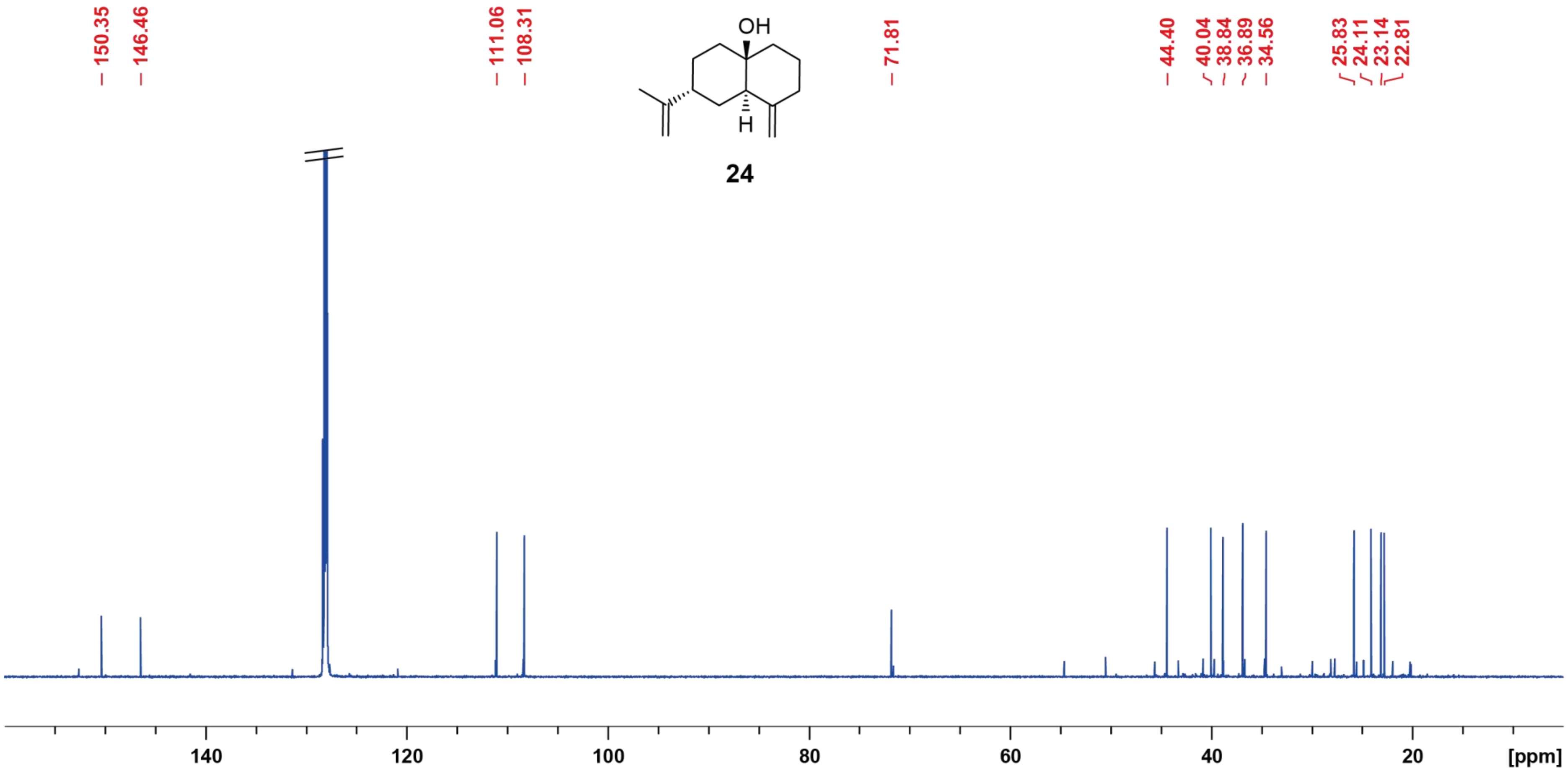

Figure S23. ^{13}C NMR spectrum of compound **24** (C_6D_6, 176 MHz).

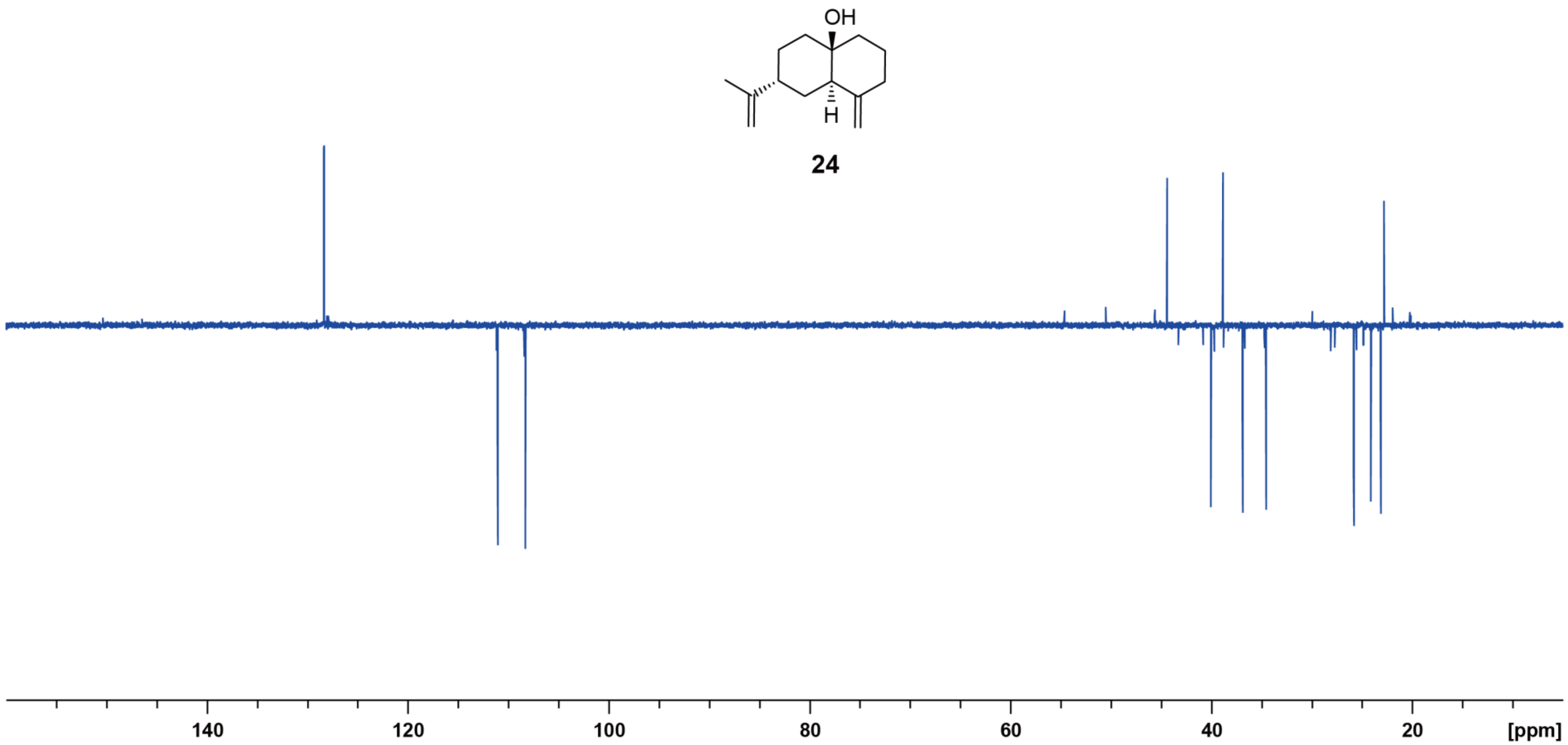

Figure S24. ^{13}C DEPT spectrum of compound **24** (C_6D_6, 176 MHz).

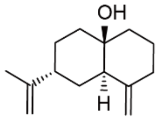

24

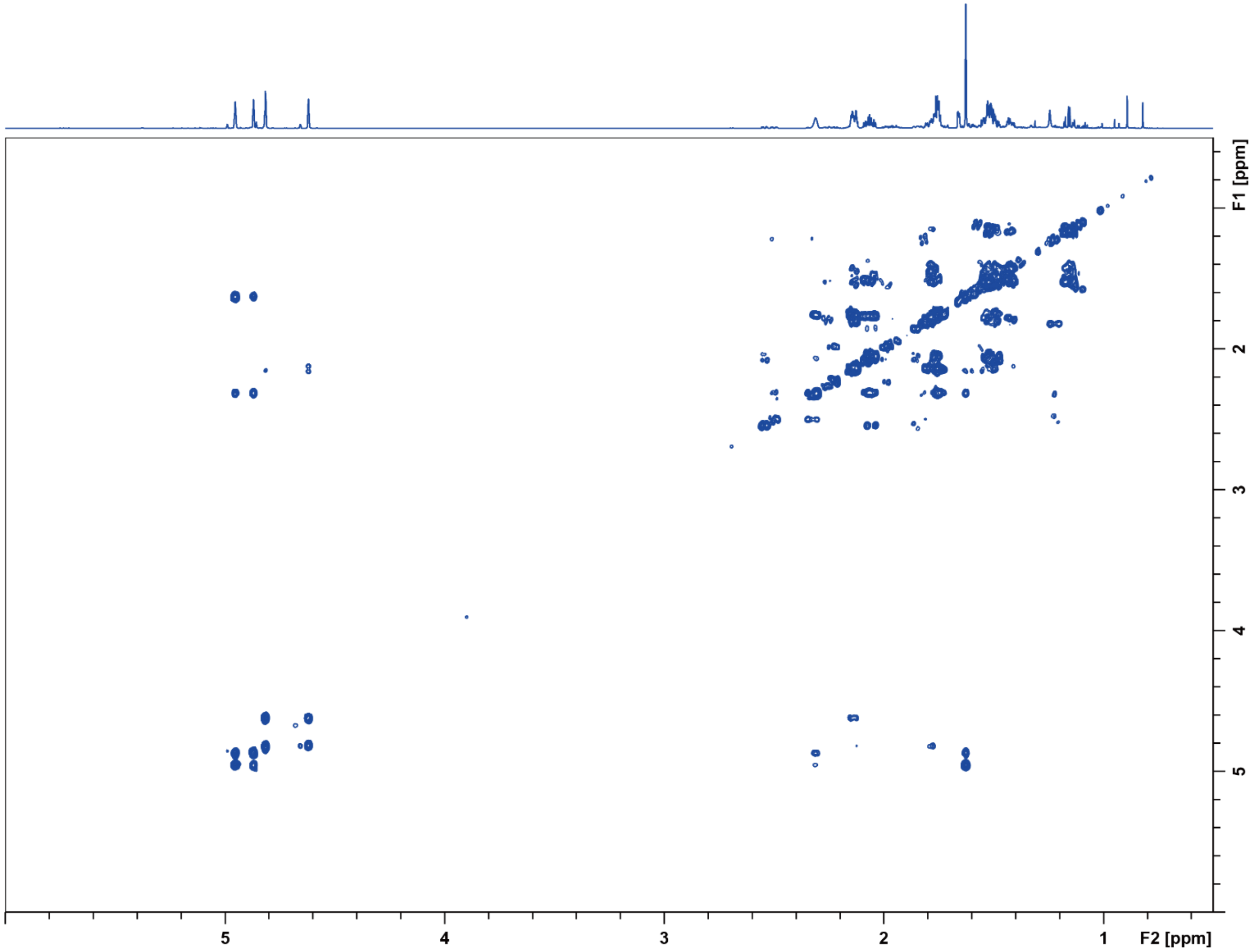

Figure S25. ^{1}H-^{1}H-COSY spectrum of compound **24** (C_6D_6, 700 MHz).

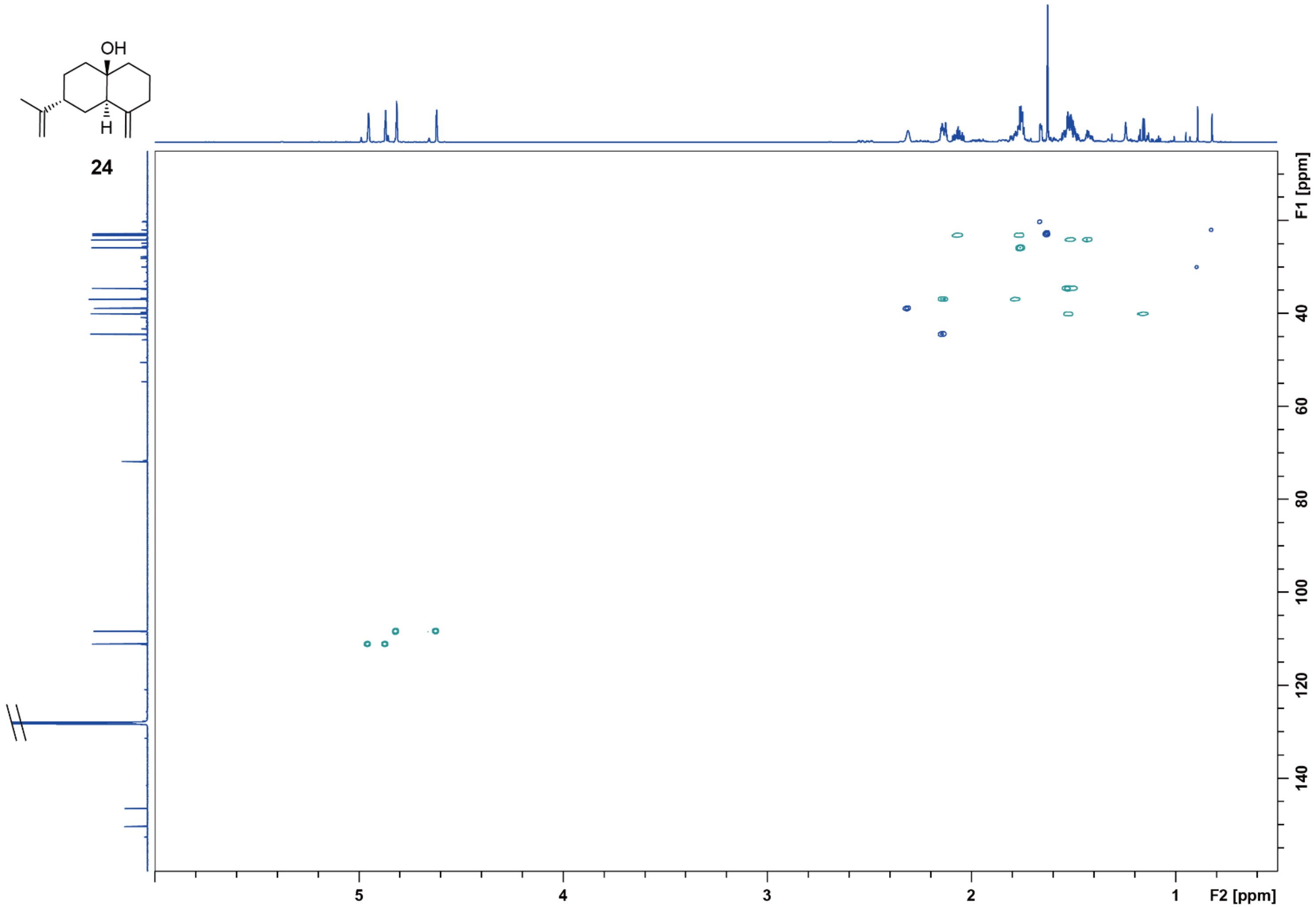

Figure S26. HSQC spectrum of compound **24** (C_6D_6).

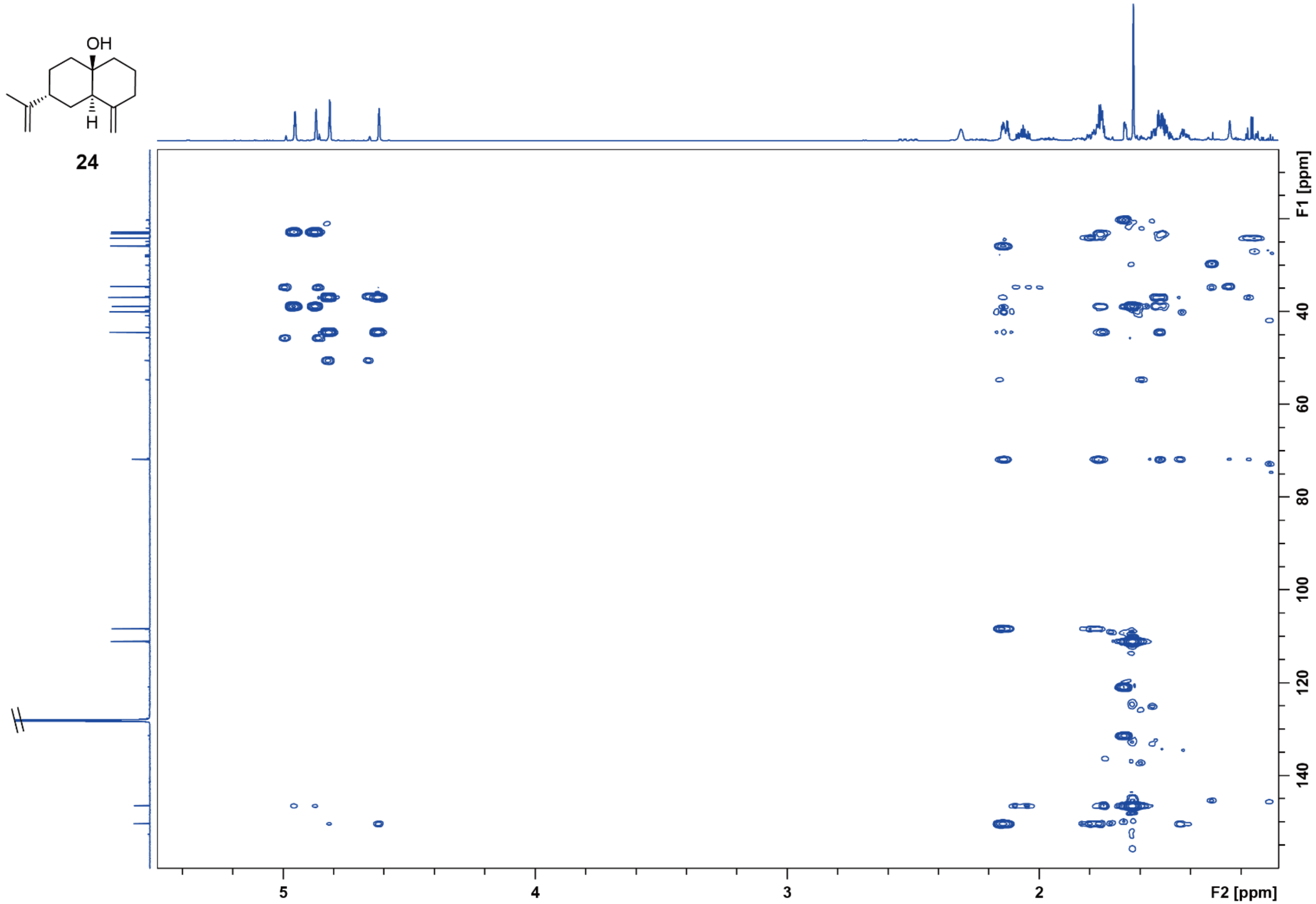

Figure S27. HMBC spectrum of compound **24** (C_6D_6).

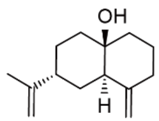

24

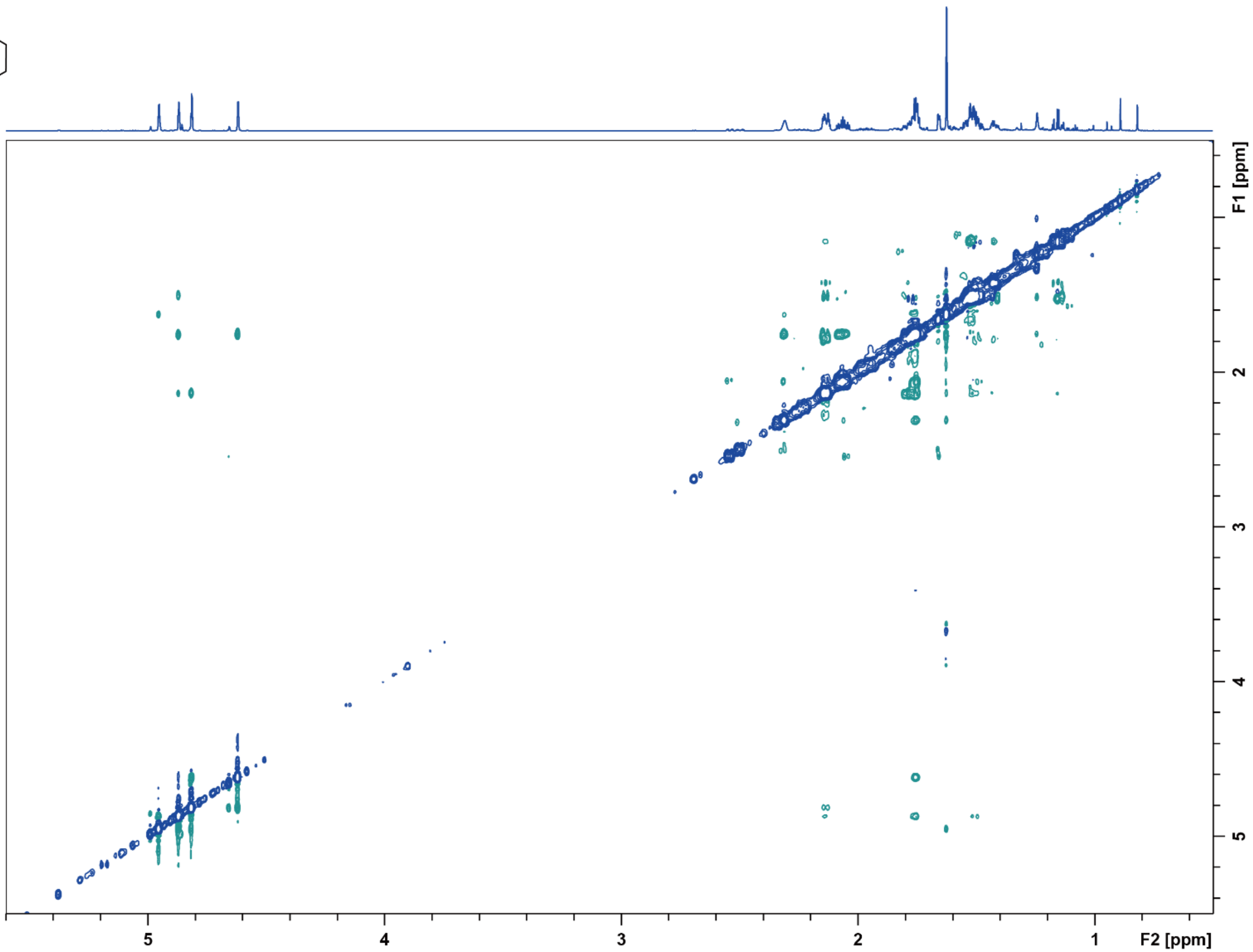

Figure S28. NOESY spectrum of compound **24** (C_6D_6, 700 MHz).

Catalytic hydrogenations

(R)-Nerolidol (ca. 0.1 mg, in 50 μL pentane) was mixed with methanol (1 mL) and Pd/C (4.5 mg, 5 % Pd) in a flask. The flask was vacuumed shortly and flushed with H_2 by using a balloon of hydrogen, the process was repeated three times to exchange the atmosphere to H_2 totally. The mixture was stirred at room temperature for 30 min. The catalyst was filtered off and washed with CH_2Cl_2 (1 mL). The filtrate was concentrated and redissolved in ethyl acetate or C_6D_6, and analysed by GC/MS or NMR.

(S)-Nerolidol and compounds **19**, **27**, **33** and **34** were catalytically hydrogenated by the similar procedure.

Conversion of 23 into 27 by Wittig reaction

CH_3PPh_3I (0.20 g, 0.5 mmol) was suspended in THF (10 mL). The mixture was cooled to 0 °C, followed by the dropwise addition of *n*-BuLi (0.3 mL, 1.6 M in hexane, 0.48 mmol). The mixture was stirred at 0 °C for 1 h. Then 4 mL of the mixture was transferred to a new flask at 0 °C, and compound **23** (5 mg, 0.022 mmol) was added dropwise to the mixture. The reaction mixture was stirred at 0 °C for 2 h and at room temperature for 3 h. The reaction was quenched by pouring onto ice-water (20 mL). The product was extracted with Et_2O (3 x 20 mL). The combined extracts were washed with brine and concentrated under reduced pressure. The product **27** was purified via silica gel chromatography (pentane/Et_2O, 1:1).

(*R*)-3,11-Dimethyl-7-methylenedodeca-1,10-dien-3-ol (27): Yield: 1.9 mg, 0.009 mmol, 38%. TLC (pentane/Et_2O, 1:1): R_f = 0.73. Optical rotation: $[\alpha]_D^{25}$ = –2.1 (*c* 0.19, CH_2Cl_2). EI-MS (70 eV): *m*/*z* (%) = 204 (1), 189 (4), 175 (4), 161 (8), 149 (2), 135 (12), 122 (10), 107 (20), 93 (49), 81 (26), 69 (100), 53 (16), 41 (73). GC (HP5-MS): *I* = 1563. HRMS (APCI): *m*/*z* = 205.1960 (calc. for $[C_{15}H_{26}O - OH]^+$: 205.1951). IR (diamond ATR): $\tilde{\nu}$ = 3408 (br w), 3083 (w), 2967 (m), 2926 (s), 2855 (m), 2279 (w), 2159 (w), 1738 (w), 1724 (w), 1673 (w), 1644 (w), 1566 (w), 1440 (m), 1411 (w), 1375 (m), 1345 (w), 1261 (w), 1217 (w), 1105 (m), 995 (m), 920 (m), 889 (m), 816 (w), 804 (w), 746 (w), 723 (w), 693 (w), 627 (w), 587 (w), 544 (s), 466 (w), 437 (w) cm^{-1}. NMR data cf. Table S4 and Figures S30 – S37.

GC analysis using a chiral stationary phase

Enantioselective GC analyses were performed on an Agilent 7820A GC system (Agilent, Santa Clara, CA, USA) equipped with an FID detector and a Cyclosil-B capillary column (Agilent, 30 m, 0.25 mm i. d., 0.25 μm film). For analysis of **26a** and **26b**, the GC program was: starting from 100 °C, increasing with 0.5 °C/min to 130 °C, followed by increasing with 20 °C/min to 245 °C, then holding the temperature for 5 min. For analysis of (R)- and (S)-**23**, the GC program was: starting from 120 °C, increasing with 0.5 °C/min to 150 °C, followed by increasing with 20 °C/min to 245 °C, then holding the temperature for 5 min. Inlet temperature was 250 °C, inject volume was 1 – 2 μL, split ratio was 1/1 – 1/50, the carrier gas was H_2 at 2.3 mL/min.

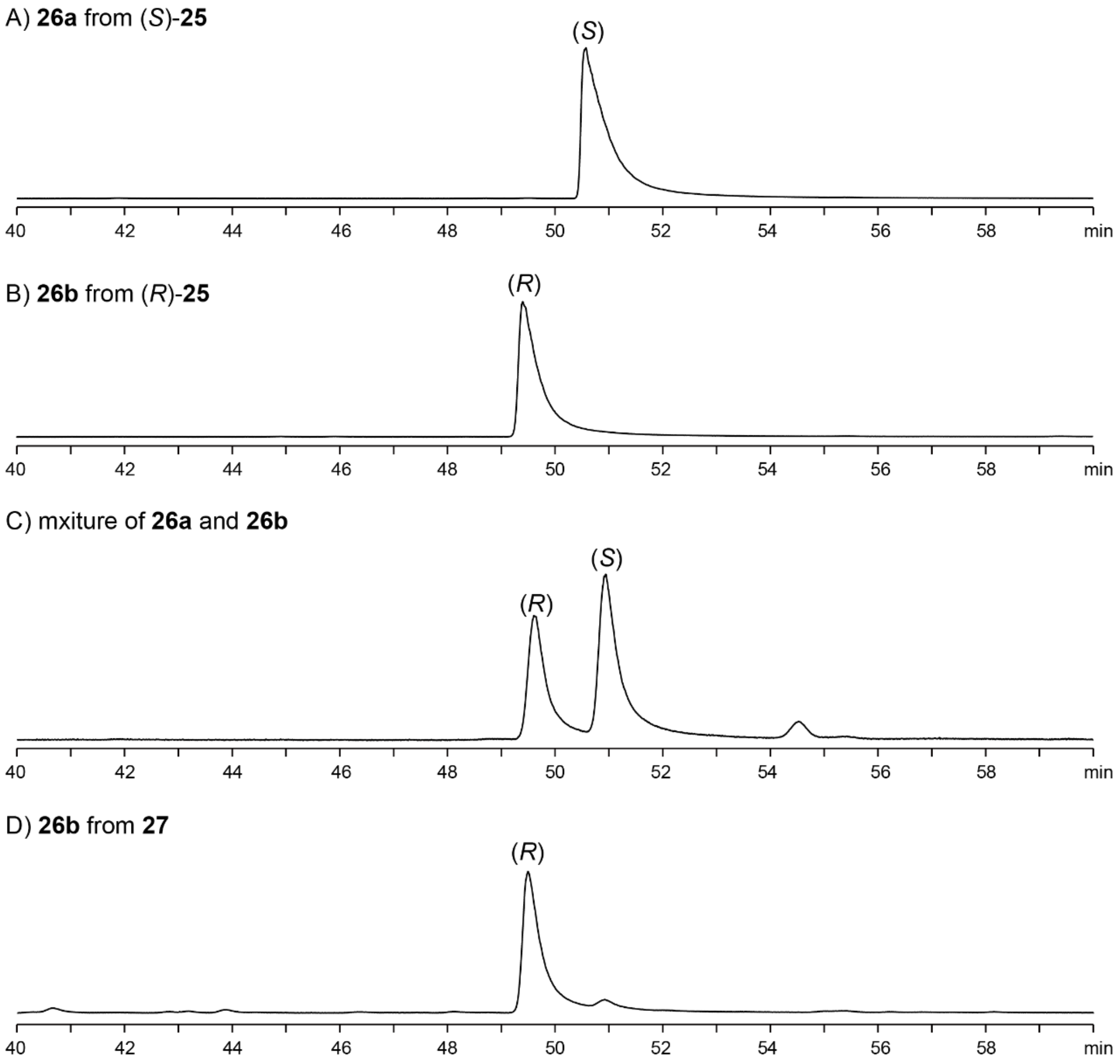

Figure S29. Determination of the absolute configuration of **23**. The gas chromatograms using a chiral stationary phase of A) hydrogenation product **26a** from (*S*)-nerolidol (**25**), B) hydrogenation product **26b** from (*R*)-nerolidol (**25**), C) mixture of compounds **26a** and **26b**, D) hydrogenation product **26b** from **27** obtained by Wittig reaction from enzymatically generated **23**.

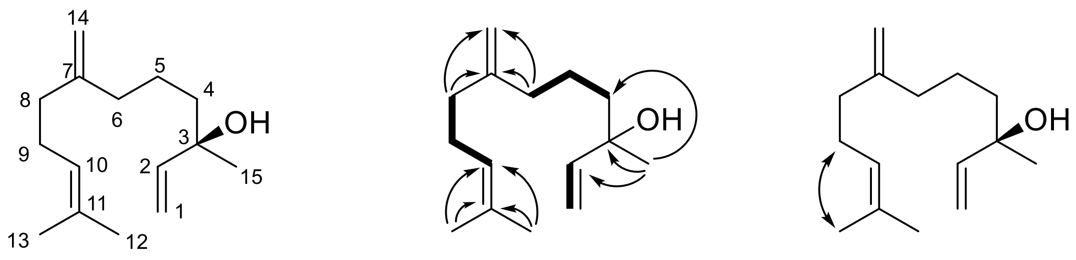

27

Figure S30. Structure elucidation of **27**. Bold: ^{1}H,^{1}H-COSY, single headed arrows: key HMBC, and double headed arrows: key NOESY correlations.

Table S4. NMR data of **27** in C_6D_6 recorded at 298 K.

C[a]	type	^{13}C[b]	^{1}H[b]
1	CH_2	111.31	5.16 (dd, *J* = 17.3, 1.6 Hz, 1H)
			4.93 (dd, *J* = 10.7, 1.6 Hz, 1H)
2	CH	145.77	5.74 (dd, *J* = 17.3, 10.7 Hz, 1H)
3	C_q	72.79	–
4	CH_2	42.30	1.39 (m, 2H)
5	CH_2	22.33	1.50 (m, 2H)
6	CH_2	36.85	1.99 (t, *J* = 7.5 Hz, 2H)
7	C_q	149.49	–
8	CH_2	36.40	2.09 (dd, *J* = 9.2, 6.3 Hz, 2H)
9	CH_2	26.93	2.20 (m, 2H)
10	CH	124.83	5.23 (m, 1H)
11	C_q	131.37	–
12	CH_3	25.85	1.67 (d, *J* = 1.3 Hz, 3H)
13	CH_3	17.75	1.56 (d, *J* = 1.2 Hz, 3H)
14	CH_2	109.57	4.87 (d, *J* = 7.0 Hz, 2H)
15	CH_3	28.24	1.09 (s, 3H)

[a] Carbon numbering as shown in Figure S30. [b] Chemical shifts δ in ppm, multiplicity: s = singlet, d = doublet, t = triplet, m = multiplet.

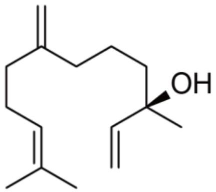

27

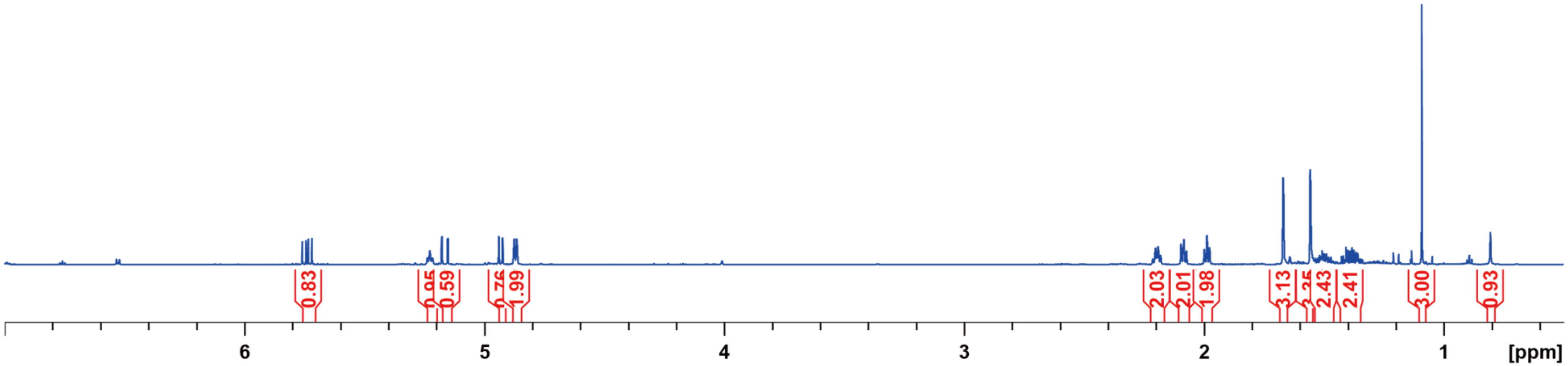

Figure S31. ^{1}H NMR spectrum of compound **27** (C_6D_6, 700 MHz).

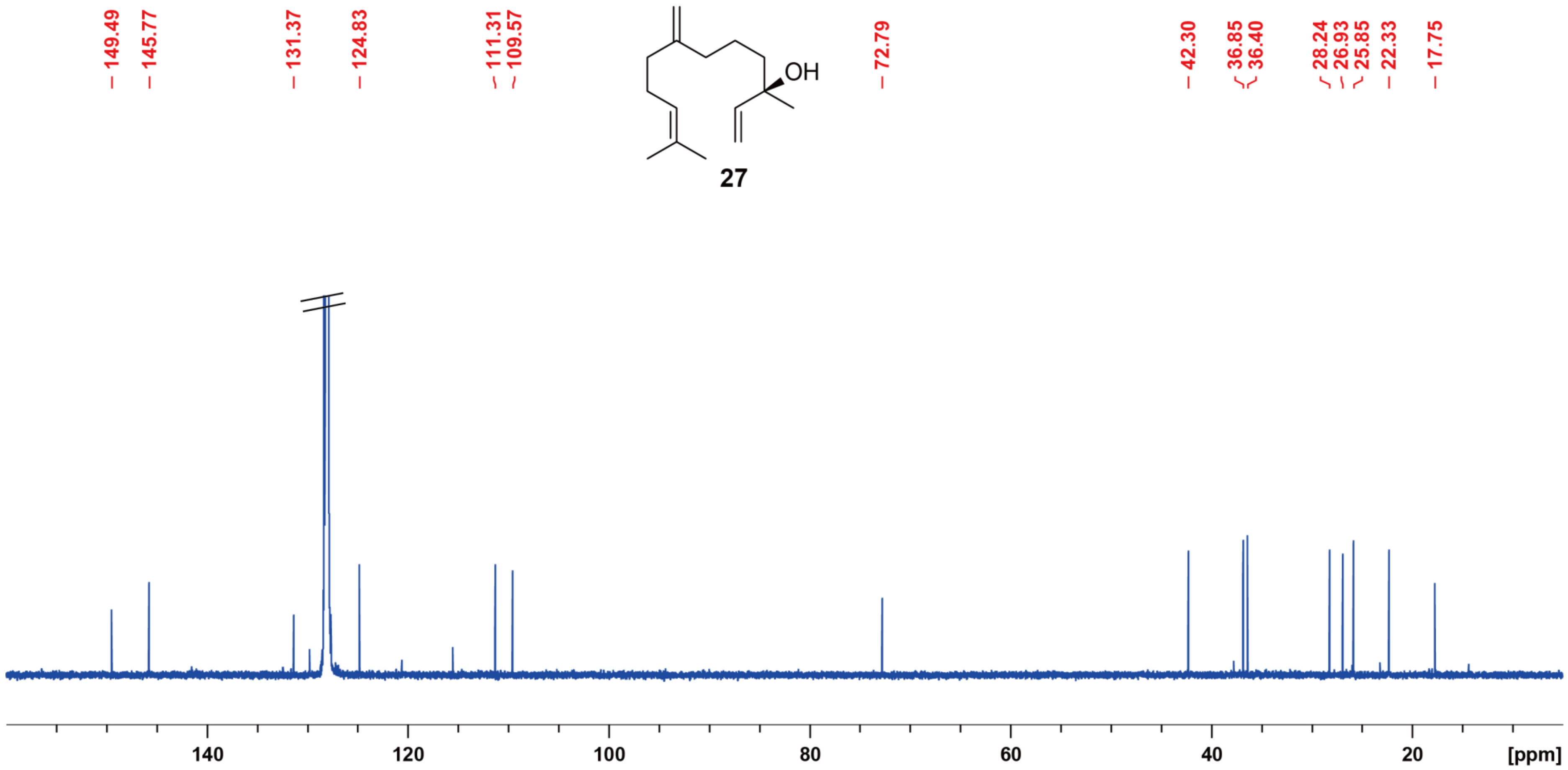

Figure S32. ^{13}C NMR spectrum of compound **27** (C_6D_6, 176 MHz).

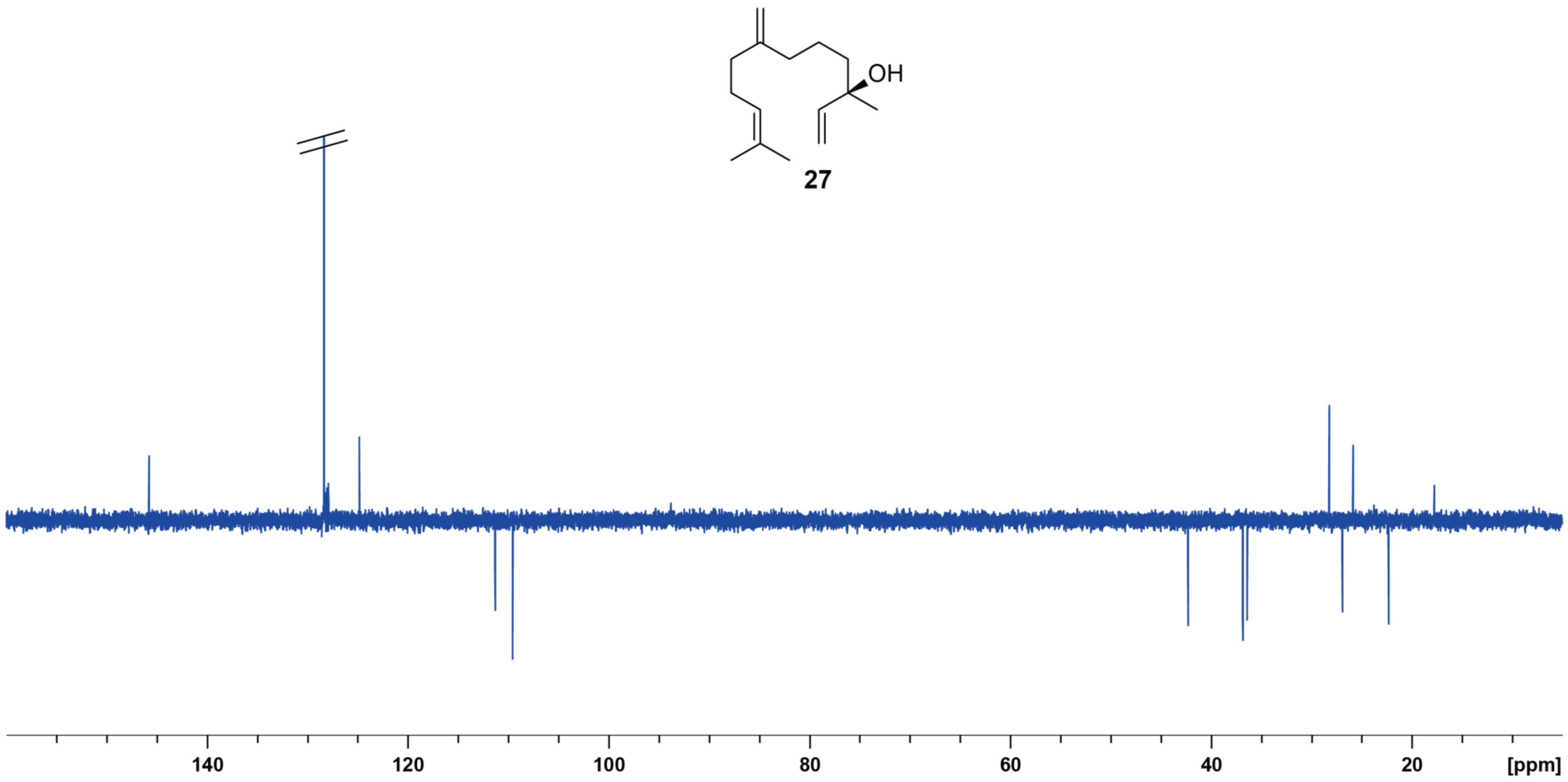

Figure S33. ^{13}C DEPT spectrum of compound **27** (C_6D_6, 176 MHz).

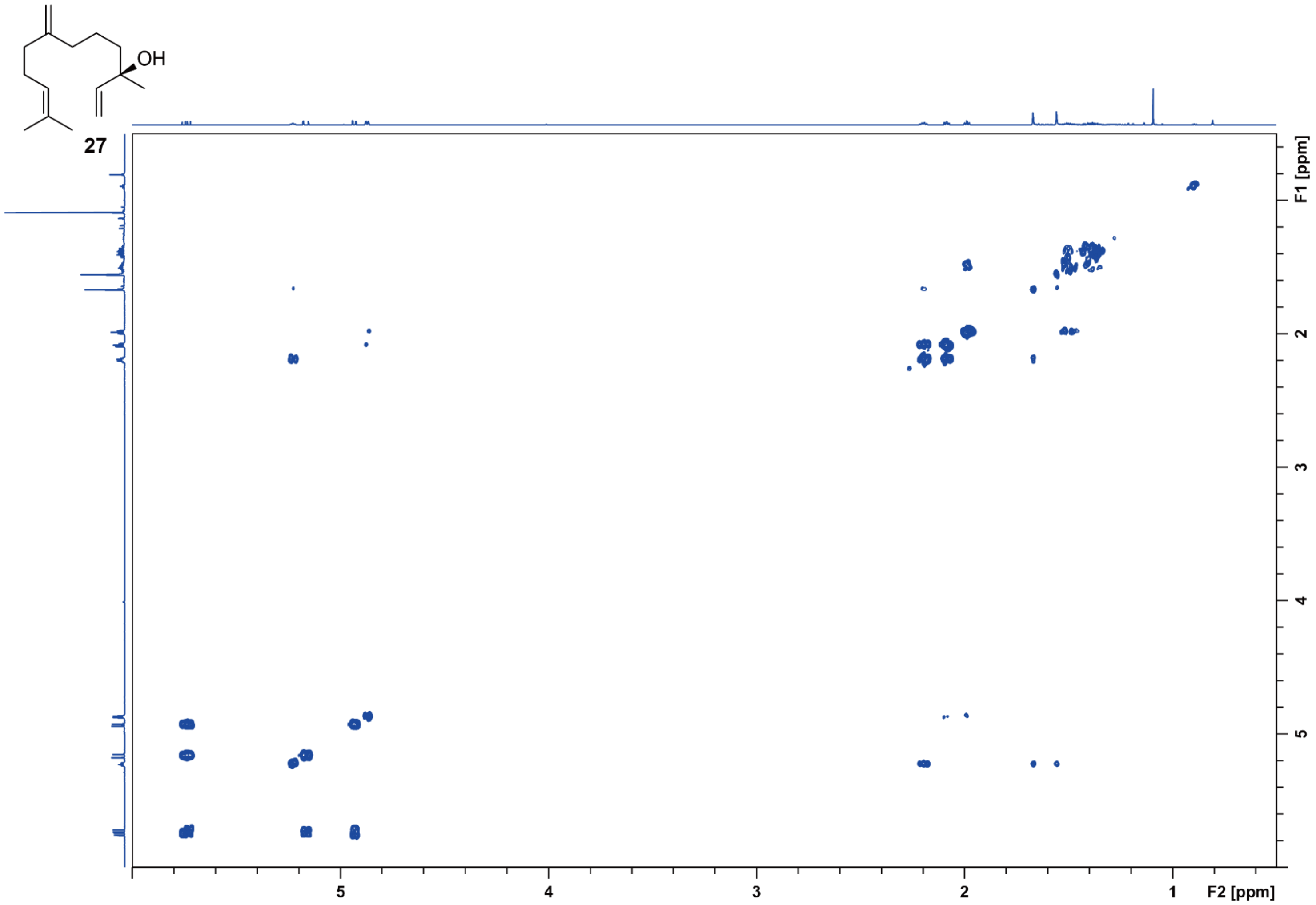

Figure S34. ^{1}H-^{1}H-COSY spectrum of compound **27** (C_6D_6, 700 MHz).

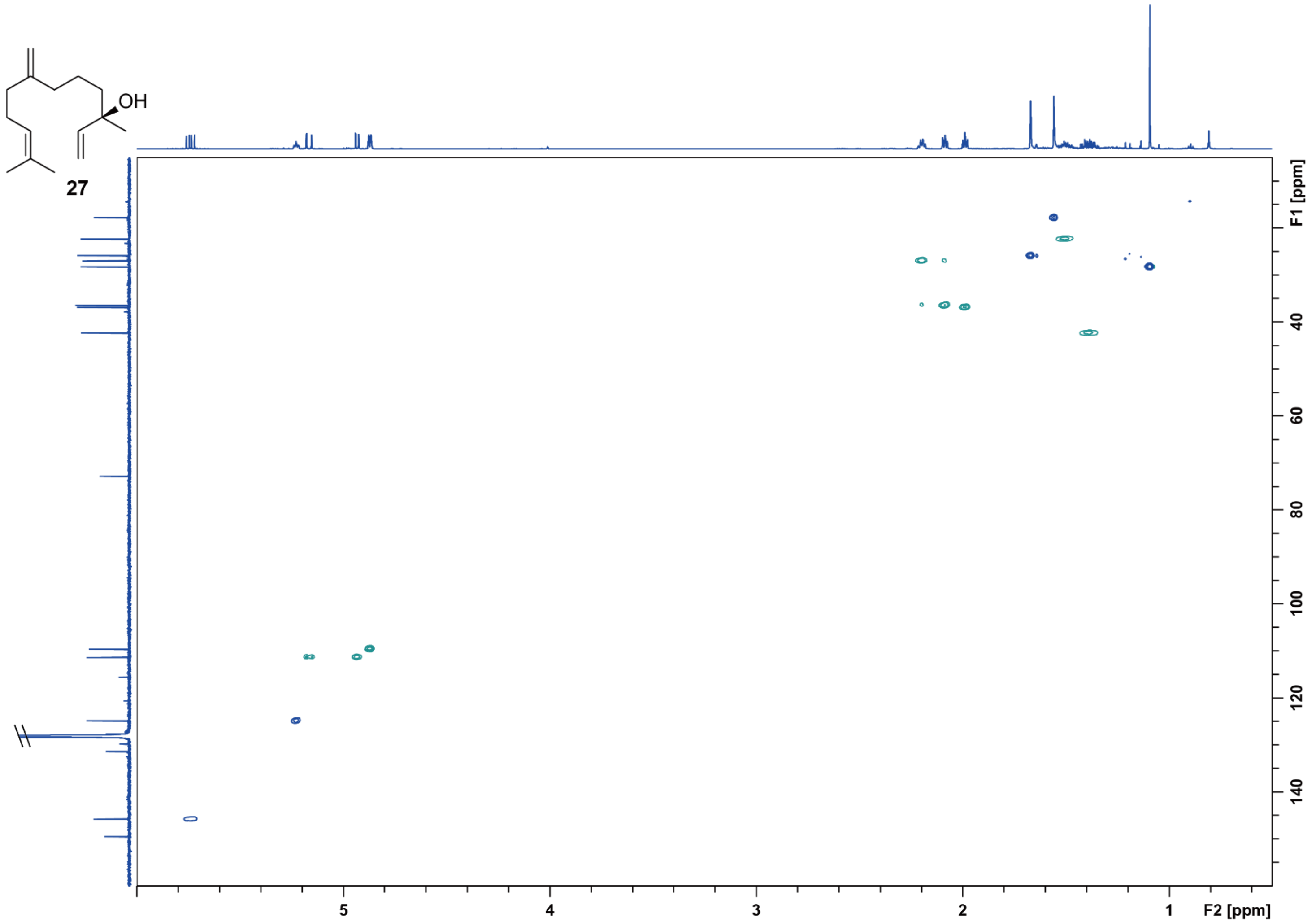

Figure S35. HSQC spectrum of compound **27** (C_6D_6).

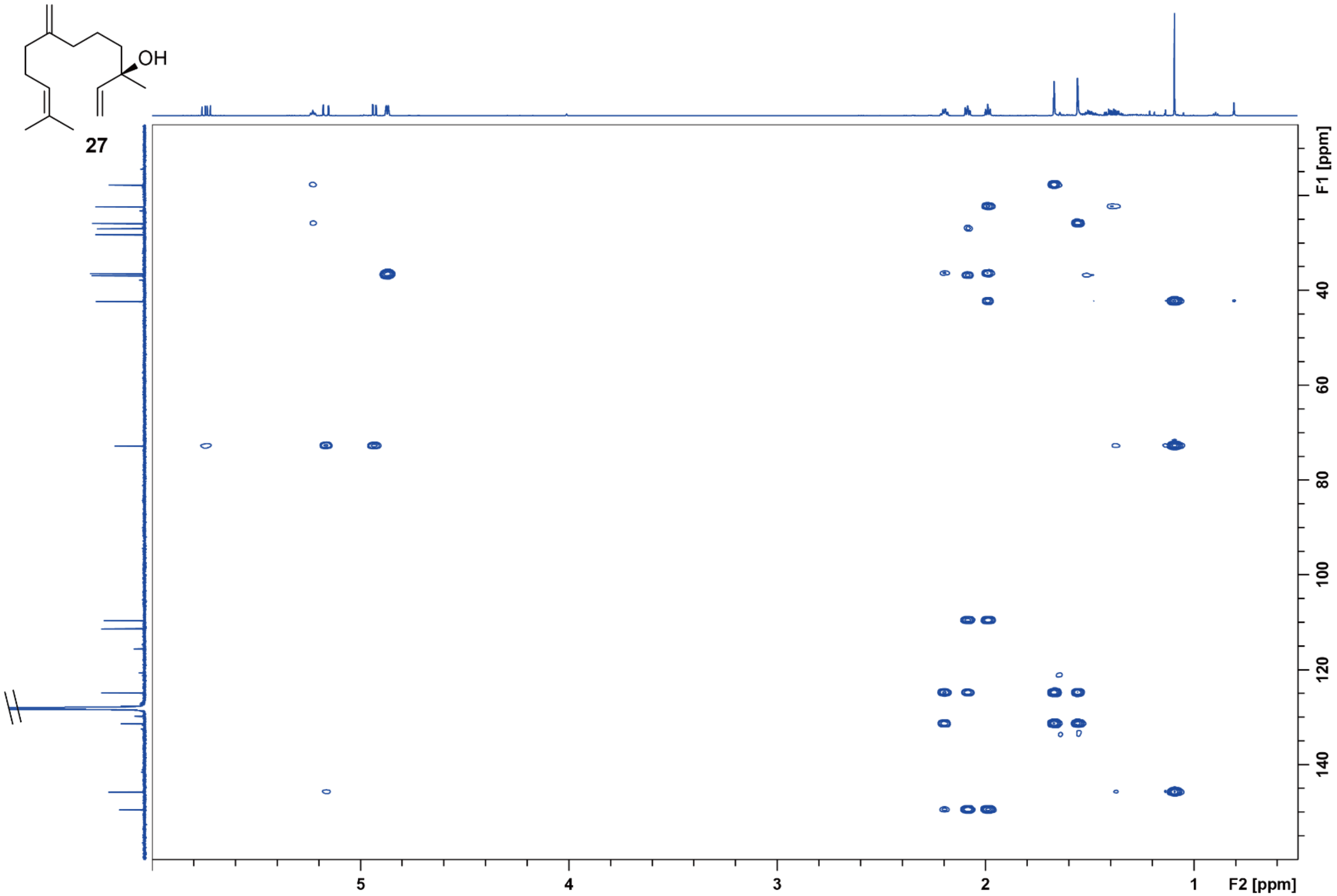

Figure S36. HMBC spectrum of compound **27** (C_6D_6).

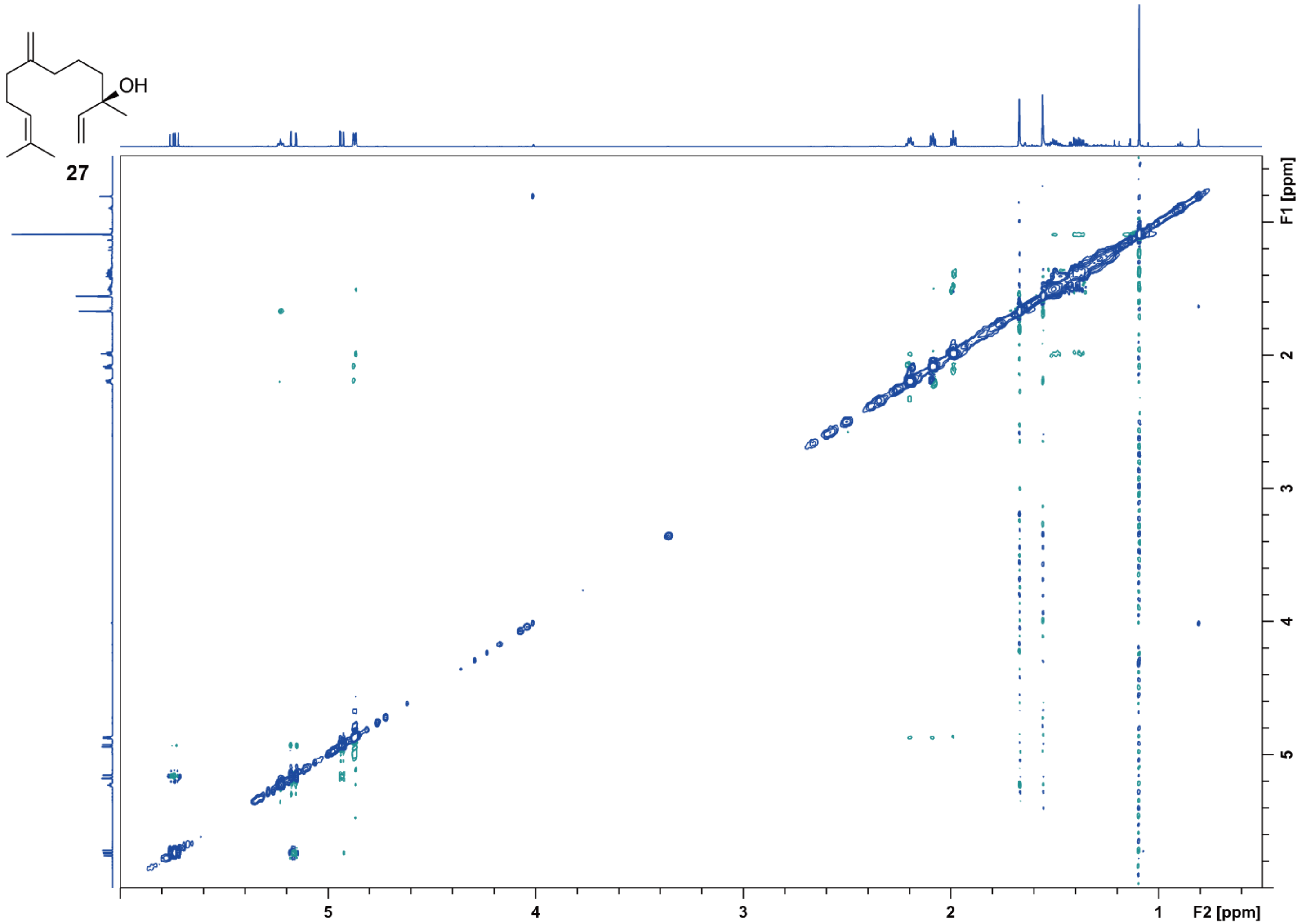

Figure S37. NOESY spectrum of compound **27** (C_6D_6, 700 MHz).

Ozonolysis of compound 24

Compound **24** (2.5 mg, 0.012 mmol) was dissolved in CH_2Cl_2 (5 mL) and MeOH (0.2 mL), and the mixture was cooled to –78 °C. O_3 was bubbled through the reaction solution and kept for 15 min. Then the reaction was quenched by the addition of SMe_2 (1 mL). The mixture was stirred at room temperature for 2 h and the solvent was removed under reduced pressure. The product **28** was purified via silica gel chromatography (Et_2O, 100%).

(4a*R*,7*R*,8a*S*)-7-Acetyl-4a-hydroxyoctahydronaphthalen-1(*2H*)-one (28): Yield, 1.6 mg, 0.008 mmol, 63%. TLC (Et_2O, 100%): R_f = 0.33. Optical rotation: $[\alpha]_D^{25}$ = +5.0 (*c* 0.16, CH_2Cl_2). EI-MS (70 eV): *m*/*z* (%) = 210 (2), 192 (36), 182 (23), 174 (7), 167 (10), 149 (41), 139 (90), 131 (25), 121 (12), 107 (10), 91 (20), 79 (22), 71 (11), 55 (34), 43 (100). GC (HP5-MS): *I* = 1820. HRMS (APCI): *m*/*z* = 211.1327 (calc. for $[C_{12}H_{18}O_3 + H]^+$: 211.1329). IR (diamond ATR): $\tilde{\nu}$ = 3463 (w), 2939 (m), 2872 (w), 2853 (w), 1702 (s), 1430 (w), 1360 (m), 1314 (w), 1296 (w), 1260 (w), 1237 (w), 1184 (w), 1174 (w), 1136 (w), 1104 (w), 1044 (w), 1029 (w), 1014 (w), 945 (w), 923 (w), 838 (w), 799 (w), 675 (w), 547 (w), 511 (w). NMR data cf. Table S5 and Figures S38 – S45.

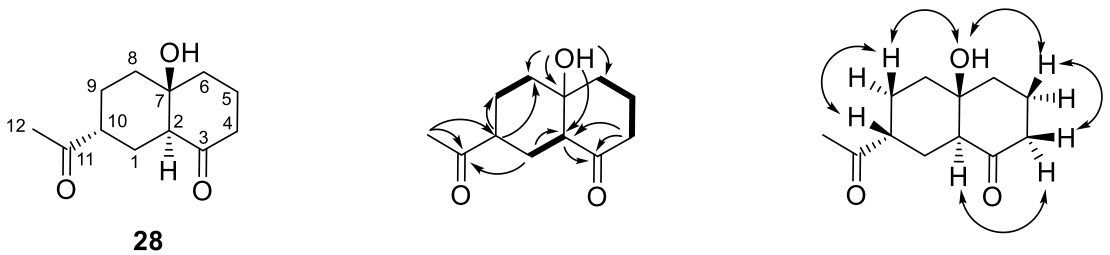

28

Figure S38. Structure elucidation of **28**. Bold: $^{1}H,^{1}H$-COSY, single headed arrows: key HMBC, and double headed arrows: key NOESY correlations.

Table S5. NMR data of **28** in C_6D_6 recorded at 298 K.

C[a]	type	^{13}C[b]	^{1}H[b]
1	CH_2	21.32	2.13 (m, 1H))
			1.87 (ddd, J = 14.7, 12.6, 5.8 Hz, 1H)
2	CH	52.03	2.35 (dd, J = 12.6, 3.7 Hz, 1H)
3	C_q	208.39	–
4	CH_2	40.99	2.23 (m, 1H)
			1.74 (m, 1H)
5	CH_2	21.6	1.79 (m, 1H)
			1.32 (m, 1H)
7	C_q	74.71	–
8	CH_2	35.91	1.48 (m, 1H)
			1.01 (m, 1H)
9	CH_2	21.33	1.56 (m, 1H)
			1.47 (m, 1H)
6	CH_2	38.38	1.11 (m, 2H)
10	CH	45.66	2.13 (m, 1H)
11	C_q	209.78	–
12	CH_3	27.51	1.63 (s, 3H)

[a] Carbon numbering as shown in Figure S38. [b] Chemical shifts δ in ppm, multiplicity: s = singlet, d = doublet, m = multiplet.

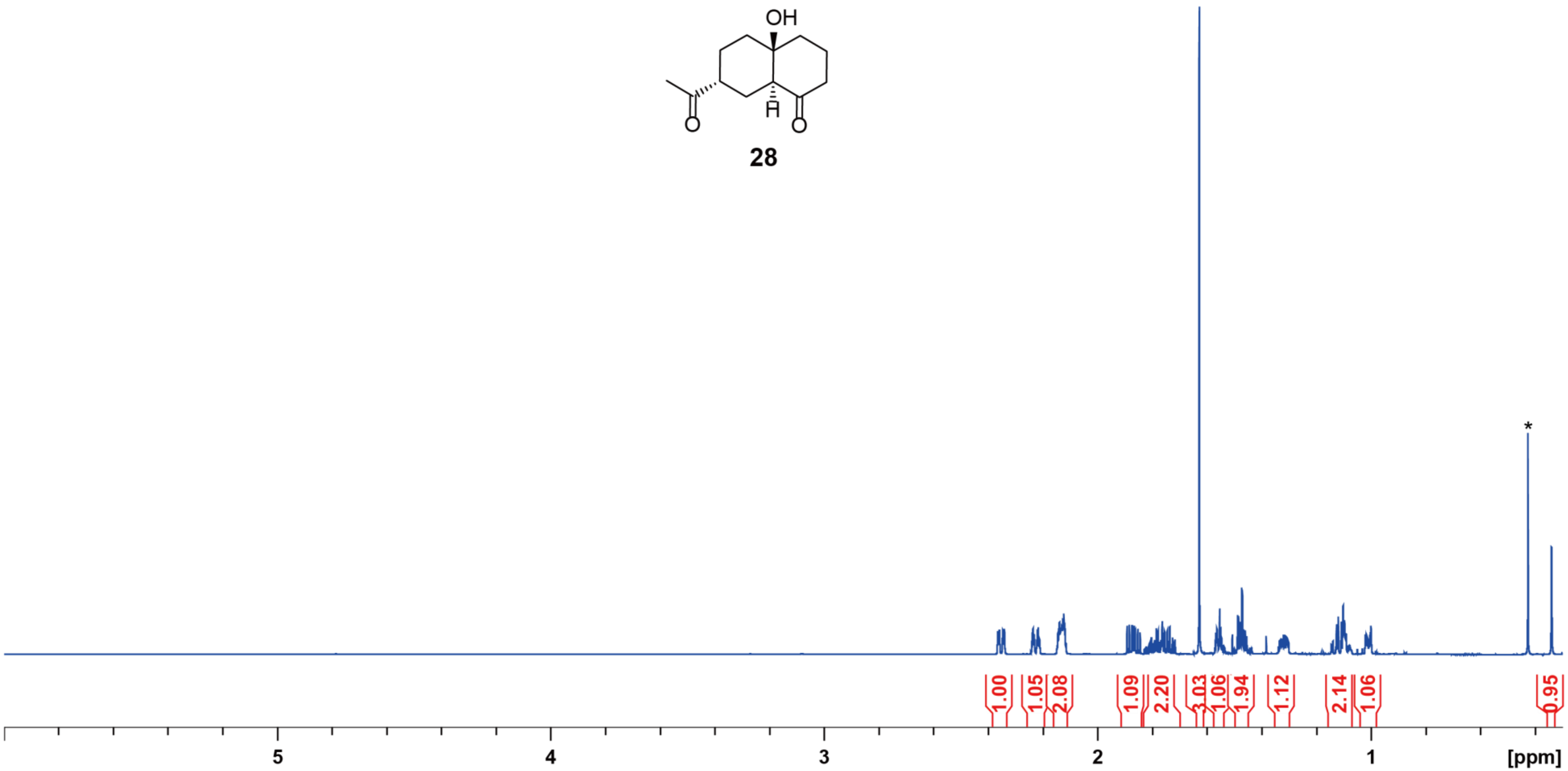

Figure S39. ^{1}H NMR spectrum of compound **28** (C_6D_6, 700 MHz). The asterisk indicates a peak from residual water.

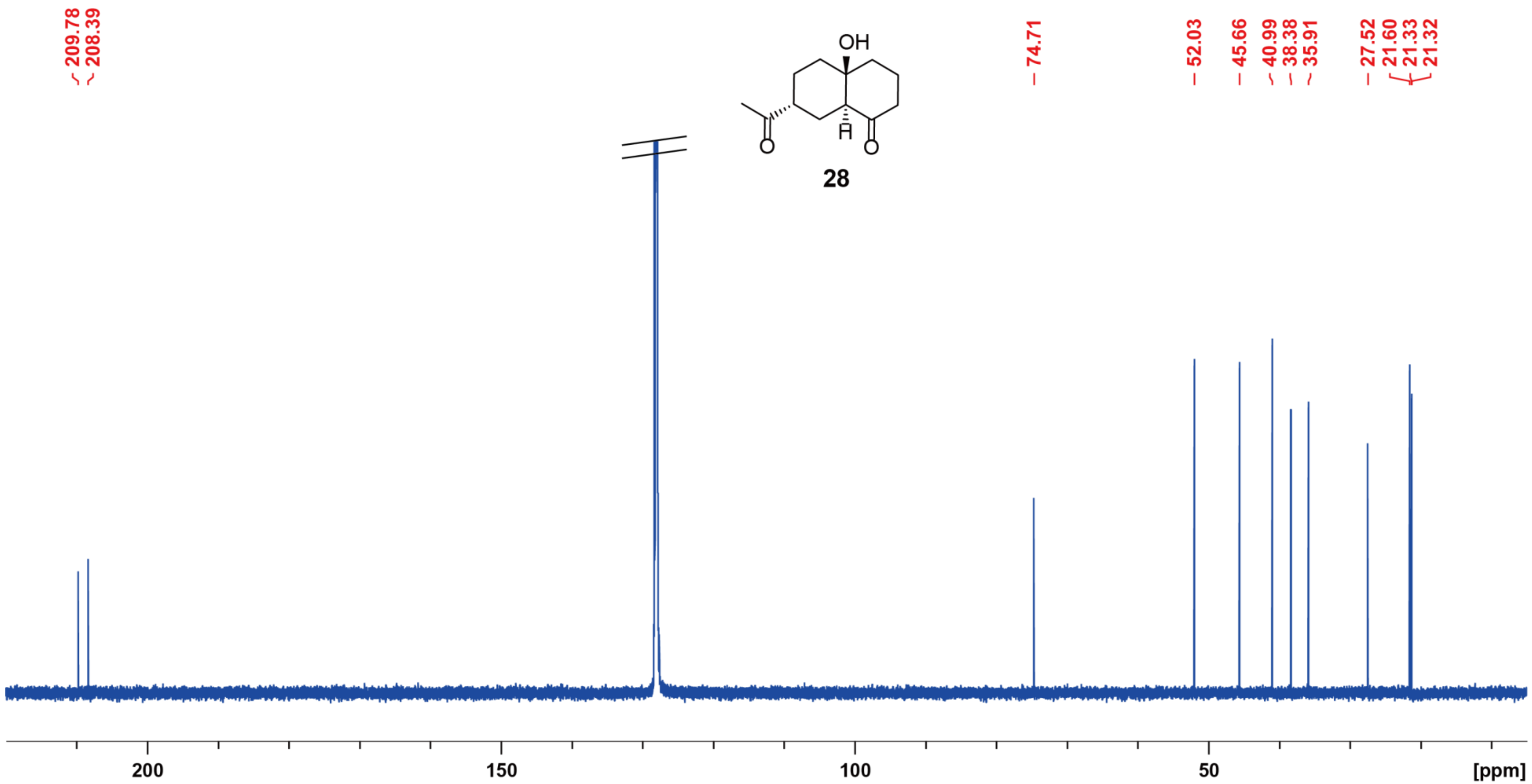

Figure S40. ^{13}C NMR spectrum of compound **28** (C_6D_6, 176 MHz).

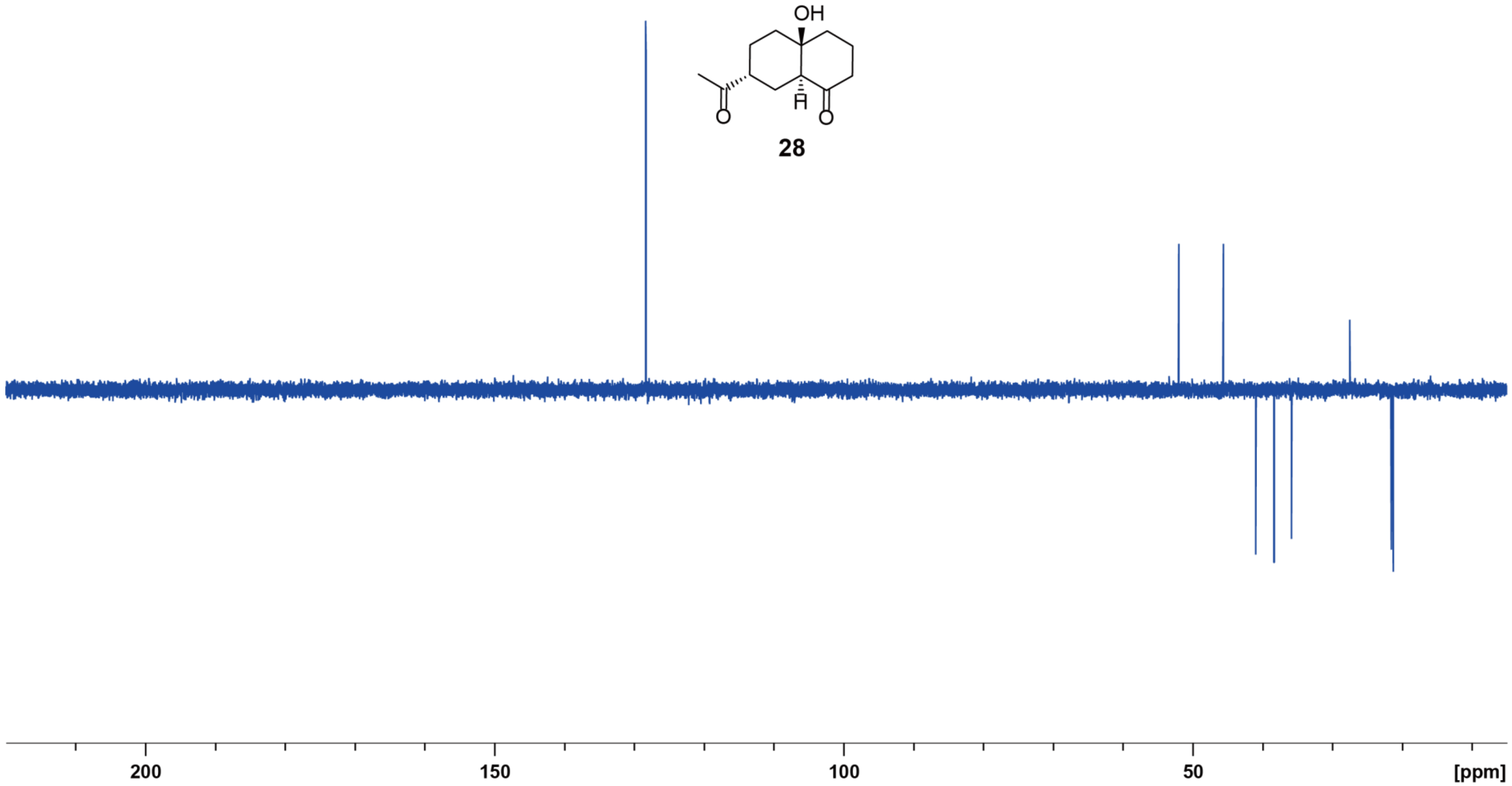

Figure S41. ^{13}C DEPT spectrum of compound **28** (C_6D_6, 176 MHz).

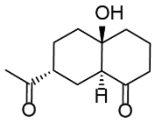

28

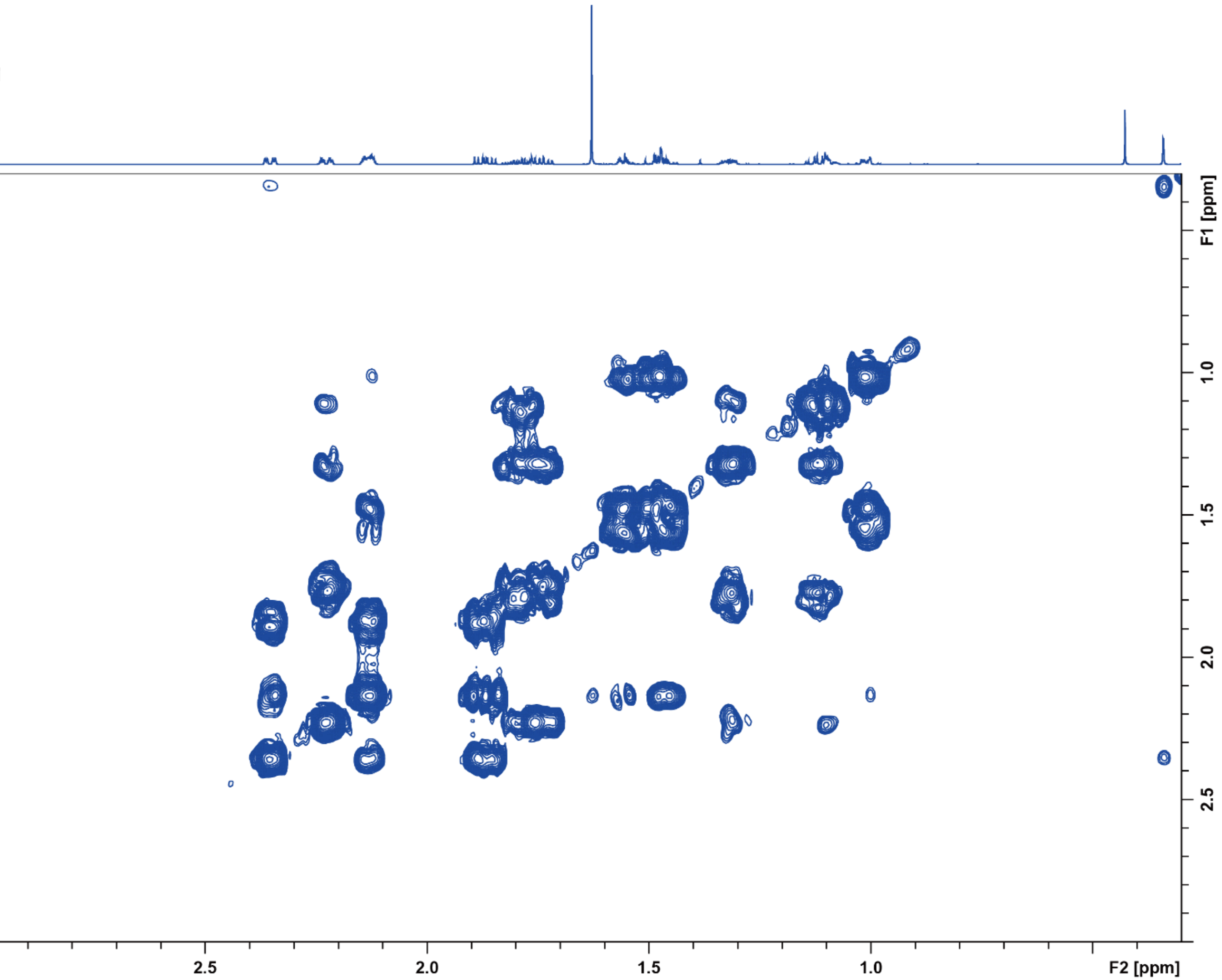

Figure S42. ^{1}H-^{1}H-COSY spectrum of compound **28** (C_6D_6, 700 MHz).

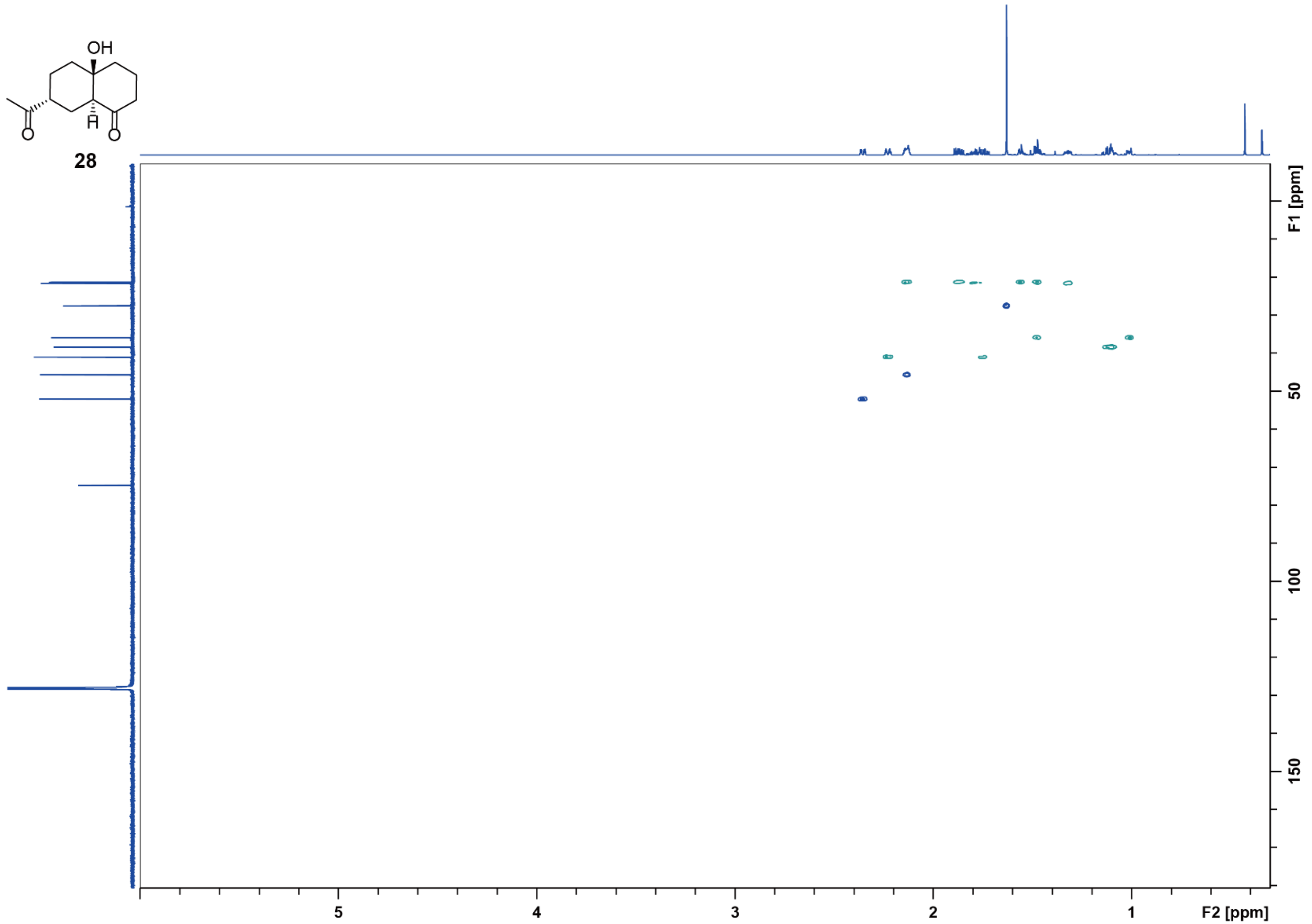

Figure S43. HSQC spectrum of compound **28** (C_6D_6).

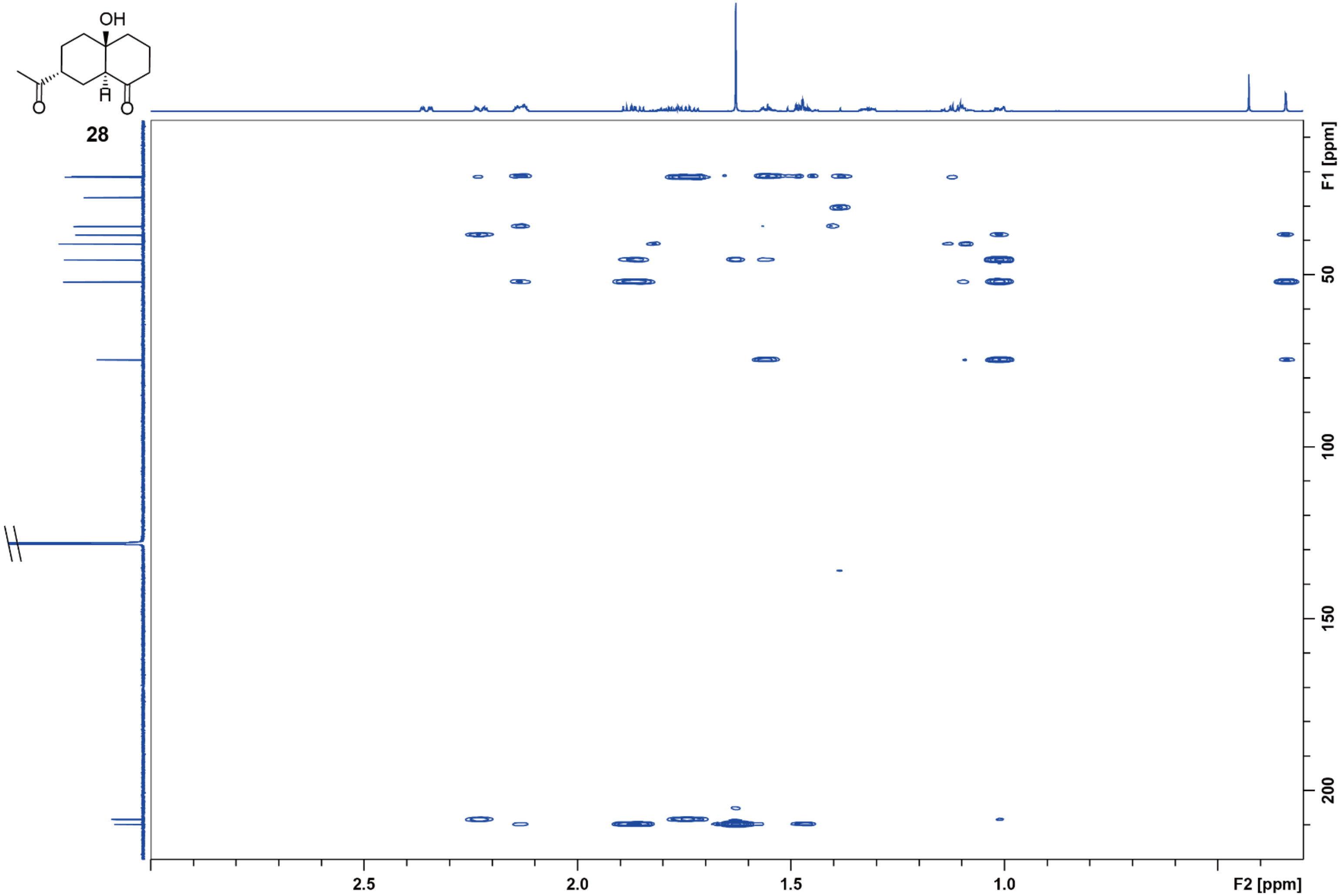

Figure S44. HMBC spectrum of compound **28** (C_6D_6).

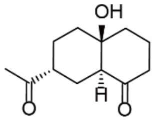

28

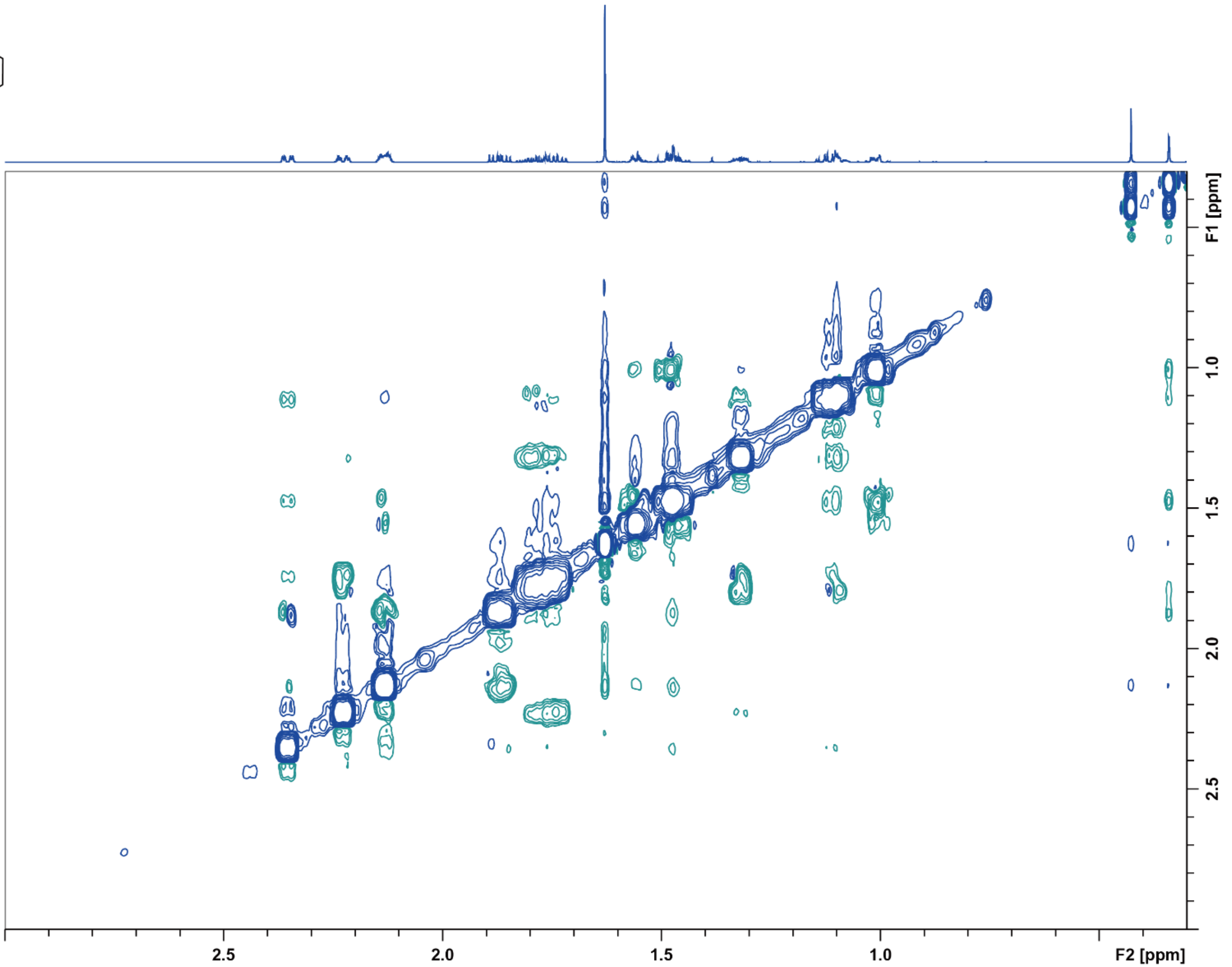

Figure S45. NOESY spectrum of compound **28** (C_6D_6, 700 MHz).

Incubation reactions with BbS and substrates 10 – 13

BbS was purified following a published procedure.[3] The obtained protein concentration was ca. 2 mg/mL. The small scale and large scale reactions were performed by the same procedure as reported above for enzyme reactions with HcS.

(*Z*)-1-Methyl-4-(6-methylheptan-2-ylidene)cyclohex-1-ene (29). Yield: 1.2 mg, 5.8 μmol, 3%. TLC (pentane, 100%): R_f = 0.58. MS spectrum cf. Figure S47A. GC (HP5-MS): *I* = 1493. HRMS (APCI): *m*/*z* = 207.2109 (calc. for $[C_{15}H_{26} + H]^+$: 207.2107). IR (diamond ATR): $\tilde{\nu}$ = 2955 (s), 2926 (w), 2868 (m), 2856 (m), 1980 (w), 1739 (w), 1466 (m), 1448 (m), 1377 (m), 1366 (m), 1205 (w), 1169 (w), 1124 (w), 1057 (w), 931 (w), 915 (w), 891 (w), 794 (w), 766 (w), 475 (w), 421(w) cm^{-1}. NMR data cf. Table S6 and Figures S48 – S55.

(*Z*)-1-Methyl-4-(6-methylhept-6-en-2-ylidene)cyclohex-1-ene (30). Yield: 5.6 mg, 27.4 μmol, 12%. TLC (pentane, 100%): R_f = 0.50. MS spectrum cf. Figure S47B. GC (HP5-MS): *I* = 1524. HRMS (APCI): *m*/*z* = 205.1961 (calc. for $[C_{15}H_{24} + H]^+$: 205.1951). IR (diamond ATR): $\tilde{\nu}$ = 3073 (w), 2965 (m), 2928 (m), 2910 (m), 2855 (m), 2811 (w), 1649 (m), 1445 (m), 1374 (m), 1345 (w), 1227 (w), 1206 (w), 1156 (w), 1093 (w), 1051 (w), 930 (w), 914 (w), 885 (s), 816 (w), 795 (w), 766 (w), 543 (m), 466 (w), 431 (w) cm^{-1}. NMR data cf. Table S7 and Figures S56 – S63.

(*Z*)-6-(4-Methylcyclohex-3-en-1-ylidene)heptan-2-one (31). Yield: 0.4 mg, 1.9 μmol, 1%, TLC (pentane/Et_2O, 3:1): R_f = 0.56. MS spectrum cf. Figure S47C. GC (HP5-MS): *I* = 1642. HRMS (APCI): *m*/*z* = 207.1755 (calc. for $[C_{14}H_{22}O + H]^+$: 207.1743). IR (diamond ATR): $\tilde{\nu}$ = 2961 (m), 2924 (m), 2912 (m), 2855 (m), 2278 (w), 1737 (m), 1716 (s), 1566 (w), 1437 (m), 1365 (m), 1345 (m), 1228 (w), 1217 (w), 1206 (w), 1159 (w), 816 (w), 796 (w), 544 (s) cm^{-1}. NMR data cf. Table S8 and Figures S64 – S71.

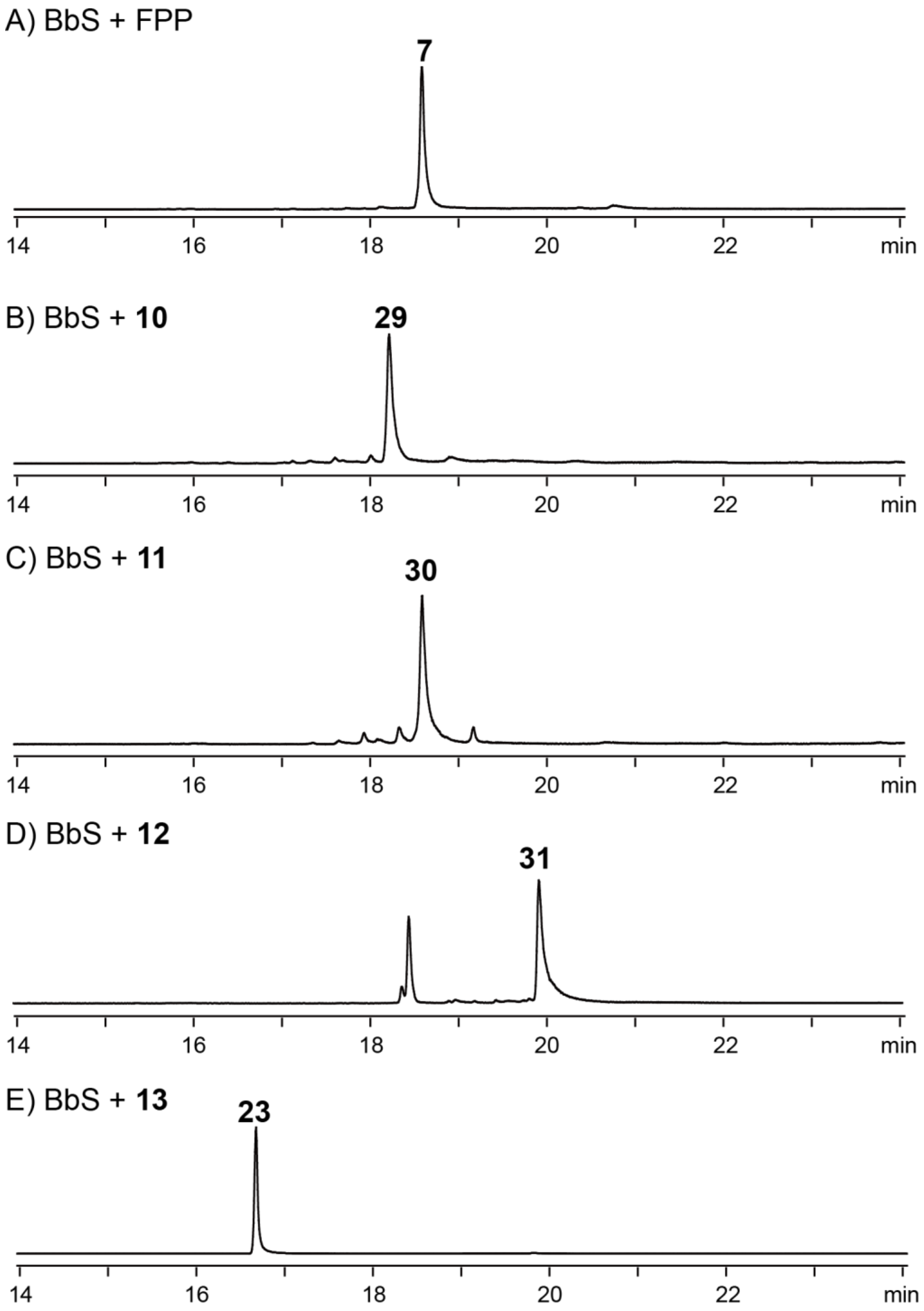

Figure S46. Total ion chromatograms of the extracts from the incubation reactions with BbS and A) FPP, B) substrates **10**, C) **11**, D) **12** and E) **13**.

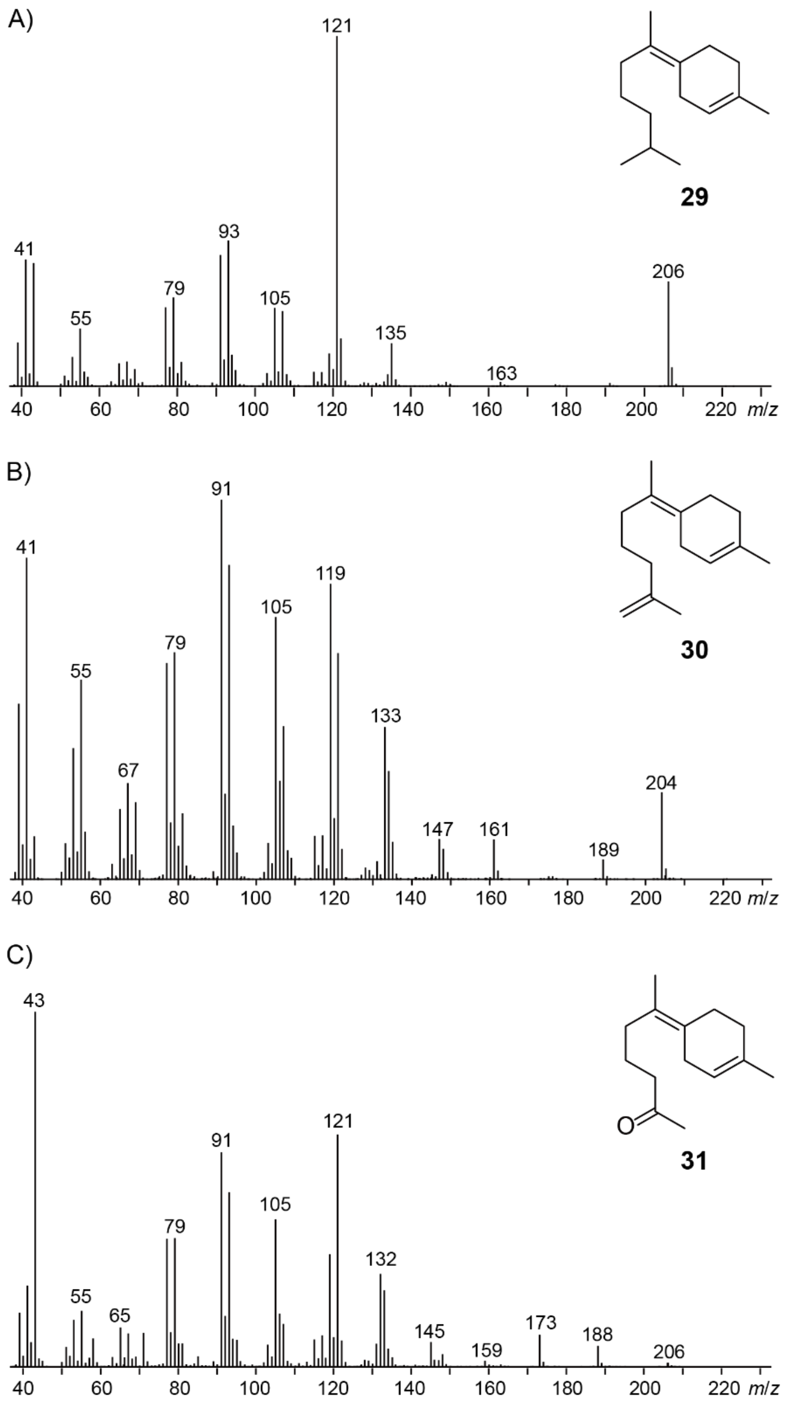

Figure S47. Mass spectra of compounds **29**, **30** and **31**.

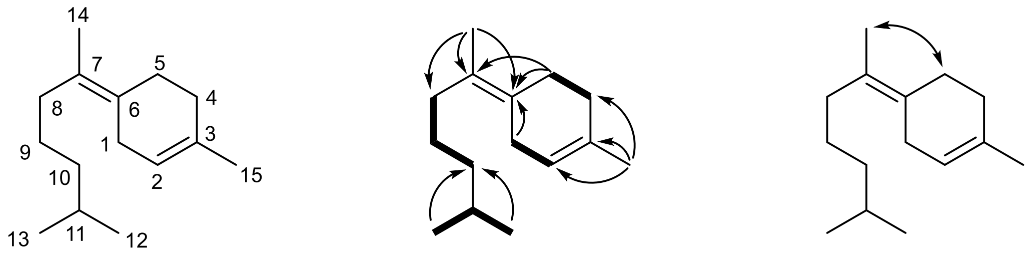

29

Figure S48. Structure elucidation of **29**. Bold: $^1H,^1H$-COSY, single headed arrows: key HMBC, and double headed arrows: key NOESY correlations.

Table S6. NMR data of **29** in C_6D_6 recorded at 298 K.

C[a]	^{13}C[b]	^1H[b]
1	29.82	2.84 (br s, 2H)
2	121.37	5.42 (m, 1H)
3	134.09	–
4	31.88	1.96 (m, 2H)
5	27.37	2.34 (t, *J* = 6.4 Hz, 2H)
6	128.07	–
7	126.19	–
8	34.88	2.05 (m, 2H)
9	26.43	1.40 (m, 2H)
10	39.34	1.17 (m, 2H)
11	28.29	1.49 (dt, *J* = 13.3, 6.6 Hz, 1H)
12, 13	22.84	0.88 (d, *J* = 6.6 Hz, 6H)
14	17.97	1.71 (s, 3H)
15	23.60	1.64 (s, 3H)

[a] Carbon numbering as shown in Figure S48. [b] Chemical shifts δ in ppm, multiplicity: s = singlet, d = doublet, t = triplet, m = multiplet, br = broad.

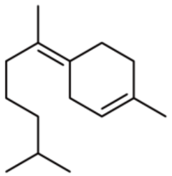

29

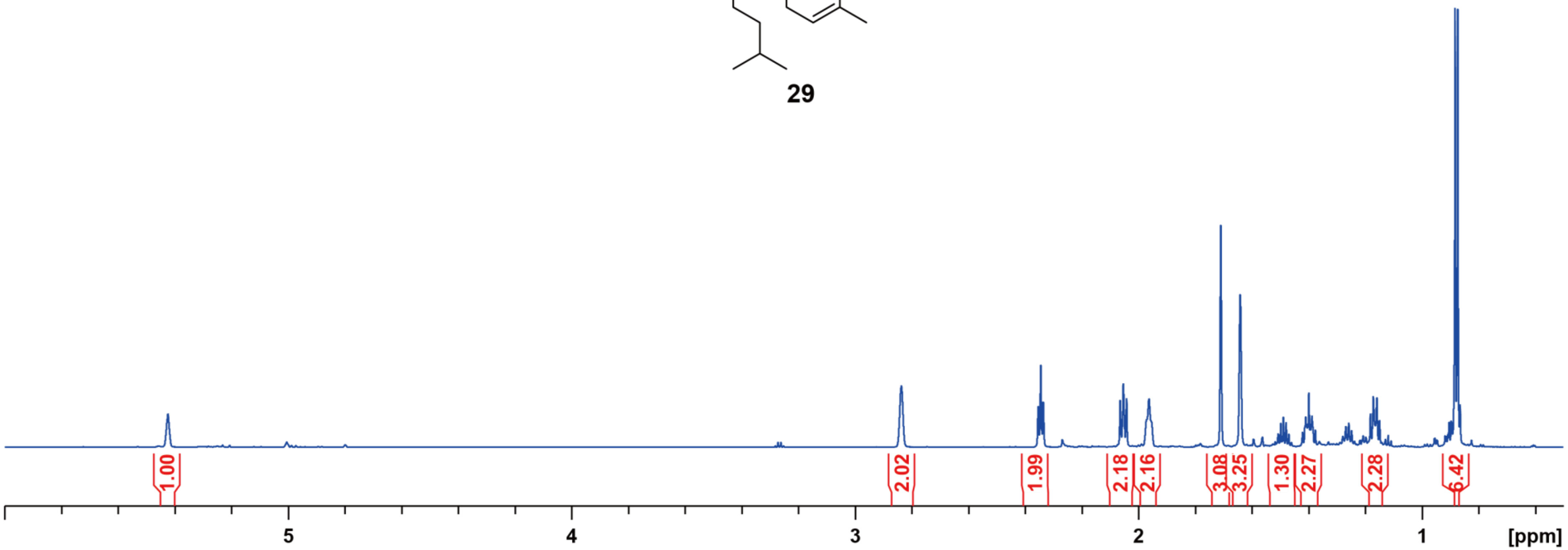

Figure S49. ^{1}H NMR spectrum of compound **29** (C_6D_6, 700 MHz).

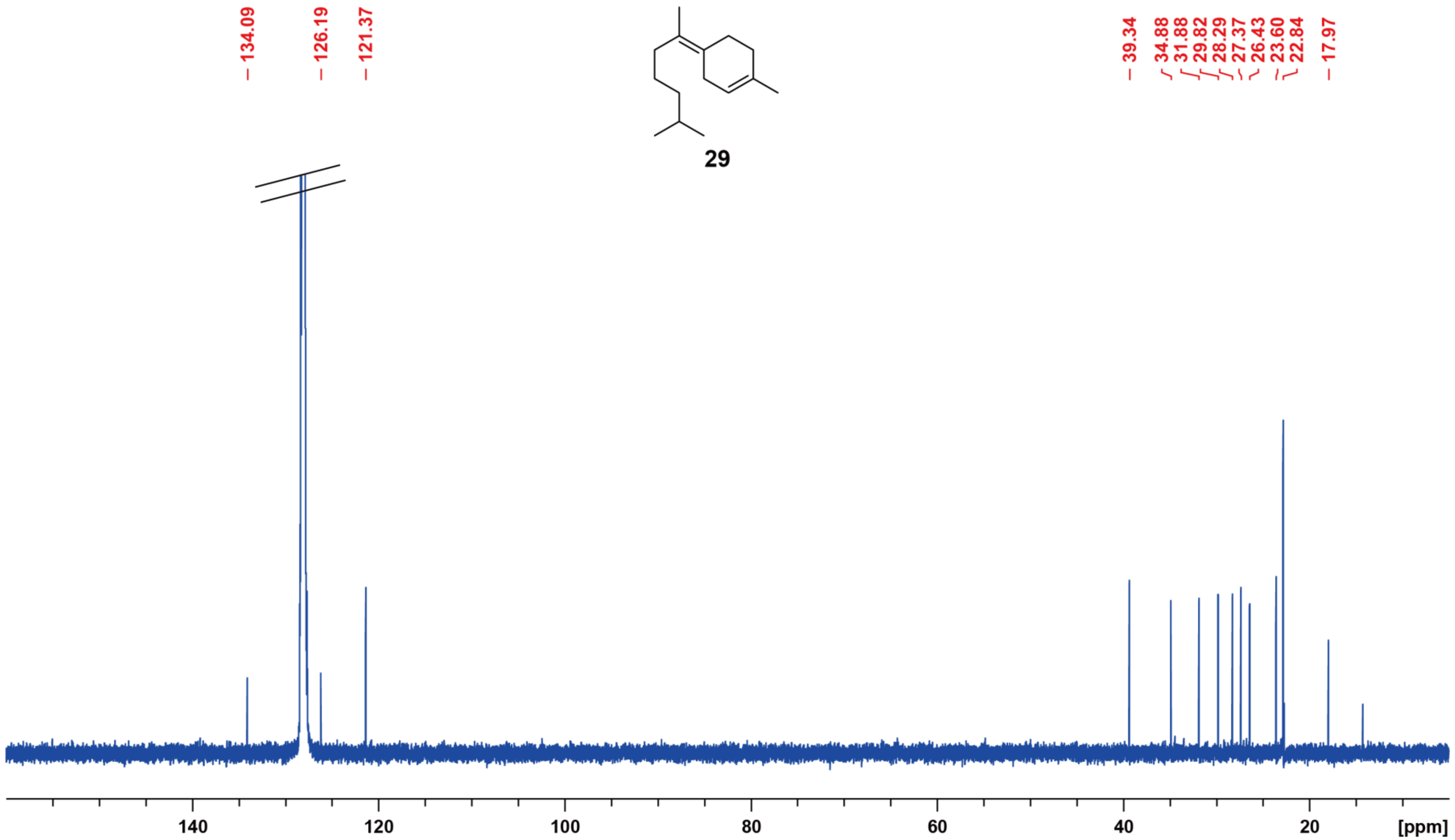

Figure S50. ^{13}C NMR spectrum of compound **29** (C_6D_6, 176 MHz). One peak was buried in the solvent signal of C_6D_6, which was assigned from HMBC spectrum Figure S54.

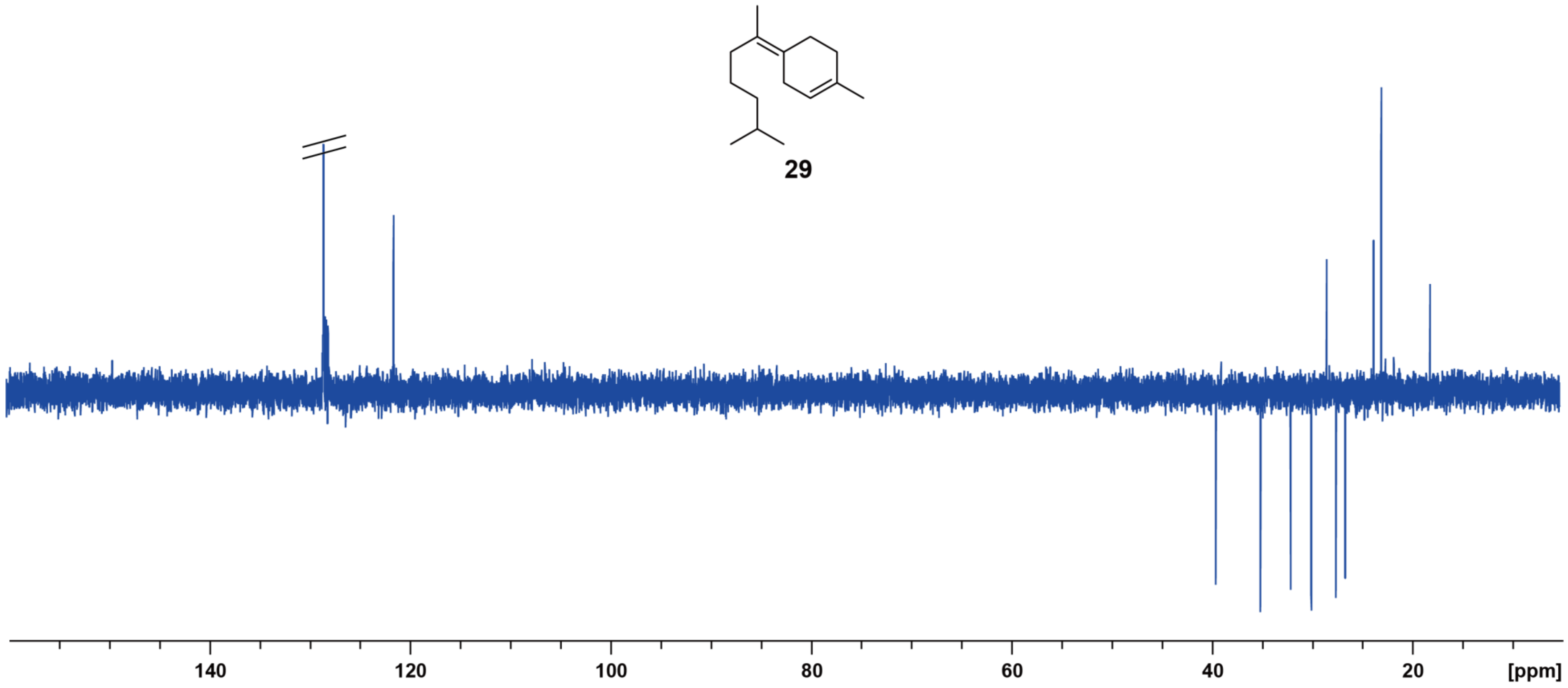

Figure S51. ^{13}C DEPT spectrum of compound **29** (C_6D_6, 176 MHz).

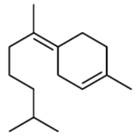

29

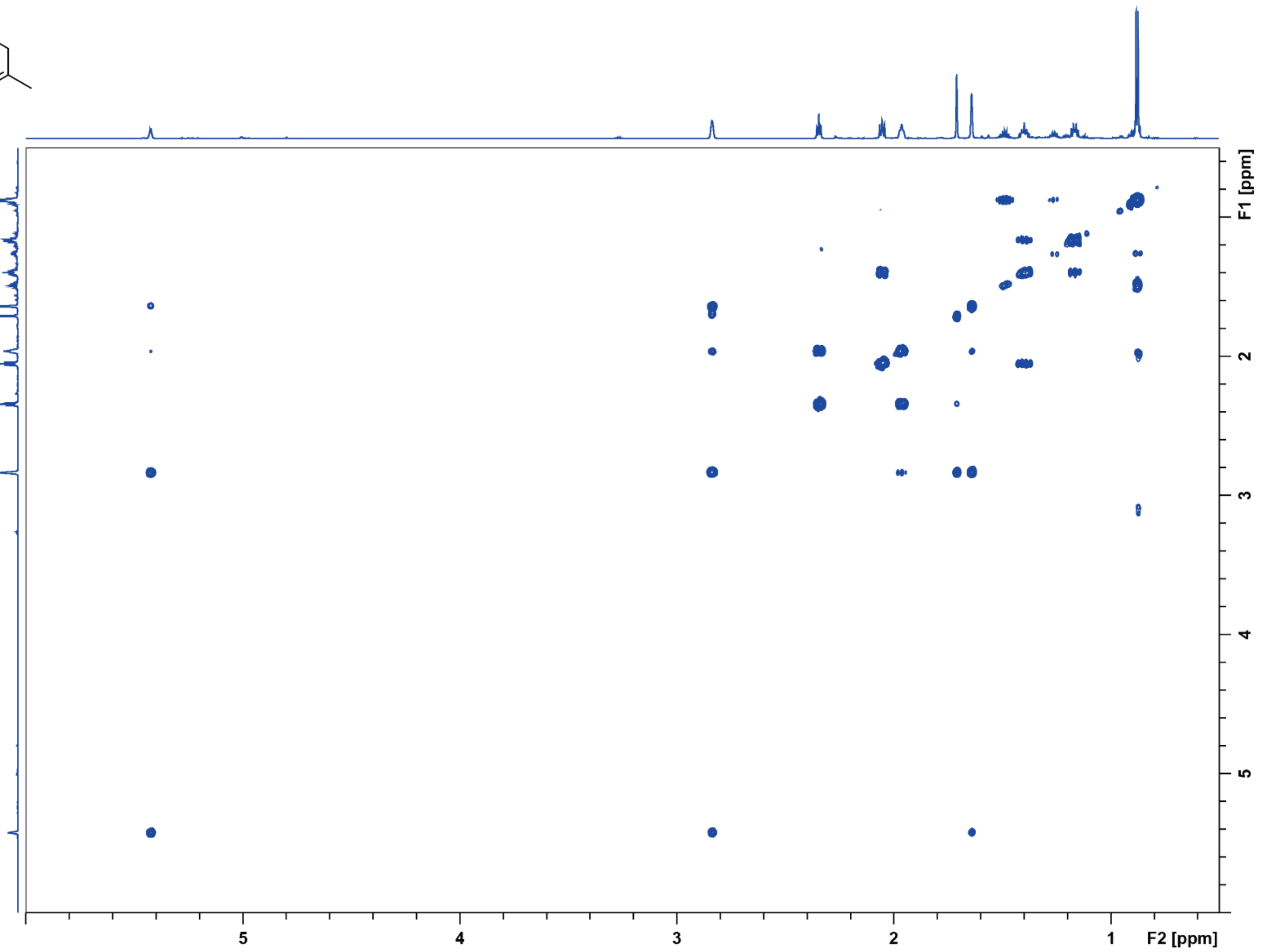

Figure S52. ^{1}H-^{1}H-COSY spectrum of compound **29** (C_6D_6, 700 MHz).

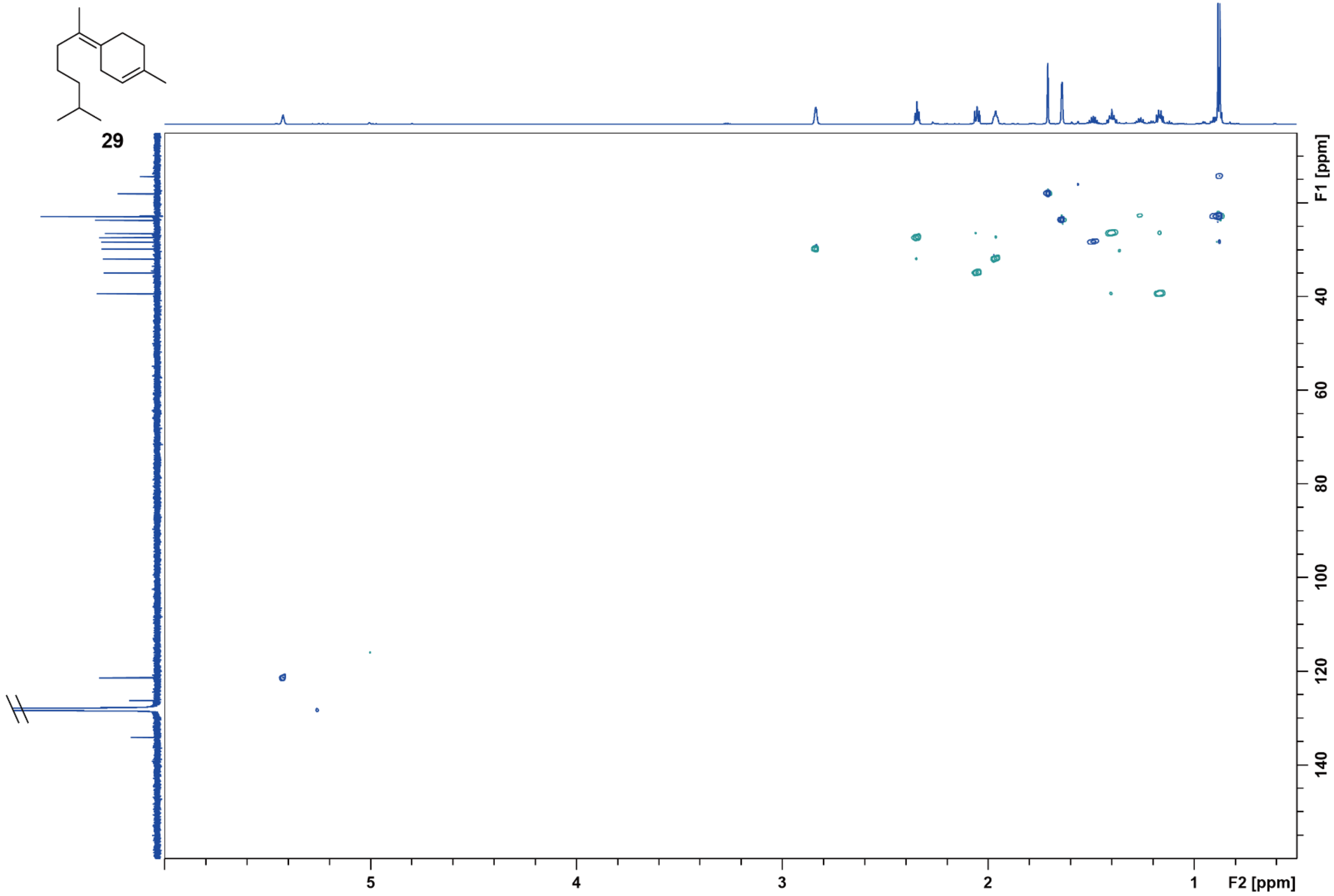

Figure S53. HSQC spectrum of compound **29** (C_6D_6).

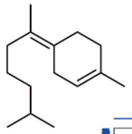
29

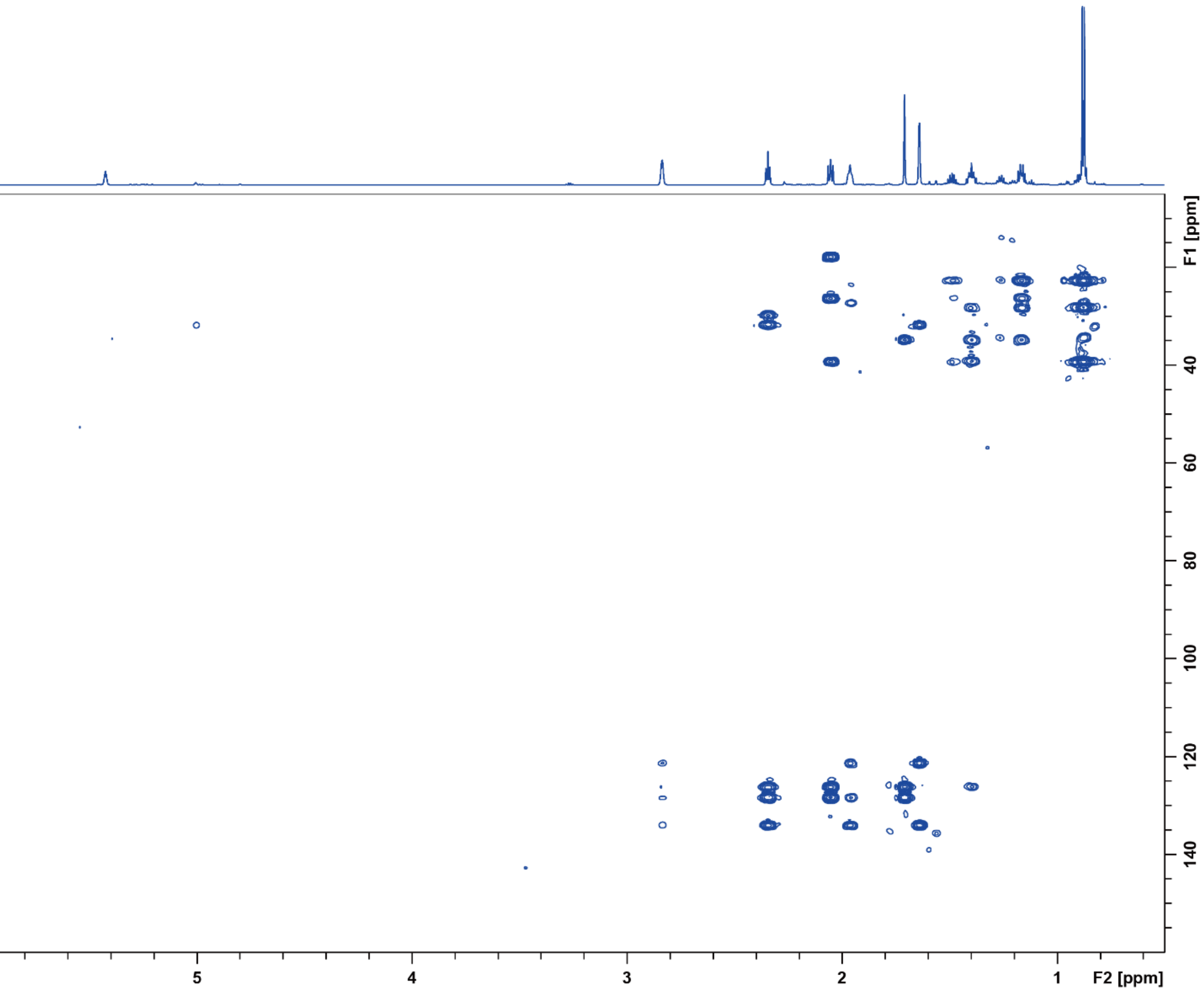

Figure S54. HMBC spectrum of compound **29** (C_6D_6).

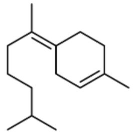

29

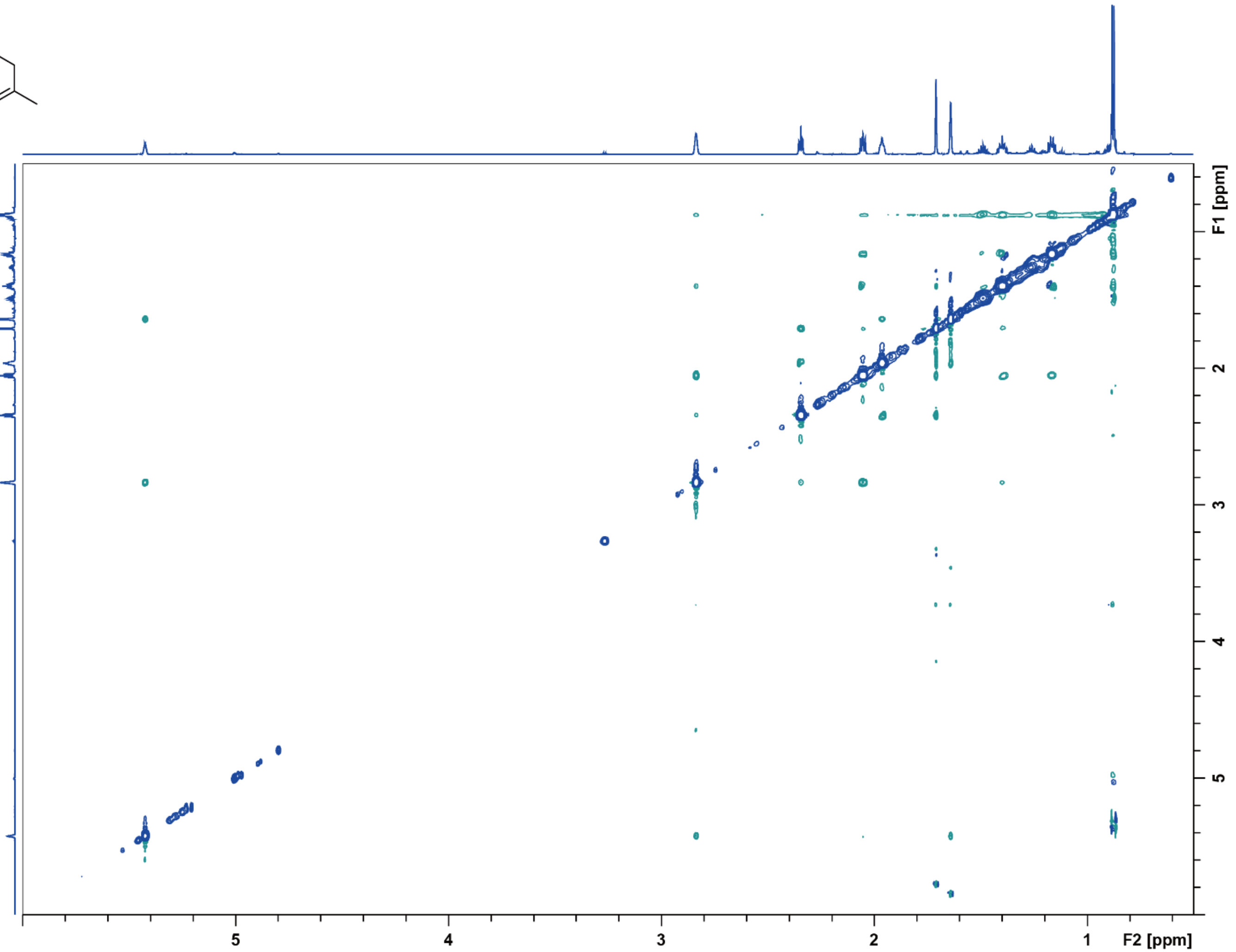

Figure S55. NOESY spectrum of compound **29** (C_6D_6).

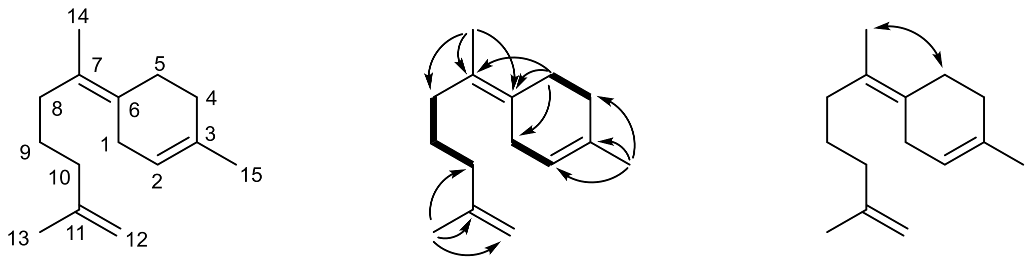

30

Figure S56. Structure elucidation of **30**. Bold: ^{1}H,^{1}H-COSY, single headed arrows: key HMBC, and double headed arrows: key NOESY correlations.

Table S7. NMR data of **30** in C_6D_6 recorded at 298 K.

C[a]	type	^{13}C[b]	^{1}H[b]
1	CH_2	29.80	2.81 (br s, 2H)
2	CH	121.34	5.41 (m, 1H)
3	C_q	134.07	–
4	CH_2	31.86	1.95 (m, 2H)
5	CH_2	27.38	2.33 (t, J = 6.4 Hz, 2H)
6	C_q	128.78	–
7	C_q	125.83	–
8	CH_2	34.25	2.04 (m, 2H)
9	CH_2	26.50	1.53 (m, 2H)
10	CH_2	38.03	1.96 (m, 2H)
11	C_q	145.84	–
12	CH_2	110.29	4.81 (m, 2H)
13	CH_3	22.54	1.65 (t, J = 1.1 Hz, 3H)
14	CH_3	17.88	1.68 (s, 3H)
15	CH_3	23.59	1.64 (td, J = 2.2, 1.2 Hz, 3H)

[a] Carbon numbering as shown in Figure S56. [b] Chemical shifts δ in ppm, multiplicity: s = singlet, d = doublet, t = triplet, m = multiplet, br = broad.

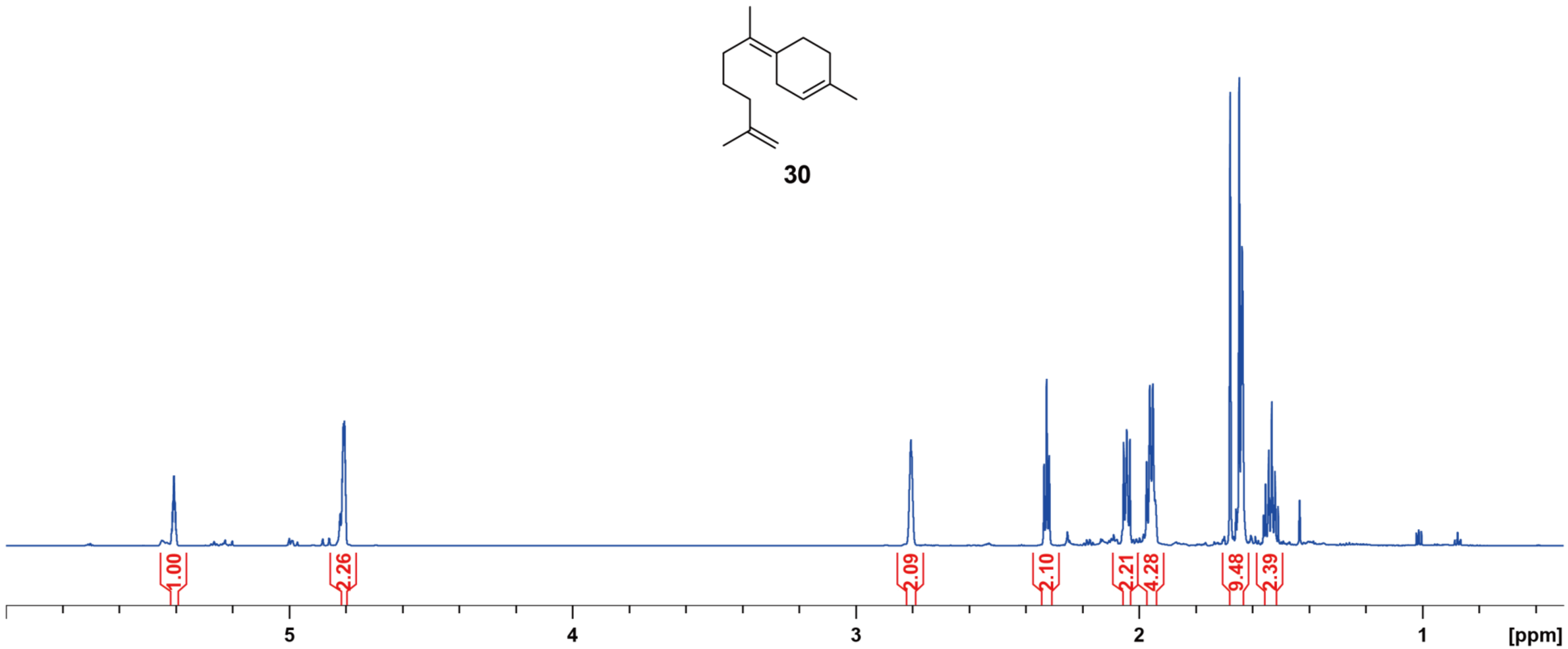

Figure S57. ^{1}H NMR spectrum of compound **30** (C_6D_6, 700 MHz).

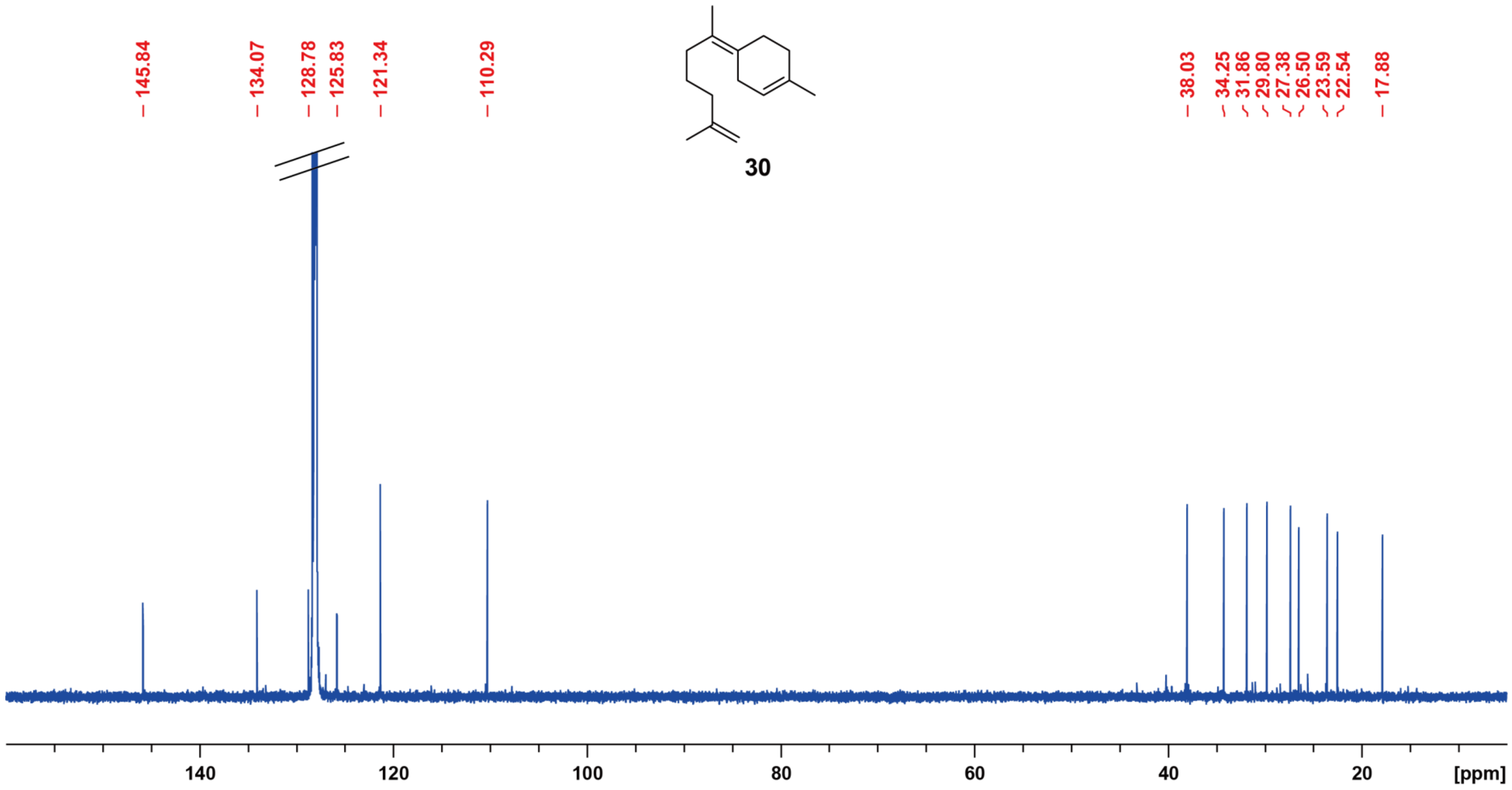

Figure S58. ^{13}C NMR spectrum of compound **30** (C_6D_6, 176 MHz).

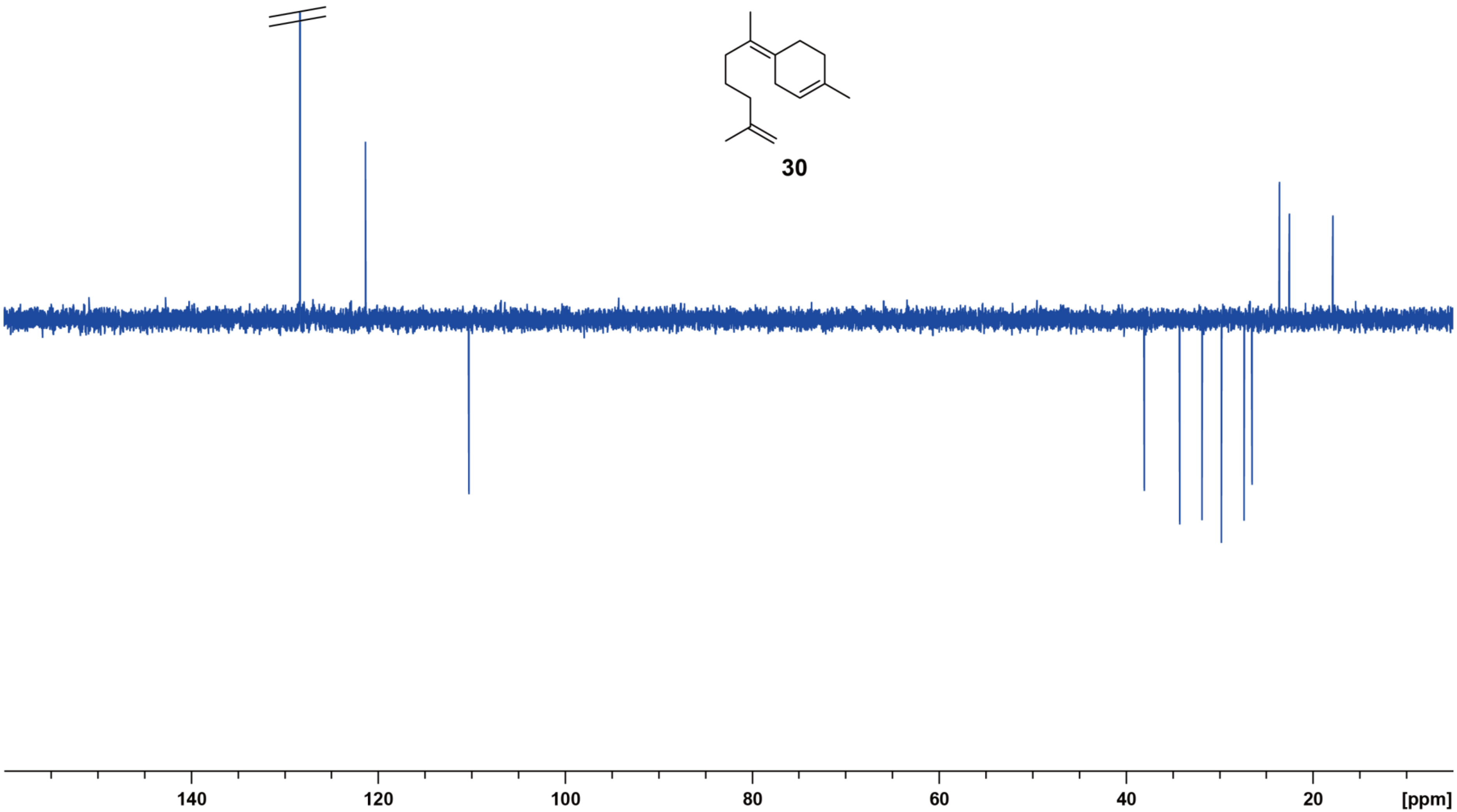

Figure S59. ^{13}C DEPT spectrum of compound **30** (C_6D_6, 176 MHz).

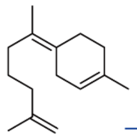

30

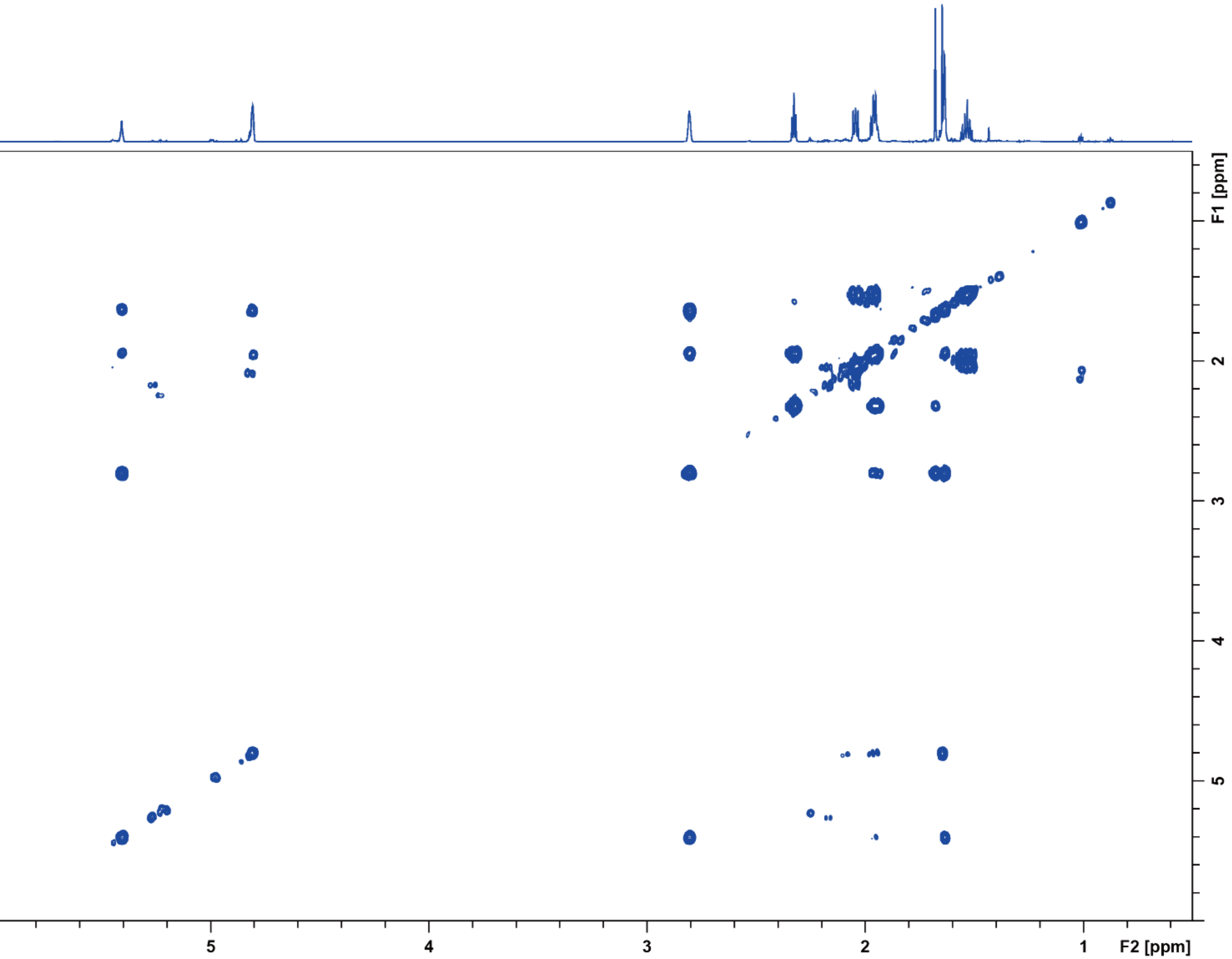

Figure S60. ^{1}H-^{1}H-COSY spectrum of compound **30** (C_6D_6, 700 MHz).

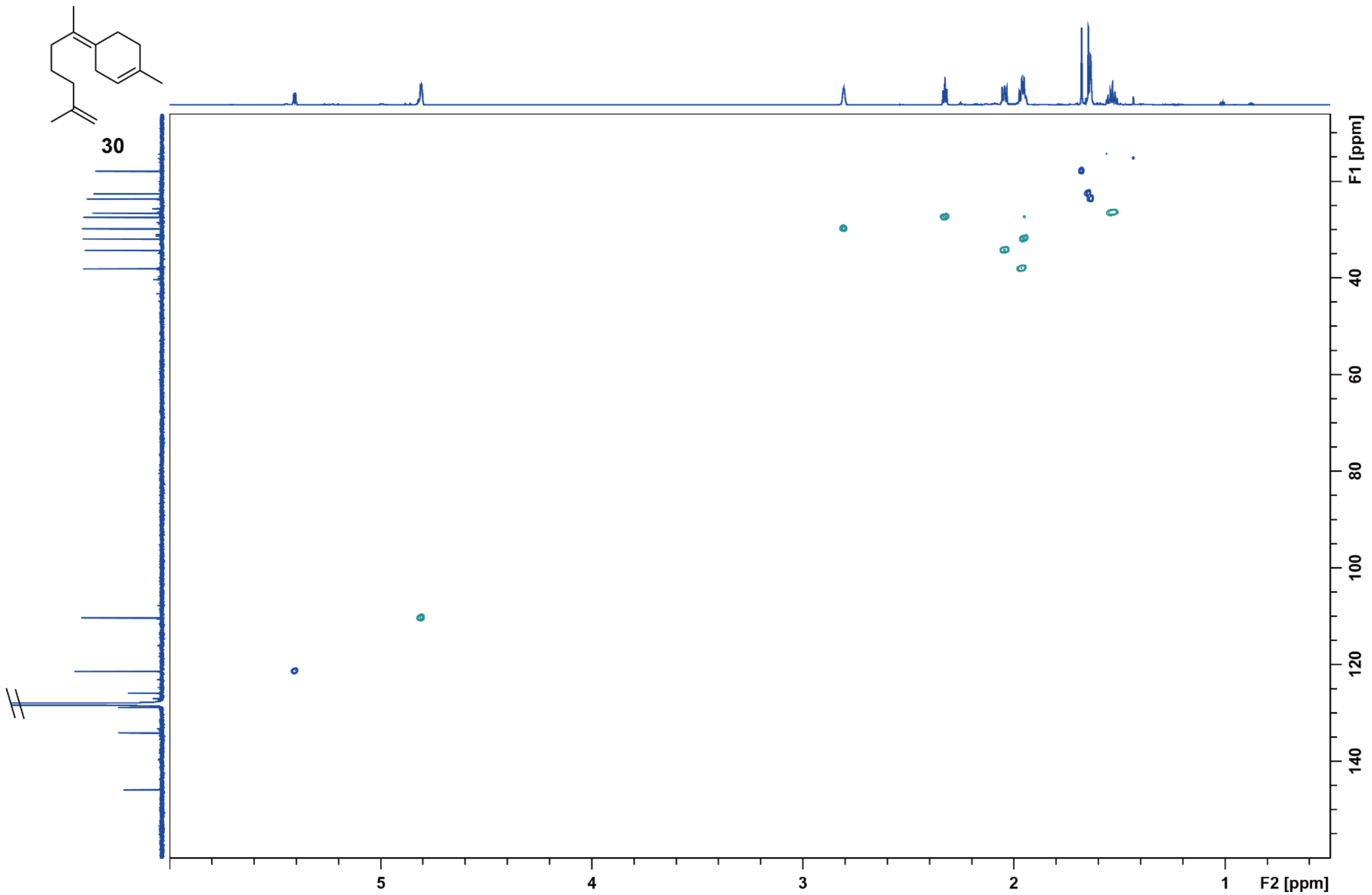

Figure S61. HSQC spectrum of compound **30** (C_6D_6).

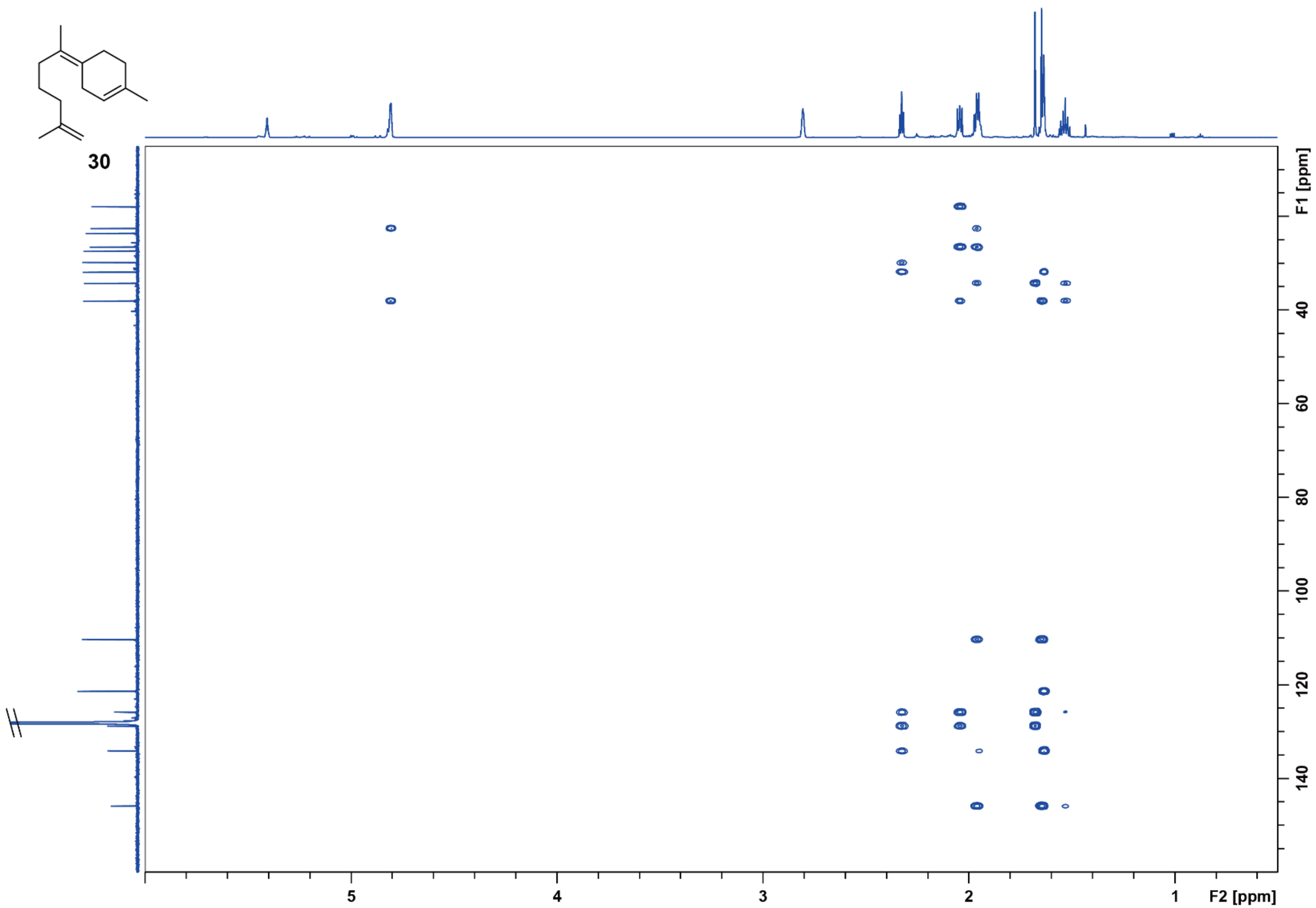

Figure S62. HMBC spectrum of compound **30** (C_6D_6).

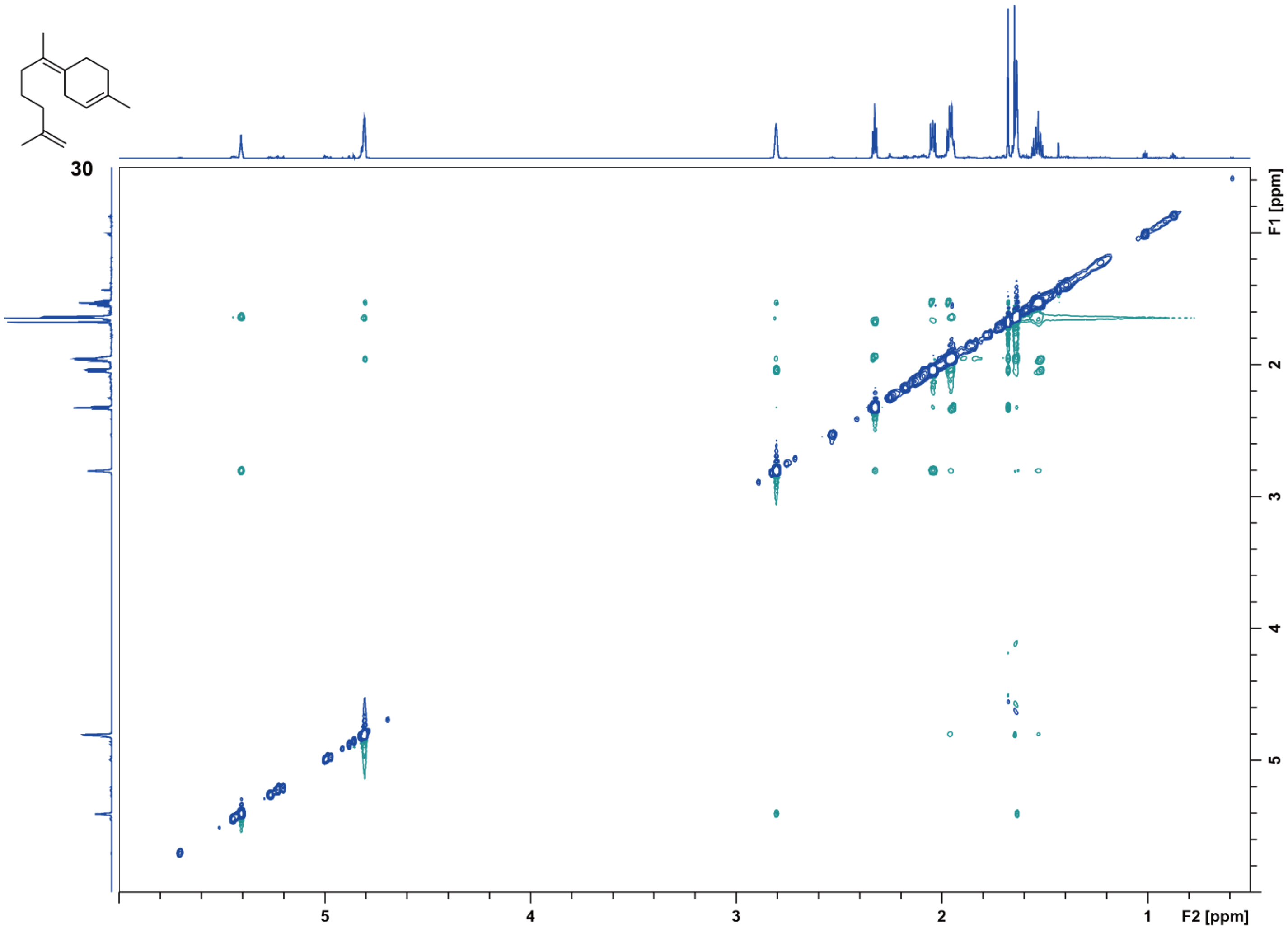

Figure S63. NOESY spectrum of compound **30** (C_6D_6, 700 MHz).

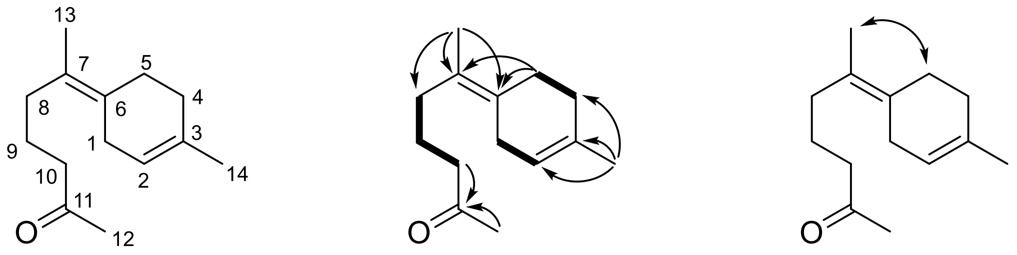

31

Figure S64. Structure elucidation of **31**. Bold: $^1H,^1H$-COSY, single headed arrows: key HMBC, and double headed arrows: key NOESY correlations.

Table S8. NMR data of **31** in C_6D_6 recorded at 298 K.

C[a]	type	^{13}C[b]	^1H[b]
1	CH_2	29.82	2.78 (br s, 2H)
2	CH	121.32	5.40 (m, 1H)
3	C_q	134.11	–
4	CH_2	31.88	1.94 (m, 2H)
5	CH_2	27.37	2.30 (t, J = 6.4 Hz, 2H)
6	C_q	129.39	–
7	C_q	125.34	–
8	CH_2	33.67	1.97 (dd, J = 8.7, 6.7 Hz, 2H)
9	CH_2	22.29	1.61 (m, 2H)
10	CH_2	42.65	1.92 (t, J = 7.1 Hz, 2H)
11	C_q	206.16	–
12	CH_3	29.37	1.64 (s, 3H)
13	CH_3	17.69	1.64 (s, 3H)
14	CH_3	23.55	1.63 (m, 3H)

[a] Carbon numbering as shown in Figure S64. [b] Chemical shifts δ in ppm, multiplicity: s = singlet, d = doublet, t = triplet, m = multiplet, br = broad.

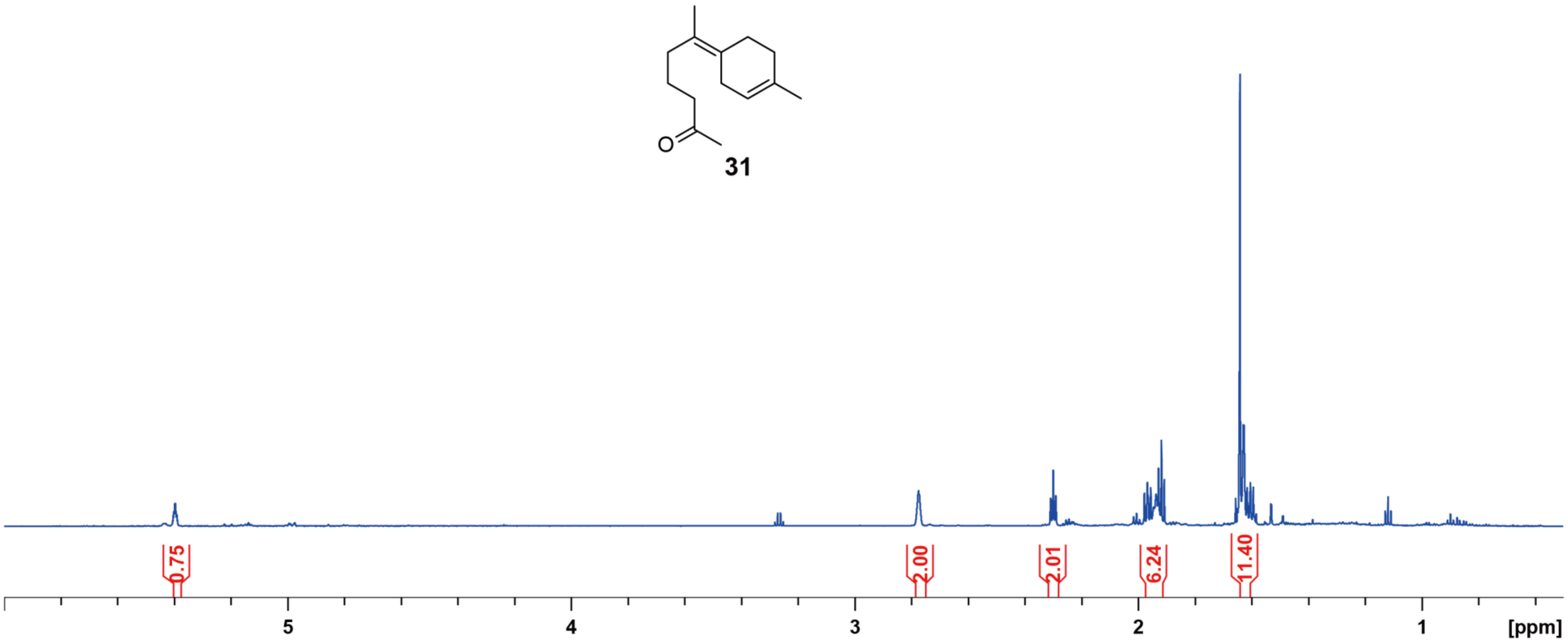

Figure S65. ^{1}H NMR spectrum of compound **31** (C_6D_6, 700 MHz).

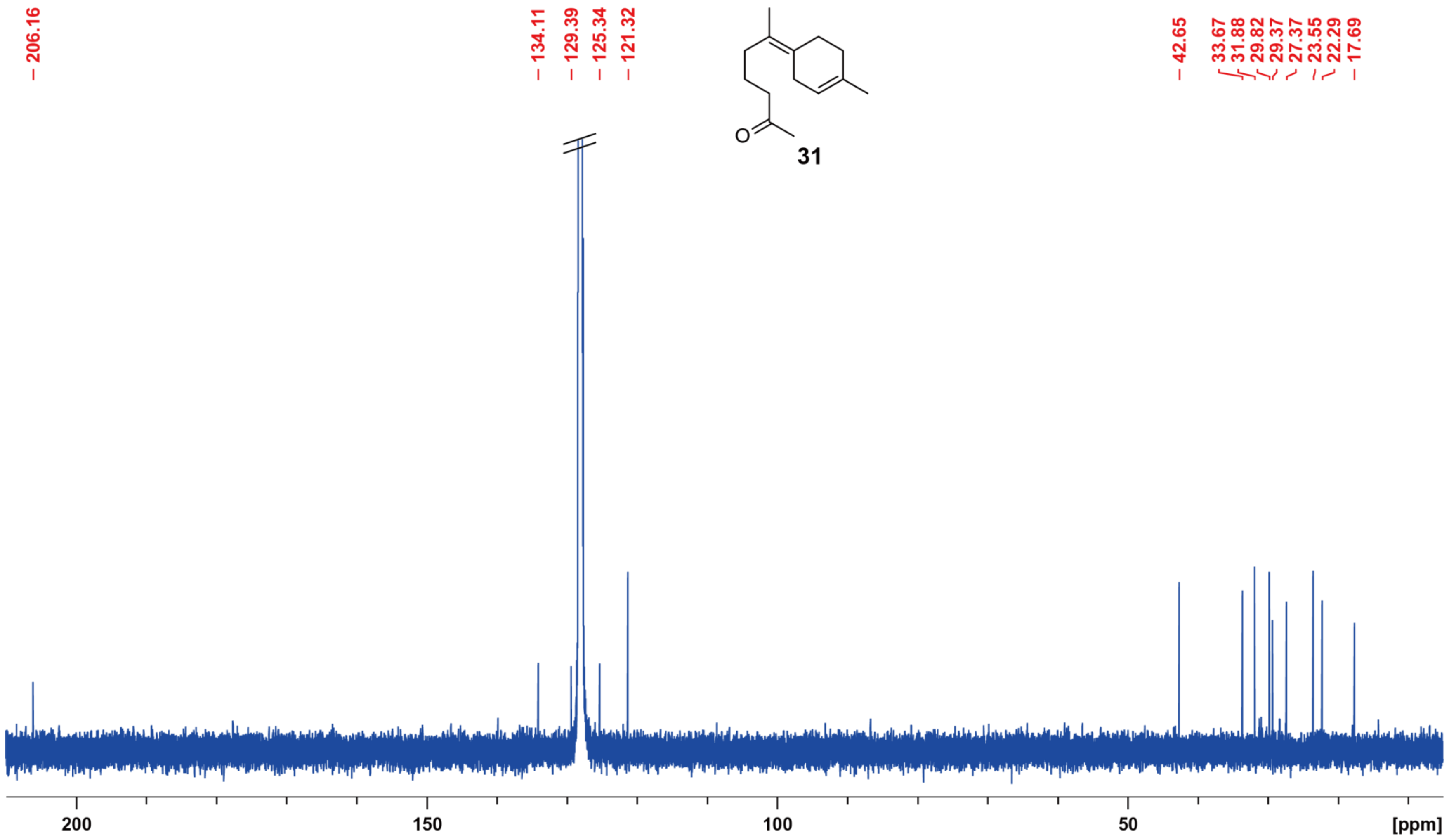

Figure S66. ^{13}C NMR spectrum of compound **31** (C_6D_6, 176 MHz).

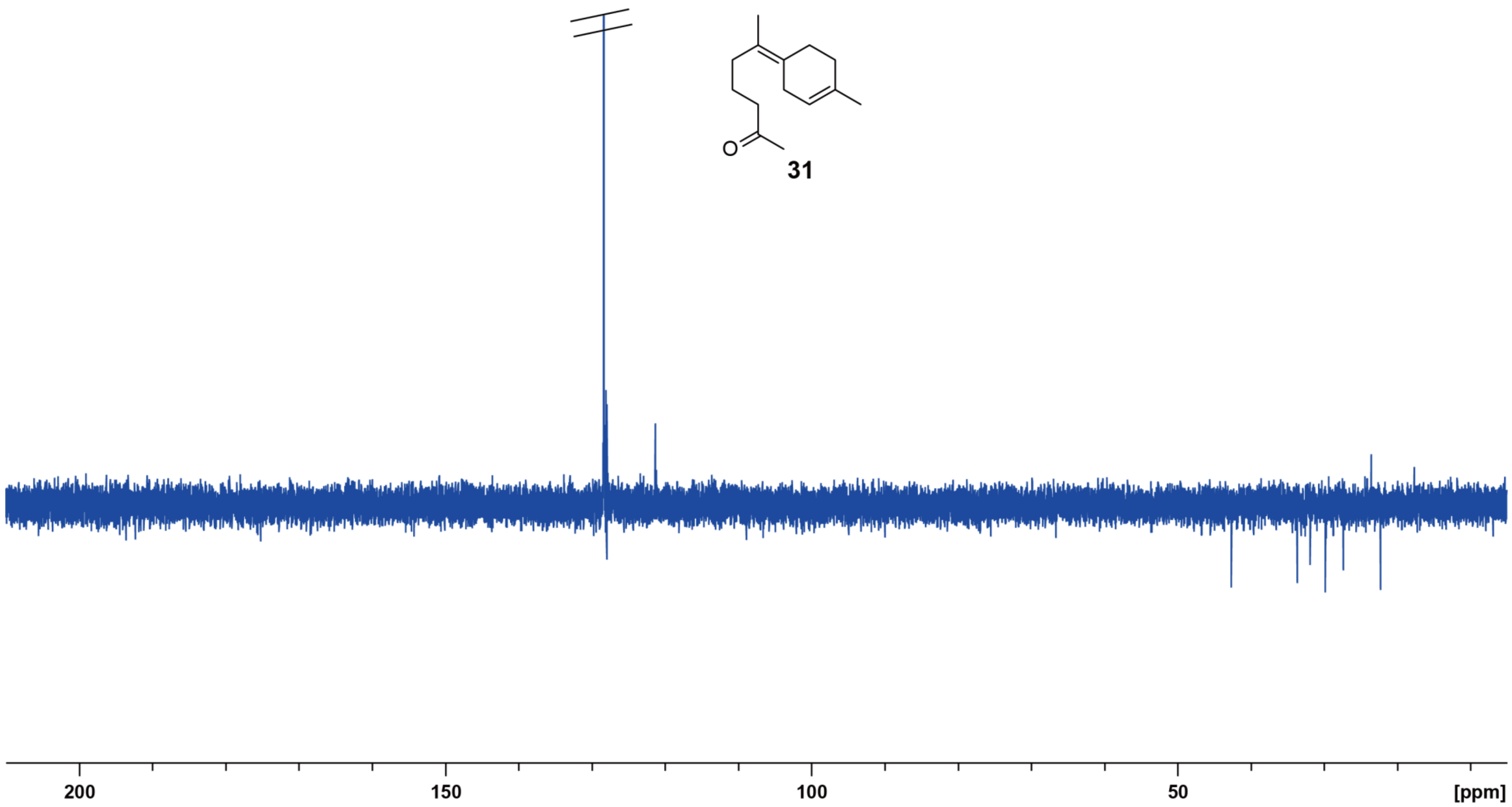

Figure S67. ^{13}C DPET spectrum of compound **31** (C_6D_6, 176 MHz).

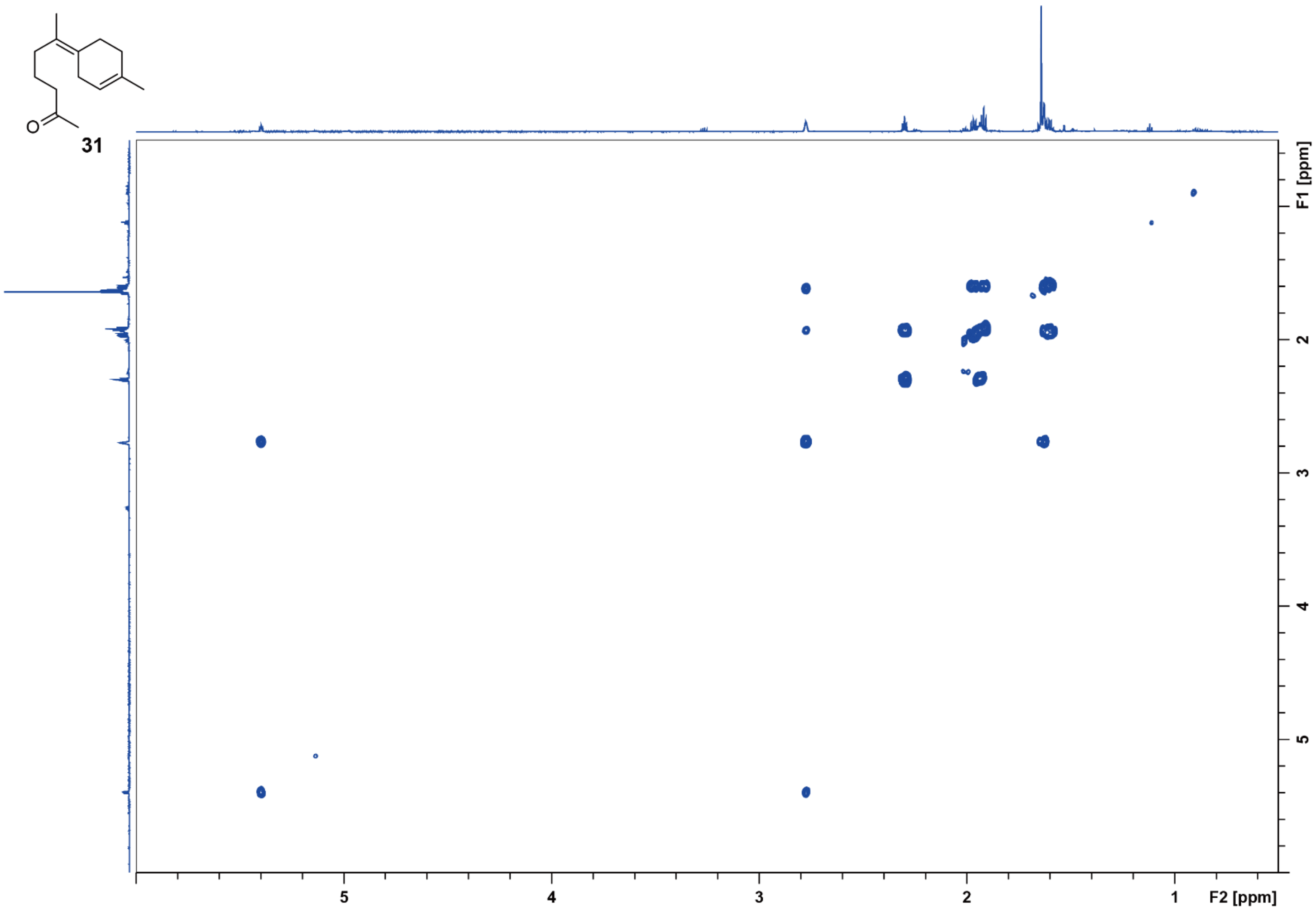

Figure S68. ^{1}H-^{1}H-COSY spectrum of compound **31** (C_6D_6, 700 MHz).

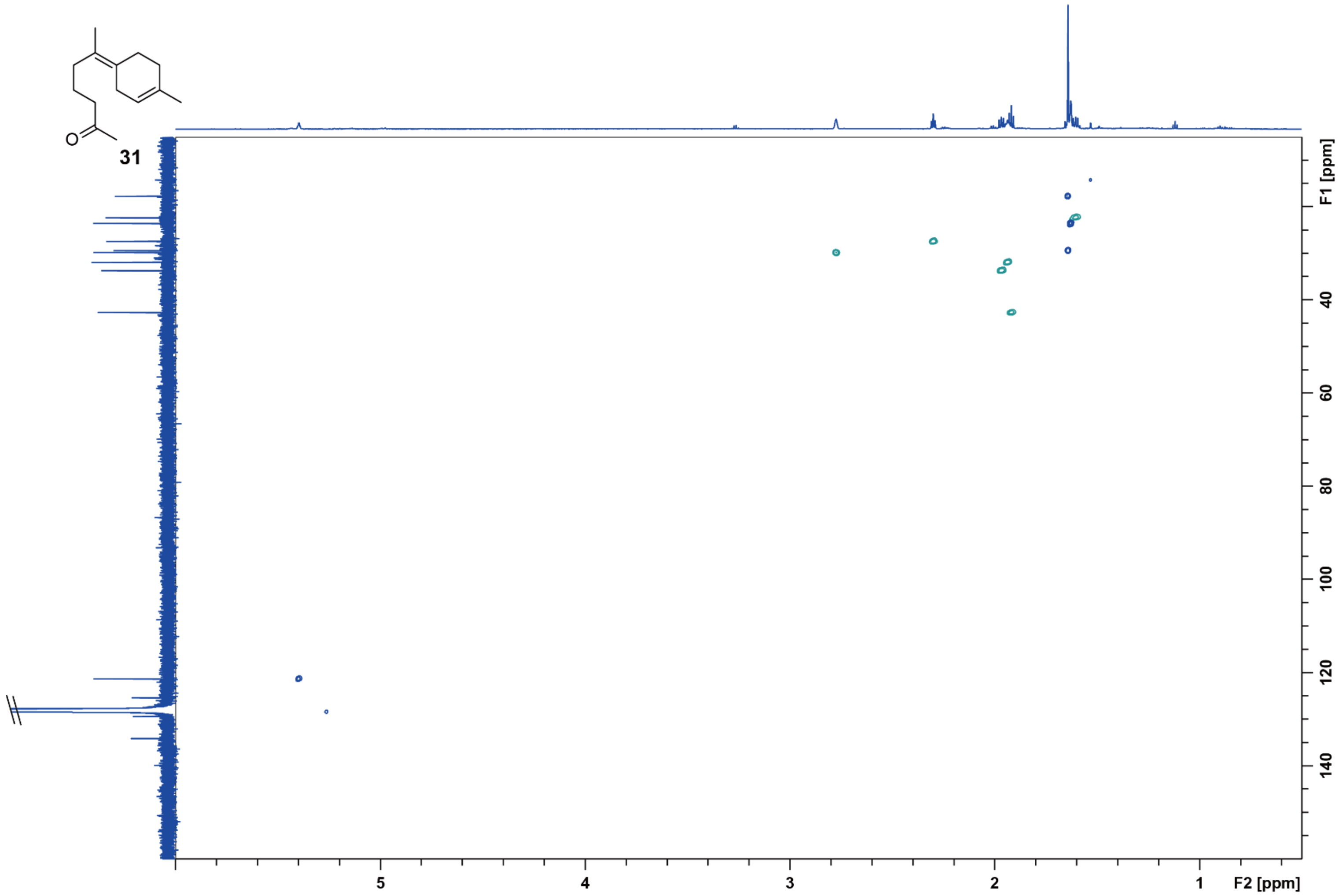

Figure S69. HSQC spectrum of compound **31** (C_6D_6).

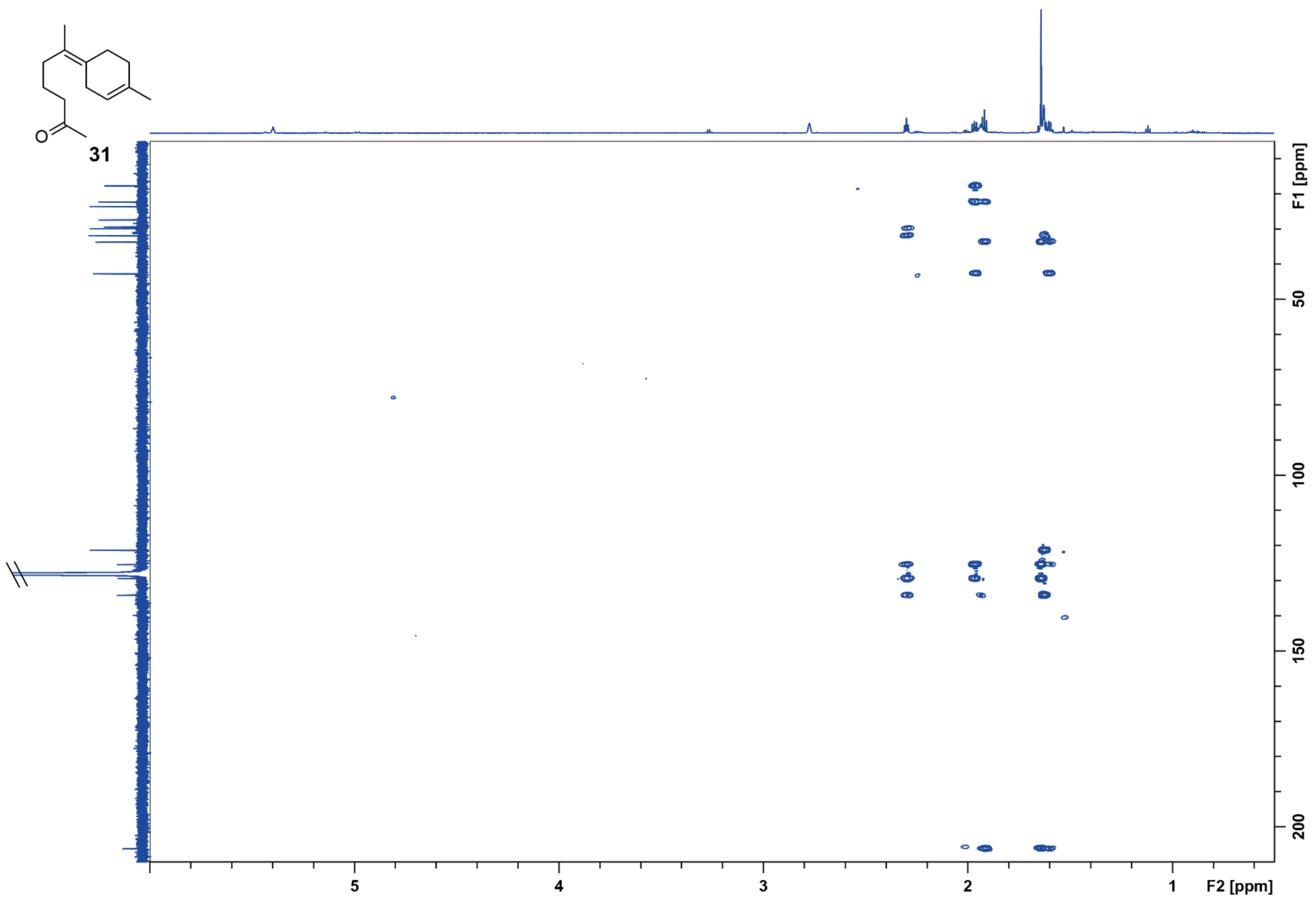

Figure S70. HMBC spectrum of compound **31** (C_6D_6).

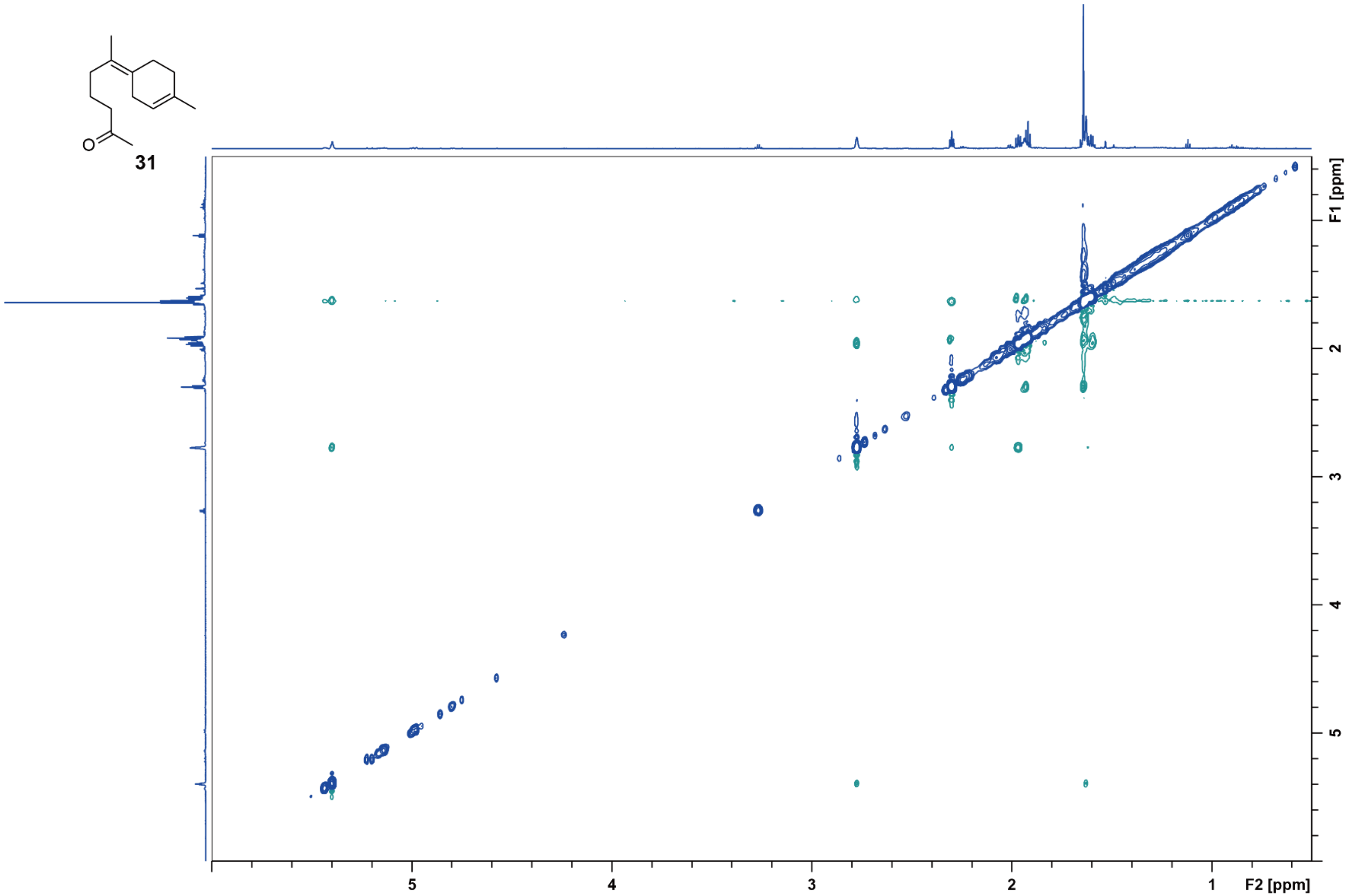

31

Figure S71. NOESY spectrum of compound **31** (C_6D_6).

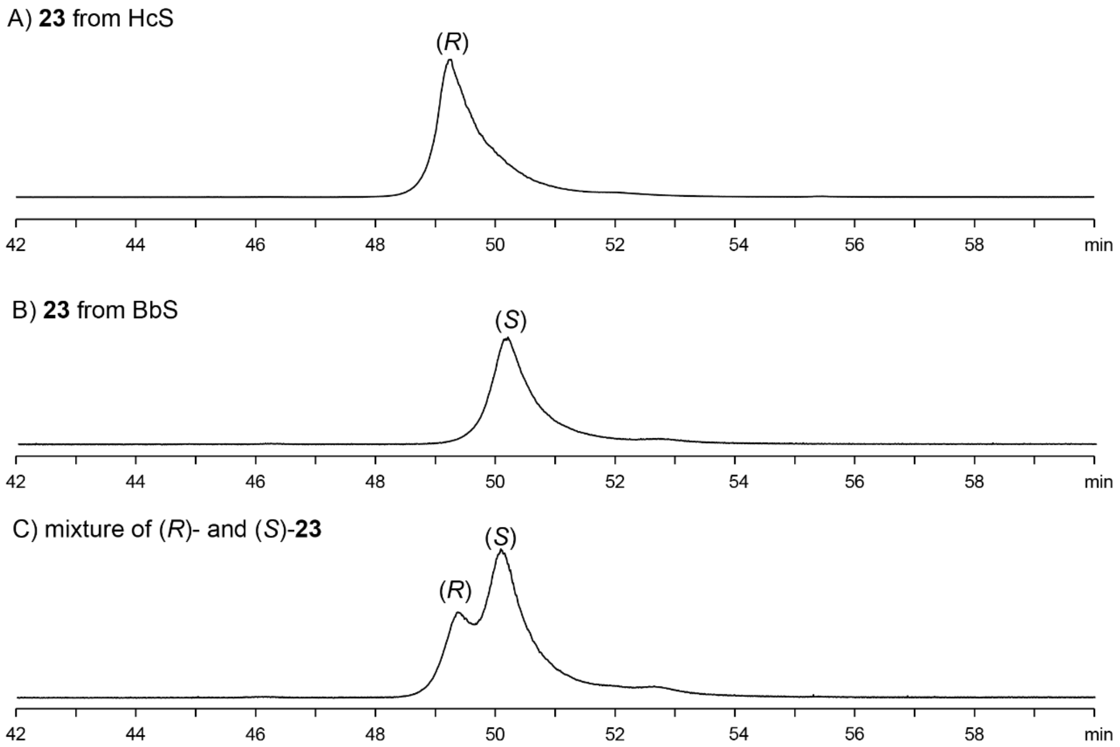

Figure S72. Determination of the absolute configuration of **23** from BbS. The gas chromatograms using a chiral stationary phase of A) (*R*)-**23** from HcS, B) (*S*)-**23** from BbS and C) mixture of (*R*)- and (*S*)-**23**.

Incubation reactions with SmTS6 and substrates 10 – 13

SmTS6 was purified following a published procedure.[4] The obtained protein concentration was ca. 4 mg/mL. The small scale and large scale reactions were performed by the same procedure as reported above for enzyme reactions with HcS.

(*E*)-7,11-Dimethyl-3-methylenedodeca-1,6-diene (32). Yield: 1.2 mg, 5.8 μmol, 3%. TLC (pentane, 100%): R_f = 0.72. MS spectrum cf. Figure S74A. GC (HP5-MS): *I* = 1426. HRMS (APCI): *m*/*z* = 207.2110 (calc. for $[C_{15}H_{26} + H]^+$: 207.2107). IR (diamond ATR): $\tilde{\nu}$ = 3090 (w), 2955 (s), 2929 (s), 2869 (m), 2274 (w), 1595 (m), 1462 (m), 1384 (w), 1345 (w), 1261 (m), 1096 (m), 1017 (m), 990 (m), 893 (s), 815 (m), 806 (m), 543 (s), 424 (w), 409 (w) cm^{-1}. NMR data cf. Table S9 and Figures S75 – S82.

(*R*,*E*)-3,7,11-Trimethyldodeca-1,6-dien-3-ol (33). Yield: 1.5 mg, 6.7 μmol, 3%. TLC (pentane/Et_2O, 1:1): R_f = 0.69. Optical rotation: $[\alpha]_D^{25}$ = –16 (*c* 0.15, CH_2Cl_2). MS spectrum cf. Figure S74B. GC (HP5-MS): *I* = 1538. HRMS (APCI): *m*/*z* = 225.2214 (calc. for $[C_{15}H_{28}O + H]^+$: 225.2213). IR (diamond ATR): $\tilde{\nu}$ = 3398 (br w), 2955 (s), 2927 (s), 2869 (m), 1668 (w), 1642 (w), 1460 (m), 1411 (w), 1383 (w), 1366 (m), 1345 (w), 1261 (w), 1229 (w), 1168 (w), 1124 (w), 1107 (m), 995 (m), 919 (s), 816 (w), 803 (w), 736 (w), 689 (w), 586 (w), 544 (m), 527 (w), 467 (w), 440 (w) cm^{-1}. NMR data cf. Table S10 and Figures S83 – S90.

(1*E*,5*E*,9*E*)-1,5,9-Trimethylcyclododeca-1,5,9-triene (34). Yield: 1.5 mg, 7.3 μmol, 3%. TLC (hexane, 100%): R_f = 0.50. MS spectrum cf. Figure S74C. GC (HP5-MS): *I* = 1578. HRMS (APCI): *m*/*z* = 204.1871 (calc. for $[C_{15}H_{24}]^+$: 204.1873). IR (diamond ATR): $\tilde{\nu}$ = 2979 (m), 2953 (m), 2905 (s), 2847 (s), 1438 (m), 1383 (w), 1260 (m), 1226 (w), 1147 (w), 1083 (m), 1015 (w), 928 (w), 887 (w), 827 (s), 803 (s), 736 (w), 544 (m), 490 (w), 450 (w) cm^{-1}. NMR data cf. Table S11 and Figures S92 – S99.

(4*E*,8*E*)-1,5,9-Trimethyl-cyclododeca-4,8-dien-1-ol (35). Yield: 2.8 mg, 12.6 μmol, 5%. TLC (hexane/Et_2O, 1:1): R_f = 0.40. Optical rotation: $[\alpha]_D^{25}$ = +5.2 (*c* 0.28, CH_2Cl_2). MS spectrum cf. Figure S74D. GC (HP5-MS): *I* = 1727. HRMS (APCI): *m*/*z* = 223.2066 (calc. for $[C_{15}H_{26}O + H]^+$: 223.2056). IR (diamond ATR): $\tilde{\nu}$ = 3370 (br w), 2962 (m), 2912 (m), 2850 (m), 1739 (w), 1667 (w), 1438 (m), 1382 (w), 1371 (w), 1260 (m), 1227 (w), 1200 (w), 1145 (m), 1088 (m), 1020 (w), 937 (w), 922 (w), 905 (w), 878 (w), 863 (w), 801 (s), 661 (w), 543 (m), 491 (w) cm^{-1}. NMR data cf. Table S12 and Figures S100 – S107.

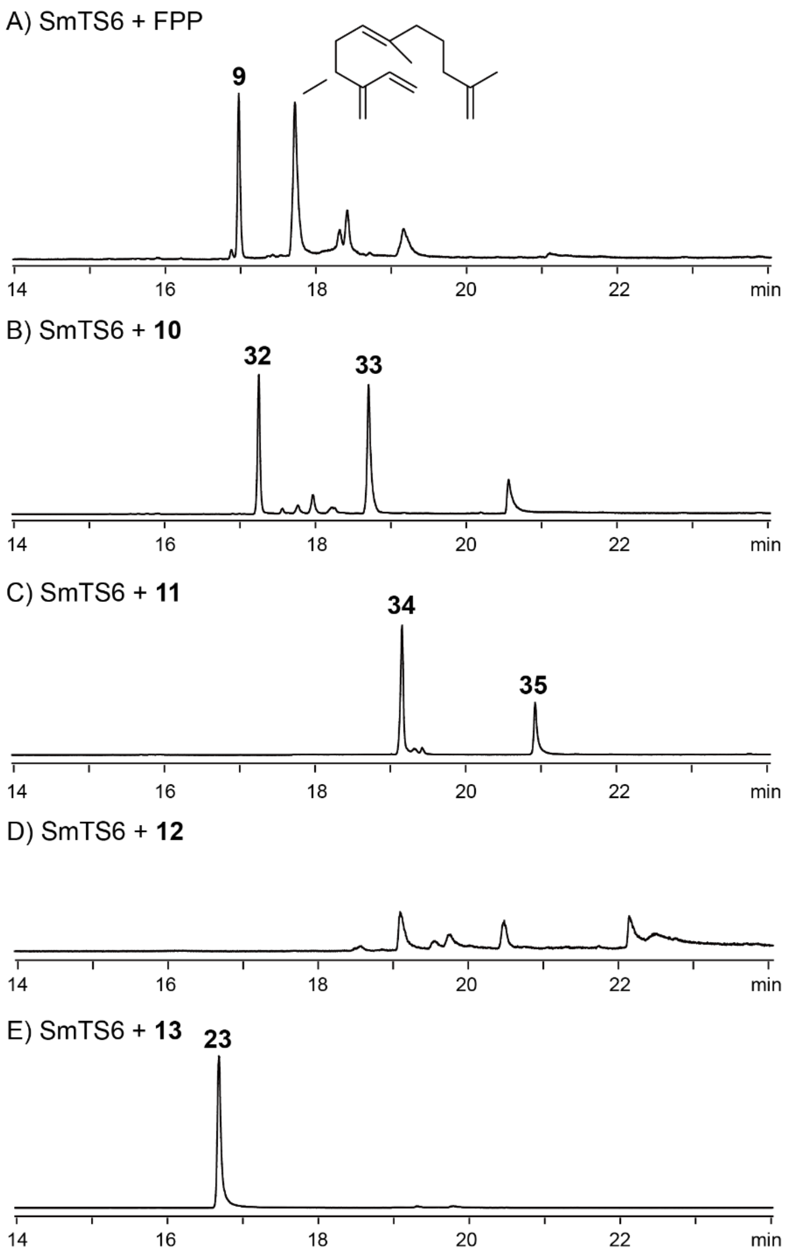

Figure S73. Total ion chromatograms of the extracts from the incubation reactions with SmTS6 and A) FPP, B) substrates **10**, C) **11**, D) **12** and E) **13**.

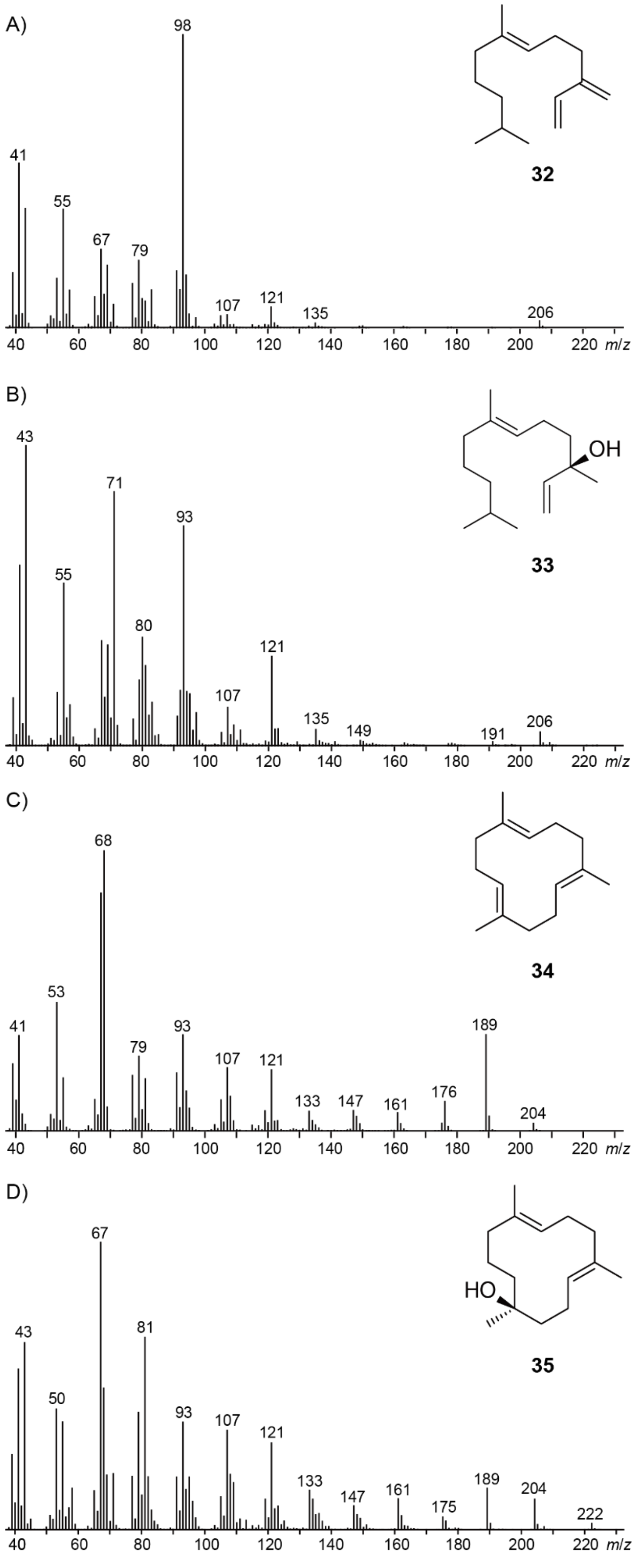

Figure S74. Mass spectra of A) **32**, B) **33**, C) **34** and D) **35**.

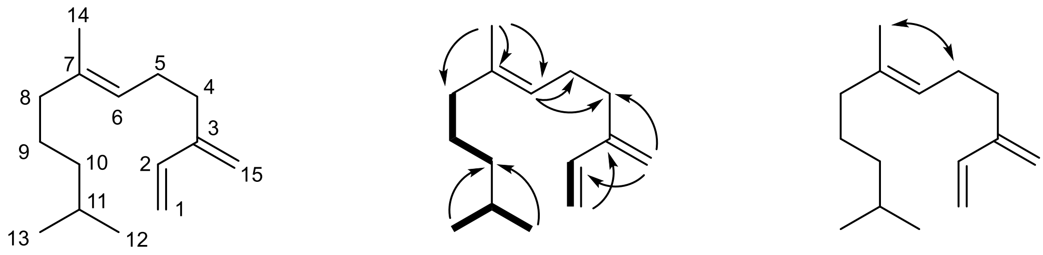

32

Figure S75. Structure elucidation of **32**. Bold: $^{1}H,^{1}H$-COSY, single headed arrows: key HMBC, and double headed arrows: key NOESY correlations.

Table S9. NMR data of **32** in C_6D_6 recorded at 298 K.

C[a]	type	^{13}C[b]	^{1}H[b]
1	CH_2	113.17	5.22 (d, *J* = 17.6 Hz, 1H)
			4.98 (dq, *J* = 10.8, 1.1 Hz, 1H)
2	CH	139.45	6.38 (ddd, *J* = 17.6, 10.8, 0.7 Hz, 1H)
3	C_q	146.47	–
4	CH_2	31.92	2.27 (m, 2H)
5	CH_2	27.07	2.27 (m, 2H)
6	CH	124.42	5.25 (m, 1H)
7	C_q	135.73	–
8	CH_2	40.34	1.99 (t, *J* = 7.6 Hz, 2H)
9	CH_2	26.17	1.41 (m, 2H)
10	CH_2	38.98	1.16 (m, 2H)
11	CH	28.27	1.50 (dt, *J* = 13.4, 6.6 Hz, 1H)
12,13	2 x CH_3	22.85	0.89 (d, *J* = 6.6 Hz, 6H)
14	CH_3	16.03	1.56 (br s, 3H)
15	CH_2	116.06	5.00 (m, 2H)

[a] Carbon numbering as shown in Figure S75. [b] Chemical shifts δ in ppm, multiplicity: s = singlet, d = doublet, t = triplet, q = quartet, m = multiplet, br = broad.

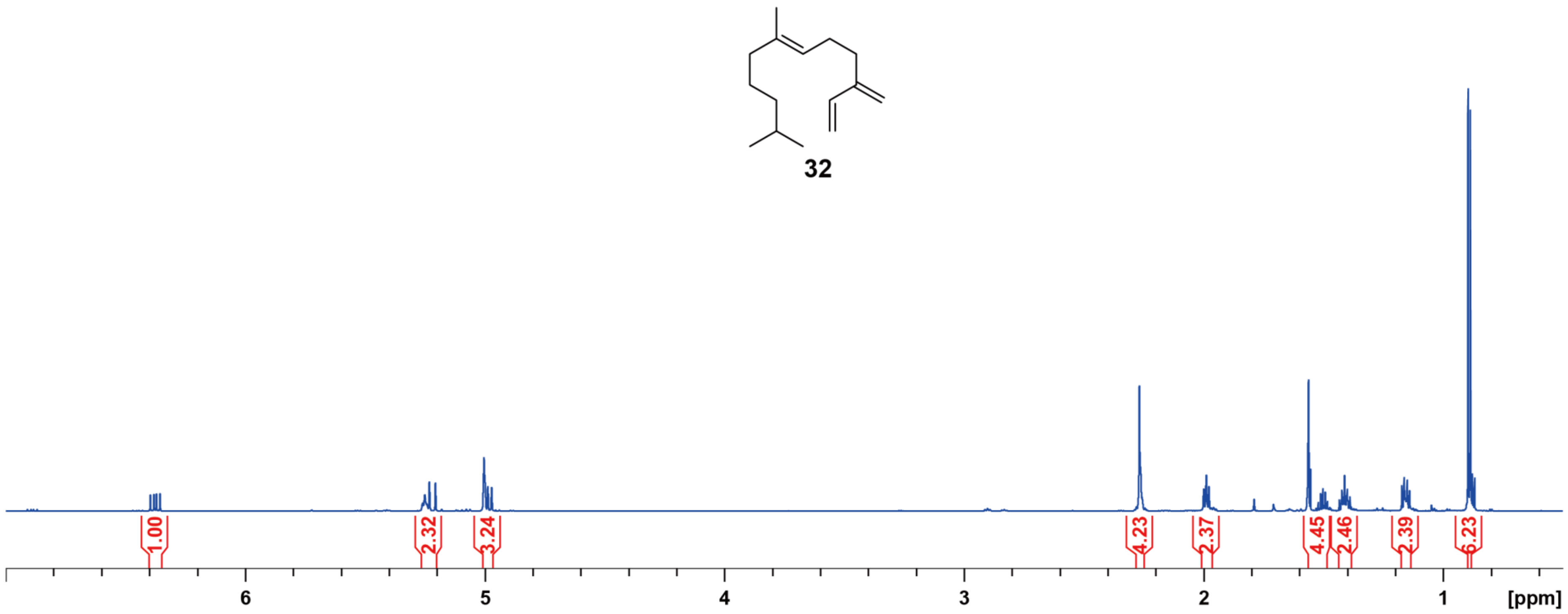

Figure S76. ^{1}H NMR spectrum of compound **32** (C_6D_6, 700 MHz).

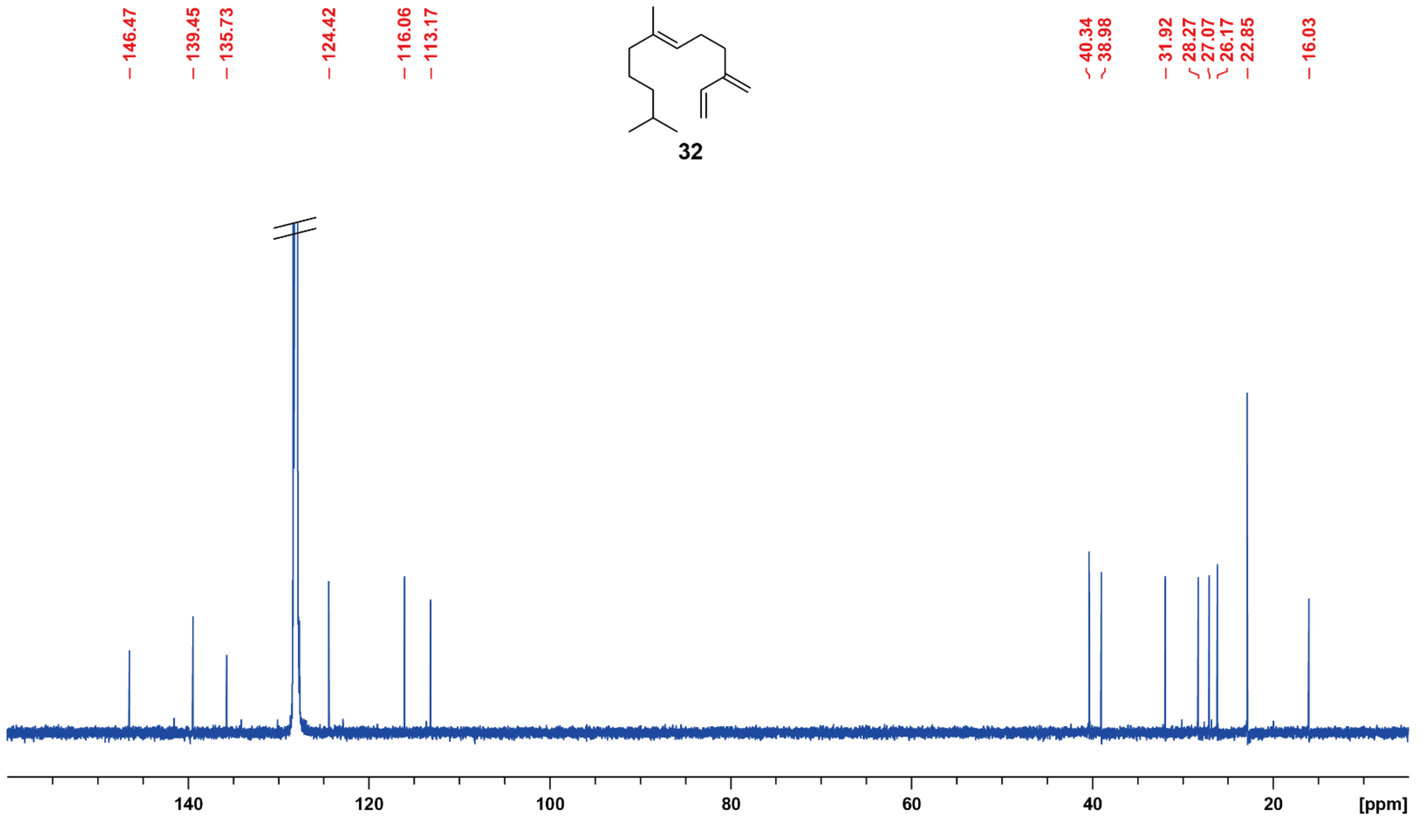

Figure S77. ^{13}C NMR spectrum of compound **32** (C_6D_6, 176 MHz).

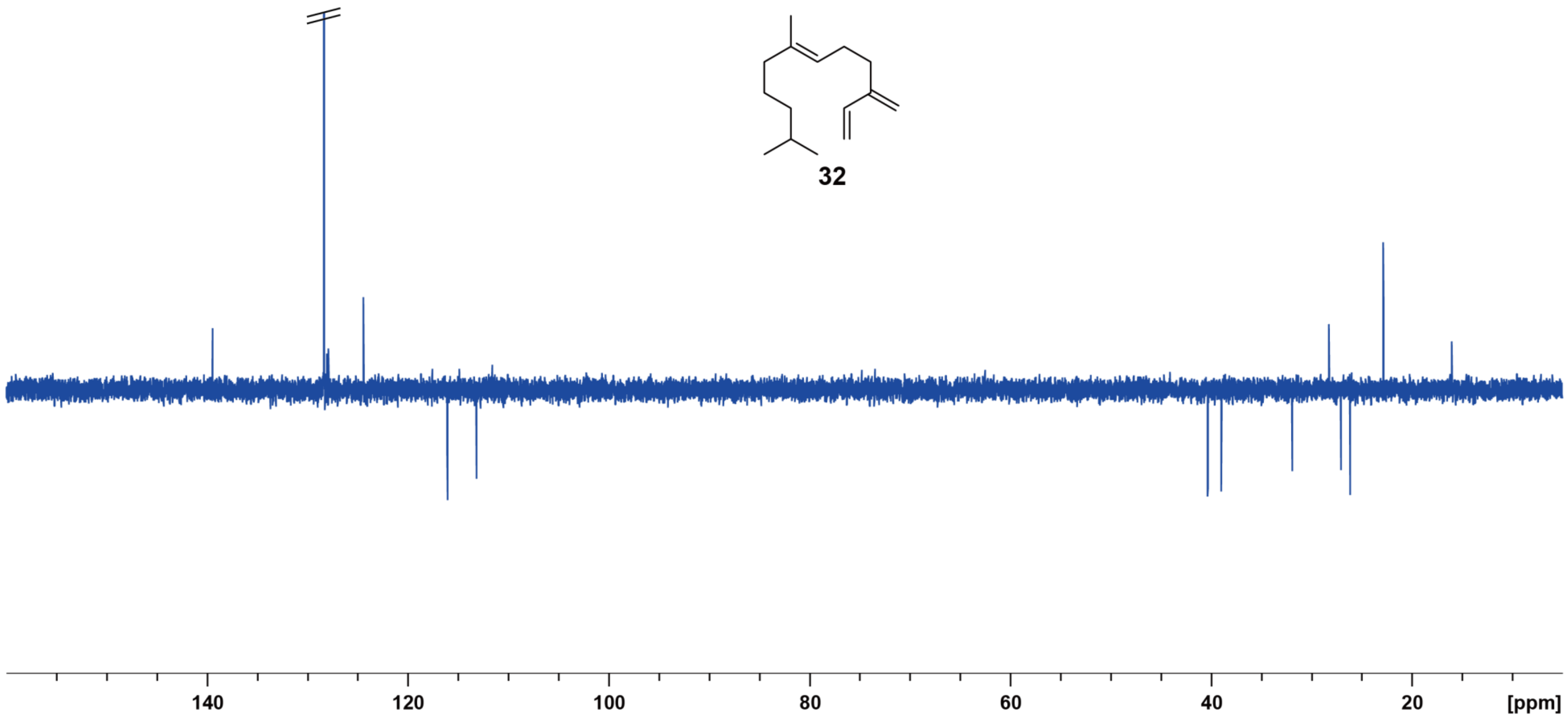

Figure S78. ^{13}C DPET spectrum of compound **32** (C_6D_6, 176 MHz).

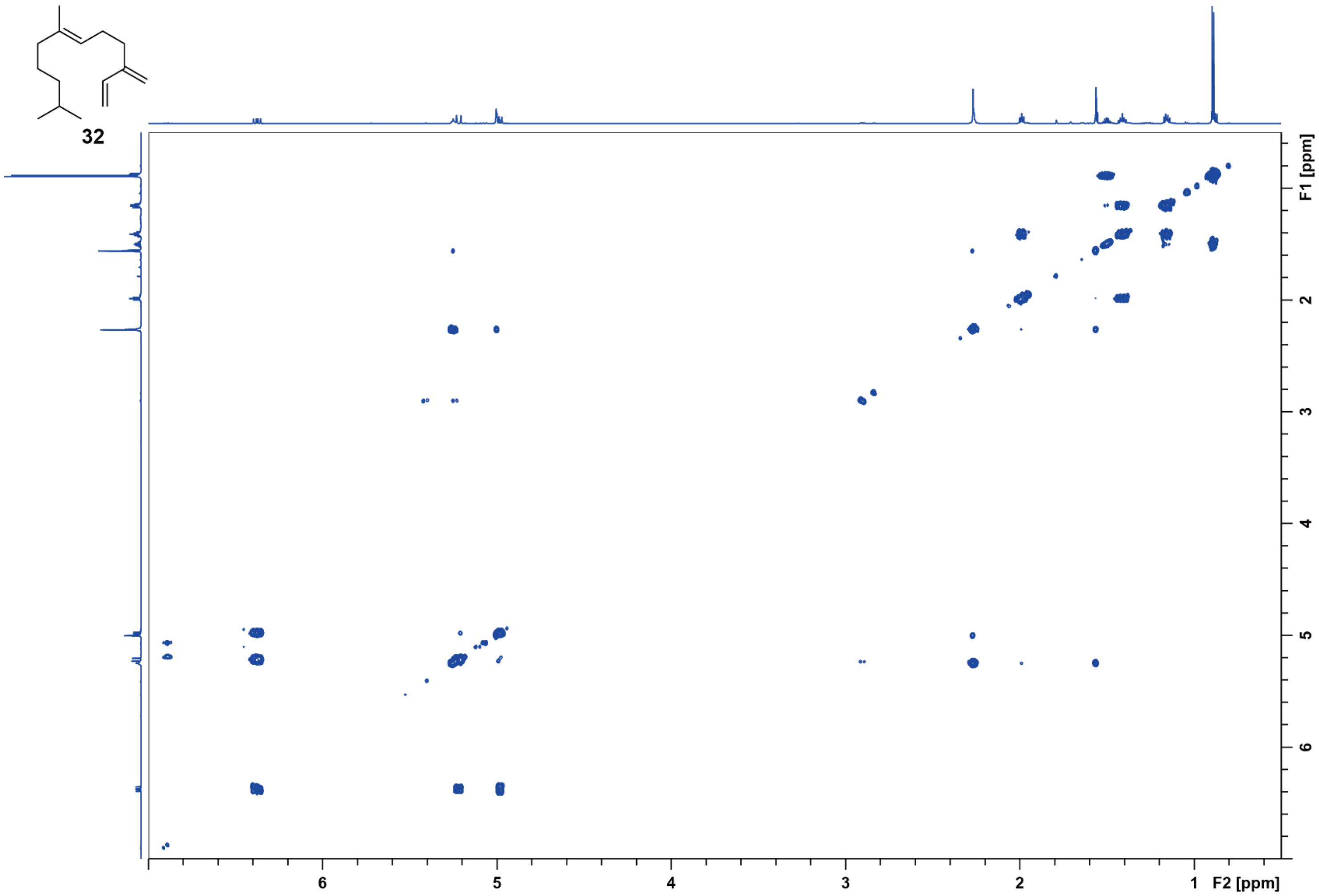

Figure S79. ^{1}H-^{1}H-COSY spectrum of compound **32** (C_6D_6, 700 MHz).

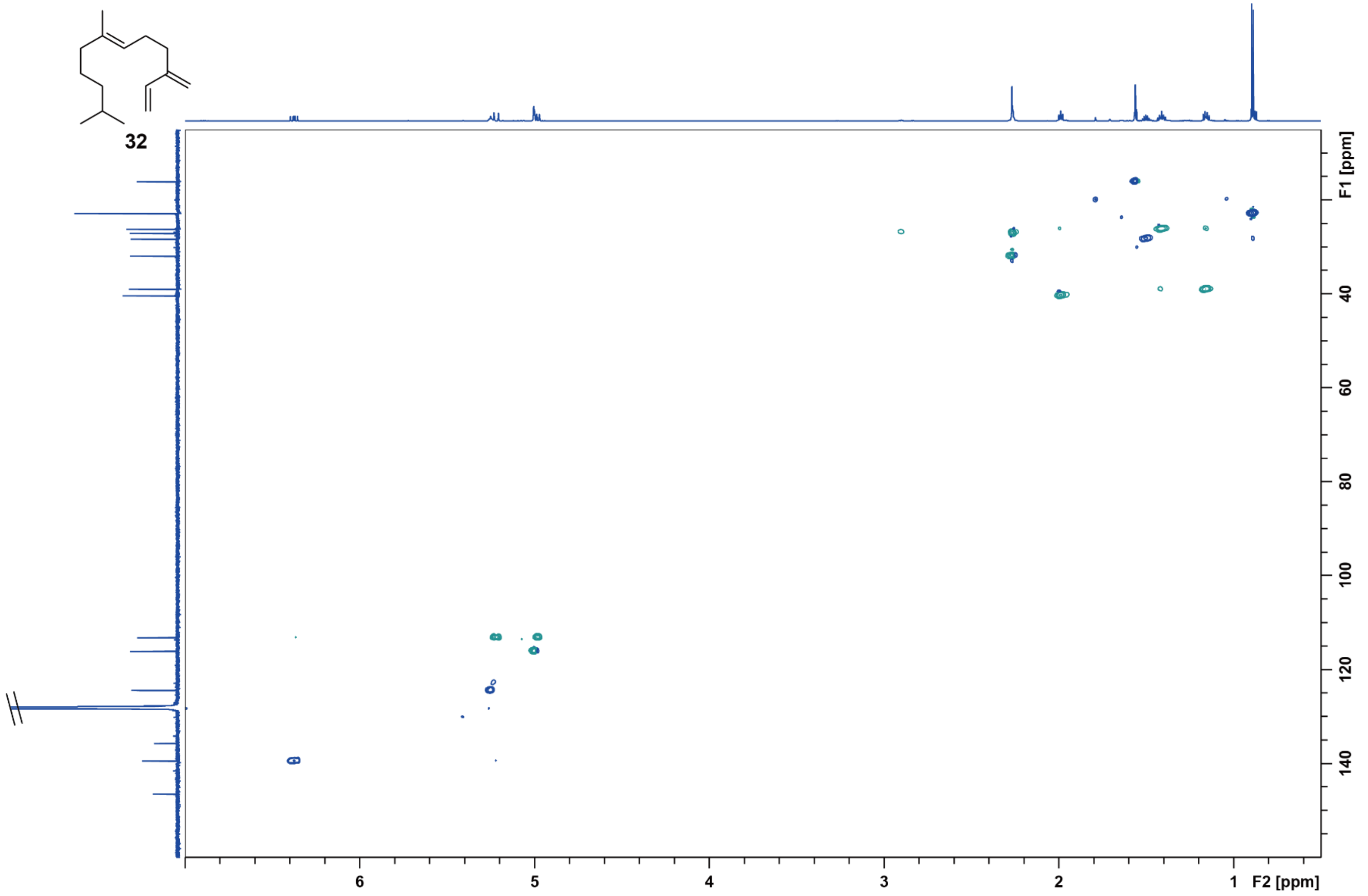

Figure S80. HSQC spectrum of compound **32** (C_6D_6).

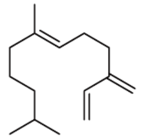

32

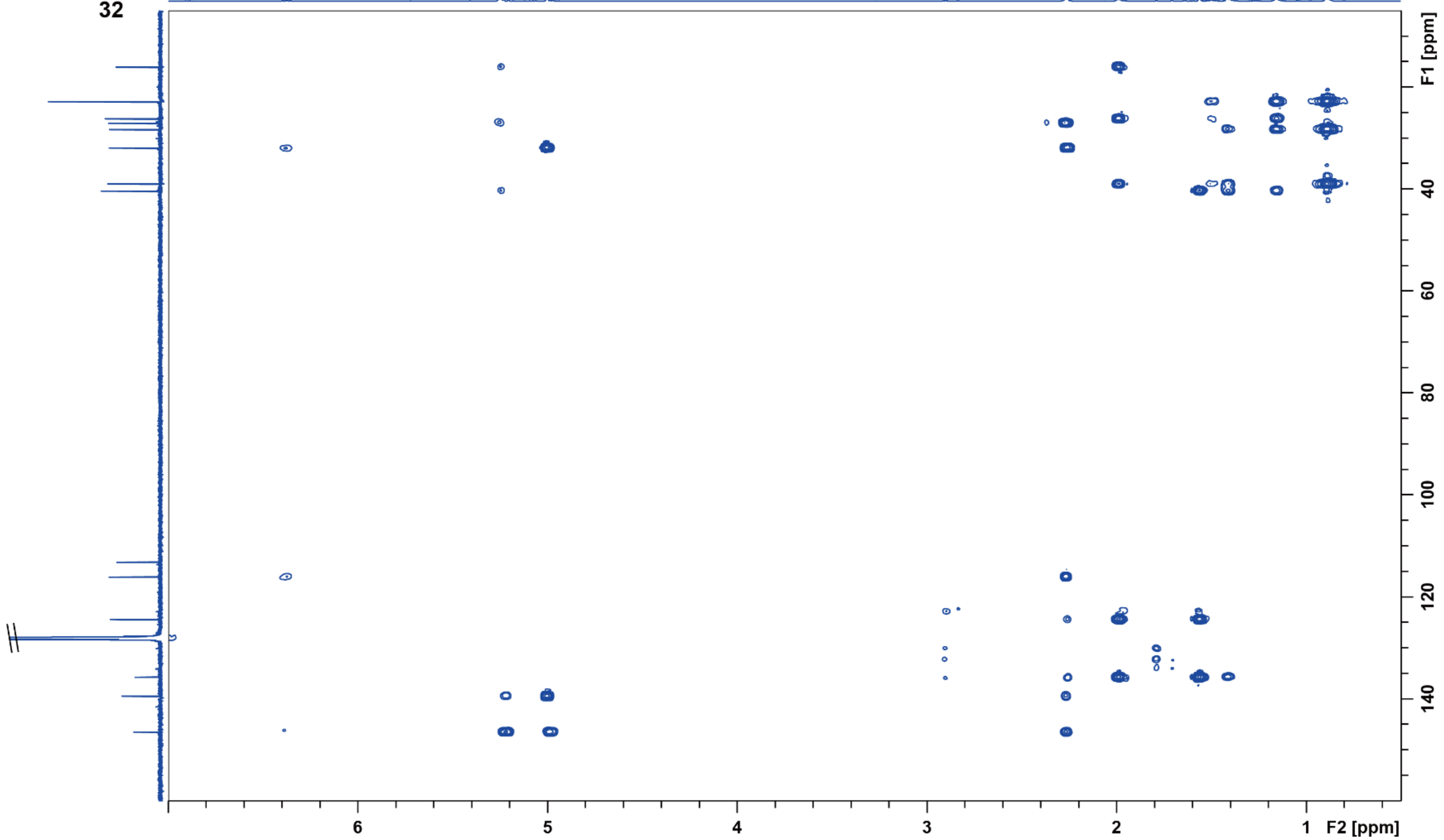

Figure S81. HMBC spectrum of compound **32** (C_6D_6).

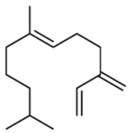

32

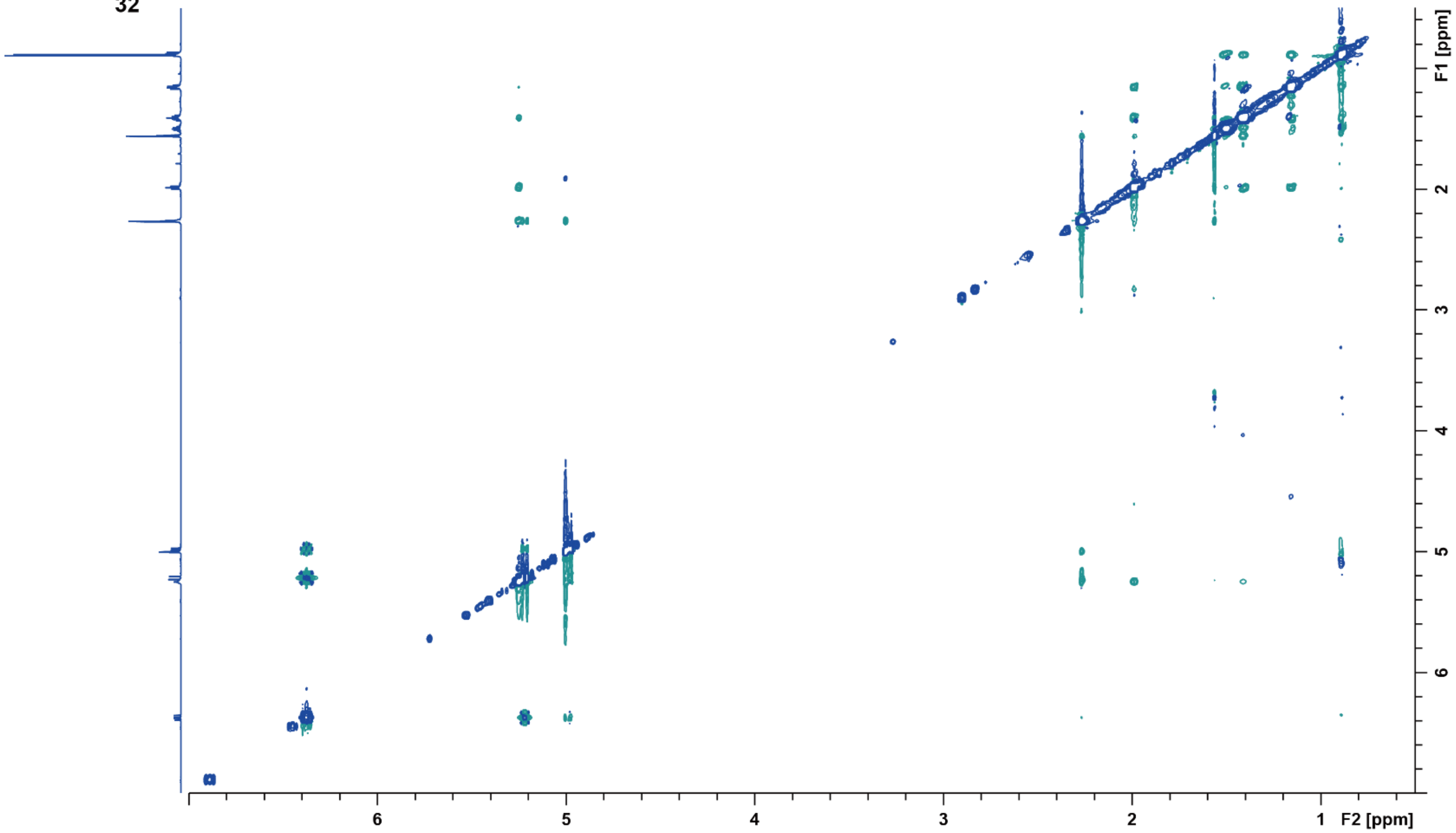

Figure S82. NOESY spectrum of compound **32** (C_6D_6, 700 MHz).

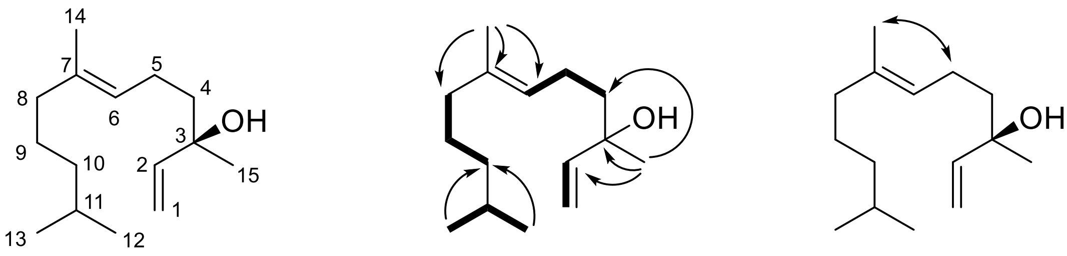

33

Figure S83. Structure elucidation of **33**. Bold: $^{1}H,^{1}H$-COSY, single headed arrows: key HMBC, and double headed arrows: key NOESY correlations.

Table S10. NMR data of **33** in C_6D_6 recorded at 298 K.

C[a]	type	^{13}C[b]	^{1}H[b]
1	CH_2	111.43	5.21 (dd, J = 17.3, 1.6 Hz, 1H)
			4.96 (dd, J = 10.7, 1.6 Hz, 1H)
2	CH	145.70	5.76 (dd, J = 17.3, 10.7 Hz, 1H)
3	C_q	73.02	–
4	CH_2	42.67	1.53 (m, 1H); 1.48 (m, 1H)
5	CH_2	23.12	2.15 (m, 1H); 2.09 (m, 1H)
6	CH	124.91	5.24 (tq, J = 7.2, 1.3 Hz, 1H)
7	C_q	135.55	–
8	CH_2	40.36	1.98 (t, J = 7.6 Hz, 2H)
9	CH_2	26.19	1.42 (m, 2H)
10	CH_2	39.01	1.16 (m, 2H)
11	CH	28.28	1.51 (m, 1H)
12,13	2 x CH_3	22.86	0.89 (d, J = 6.6 Hz, 6H)
14	CH_3	16.04	1.59 (s, 3H)
15	CH_3	28.34	1.12 (s, 3H)

[a] Carbon numbering as shown in Figure S83. [b] Chemical shifts δ in ppm, multiplicity: s = singlet, d = doublet, t = triplet, q = quartet, m = multiplet.

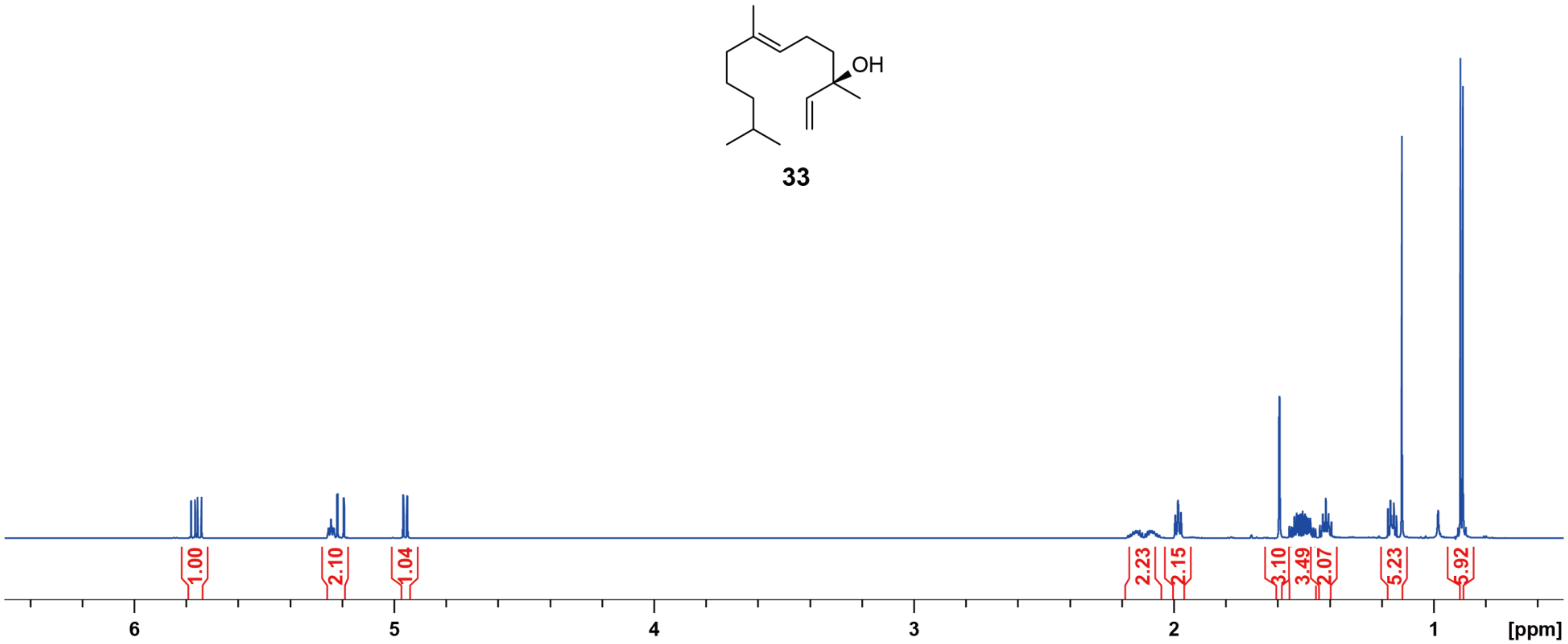

Figure S84. ^{1}H NMR spectrum of compound **33** (C_6D_6, 700 MHz).

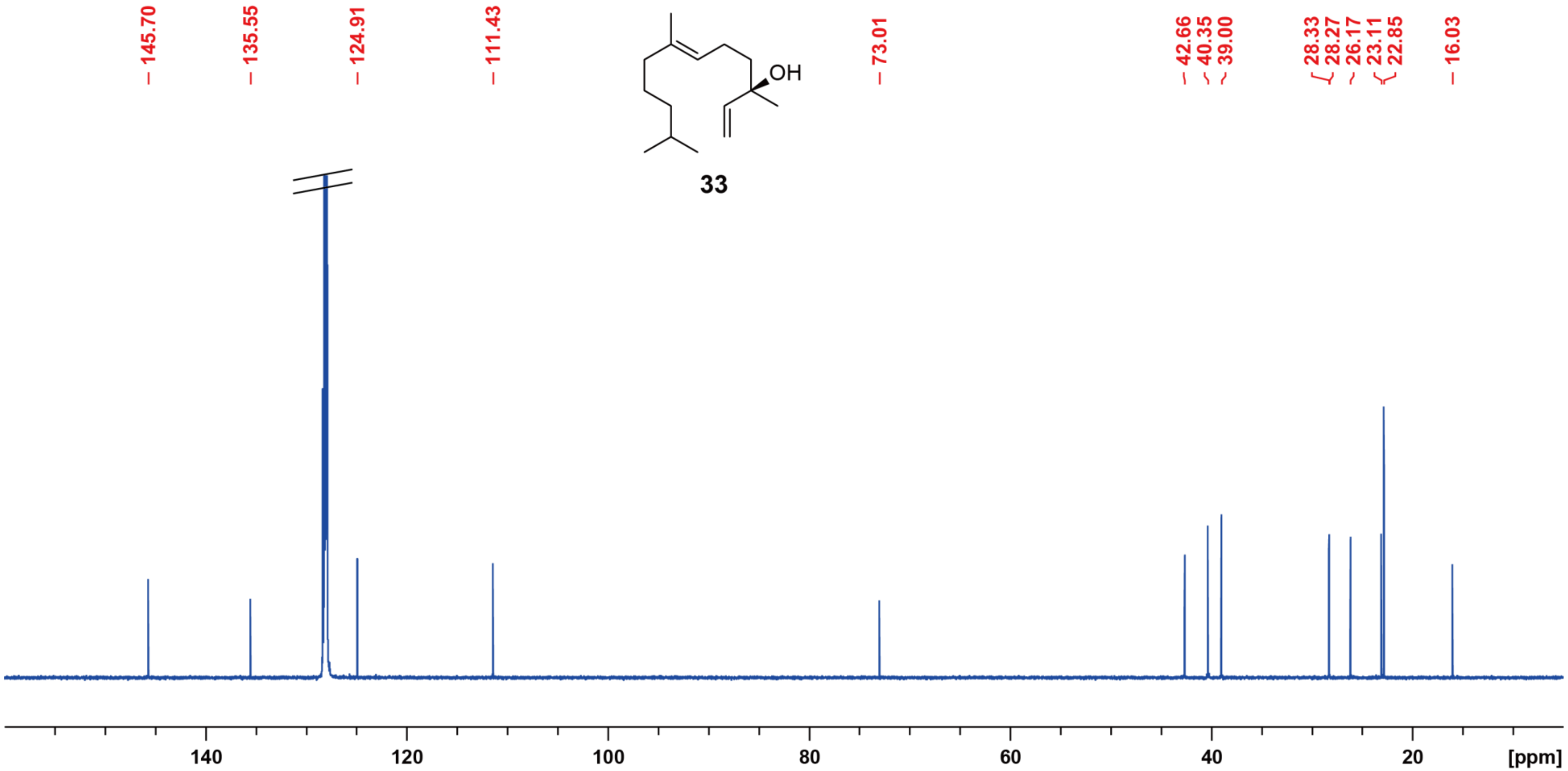

Figure S85. ^{13}C NMR spectrum of compound **33** (C_6D_6, 176 MHz).

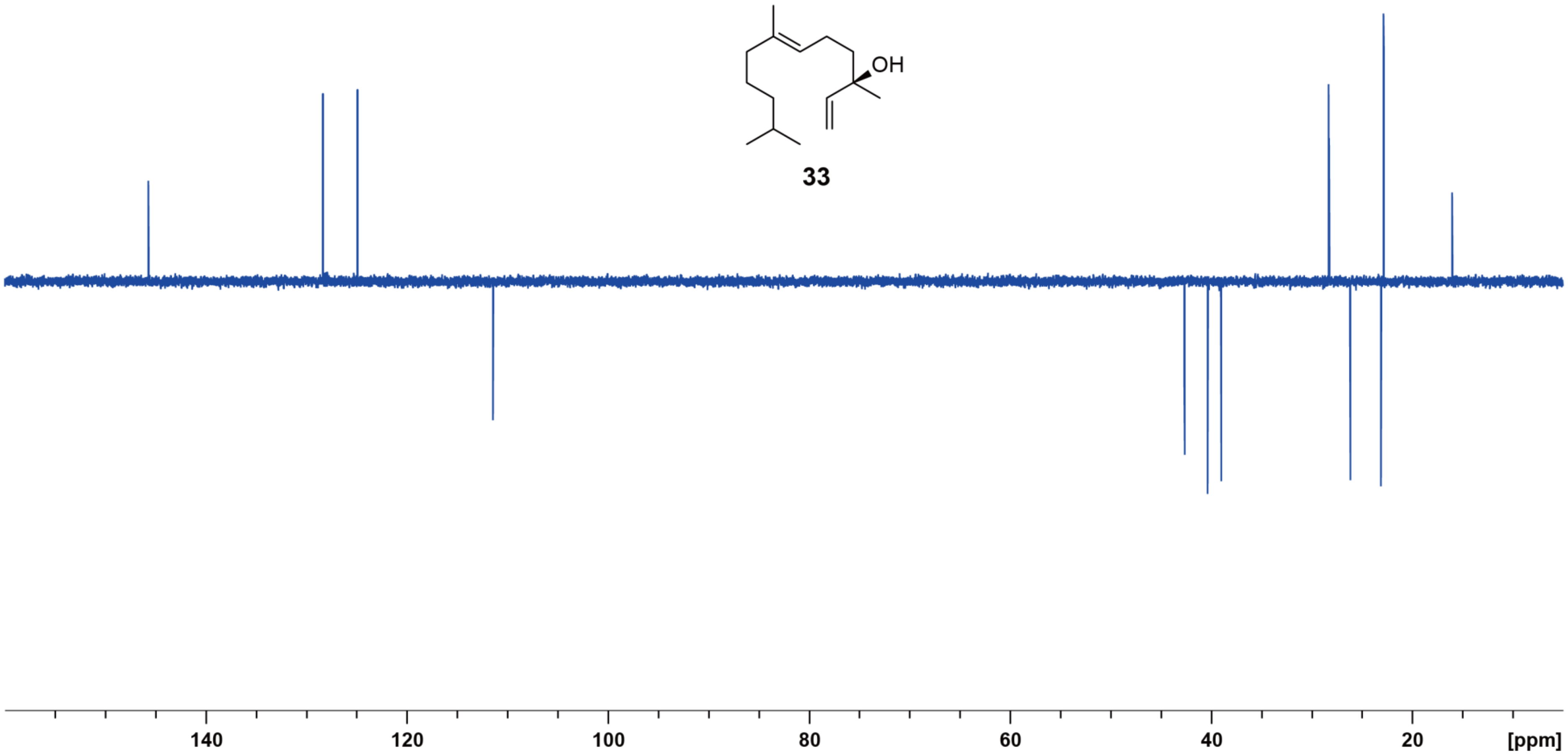

Figure S86. ^{13}C DEPT spectrum of compound **33** (C_6D_6, 176 MHz).

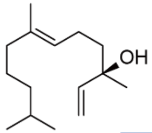

33

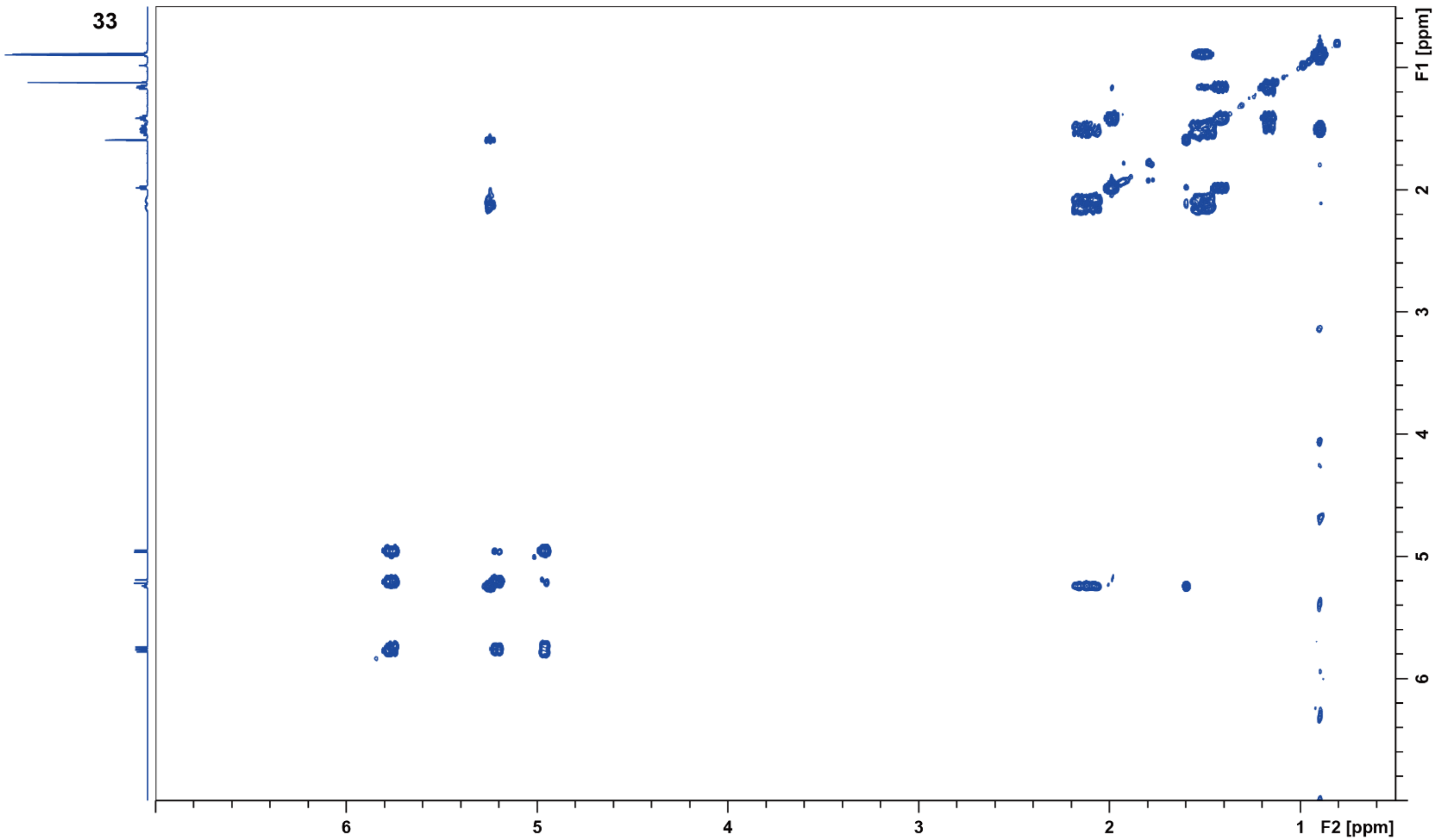

Figure S87. ^{1}H-^{1}H-COSY spectrum of compound **33** (C_6D_6, 700 MHz).

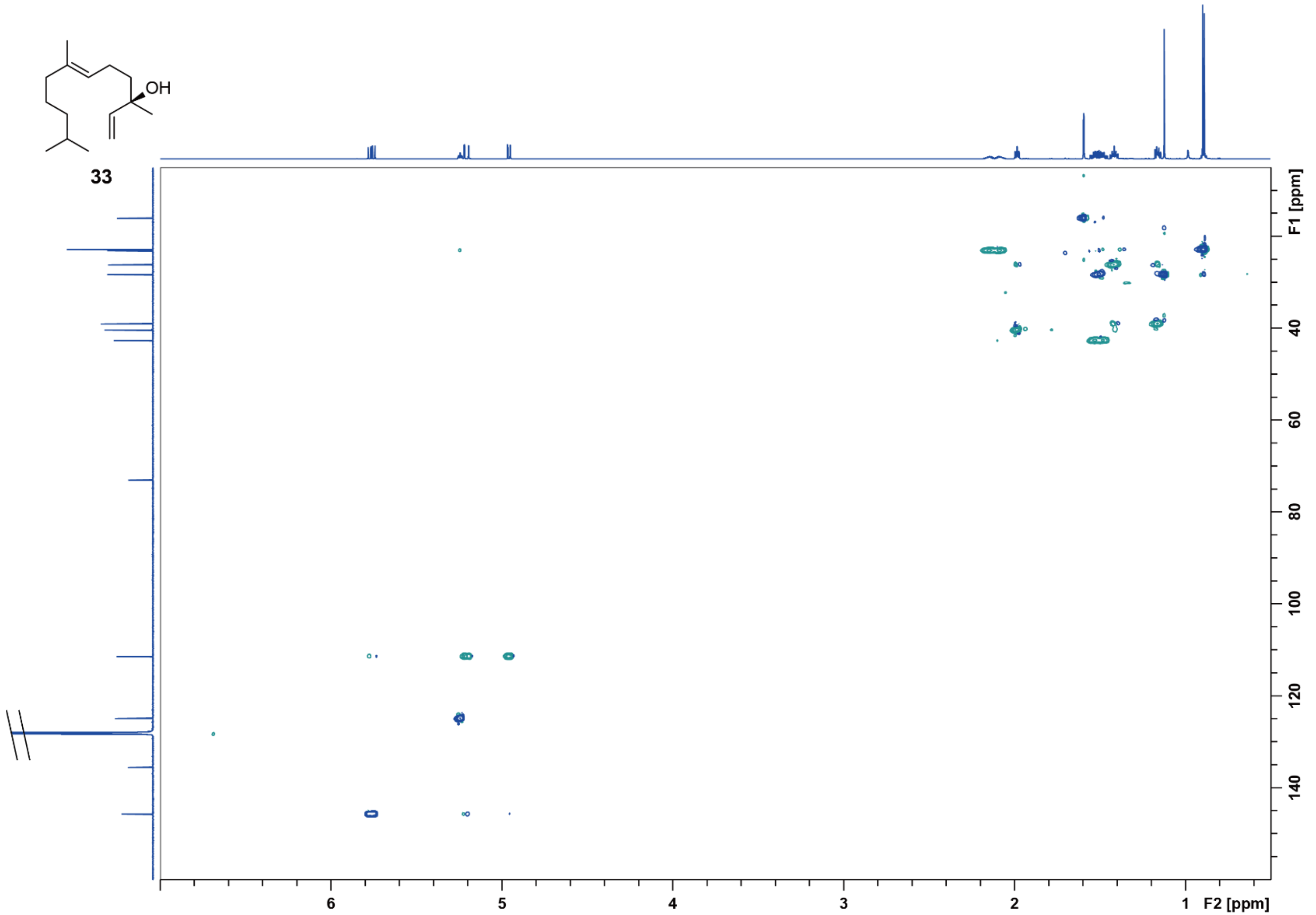

Figure S88. HSQC spectrum of compound **33** (C_6D_6).

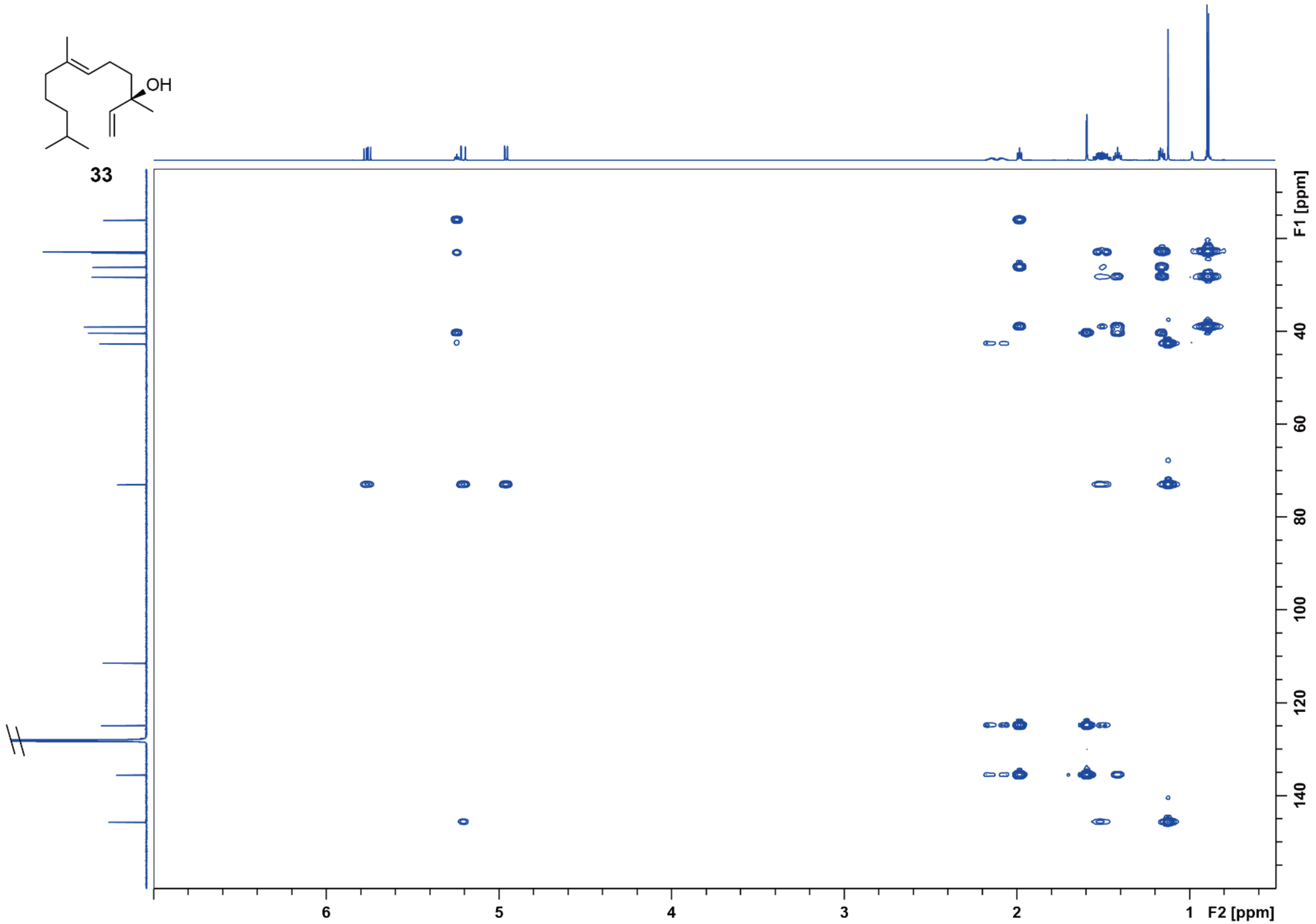

Figure S89. HMBC spectrum of compound **33** (C_6D_6).

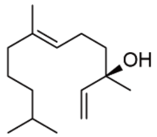

33

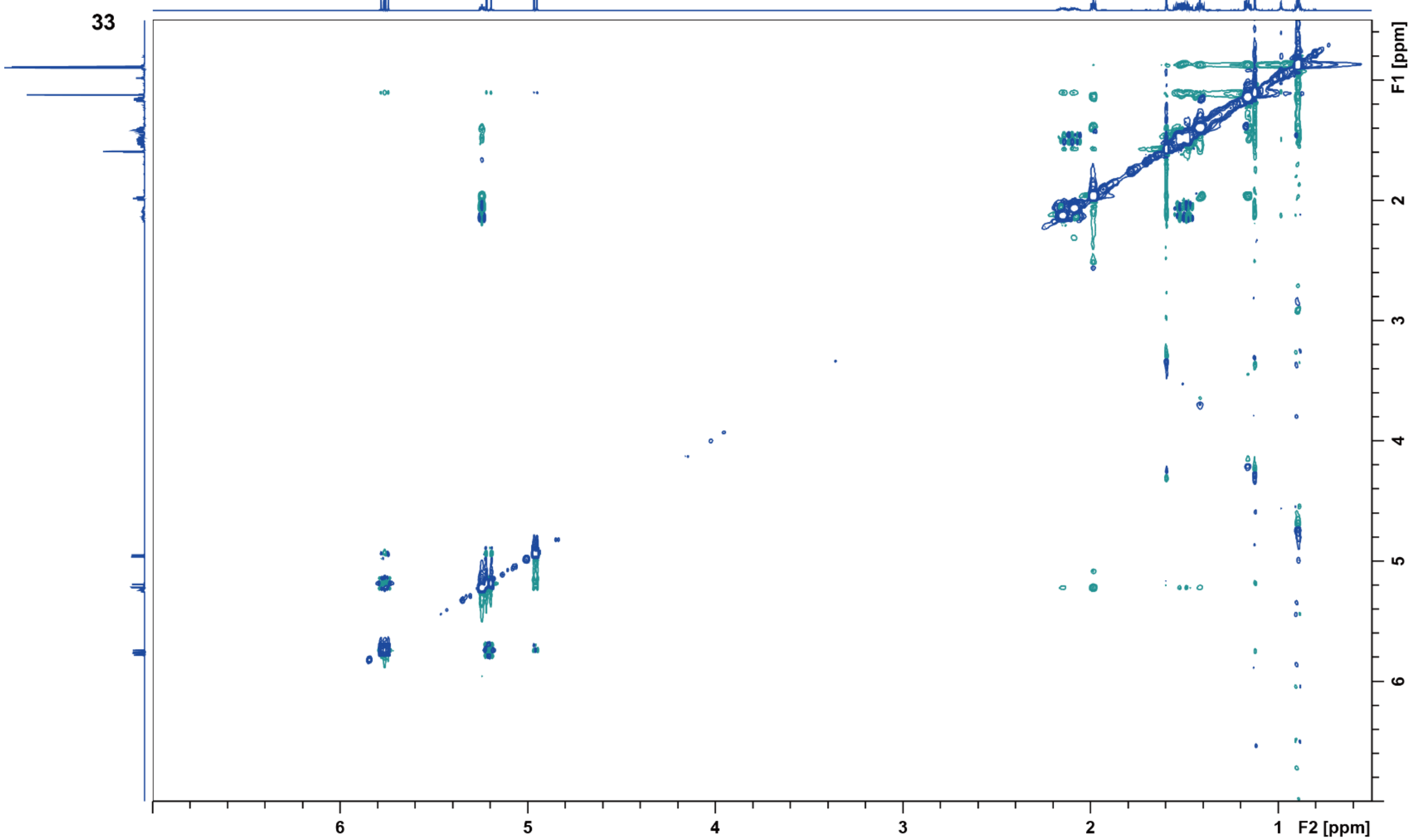

Figure S90. NOESY spectrum of compound **33** (C_6D_6, 700 MHz).

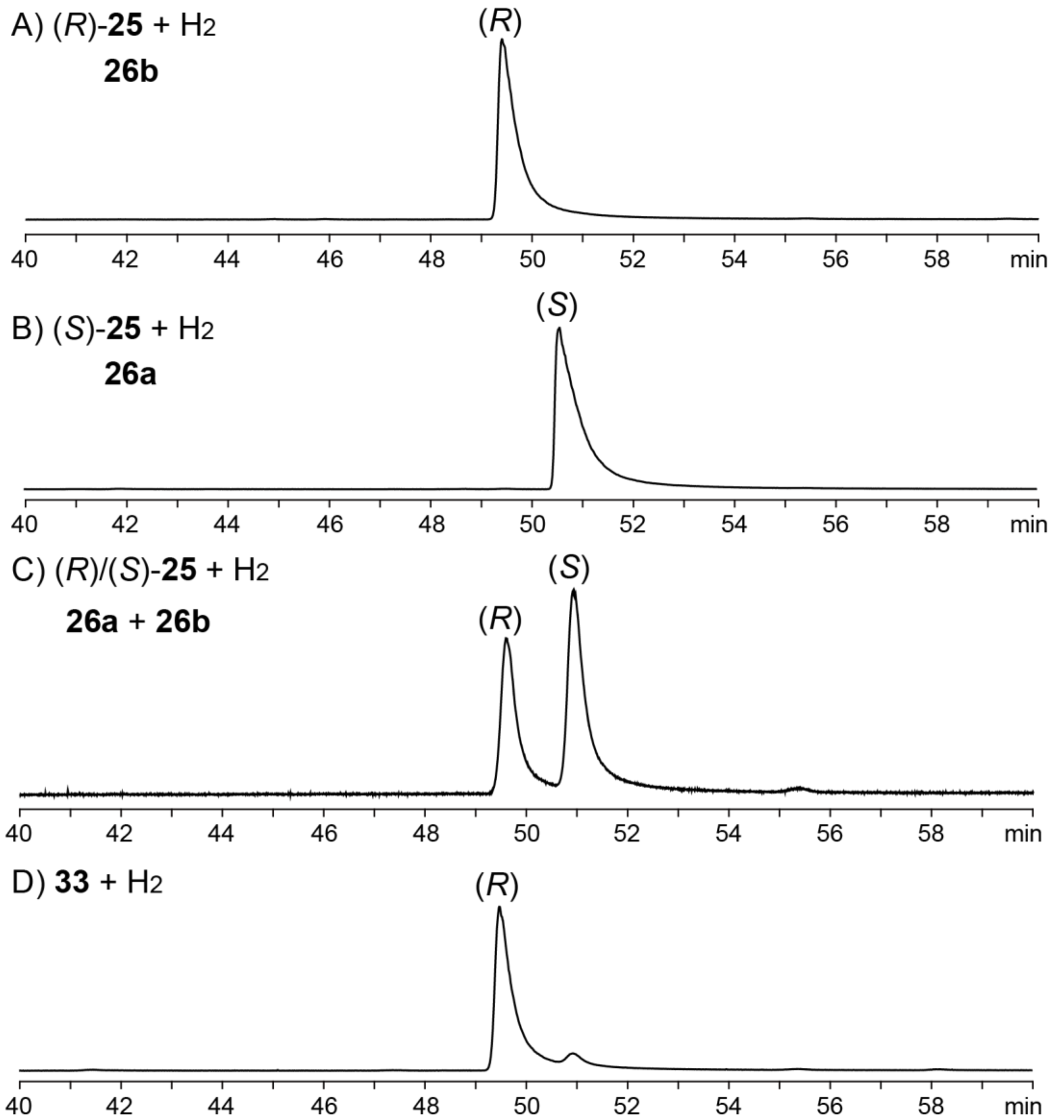

Figure S91. Determination of the absolute configuration of **33**. The gas chromatograms using a chiral stationary phase of A) the hydrogenation product **26b** from (*R*)-nerolidol (**25**), B) hydrogenation product **26a** from (*S*)-**25**, C) mixture of **26a** and **26b** and D) hydrogenation product from **33**.

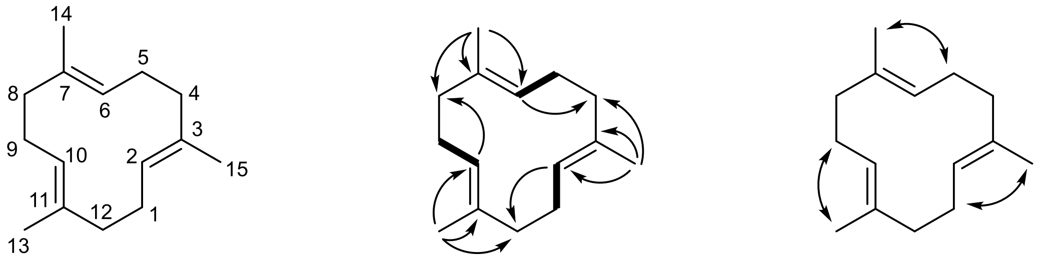

34

Figure S92. Structure elucidation of **34**. Bold: $^{1}H,^{1}H$-COSY, single headed arrows: key HMBC, and double headed arrows: key NOESY correlations.

Table S11. NMR data of **34** in C_6D_6 recorded at 298 K.

C[a]	type	^{13}C[b]	^{1}H[b]
1, 5, 9	3 x CH_2	25.60	2.09 (m, 6H)
2, 6, 10	3 x CH	126.98	4.82 (t, *J* = 7.4 Hz, 3H)
3, 7, 11	3 x C_q	133.17	–
4, 8, 12	3 x CH_2	40.23	2.05 (m, 6H)
13, 14, 15	3 x CH_3	15.21	1.43 (m, 9H)

[a] Carbon numbering as shown in Figure S92. [b] Chemical shifts δ in ppm, multiplicity: t = triplet, m = multiplet.

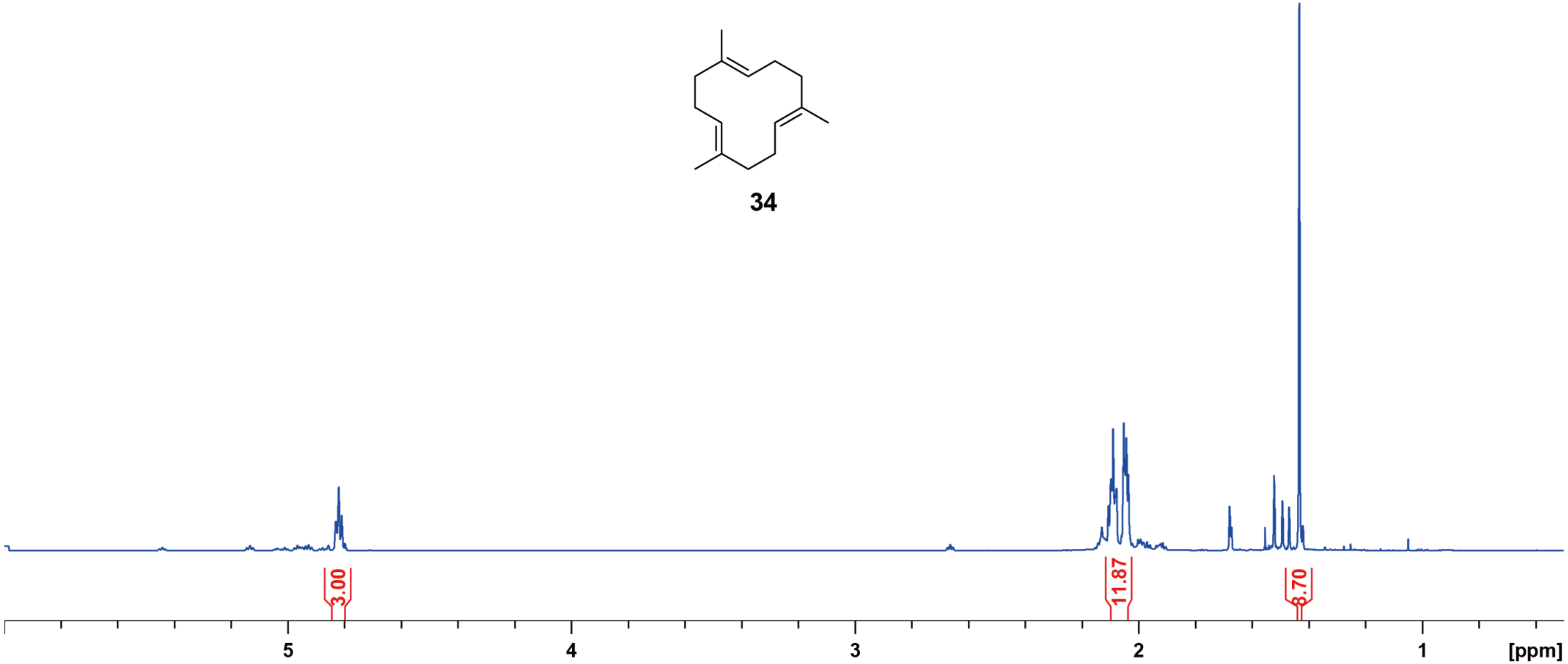

Figure S93. ^{1}H NMR spectrum of compound **34** (C_6D_6, 700 MHz).

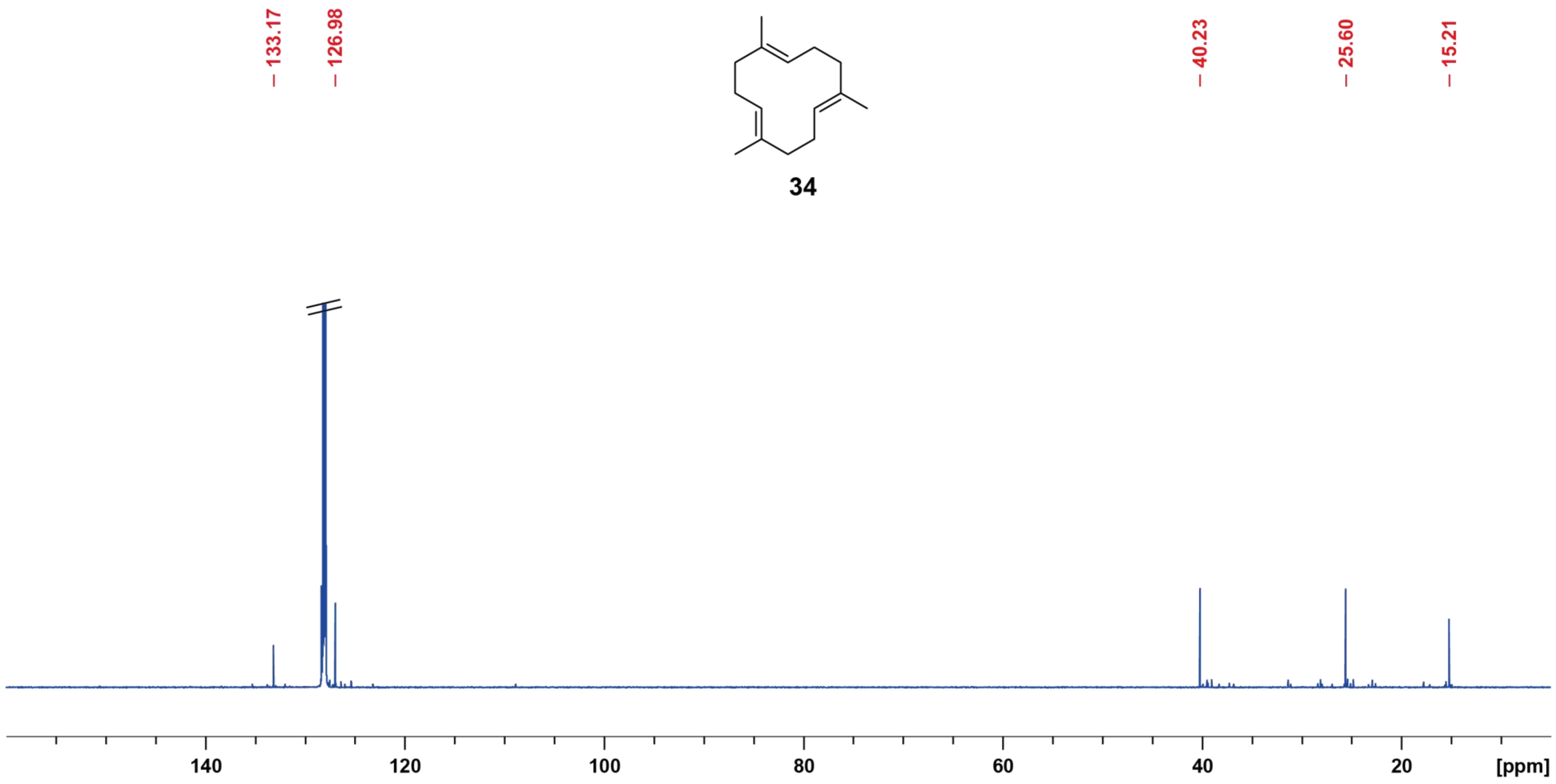

Figure S94. ^{13}C NMR spectrum of compound **34** (C_6D_6, 176 MHz).

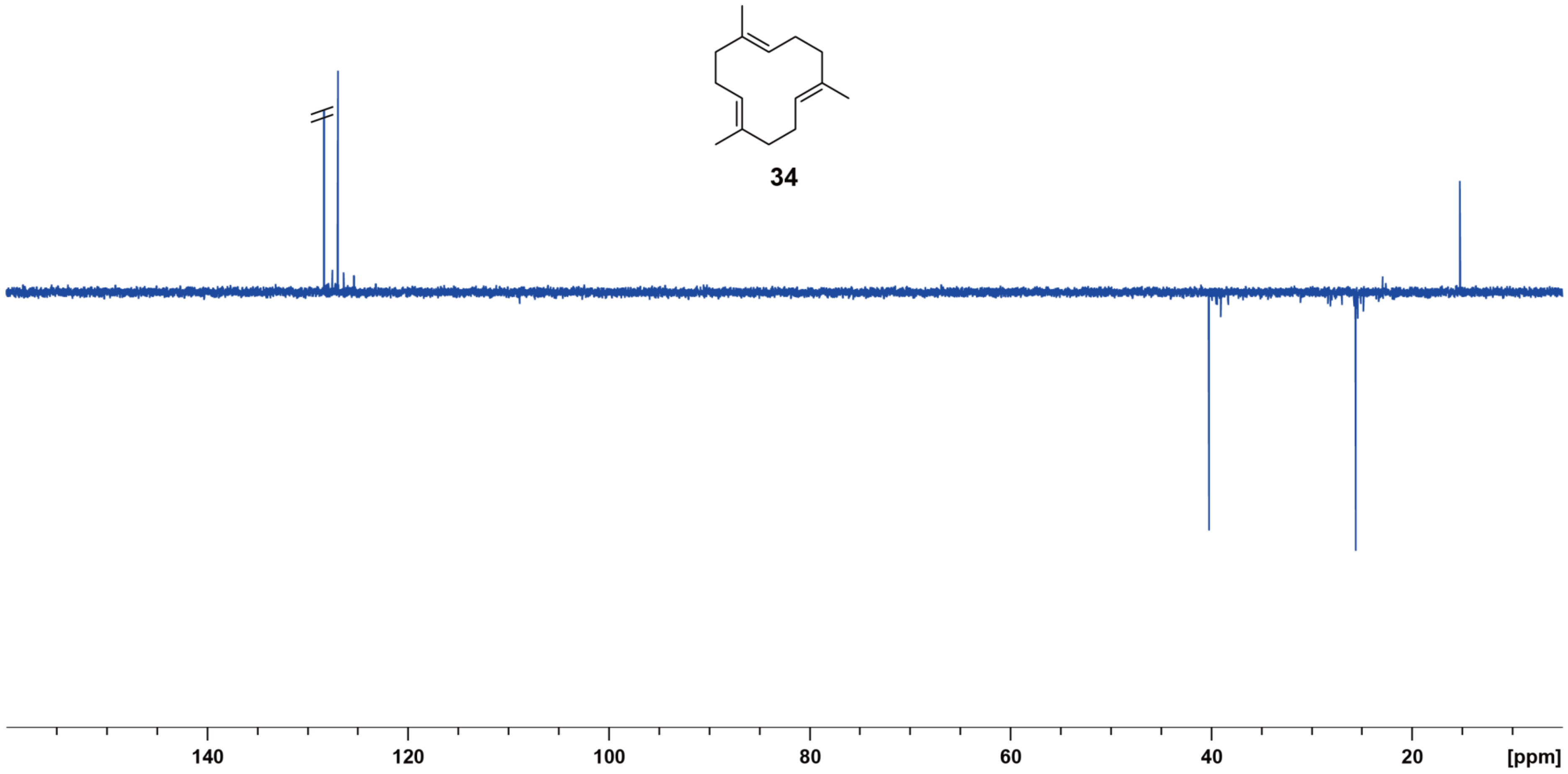

Figure S95. ^{13}C DEPT spectrum of compound **34** (C_6D_6, 176 MHz).

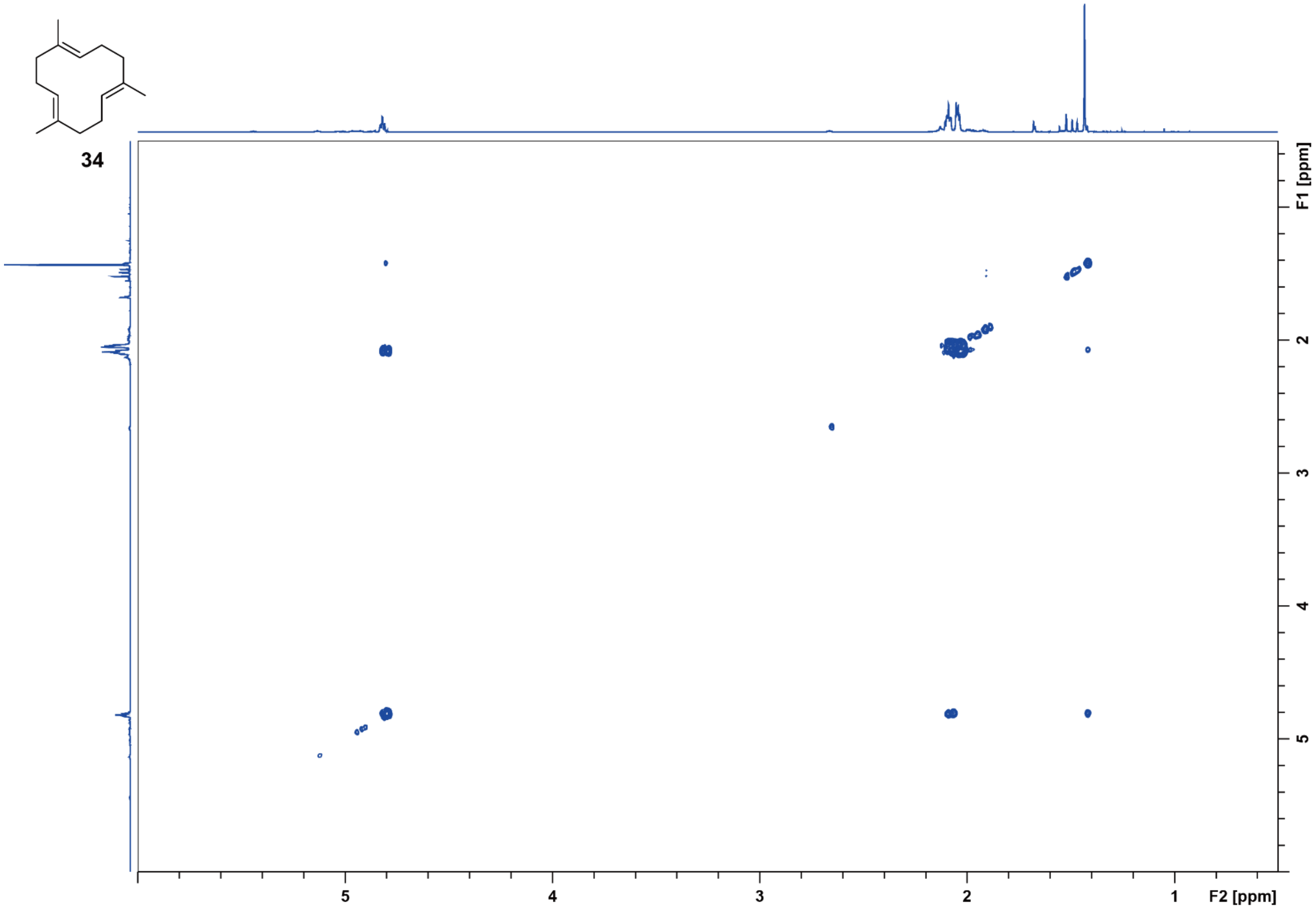

Figure S96. ^{1}H-^{1}H-COSY spectrum of compound **34** (C_6D_6, 700 MHz).

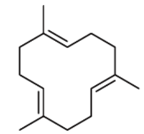

34

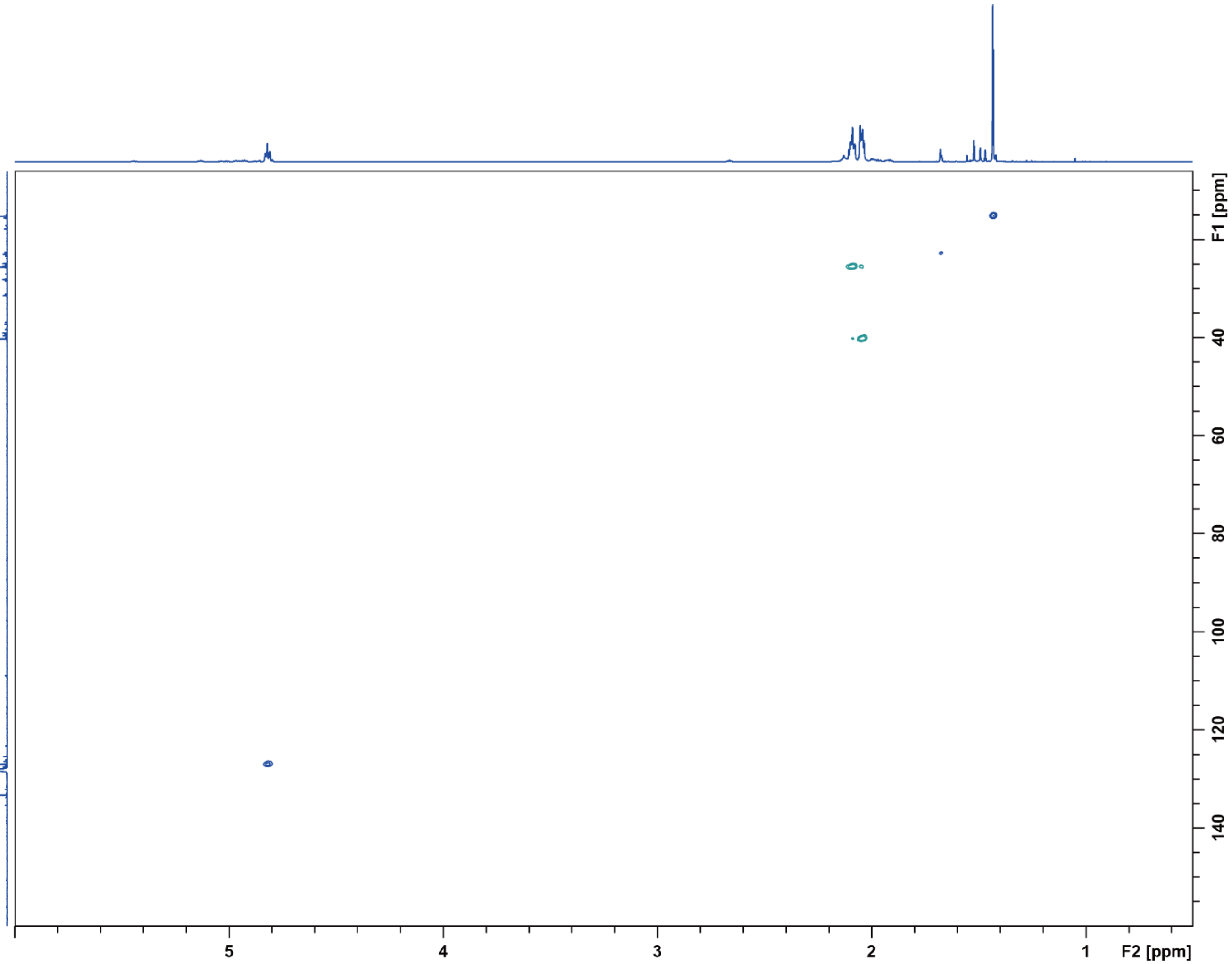

Figure S97. HSQC spectrum of compound **34** (C_6D_6).

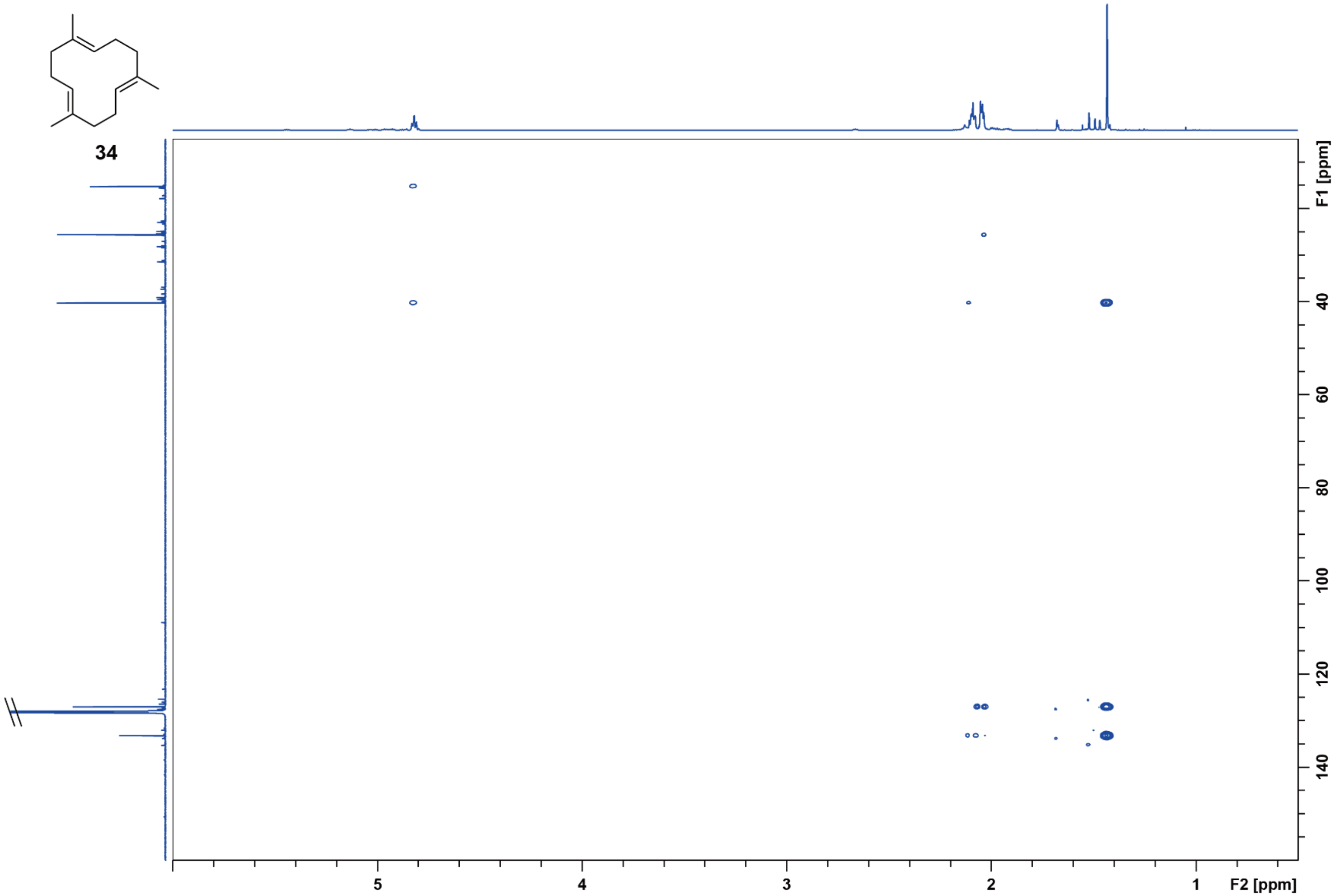

Figure S98. HMBC spectrum of compound **34** (C_6D_6).

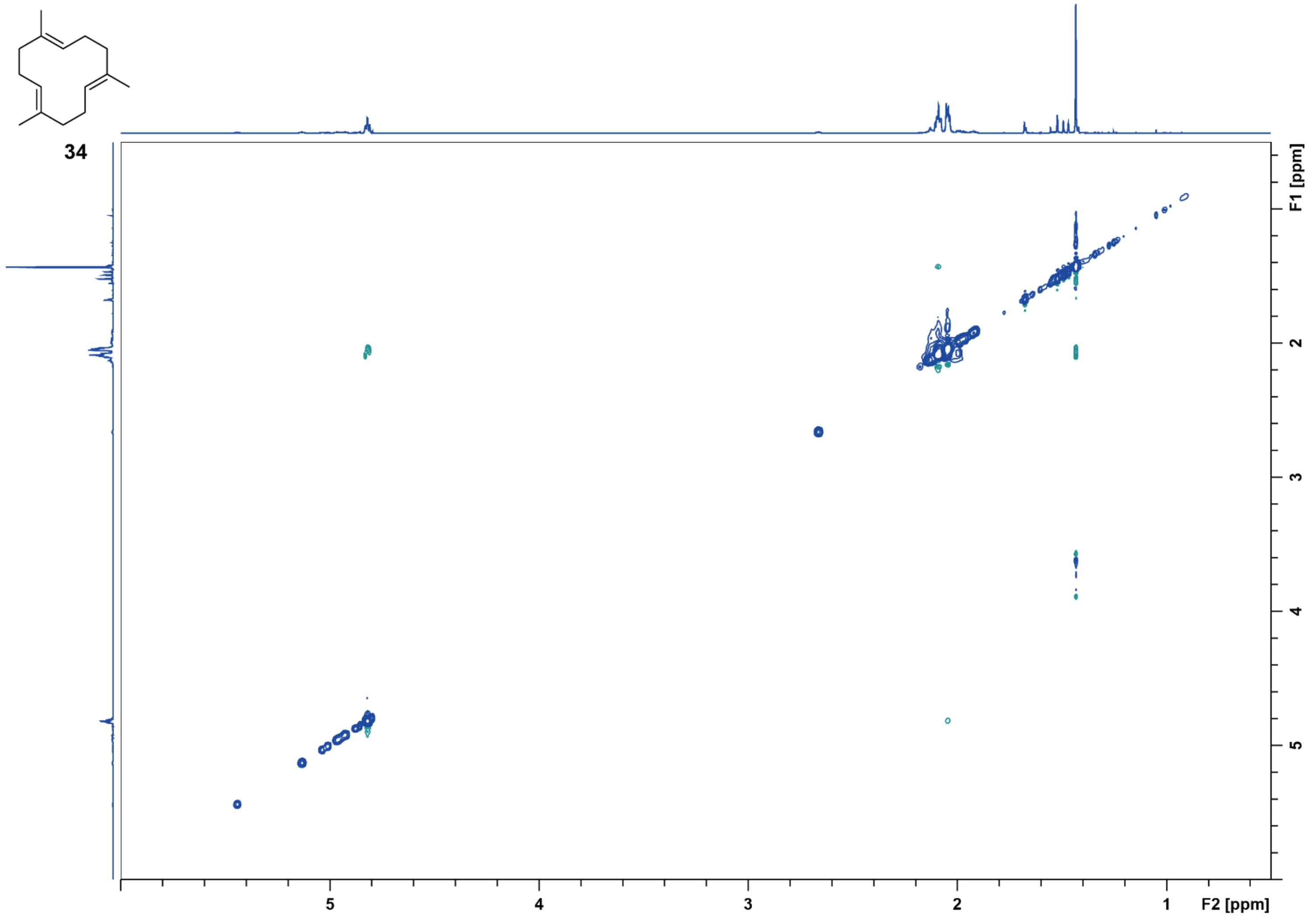

Figure S99. NOESY spectrum of compound **34** (C_6D_6, 700 MHz).

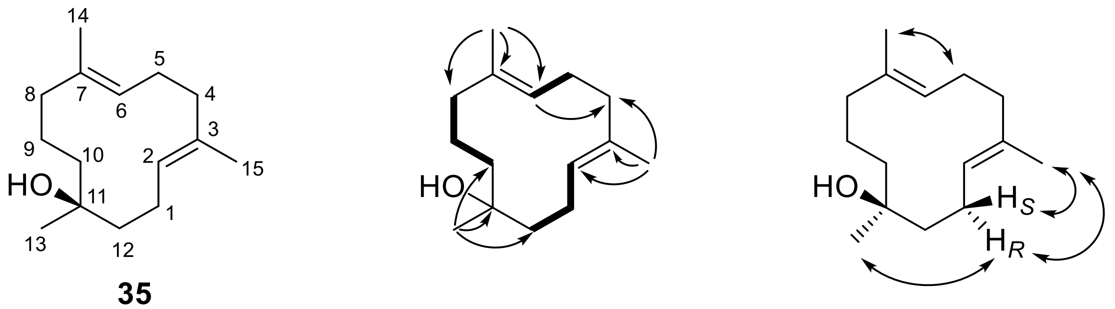

35

Figure S100. Structure elucidation of **35**. Bold: $^{1}H,^{1}H$-COSY, single headed arrows: key HMBC, and double headed arrows: key NOESY correlations.

Table S12. NMR data of **35** in C_6D_6 recorded at 298 K.

C[a]	type	^{13}C[b]	^{1}H[b]
1	CH_2	24.07	2.34 (m, 1H)
			1.94 (m, 1H)
2	CH	129.14	5.01 (t, J = 7.5 Hz, 1H)
3	C_q	132.66	–
4	CH_2	40.20	2.03 (m, 2H)
5	CH_2	25.86	2.17 (m, 1H)
			2.10 (m, 1H)
6	CH	126.90	4.86 (t, J = 7.6 Hz, 1H)
7	C_q	134.16	–
8	CH_2	38.97	1.92 (m, 2H)
9	CH_2	21.83	1.32 (m, 1H)
			1.21 (m, 1H)
10	CH_2	38.38	1.44 (m, 1H)
			1.34 (m, 1H)
11	C_q	72.46	–
12	CH_2	40.57	1.50 (m, 1H)
			1.40 (m, 1H)
13	CH_3	28.88	1.04 (s, 3H)
14	CH_3	17.00	1.50 (s, 3H)
15	CH_3	15.12	1.47 (s, 3H)

[a] Carbon numbering as shown in Figure S100. [b] Chemical shifts δ in ppm, multiplicity: s = singlet, t = triplet, m = multiplet.

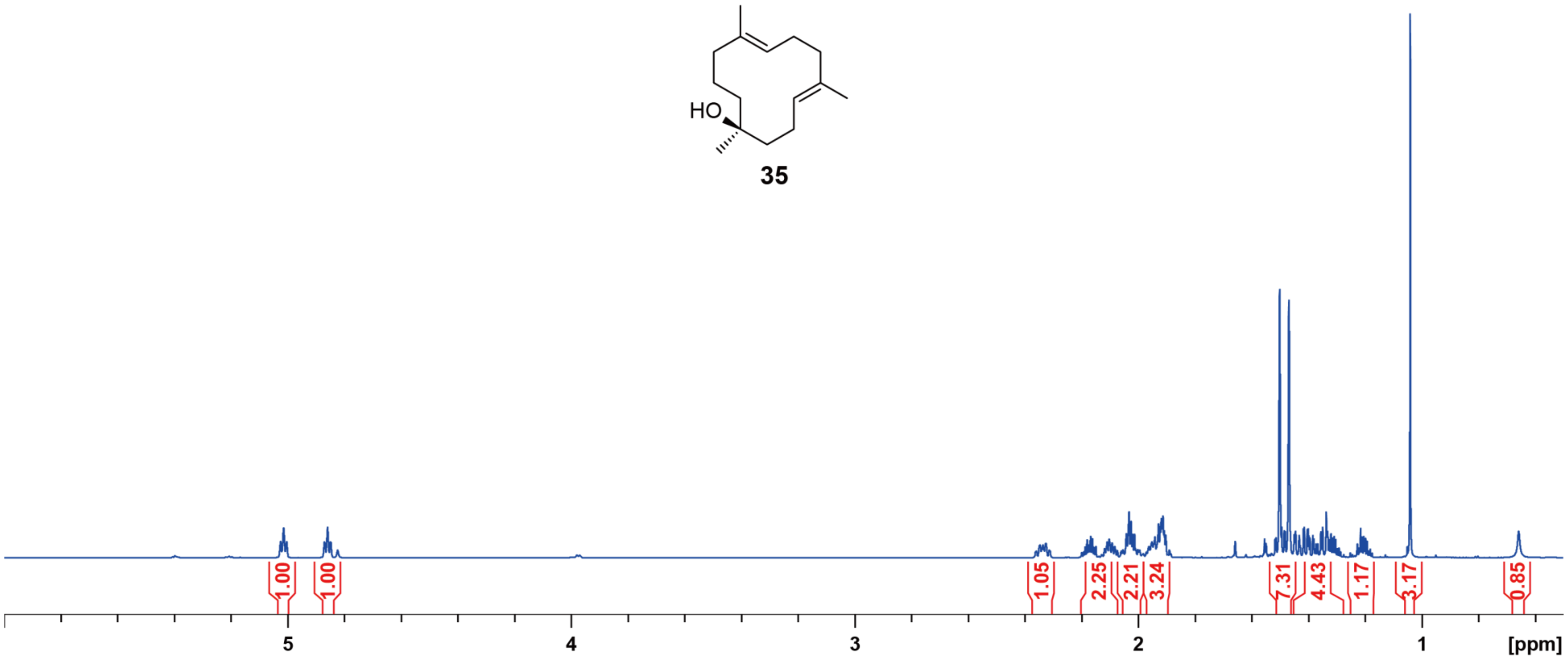

Figure S101. ^{1}H NMR spectrum of compound **35** (C_6D_6, 700 MHz).

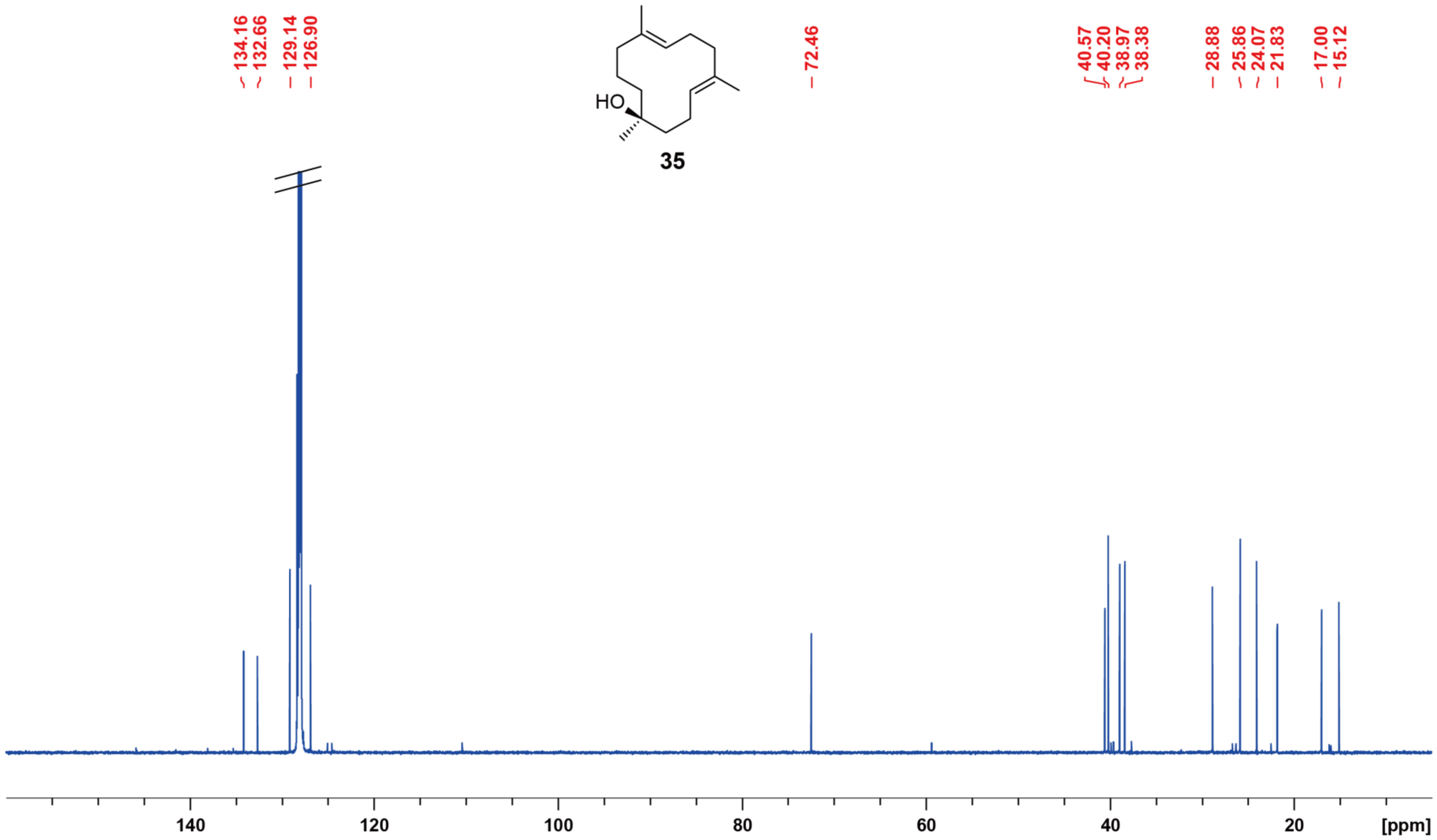

Figure S102. ^{13}C NMR spectrum of compound **35** (C_6D_6, 176 MHz).

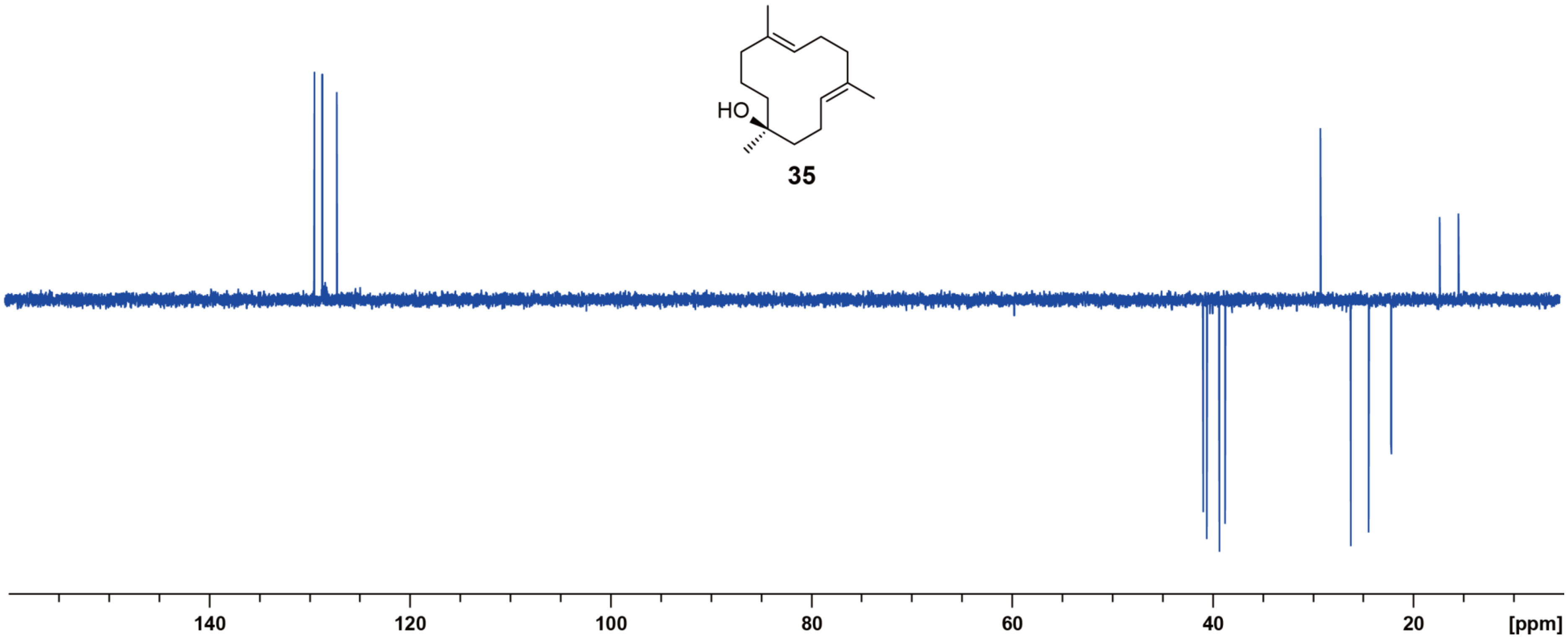

Figure S103. ^{13}C DEPT spectrum of compound **35** (C_6D_6, 176 MHz).

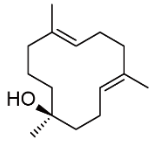

35

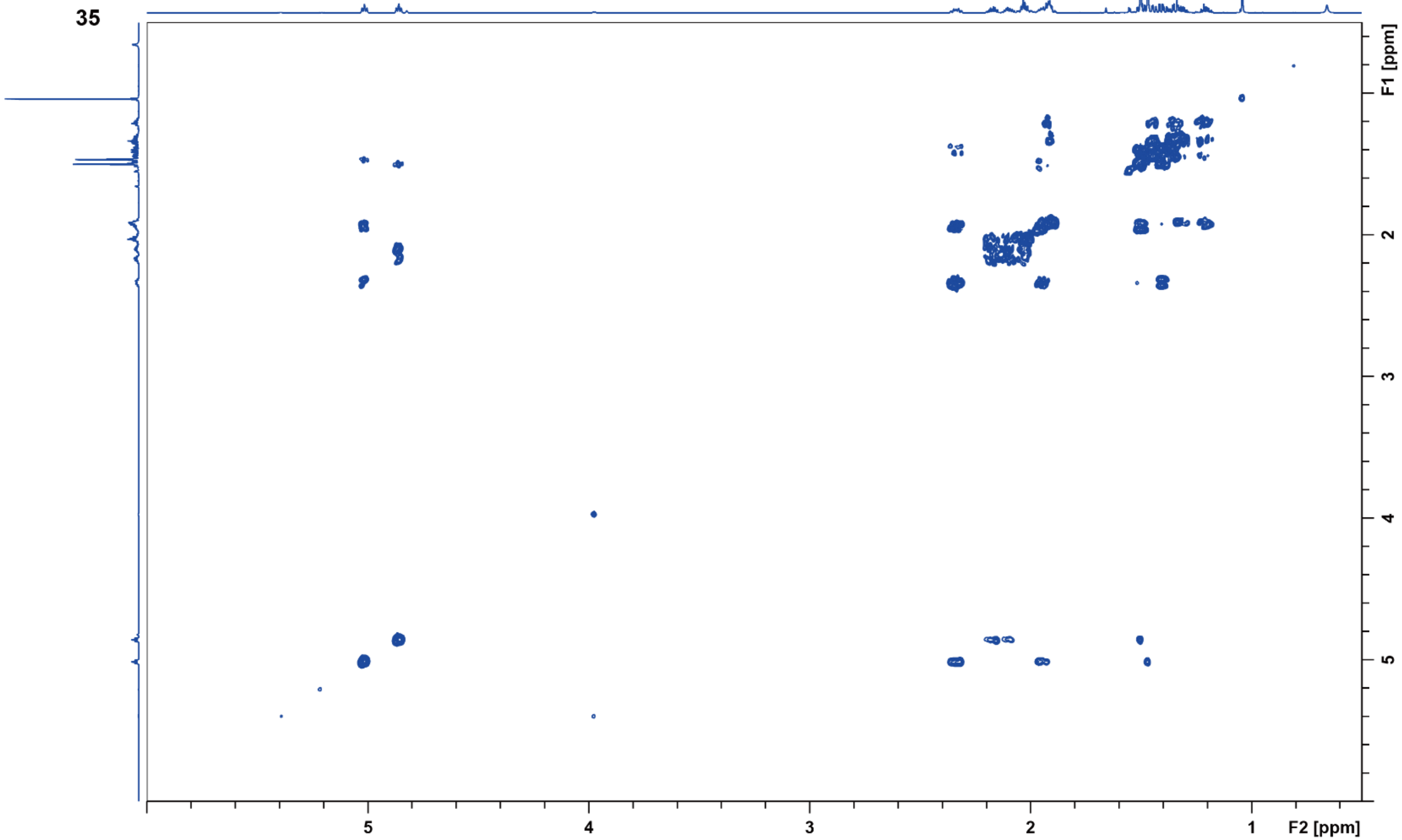

Figure S104. ^{1}H-^{1}H-COSY spectrum of compound **35** (C_6D_6, 700 MHz).

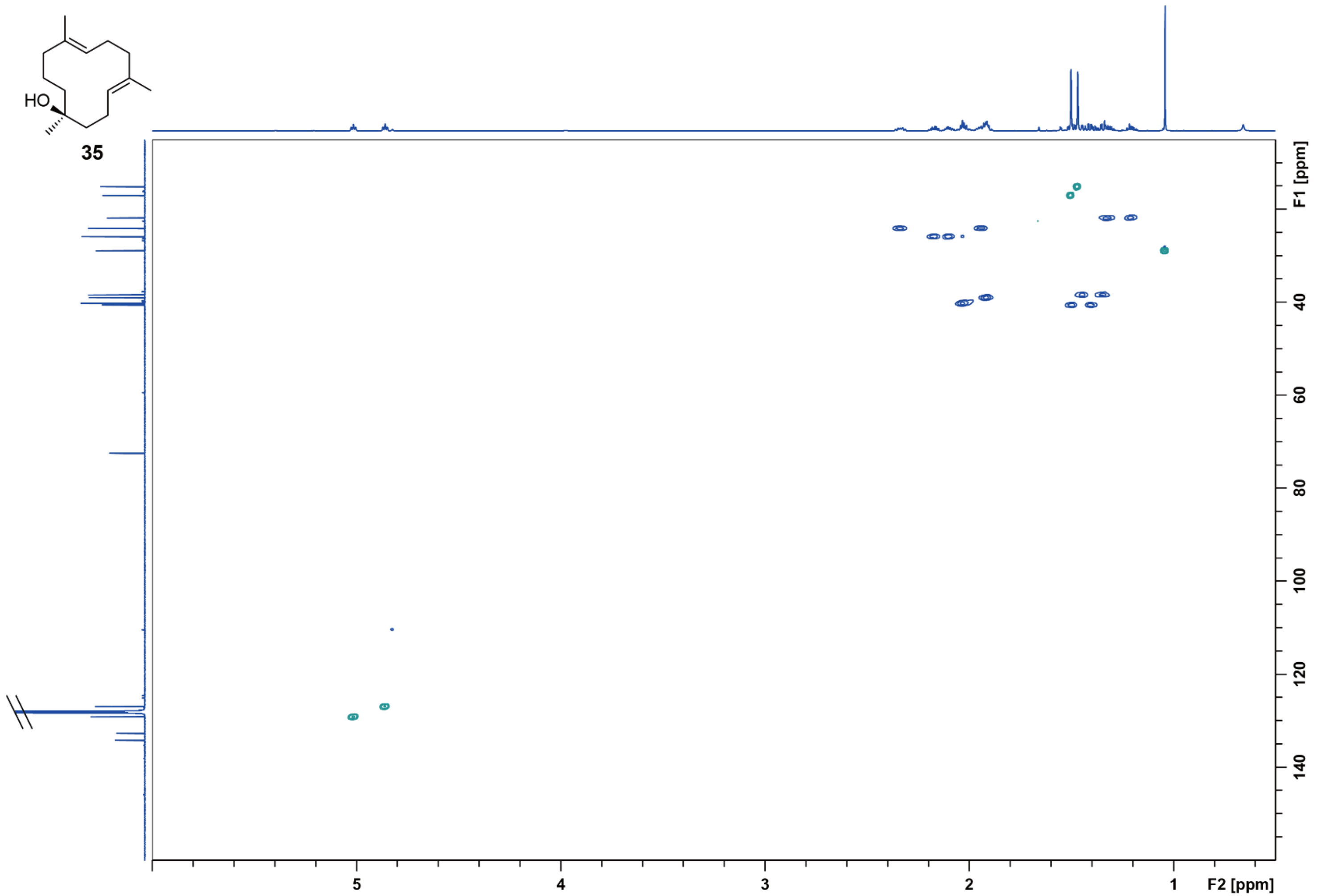

Figure S105. HSQC spectrum of compound **35** (C_6D_6).

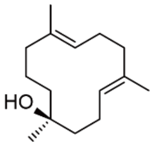

35

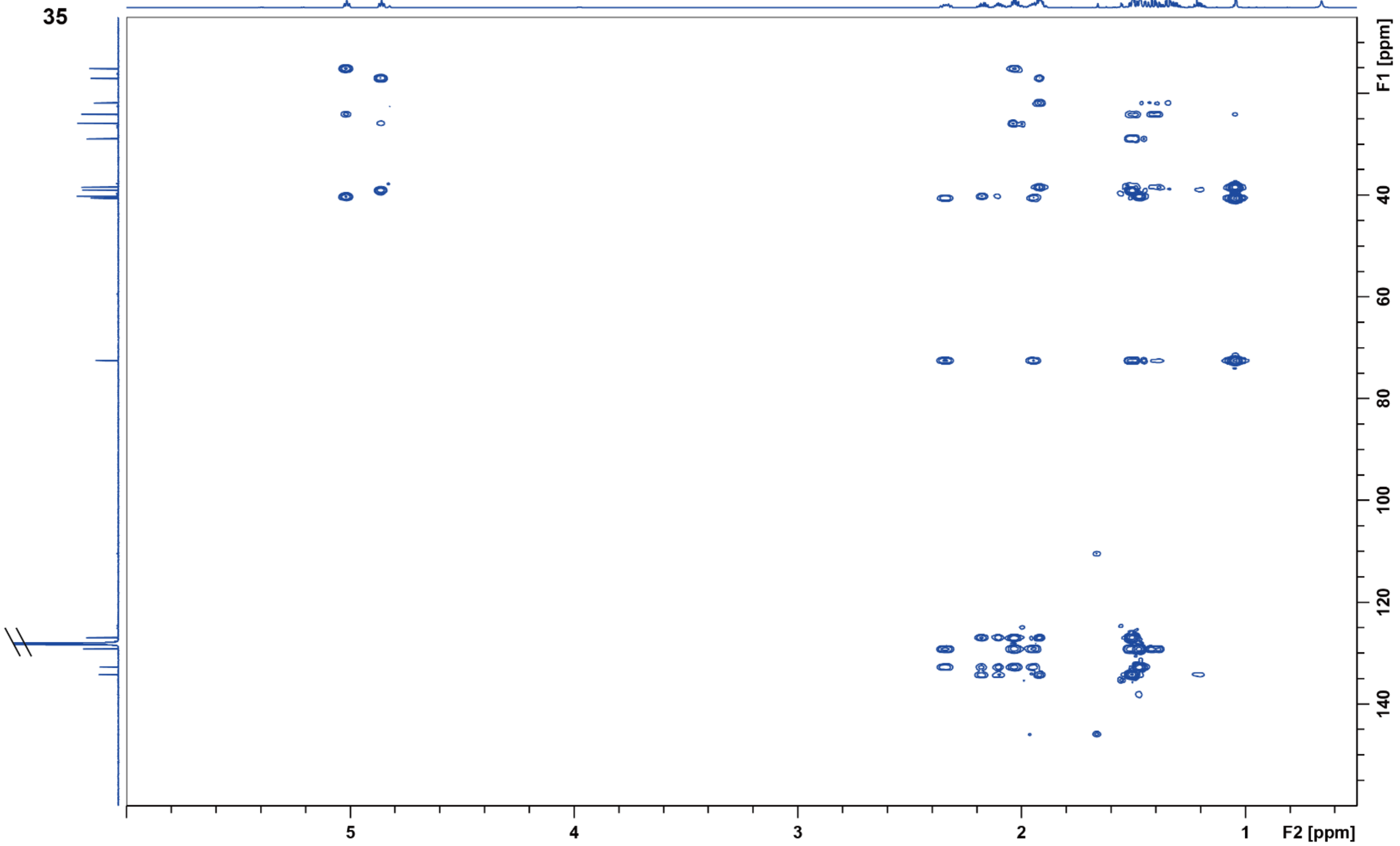

Figure S106. HMBC spectrum of compound **35** (C_6D_6).

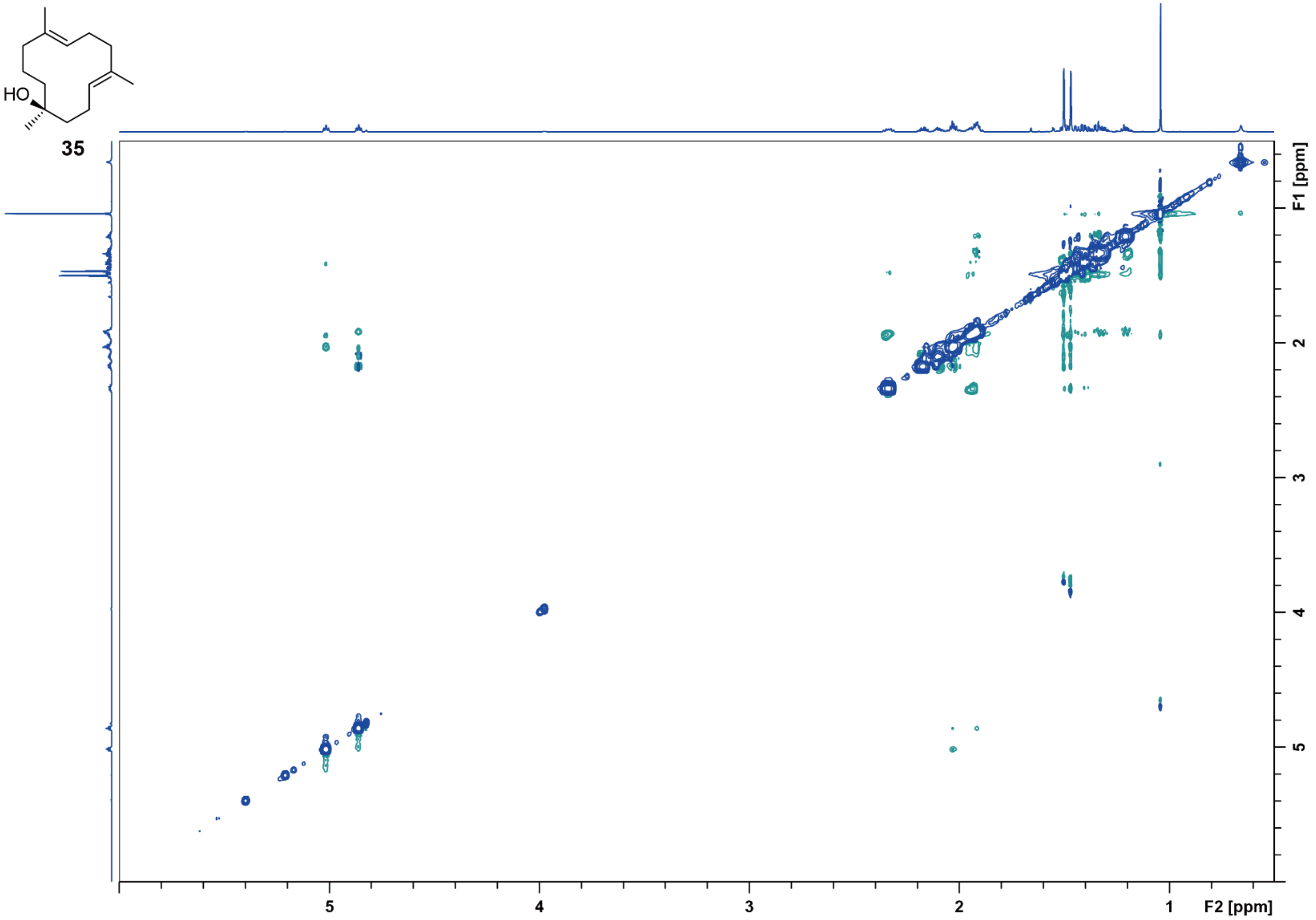

Figure S107. NOESY spectrum of compound **35** (C_6D_6, 700 MHz).

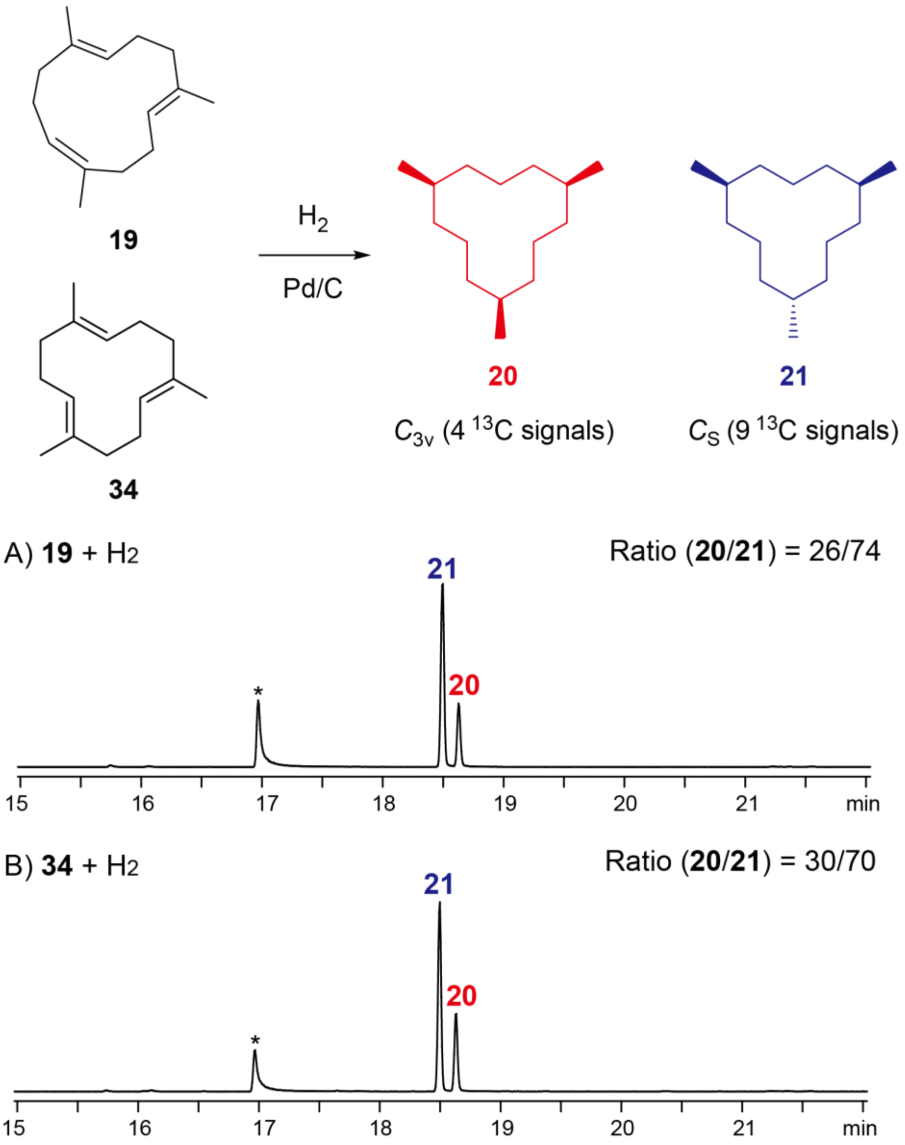

Figure S108. Total ion chromatograms of hydrogenation products from A) **19** and B) **34**. The asterisk indicates an impurity from the solvent.

Labelling experiments

The protein solution of SmTS6 (1 mL) and FPPS (1 mL), Tris buffer (3 mL; 50 mM Tris, 1 mM $MgCl_2$, pH = 7.6), incubation buffer (5 mL; 50 mM Tris, 10 mM $MgCl_2$, 20% glycerol, pH = 7.6), GPP analogue **36** (1 mL; 1 mg/mL in 25 mM aqueous NH_4HCO_3) and (*R*)- or (*S*)-(1-^{13}C,1-^{2}H)IPP were mixed. The mixture was incubated at 28 °C over night. The product was extracted with C_6D_6 (600 μL + 300 μL) and then subject to NMR for analysis.

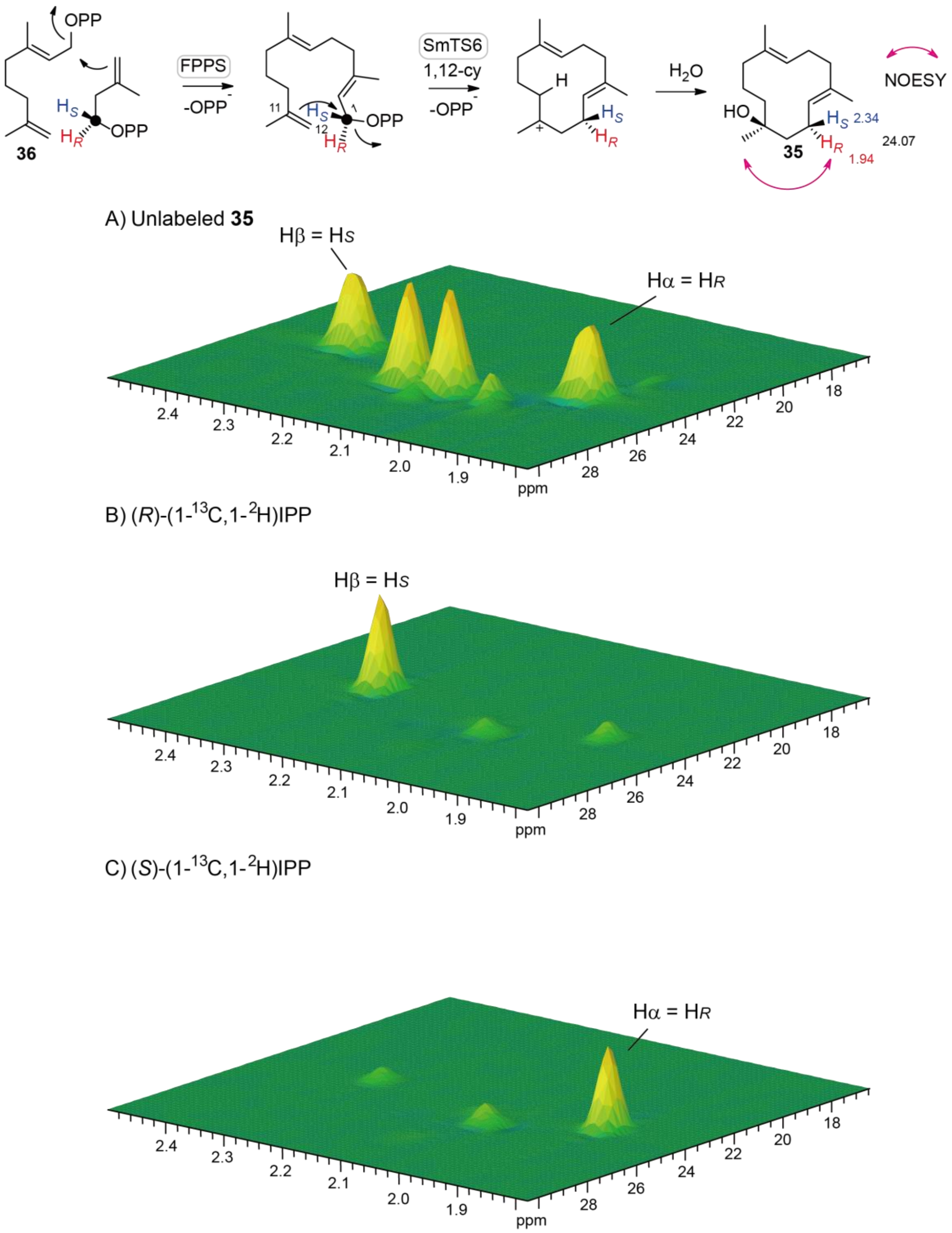

Figure S109. Determination of the absolute configuration of **35** by labelling experiments. Partial HSQC spectrum of A) unlabeled **35**, B) labeled **35** obtained from **36** with (*R*)-(1-^{13}C,1-^{2}H)IPP and C) labelled **35** obtained from **36** with (*S*)-(1-^{13}C,1-^{2}H)IPP.

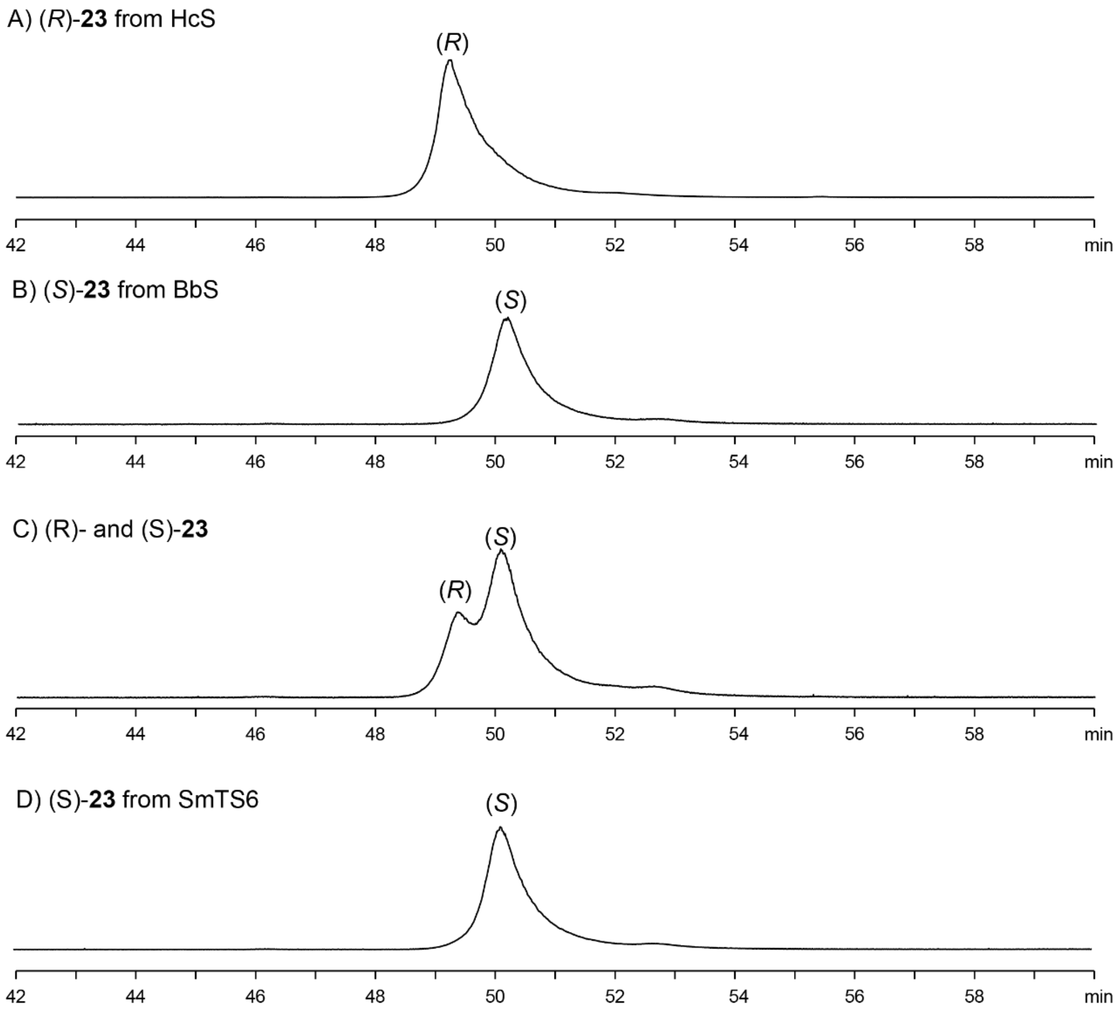

Figure S110. Determination of the absolute configuration of **23** from SmTS6. The gas chromatograms using a chiral stationary phase of A) (*R*)-**23** from HcS, B) (*S*)-**23** from BbS, C) mixture of (*R*)- and (*S*)-**23** and D) (*S*)-**23** from SmTS6.

References

[1] L. Lauterbach, A. Hou, J. S. Dickschat, *Chem. Eur. J.* **2021**, *27*, 7923-7929.
[2] J. Rinkel, J. S. Dickschat, *Beilstein J. Org. Chem.* **2019**, *15*, 1008-1019.
[3] J. Rinkel, J. S. Dickschat, *Beilstein J. Org. Chem.* **2019**, *15*, 789-794.
[4] A. Hou, J. S. Dickschat, *Angew. Chem. Int. Ed.* **2020**, *59*, 19961-19965.
